# Genome-wide maps of ribosomal occupancy provide insights into adaptive evolution and regulatory roles of uORFs during *Drosophila* development

**DOI:** 10.1371/journal.pbio.2003903

**Published:** 2018-07-20

**Authors:** Hong Zhang, Shengqian Dou, Feng He, Junjie Luo, Liping Wei, Jian Lu

**Affiliations:** 1 State Key Laboratory of Protein and Plant Gene Research, Center for Bioinformatics, School of Life Sciences, Peking University, Beijing, China; 2 Peking-Tsinghua Center for Life Sciences, Peking University, Beijing, China; University of California, Riverside, United States of America

## Abstract

Upstream open reading frames (uORFs) play important roles in regulating the main coding DNA sequences (CDSs) via translational repression. Despite their prevalence in the genomes, uORFs are overall discriminated against by natural selection. However, it remains unclear why in the genomes there are so many uORFs more conserved than expected under the assumption of neutral evolution. Here, we generated genome-wide maps of translational efficiency (TE) at the codon level throughout the life cycle of *Drosophila melanogaster*. We identified 35,735 uORFs that were expressed, and 32,224 (90.2%) of them showed evidence of ribosome occupancy during *Drosophila* development. The ribosome occupancy of uORFs is determined by genomic features, such as optimized sequence contexts around their start codons, a shorter distance to CDSs, and higher coding potentials. Our population genomic analysis suggests the segregating mutations that create or disrupt uORFs are overall deleterious in *D*. *melanogaster*. However, we found for the first time that many (68.3% of) newly fixed uORFs that are associated with ribosomes in *D*. *melanogaster* are driven by positive Darwinian selection. Our findings also suggest that uORFs play a vital role in controlling the translational program in *Drosophila*. Moreover, we found that many uORFs are transcribed or translated in a developmental stage-, sex-, or tissue-specific manner, suggesting that selective transcription or translation of uORFs could potentially modulate the TE of the downstream CDSs during *Drosophila* development.

## Author summary

Upstream open reading frames (uORFs) in the 5′ untranslated regions (UTRs) of messenger RNAs can potentially inhibit translation of the downstream regions that encode proteins by sequestering protein-making machinery the ribosome. Moreover, mutations that destroy existing uORFs or create new ones are known to cause human disease. Although mutations that create new uORFs are generally deleterious and are selected against, many uORFs are evolutionarily conserved across eukaryotic species. To resolve this dilemma, we used extensive mRNA-Seq and ribosome profiling to generate high-resolution genome-wide maps of ribosome occupancy and translational efficiency (TE) during the life cycle of the fruit fly *D*. *melanogaster*. This allowed us to identify the sequence features of uORFs that influence their ability to associate with ribosomes. We demonstrate for the first time that the majority of the newly fixed uORFs in *D*. *melanogaster*, especially the translated ones, are under positive Darwinian selection. We also show that uORFs exert widespread repressive effects on the translation of the downstream protein-coding region. We find that many uORFs are transcribed or translated in a developmental stage-, sex-, or tissue-specific manner. Our results suggest that during *Drosophila* development, changes in the TE of uORFs, as well as the inclusion/exclusion of uORFs, are frequently exploited to inversely influence the translation of the downstream protein-coding regions. Our study provides novel insights into the molecular mechanisms and functional consequences of uORF-mediated regulation.

## Introduction

Eukaryotic protein translation is highly regulated to ensure that proteins are produced from the coding DNA sequences (CDSs) in a controlled manner [1, 2]. In eukaryotic cap-dependent translation initiation, the 43S preinitiation complex (PIC) first binds near the 5′ cap of an mRNA, scans through the 5′ untranslated region (UTR), and associates with a 60S subunit to assemble into a ribosome to commence translation when the PIC encounters an AUG start codon [3]. Upstream open reading frames (uORFs), which are located in the 5′ UTRs and upstream of the AUG start codons of CDSs (cAUGs), are important in regulating translation initiations of CDSs [4–28]. When a PIC encounters a uORF, it either scans through or initiates translation of that uORF. Once initiating translation of a uORF, the PIC might drop off or stall at the stop codon of that uORF (both of which might trigger nonsense-mediated mRNA decay) [12, 15, 29]; alternatively, the PIC can reinitiate translation of the downstream CDS, and the reinitiation process reduces the translational rate (i.e., repress translation) of the CDS [11, 30–33]. The recently developed ribosome profiling (also known as Ribo-Seq) technique [13, 34] has further advanced our understanding of the regulatory roles of uORFs in translational regulation. Studies performed in yeasts, zebrafish, and mammals have systematically demonstrated how genomic features of uORFs, such as conservation levels and sequence contexts, affect the repressiveness of uORFs on the translation of CDSs [10, 13, 14, 20, 22, 34–39]. Overall, these studies have broadened our view of the genome-wide features of uORFs in modulating protein translation.

Although uORFs are prevalent in eukaryotic genomes [21, 40–42], the observed uORFs in the 5′ UTRs are significantly fewer than expected by chance in a wide range of species, presumably because new uORFs disturb normal protein translation and are hence selected against [41, 43–46]. On the other hand, the uORFs preserved in the genomes are usually evolutionarily more conserved than expected under the assumption of neutral evolution [10, 22, 43, 46, 47], suggesting those uORFs are maintained by functional constraints. The two different modes of purifying selection on uORFs are well manifested in human populations, in which both the mutations that create new uORFs or disrupt preexisting uORFs can cause diseases [15, 19, 21, 48, 49]. For example, a point mutation introducing a uORF in the 5′ UTR of *CDKN2A* decreases cyclin-dependent kinase inhibitor 2A (CDKN2A) protein level and causes melanoma [50, 51]. Similarly, creating a new uORF by a point mutation in *SRY* reduces translation of *SRY* mRNA and leads to gonadal dysgenesis [52]. On the other hand, eliminating a uORF in *THPO* mRNA increases translation of the downstream CDS and causes thrombocythemia [53]. In summary, these seemingly contradictory observations suggest further studies are needed to understand the evolutionary forces that have shaped uORFs at a genome-wide level.

If most new uORFs are deleterious and selected against, why are there so many uORFs maintained in the genomes by natural selection during evolution? Population genetics suggests that slightly deleterious mutations can be fixed due to genetic drift [54, 55]. Hence, many uORFs might be neutral (or slightly deleterious) but drift to fixation [56], given the weak repressive effects performed by uORFs on CDSs [10, 16, 21, 22]. However, if this hypothesis is correct, it is hard to explain why most uORFs are preserved by natural selection during evolution. Previous studies have proposed uORFs might serve adaptive functions by fine-tuning cellular or developmental processes [15, 27, 30, 34, 57–60]. Nevertheless, the evolutionary genetic evidence to support the adaptive evolution of uORFs is currently lacking. Therefore, many unaddressed gaps remain in our understanding of the evolutionary principles of uORFs. Since the functional uORFs might experience distinct evolutionary forces compared to the random (neutral) uORFs in the 5′ UTRs, to address these questions, we have to combine evolutionary analysis and the functional genomic studies.

In this study, we constructed high-resolution genome-wide maps of uORF ribosome occupancy in the major developmental stages of *Drosophila melanogaster* with extensive mRNA-Seq and Ribo-Seq experiments. These data, together with an analysis of the genomic features and evolutionary patterns, enable us to discover that many of the newly fixed uORFs in *D*. *melanogaster* are driven by positive selection, especially for those associated with ribosomes. We also present evidence that ribosome-associated uORFs exert widespread inhibitory effects on CDSs and modulate translation during *Drosophila* development.

## Results

### uORF ribosome occupancy and translation revealed by genome-wide mRNA-Seq and Ribo-Seq data

We annotated the canonical uORFs (beginning with AUG and ending with a stop codon UAA/UAG/UGA) in the protein-coding genes of *D*. *melanogaster* and identified 37,619 candidate uORFs (with a median length of 39 nt) that were supported by the published mRNA-Seq and cap analysis of gene expression (CAGE)-Seq data from the modENCODE project [61–63] (S1 and S2 Tables; Materials and methods). Of these uORFs, 29,624 were detected if we only considered the longest transcript of each gene, significantly lower (*P* < 0.001) than the number of uORFs obtained by randomly shuffling the 5′ UTR sequences (the mean is 51,942 [95% CI 51,642–52,241], Materials and methods). This comparison is consistent with previous results that the uORFs are overall deleterious and selected against [41, 43–46]. To investigate how many uORFs show evidence of ribosome occupancy, we carried out mRNA-Seq (measuring mRNA abundances) and Ribo-Seq (measuring abundances of ribosome-protected mRNA fragments [RPFs]) experiments using samples from the ISO-1 strain of *D*. *melanogaster* at the following stages: embryos at 0–2 h, 2–6 h, 6–12 h, and 12–24 h old; third-instar larvae; stage P7–8 pupae; female heads; male heads; adult female bodies (heads removed); male bodies; and *Drosophila* S2 cells (Materials and methods). The Ribo-Seq procedures were performed following Dunn and colleagues [64], with modifications (see S1 Text, S3 Table, and S1 Fig for detailed information). To cover more developmental stages, we also analyzed the mRNA-Seq and Ribo-Seq data of mature oocytes of *D*. *melanogaster* generated in a previous study [65] (Table 1).

**Table 1:** Summary of the mRNA-Seq and Ribo-Seq results.

Sample	Uniquely mapped reads (M)		Expressed genes			Expressed uORFs		
	mRNA-Seq	Ribo-Seq	Total	TE_CDS_ ≥ 0.1 (%)	TE_CDS_ ≥ 0.5 (%)	Total	TE_uORF_ ≥ 0.1 (%)	TE_uORF_ ≥ 0.5 (%)
Mature oocytes*	13.6	9.82	6,737	6,309 (93.7)	4,187 (62.1)	14,505	11,058 (76.2)	9,939 (68.5)
0–2 h embryos	49.1	47.0	7,897	7,385 (93.5)	5,033 (63.7)	17,603	14,271 (81.1)	10,031 (57.0)
2–6 h embryos	48.6	28.7	8,427	8,247 (97.9)	7,023 (83.3)	18,730	16,296 (87.0)	14,125 (75.4)
6–12 h embryos	41.6	40.2	8,994	8,796 (97.8)	7,798 (86.7)	21,601	17,066 (79.0)	11,724 (54.3)
12–24 h embryos	46.5	42.2	10,200	10,024 (98.3)	8,894 (87.2)	25,924	22,151 (85.4)	17,031 (65.7)
Larvae	40.2	13.0	11,089	9,990 (90.1)	7,613 (68.7)	23,184	16,887 (72.8)	14,386 (62.1)
Pupae	38.1	16.5	11,703	10,655 (91.1)	8,204 (70.1)	26,899	20,400 (75.8)	17,416 (64.7)
Female heads	15.7	37.7	9,542	9,365 (98.2)	7,779 (81.5)	24,058	18,870 (78.4)	13,135 (54.6)
Male heads	12.9	22.3	9,277	9,035 (97.4)	7,367 (79.4)	23,916	18,647 (78.0)	13,833 (57.8)
Female bodies (2 replicates)	27.1	30.0	9,513	9,381 (98.6)	7,472 (78.5)	22,455	18,748 (83.5)	13,849 (61.7)
Male bodies (2 replicates)	21.5	38.3	11,707	11,418 (97.5)	7,933 (67.8)	25,112	20,908 (83.3)	15,088 (60.1)
S2 cells (DMSO)	19.6	9.33	7,080	7,026 (99.2)	6,271 (88.6)	17,112	13,874 (81.1)	12,215 (71.4)

Uniquely mapped reads represent the total number of reads uniquely mapped to the mRNA regions of reference genome of *D*. *melanogaster*. For each sample, the most abundant isoform for each gene was used. Expressed genes were defined as genes with mRNA RPKM ≥ 1. Translated genes were detected with mRNA RPKM ≥ 1 and TE_CDS_ ≥ 0.1 or 0.5. The same criteria were applied to uORFs in expressed genes. The proportion of translated genes (uORFs) among all the expressed genes (uORFs) was shown in parentheses. For female bodies and male bodies, the reads of the two biological replicates were combined.

*mRNA-Seq and Ribo-Seq data of mature oocytes were obtained from GSE52799 [65].

Abbreviations: CDS, coding DNA sequence; DMSO, dimethyl sulfoxide; M, million; RPKM, reads per kilobase of transcript per million mapped reads; TE, translational efficiency; uORF, upstream open reading frame.

**Fig 1:**
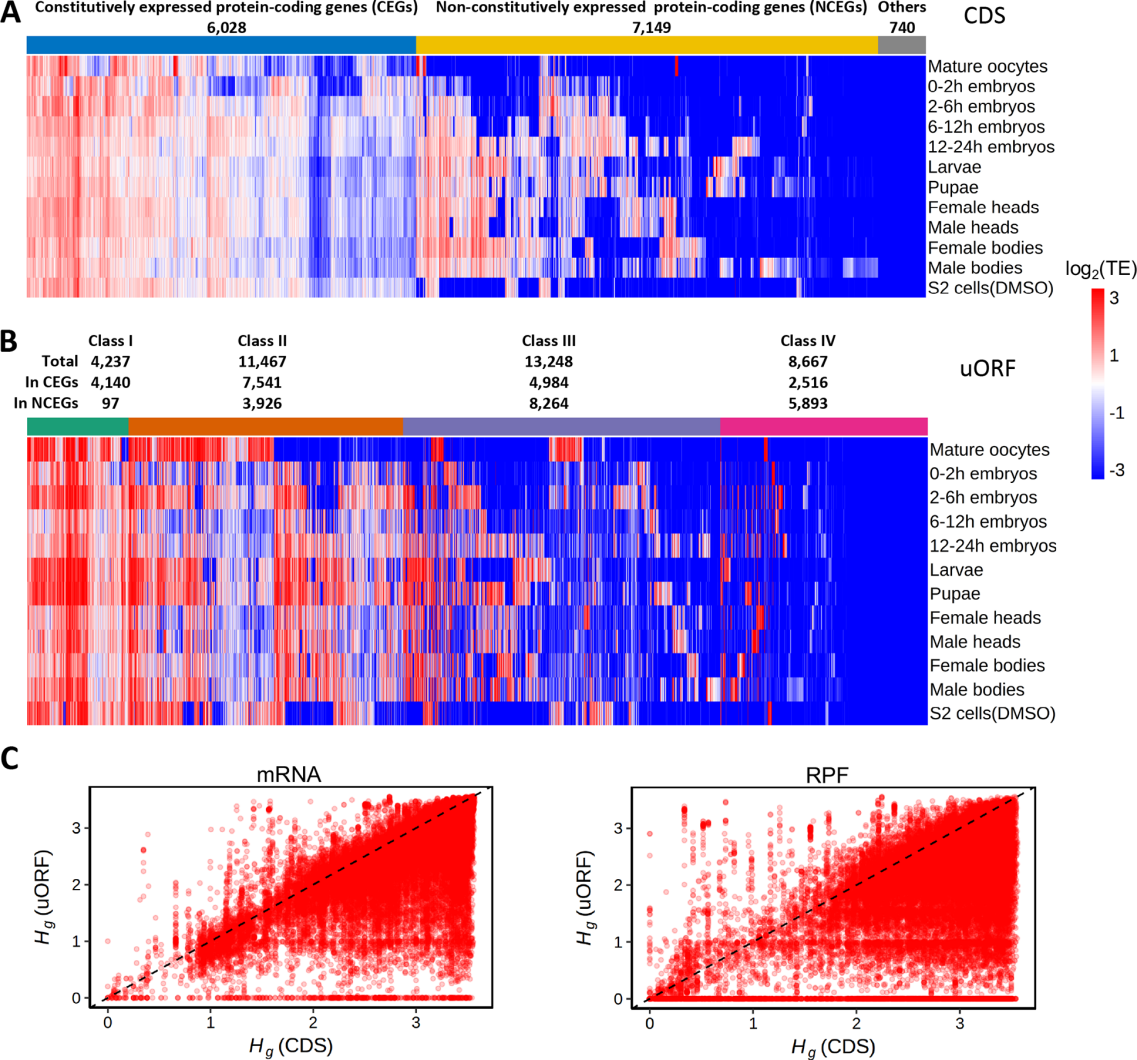
Genome-wide translational events on CDSs and uORFs during *Drosophila* development. (A) Heat map showing log_2_(TE) of CDSs for 13,917 protein-coding genes (column) in the 12 samples (rows). The numbers of CEGs and NCEGs are presented. TE values smaller than 0.1 or larger than 10 are displayed as 0.1 and 10, respectively. (B) Heat map showing log_2_(TE) of Class I–IV uORFs in the 12 samples. For each category, the total number of uORFs and the number of uORFs in CEGs or NCEGs are presented, respectively. TE values smaller than 0.1 or larger than 10 are displayed as 0.1 and 10, respectively. (C) The tissue specificity index *H*_*g*_ of uORFs (y-axis) against that for CDSs (x-axis) in mRNA-Seq (left) and Ribo-Seq (right) experiments. The exact values can be found in S1 Data. CDS, coding DNA sequence; CEG, constitutively expressed gene; DMSO, dimethyl sulfoxide; NCEG, nonconstitutively expressed gene; TE, translational efficiency; RPF, ribosome-protected mRNA fragment; uORF, upstream open reading frame

We mapped the RPFs (27–34 nt in length) to the reference genome, assigned each RPF read to its P-site (corresponding to the second binding site for a tRNA in the ribosome) as previously described [64], and calculated the density of RPFs (reads per kilobase of transcript per million mapped reads [RPKM]) for each feature (CDS or uORF, see Materials and methods). When the P-site of an RPF was located in multiple overlapping uORFs, it was assigned to all the overlapping uORFs, as previously described [10, 22]. For uORFs that were overlapping with CDSs, only the nonoverlapping regions of uORFs were used in calculating RPKM. We also mapped the mRNA-Seq reads and calculated the RPKM value for each feature (CDS or uORF) in each library (Materials and methods). We obtained 1,077 million reads in total (see Table 1 for sequencing summary and S4 Table for mapping statistics). We performed two biological replicates for both female bodies and male bodies and observed high correlations between the replicates (Pearson’s *r*^2^ > 0.972 and *P* < 10^−307^ in both mRNA-Seq and Ribo-Seq results of CDSs, S2A Fig), suggesting the high reproducibility of our experimental procedures. Statistically significant but lower *r*^2^ (ranging from 0.636 to 0.760) between the biological replicates was observed for uORFs in both the mRNA-Seq and Ribo-Seq experiments, presumably due to the larger sampling variance caused by the shorter length of uORFs than CDSs (S2B Fig). Indeed, if we calculated the RPKM in the 5′ region of each CDS with the same length of a uORF, we obtained *r*^2^ values comparable to those for the uORFs (S2C Fig). Moreover, when we modeled the read count *K*_*ij*_ for a feature (CDS or uORF) *i* in biological replicate *j* (*j =* 1 or 2) as following a negative binomial distribution with mean *μ*_*ij*_ and dispersion *φ*_*i*_ (the variability between replicates) as previously described [66–70] (Materials and methods), we found the estimated *φ*_*i*_ values of uORFs are significantly higher than those of the CDSs for both mRNA-Seq and Ribo-Seq counts (S3 Fig). Consistent with Dunn and colleagues [64], the phase of mapped RPF reads along CDS was compromised, owing to the cutting bias of micrococcal nuclease (MNase) (S4 Fig and S5 Fig). As observed in mammals [13, 37] and yeasts [34], the ribosome occupancy around the cAUGs or AUG start codons of uORFs (uAUGs) was considerably higher than that of the flanking triplets in each of the 12 *D*. *melanogaster* samples (S6 Fig). For each sample, we followed previous procedures [34, 64, 71, 72] and calculated the translational efficiency (TE) for each feature (CDS or uORF) to measure its translational rate, by contrasting the RPKM of Ribo-Seq versus mRNA-Seq for that feature (in each sample, the median TE value for a feature is around 1, S7 Fig). With mRNA-Seq RPKM ≥ 1 as an arbitrary cutoff, we identified 6,028 protein-coding genes that were constitutively expressed (CEGs) in all 12 samples and another 7,149 protein-coding genes that were nonconstitutively expressed (NCEGs) but detected in at least 1 sample (Fig 1A). With TE_CDS_ ≥ 0.1 as an arbitrary cutoff, 94.5%–99.9% of the CEGs showed evidence of translation, and a slightly lower percentage (92.1%–97.3%) of the NCEGs were translated in a sample (see Table 1 and S8 Fig for details). We still found 62.1%–88.6% of the expressed genes are translated if we increased the TE_CDS_ cutoff to 0.5 (Table 1). Overall, these results suggest that our Ribo-Seq data detected the genome-wide translational activities of CDSs with high sensitivity.

Next, we examined whether our Ribo-Seq experiments efficiently captured the translational signals of uORFs. We focused on the 35,735 uORFs that were annotated in the modENCODE mRNA-Seq and CAGE-Seq data and also expressed in at least 1 of the 12 samples we examined (mRNA-Seq RPKM ≥ 1). We found 32,224 (90.2%) and 28,952 (81.0%) of these uORFs showed evidence of translation at TE_uORF_ ≥ 0.1 and ≥ 0.5 in at least 1 of the 12 samples, respectively (Fig 1B). In an individual sample, 72.8%–87.0% and 54.3%–75.4% were translated with TE_uORF_ ≥ 0.1 and ≥ 0.5, respectively (Table 1). Overall, the number of ribosome-associated uORFs in a sample varied from 9,939 to 17,416 (Table 1), with pupae having the highest number of translated uORFs and mature oocytes having the lowest number of uORFs, evidenced by ribosome occupancy. The gene enrichment analysis suggests that genes lacking ribosome-associated uORFs were enriched in the pathways such as “cuticle structure,” “energy metabolism,” or “chromatin organization” (S9A Fig and S5 Table). The genes with ribosome-associated uORFs were significantly enriched for “regulation of transcription,” “protein kinase,” “axon guidance,” or receptor activities (S9B Fig and S5 Table), suggesting uORFs might play regulatory roles in these biological pathways.

Notably, 62.9%–65.5% of the expressed uORFs were overlapping with other features (uORFs or CDSs) in a sample (S6 Table). Although we only considered the nonoverlapping region of a uORF if it was overlapping with a CDS, it is possible that we might have overestimated the proportion of the translated uORFs by assigning a single RPF to multiple overlapping uORFs. To evaluate this possibility, in each sample, we separately considered the uORFs that overlapped with other features (overlapping) and those not overlapping with any other feature (nonoverlapping). At the cutoff of TE_uORF_ ≥ 0.5, the percentages of the nonoverlapping and overlapping uORFs that showed evidence of translation in a sample were comparable (49.2%–75.2% versus 55.5%–75.5% for the former versus the latter, *P* = 0.24, Student paired *t* test; S6 Table). Moreover, even if we assigned an RPF to the longest uORF in case it was matched to multiple overlapping uORFs, the proportion of the translated uORFs in a sample was only modestly affected (S7 Table). Thus, our observation that most uORFs were translated might not be affected by the overlapping between uORFs.

### uORFs overall have lower TE than the downstream CDSs

Previous studies suggest that uAUGs are generally located in disfavored Kozak sequence contexts compared to cAUGs [16, 18, 22]. To examine whether TE is different between uORFs and CDSs, for a uORF *i* and its downstream CDS *i*, we denoted *β*_*i*_ = TE_uORF,*i*_/TE_CDS,*i*_, and tested whether log_2_(*β*_*i*_) = log_2_(TE_uORF,*i*_)–log_2_(TE_CDS,*i*_) is significantly different from 0 in a sample (S10 Fig; Materials and methods). Note that for a feature (uORF or CDS), we assume its log_2_(TE) follows a normal distribution. We first estimated the standard error (SE) of log_2_(TE) based on the biological replicates of female and male bodies, grouped them according to increasing normalized mRNA counts and log_2_(TE), and then fitted the SE values against the mRNA counts and log_2_(TE) to obtain a smooth surface. For a feature in the samples without biological replicates, we estimated the SE of log_2_(TE) by subjecting the observed mRNA count and log_2_(TE) to the fitted surface obtained with the biological replicates of female and male bodies (Materials and methods). When we focused on the uORFs and CDSs that were well transcribed (RPKM ≥ 1 and normalized read counts ≥ 30 in mRNA-Seq), we found 27.3%–66.3% of the uORFs are significantly different from CDSs in TE (false discovery rate [FDR] < 0.05, Table 2 and S11 Fig). Although 7.2%–49.2% of the uORFs had higher TE than CDSs, significantly higher proportions (9.5%–54.3%) of uORFs have lower TE than CDSs (*P* = 0.031, Wilcoxon signed-rank test), suggesting uORFs are overall translated at lower efficiency than the downstream CDSs. Moreover, for a certain uORF, the *β* value (TE_uORF_/TE_CDS_) often varies across samples (S11 Fig), suggesting a uORF might play a regulatory role in a stage- or tissue-specific manner.

**Table 2:** Summary of well-expressed uORFs that have significantly different TE relative to the downstream CDSs or zero RPF coverage in each sample.

Sample	Total	*β* < 1 (%)	*β* > 1 (%)	RPF = 0 (%)	*P*_*m*_(*R*_*0*_)		
					*H*_*0*_(*c*)	*H*_*0*_(*u*)	*H*_*0*_(*0*.*1*)
Mature oocytes	1,867	178 (9.5)	919 (49.2)	71 (3.8)	20	24	0
0–2 h embryos	10,517	4,151 (39.5)	1,556 (14.8)	339 (3.2)	147	58	2
2–6 h embryos	8,760	2,435 (27.8)	2,118 (24.2)	195 (2.2)	145	1	1
6–12 h embryos	10,891	5,917 (54.3)	1,300 (11.9)	410 (3.8)	314	42	0
12–24 h embryos	11,817	4,982 (42.2)	1,321 (11.2)	351 (3.0)	250	36	1
Larvae	5,018	1,368 (27.3)	1,163 (23.2)	341 (6.8)	177	61	6
Pupae	6,577	1,712 (26.0)	1,383 (21.0)	474 (7.2)	170	63	1
Female heads	7,138	2,706 (37.9)	1,056 (14.8)	184 (2.6)	99	0	2
Male heads	5,720	1,923 (33.6)	899 (15.7)	175 (3.1)	68	2	5
Female bodies	9,815	2,679 (27.3)	897 (9.1)	230 (2.3)	135	13	0
Male bodies	7,590	1,528 (20.1)	545 (7.2)	86 (1.1)	33	2	0
S2 cells (DMSO)	5,193	1,034 (19.9)	1,278 (24.6)	81 (1.6)	61	0	2

Only well-transcribed uORFs (RPKM ≥ 1 and normalized read counts ≥ 30 in mRNA-Seq) were considered in each sample. *β* = TE_uORF_/TE_CDS_. The percentage of uORFs with *β* > 1, *β* <1, or RPF counts = 0 among all well-transcribed uORFs in a sample was displayed in parentheses. *P*_*m*_(*R*_*0*_) is the probability of observing 0 RPFs at a uORF under different hypotheses: *H*_*0*_(*c*), that the uORF has the same TE as the downstream CDS; *H*_*0*_(*u*), that TE_uORF_ is equal to the average TE (*u*) of this uORF in at least two other samples in which it has ≥ 30 normalized mRNA reads and ≥ 3 normalized RPF reads; *H*_*0*_(*0*.*1*), that the uORF has a fixed TE of 0.1. The numbers of significant uORFs were determined with an FDR of 0.05.

Abbreviations: CDS, coding DNA sequence; DMSO, dimethyl sulfoxide; FDR, false discovery rate; RPF, ribosome-protected mRNA fragment; RPKM, reads per kilobase of transcript per million mapped reads; TE, translational efficiency; uORF, upstream open reading frame.

### Many uORFs are transcribed or translated in a stage- or tissue-specific manner

The transcriptome of *D*. *melanogaster* is highly dynamic during development, with prevalent stage-, tissue-, or sex-specific gene expression, alternative transcription initiation, or splicing [61, 63]. Consistently, we found many CDSs are not constitutively transcribed or translated in all 12 samples (Fig 1A). To explore in depth the transcriptional dynamics of uORFs across samples, we examined the uORFs in the CEGs and NCEGs separately. At the mRNA RPKM ≥ 1, 13,230–16,005 uORFs in the CEGs were expressed in a sample, and 9,162 of these uORFs are constitutively expressed in all 12 samples. Due to the developmental stage- or tissue-specific expression of the NCEGs, the numbers of uORFs expressed in the NCEGs varied wildly across samples (ranging from 1,275 to 11,198), suggesting the widespread transcriptional dynamics of uORFs across samples. At TE_uORF_ ≥ 0.5, 56.3%–78.3% of the expressed uORFs in CEGs and 42.0%–66.2% of the uORFs in NCEGs were evidenced with ribosomal P-site occupancy (S8 Fig), suggesting some uORFs might be selectively translated during *Drosophila* development, although the patterns might be different between uORFs in CEGs and NCEGs.

To quantitatively measure to what extent a feature (uORF or CDS) is expressed in a stage- or tissue-specific manner, we calculated the tissue specificity index *H*_*g*_ [73]. An *H*_*g*_ value closer to 0 indicates more restricted expression, while an *H*_*g*_ value closer to log_2_(*N*) means broader expression, where *N* is the number of samples. We found uORFs have significantly smaller *H*_*g*_ values than the corresponding downstream CDSs in the mRNA-Seq data (*P* < 10^−307^, Wilcoxon rank-sum test [WRST]; Fig 1C), and this pattern still held when we controlled for the difference in length between uORFs and CDSs (*P* < 10^−307^, WRST; S12 Fig). These results further support that many uORFs are transcribed in a stage- or tissue-specific manner. Our observation is also consistent with previous in silico studies that uORFs are significantly enriched in the alternatively rather than the constitutively expressed regions in 5′ UTRs of mammals [47]. Notably, the uORFs also had significantly smaller *H*_*g*_ values than the corresponding downstream CDSs in the Ribo-Seq data (*P* < 10^−307^, WRST; Fig 1C and S12 Fig), suggesting the transcription or translation of uORFs is more restricted than that of CDSs.

To examine in depth whether uORFs are selectively translated, we only focused on the uORFs in the genes that had the same dominant transcripts constitutively expressed in all the 12 samples. Briefly, we employed kallisto [74] to quantify the abundance of each mRNA transcript and detected the major (most abundant) transcript in each mRNA-Seq library (Materials and methods). The major isoforms that were 2-fold dominant [75] (expressed at least twice as much as the second most abundant isoform) in each of our mRNA-Seq libraries (ranging from 5,581 to 9,324) were well supported (85.5%–92.6%) by the mRNA-Seq data in the matched samples of the modENCODE project (S8 Table). By this way, the possible bias caused by the minor transcripts and selective transcription of the uORFs are well controlled. Among the 1,515 uORFs that were constantly expressed in these dominant isoforms across all 12 samples (mRNA RPKM ≥ 1 in each library), only 443 (29.2%) and 1,081 (71.4%) of them showed evidence of translation in all the samples under TE_uORF_ ≥ 0.5 and TE_uORF_ > 0, respectively. These results are in line with the notion that uORFs might be selectively translated during *Drosophila* development. Nevertheless, the translation of some uORFs might not be detected in our Ribo-Seq because of sampling errors, since uORFs are overall short and poorly translated, especially for the lowly expressed ones. Indeed, for the well-transcribed uORFs in each sample (RPKM ≥ 1 and normalized read counts ≥ 30 in mRNA-seq), only 1.1%–7.2% of them did not show any signal of translation (i.e., 0 RPFs, Table 2). To further evaluate the effect of sampling bias, we calculated *P*_*m*_(*R*_*0*_), the probability of observing 0 RPF reads for a uORF with the observed mRNA read count in a sample *m* under the null hypothesis (*H*_*0*_(*c*)) that the TE of that uORF is the same as that of the downstream CDS (S13 Fig). Note that our calculation of *P*_*m*_(*R*_*0*_) takes into account the possible sampling errors in mRNA and RPF read counts of both uORFs and CDSs (Materials and methods). At the FDR of 0.05, we found roughly 28.2%–76.6% of the well-transcribed uORFs that have 0 RPF reads detected might be truly not translated under *H*_*0*_(*c*) (Table 2). Moreover, for a uORF that had 0 RPFs detected in a sample *m* but showed evidence of translation in at least two other samples (the average TE was *u*), we calculated *P*_*m*_(*R*_*0*_) under the null hypothesis that the TE for that uORF in sample *m* was *u* (*H*_*0*_(*u*)). Also, we calculated *P*_*m*_(*R*_*0*_) by assuming the expected TE for that uORF was 0.1 (*H*_*0*_(0.1)). Not surprisingly, we found lower proportions of the well-transcribed uORFs that did not show evidence of translation might be truly untranslated under *H*_*0*_(*u*) or *H*_*0*_(0.1), because of overall TE_CDS_ > TE_uORF_ > 0.1 (Table 2). These results reinforce the thesis that some uORFs are not translated although they are well transcribed.

In case we detected 0 RPF reads on a uORF in multiple samples in which it is well transcribed, we aggregated the *P* values using Fisher’s method [76] or calculated the *P* value after pooling the mRNA or RPF reads across those samples (Materials and methods). In total, there are 2,077 well-transcribed uORFs that had 0 RPFs in at least 1 sample. At the FDR of 0.05, 1,152 (55.5%, Fisher’s method) or 1,190 (57.3%, pooling method) of them might not be translated under *H*_*0*_(*c*) (S9 Table). Note that here we only focused on the well-transcribed uORFs and did not consider the lowly transcribed ones, because of limited statistical power. Overall, our deep sequencing results suggest that a large number of uORFs were transcribed and translated during *Drosophila* development, and many of these uORFs were not constitutively transcribed. Interestingly, even if the uORFs are transcribed, some of them might be selectively translated. Based on the ribosome occupancy patterns, we classified the 28,952 uORFs that showed strong evidence of translation (TE_uORF_ ≥ 0.5) into 3 classes (Fig 1B). Class I contained 4,237 uORFs that were associated with RPFs in ≥ 11 out of 12 samples, Class II was comprised of 11,467 uORFs translated in 5–10 samples, and Class III consisted of 13,248 uORFs evidenced with ribosome occupancy in 1–4 samples. Class IV was made up of 8,667 uORFs, including 6,783 uORFs that were expressed with mRNA-Seq RPKM ≥ 1 in at least one of our samples but did not show evidence of translation at the cutoff of TE_uORF_ ≥ 0.5, and 1,884 uORFs that were only expressed in the modENCODE mRNA-Seq data. Not surprisingly, we found the fraction of the well-transcribed uORFs that were not detected in the Ribo-Seq data in at least 1 sample increased in the order of the Class I, II, III, and IV (S9 Table). The difference in translational breadth (defined as the number of samples in which a uORF is translated) among the four classes of uORFs might cause them to show differences in genomic features and evolutionary patterns.

### Validating the ribosome-occupied uORFs

To provide further evidence that uORF-associated RPFs were generated as a result of those uORFs having undergone translation, we employed two different approaches: (1) contrasting the coverage of RPFs in the bona fide and hypothetical uORFs and (2) profiling translation initiation events in harringtonine-treated S2 cells.

**Fig 2:**
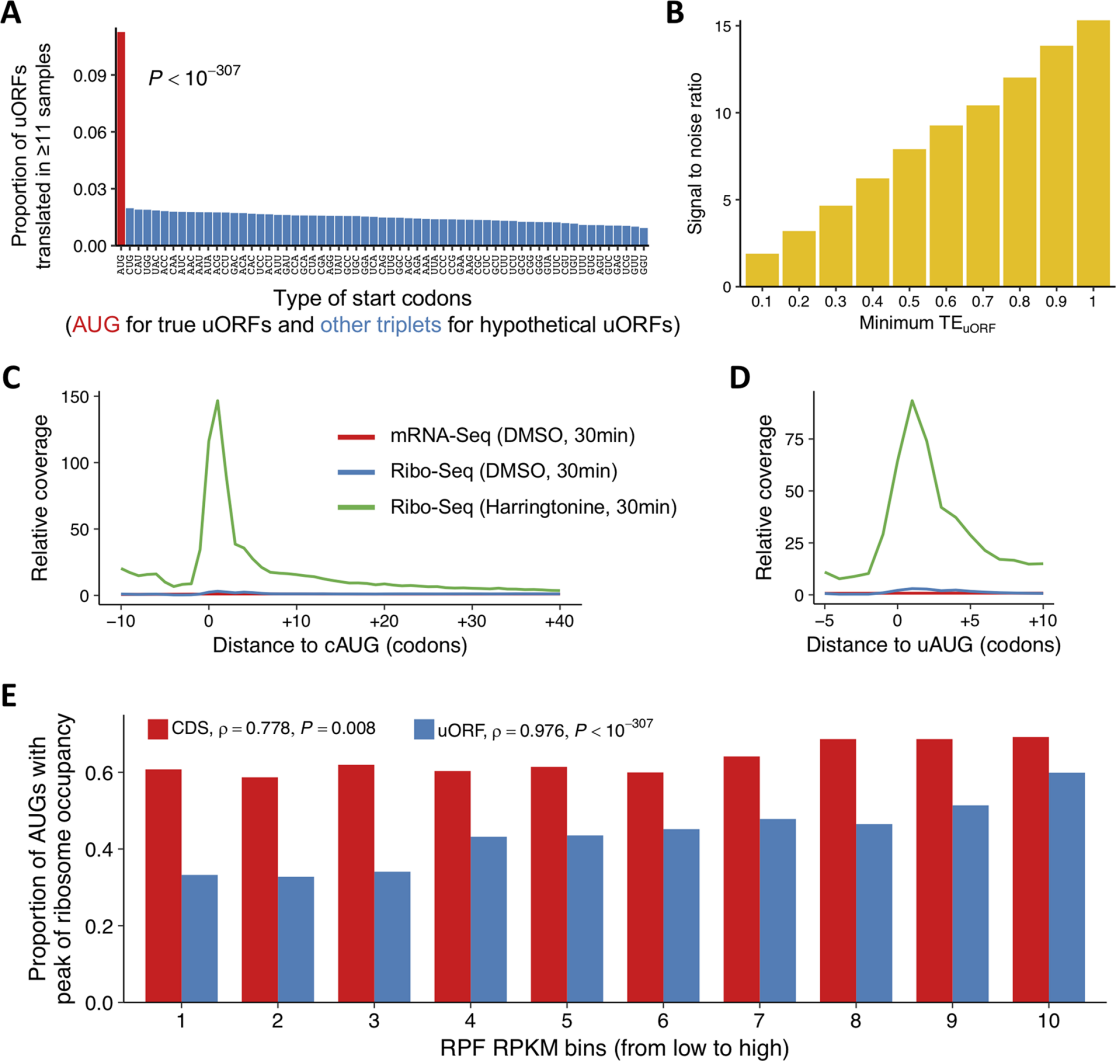
Validating the translation initiation of uORFs. (A) The proportions of canonical uORFs (beginning with AUG, red) and the other 60 kinds of hypothetical uORFs (each beginning with a distinct non-stop-codon triplet, blue) that are bound with ribosomes in at least 11 out of 12 samples (only uORFs with mRNA RPKM ≥ 1 and TE ≥ 0.5 were considered). (B) Signal-to-noise ratio at different cutoffs of minimum TE_uORF_. For each cutoff of minimum TE_uORF_, the proportion of canonical uORFs that are bound with ribosomes at this cutoff is divided by those for the other 60 kinds of hypothetical uORFs. The average of those ratios is used as signal-to-noise ratio at this cutoff. (C) The relative mRNA and RPF coverage around cAUGs in S2 cells. For each codon downstream (or triplet upstream) the cAUG of a gene (x-axis), the sequencing coverage was calculated, and the relative coverage of that codon (triplet) was calculated by normalization with the median coverage of CDS of this gene. And then, the relative sequencing coverage was averaged across all the genes (y-axis). Red and blue lines represent mRNA-Seq and Ribo-Seq, respectively, of S2 cells treated with DMSO for 30 min. The green line represents Ribo-Seq of S2 cells treated with harringtonine for 30 min. (D) The relative mRNA and RPF coverage around uAUGs in S2 cells. The data normalization and line colors are the same as those in (C). (E) The proportions of CDSs and uORFs (y-axis) with start codons that showed peaks in Ribo-Seq of S2 cells treated with harringtonine for 30 min. CDSs and uORFs with RPF RPKM > 10 in Ribo-Seq of S2 cells are stratified into 10 bins of equal size based on increasing RPF RPKM (x-axis). Spearman’s rank correlation between the proportion of CDS (or uORFs) with peaks of ribosome occupancy, and the median RPKM of CDSs (or uORFs) in each bin, is displayed in the plot. The raw data for panels (A-E) can be found in S1 Data. cAUG, AUG start codon of CDS; CDS, coding DNA sequence; DMSO, dimethyl sulfoxide; RPF, ribosome-protected mRNA fragment; RPKM, reads per kilobase of transcript per million mapped reads; TE, translational efficiency; uAUG, AUG start codon of uORF; uORF, upstream open reading frame

First, we compared the proportion of the canonical uORFs (beginning with AUG) that were associated with RPFs to those of the hypothetical uORFs. Briefly, after masking the canonical uORFs in the 5′ UTRs, we assumed that each of the other 60 non-stop-codon triplets was the start codon of a hypothetical uORF that did not overlap with canonical uORFs. We then calculated the density of P-site coverage for that hypothetical uORF in each library, as performed for the canonical uORFs (Materials and methods). Compared to the canonical uORFs, significantly lower proportions of the hypothetical uORFs were associated with RPFs (TE_uORF_ ≥ 0.5) in at least 11 out of the 12 libraries (*P* < 10^−307^ in each of the 60 comparisons, Fisher’s exact tests; Fig 2A). The average signal-to-noise (i.e., canonical to hypothetical uORFs) ratio for this analysis was 7.91, which might be conservative given that some hypothetical uORFs might be genuine (near-cognate uORFs) [13, 34, 77, 78]. The difference was more striking when we increased the stringency of ribosomal occupancy (Fig 2B). For example, the average signal-to-noise ratio was 1.90 and 15.3 when we set TE_uORF_ ≥ 0.1 and ≥ 1.0, respectively (*P* < 5.6 × 10^−158^ in each of the 60 comparisons in both cases, Fig 2B). Thus, the uORF-associated RPFs detected in our Ribo-Seq data well reflect the translational events on the uORFs.

Second, we treated S2 cells with harringtonine and characterized the genome-wide translation initiation events of uORFs with Ribo-Seq (Materials and methods). It has been nicely demonstrated that harringtonine, which arrests ribosomes at the translation initiation sites [79], enhances ribosome occupancy around the genuine start codons in mammalian cells [13, 80, 81]. Our metagene density profiles revealed that, compared to dimethyl sulfoxide (DMSO) treatment, the ribosome occupancy around cAUGs (position 1) was considerably higher in the Ribo-Seq of harringtonine-treated S2 cells (30 min) (Fig 2C). Importantly, we also observed very similar patterns for uAUGs (Fig 2D), suggesting the translation initiation events of uORFs were efficiently captured in the harringtonine-treatment experiments.

RPF peaks around uAUG after harringtonine treatment usually indicate translation initiation of uORFs [37]. Here, we followed a previous study [37] and identified uAUGs or cAUGs with ribosome occupancy peaks by requiring that the ribosome occupancy at the +1 codon is larger than that at the +2 codon and greater than the summed occupancy of the −1 and −2 triplets [37]. We found that in the harringtonine-treated S2 cells, 63.4% of the cAUGs (only uORFs with Ribo-Seq RPKM > 10 in DMSO-treated S2 cells were considered) showed significant peaks of ribosome occupancy (Fig 2E). Moreover, CDSs with higher RPF densities in S2 cells tended to have higher proportions of cAUGs with ribosome occupancy peaks in the harringtonine-treated S2 cells (Spearman’s *rho* = 0.778, *P* = 0.008) when the CDSs were grouped into 10 equal-sized bins with increasing densities of RPFs. Using the same criteria, we found that 43.8% of uAUGs (Ribo-Seq RPKM > 10 in S2 cells) showed significant peaks compared to the flanking regions (Fig 2E). We also found that uORFs with higher RPF densities tended to have a higher proportion of uAUGs with ribosome occupancy peaks (Spearman’s *rho* = 0.976, *P* < 10^−307^), as observed for CDSs (Fig 2E). One should note that, overall, the proportions of uAUGs that show ribosome occupancy peaks were lower than those of cAUGs, presumably due to the disfavored sequence contexts around uAUGs compared to cAUGs [16, 18, 22].

Altogether, these results suggest that our Ribo-Seq experiments (without harringtonine pretreatment) satisfactorily captured the translation initiation events at uORFs. Our datasets can be used to detect uORF translation events in *Drosophila* with considerable sensitivity and with a high degree of accuracy.

### Genomic features influencing ribosome occupancy of uORFs

**Fig 3:**
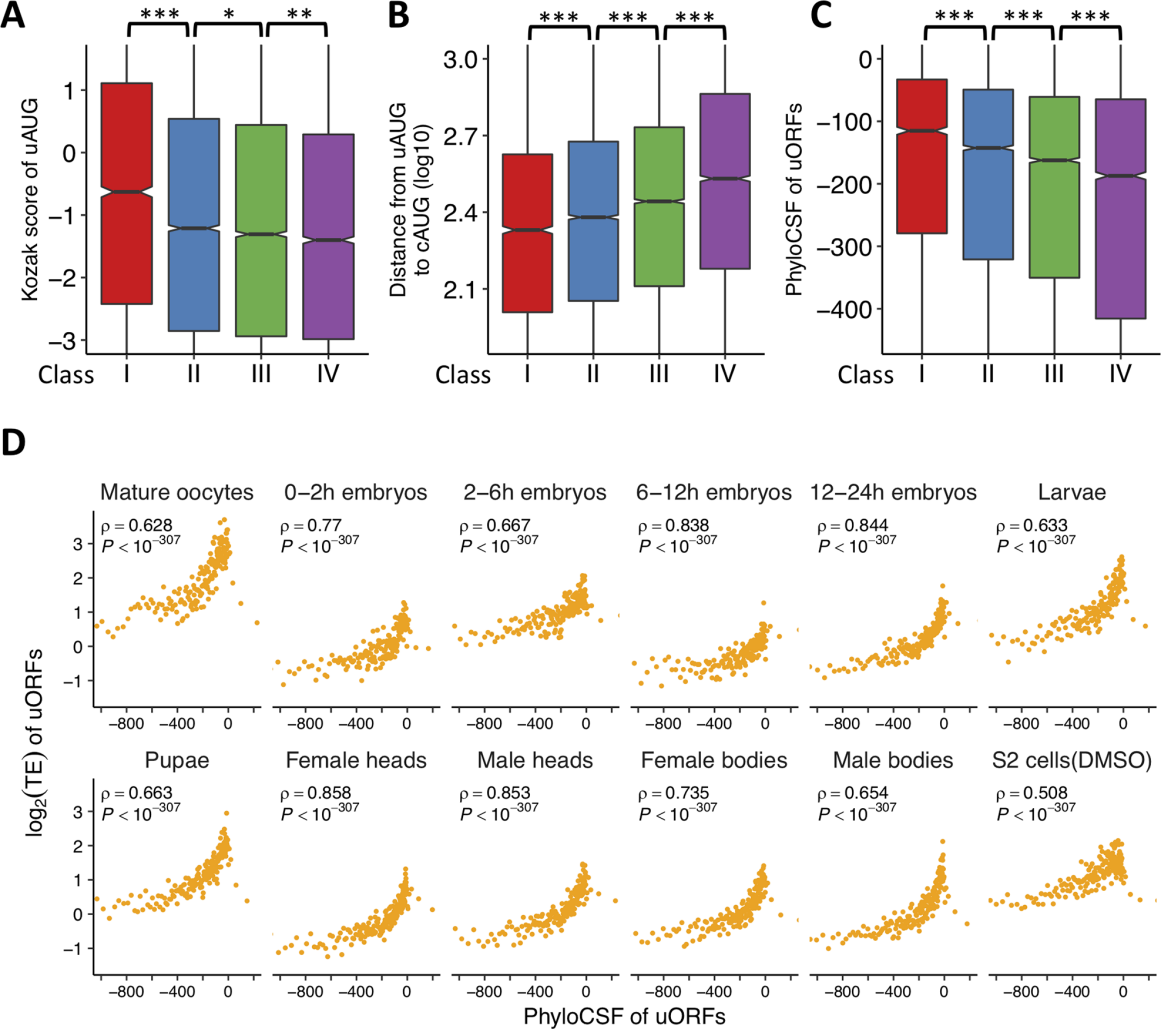
Features of uORFs with different translational breadths and efficiencies. (A-C) Box plots for Kozak score around uAUGs (A), the distance from a uAUG to the downstream cAUG (B), and the phyloCSF score (C) for each class of uORFs (*, *P* < 0.05; **, *P* < 0.01; ***, *P* < 0.001). The raw data can be found in S1 Data. (D) The relationship between phyloCSF (x-axis) and log_2_(TE) (y-axis) of ribosome-associated uORFs in each of the 12 samples. The ribosome-associated uORFs were ranked with increasing phyloCSF and divided into 200 bins of equal size. Median phyloCSF and median log_2_(TE) in each bin were displayed in the plot and used to calculate Spearman’s correlation coefficient. In each sample, only uORFs with mRNA RPKM ≥ 1 and TE ≥ 0.5 were used in the analysis. The raw data can be found in S2 Data. The raw data for panels (A-E) can be found in S1 Data. cAUG, AUG start codon of CDS; DMSO, dimethyl sulfoxide; RPKM, reads per kilobase of transcript per million mapped reads; TE, translational efficiency; uAUG, AUG start codon of uORF; uORF, upstream open reading frame

It is well established that genomic features affect the TE of uORFs as well as their repression efficiencies on CDSs [10, 13, 14, 16, 18, 20, 22, 34–37]. Nevertheless, it is unclear whether the genomic features show differences among uORFs with different translational breadth. In vertebrates and yeasts, uAUGs are generally located in disfavored Kozak sequence contexts compared to cAUGs [16, 18, 22]. To test whether this pattern holds true in *Drosophila*, we first retrieved the −6 to 1 nucleotides around each cAUG [4, 82] and derived a position probability matrix (PPM) for Kozak sequence contexts for all the CDSs (S10 Table). Then, we calculated the Kozak score for each uAUG or cAUG using this PPM (a higher Kozak score means a more preferred context for translation initiation). As shown in other species [16, 18, 22], in *Drosophila*, uAUGs also have significantly lower Kozak scores (i.e., they are located in disfavored contexts) compared to cAUGs (*P* < 10^−307^, WRST). Notably, in all the samples, higher Kozak scores tend to cause the higher TE of uORFs (S14A Fig). Interestingly, the Kozak score for each of the four classes of uORFs (Classes I to IV) monotonically decreased (*P* < 0.038, WRSTs; Fig 3A), suggesting that uORFs with higher translational breadth tend to have more preferred sequence contexts around their start codons.

AUG triplets are overall selected against within a 500 nt distance of the cAUG, while outside this distance, the selective pressure against AUG triplets is relatively weak [44]. Since the 5′ UTR regions adjacent to the cAUGs are generally less structured [83–85], it is possible that uORFs closer to the cAUGs might have a higher tendency of ribosomal occupancy and thereby experienced stronger selective pressures. Although purifying selection might have effectively removed the deleterious uORFs that are highly translated and closer to cAUGs, it is equally possible that some of the highly translated uORFs are beneficial and preserved in the genomes. To distinguish between these two possibilities, we examined the relationship between the distance from the uAUGs to the cAUGs and the tendency of ribosomal occupancy of the uORFs. The uAUGs of Classes I, II, and III were significantly closer to cAUGs (in a monotonically increasing manner) than those of Class IV (*P* < 0.001, WRSTs; Fig 3B), and uORFs whose uAUGs were closer to cAUG generally had significantly higher TE in all the samples (S14B Fig), suggesting that uORFs adjacent to cAUGs are more likely to be translated and functional in *D*. *melanogaster*.

Given the widespread translational signals of uORFs in *Drosophila*, we questioned whether the ribosome-associated uORFs have coding potential. A recent study, which identified approximately 2,700 uORFs that were translated in S2 cells by Poly-Ribo-Seq [86], suggests that the translated uORFs could not be distinguished from intergenic or random sequences in the phastCons scores [87] or amino acid compositions. Here, we pursued this issue with PhyloCSF scores, which measure the coding potentials based on sequence alignments [88]. A positive PhyloCSF score indicates the alignment is likely to encode a functional protein, whereas a negative score means otherwise. After subjecting the uORF sequence alignments across 23 insect species to the phyloCSF analysis (Materials and methods), we found the mean phyloCSF score monotonically decreased in the four classes (Class I to IV) of uORFs (*P* < 1.3 × 10^−12^, WRST; Fig 3C). Furthermore, in each sample, the uORFs with higher phyloCSF scores showed a stronger tendency to be associated with ribosomes (Fig 3D). Thus, uORFs with higher translational breadth or enhanced TE, in general, are more similar to the canonical coding regions in substitution patterns during evolution. However, these results do not necessarily suggest the translation events of such uORFs would produce functional peptides, because 93.5% of the translated uORFs had negative phyloCSF scores that were below the threshold of coding sequences [88]. This conclusion is also supported by the comparison of codon usages in the uORFs and CDSs of *D*. *melanogaster*: the relative synonymous codon usage (RSCU) [89] of uORFs was more similar to the random trinucleotide frequencies in the 5′ UTRs than to the RSCU of CDSs (S15 Fig). Altogether, these results suggest that the sequence composition of uORFs might be optimized to effectively associate with ribosomes. However, the outcome of this process is more likely to efficiently repress translation of the downstream CDSs rather than to directly encode functional peptides.

### Dual modes of purifying selection on the ribosome-associated uORFs

**Fig 4:**
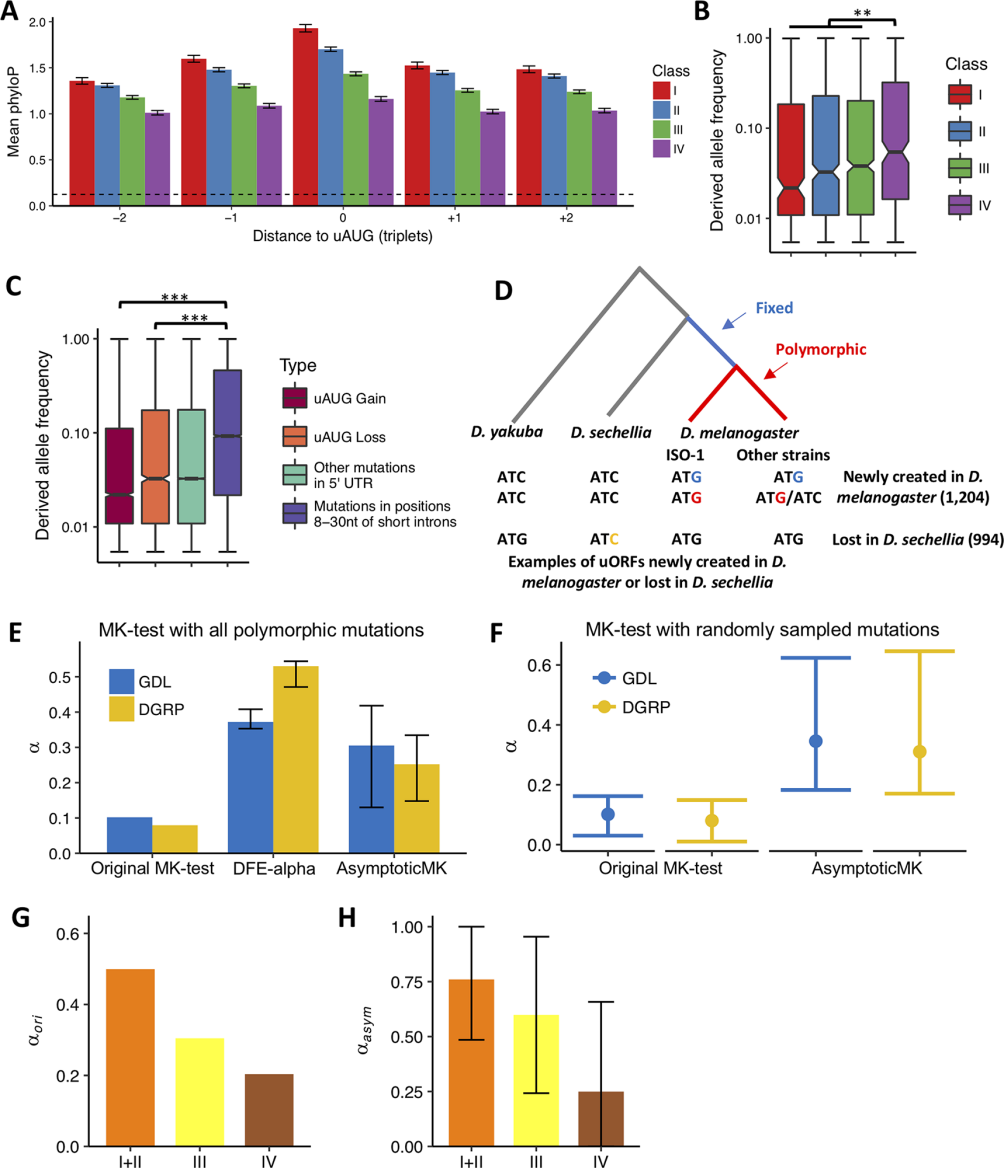
Evolutionary analysis of uAUGs. (A) The phyloP score (y-axis) of uAUGs and flanking triplets in uORFs in Classes I, II, III, and IV. The position of each triplet relative to uAUGs is shown in the x-axis. The mean and 95% CI (by bootstrapping) of the phyloP score are shown for each uORF class. The dashed line indicates the average phyloP score of positions 8–30 nt of short introns (neutral controls). The raw data can be found in S1 Data. (B) The derived allele frequency of uAUGs (from Classes I–IV) that are polymorphic in the GDL of *D*. *melanogaster*. Mutations from Class I, II, and III were combined to increase the statistical power (**, *P* < 0.01). The raw data can be found in S1 Data. (C) Frequencies of the derived mutations that cause the gain or loss of uORFs in the 5′ UTR, the remaining derived mutations in the 5′ UTR, and the derived mutations in positions 8–30 nt of short introns in the GDL of *D*. *melanogaster* (***, *P* < 0.001). The raw data can be found in S3 Data. (D) Examples of uAUGs (uORFs) that are newly created in *D*. *melanogaster* (fixed: blue; polymorphic: red) after divergence from *D*. *sechellia* or that are lost in *D*. *sechellia* (orange). The phylogenetic tree of *D*. *yakuba*, *D*. *sechellia*, and *D*. *melanogaster* (ISO-1 strain and other strains) is shown in the top panel, and the triplet sequences corresponding to each species or strains in the tree above are shown in the lower panel. For both the polymorphic and newly fixed uAUGs, only the ones present in the ISO-1 strain were considered in the analysis. (E) The *α* values of MK tests on the newly fixed mutations in uAUGs using AUGs in 8–30 nt of short introns as the neutral control for both GDL (blue) and DGRP (orange) data. Three different methods were used: the original MK test, DFE-alpha, and AsymptoticMK. The mutations in all the strains of *D*. *melanogaster* were used in the analysis. The error bars indicate 95% CI of *α*_*dfe*_ and *α*_*asym*_. The exact values can be found in S1 Data. (F) The *α* values of MK tests with randomly resampled mutations for both GDL (blue) and DGRP (orange) data. For both newly fixed and polymorphic AUGs in 5′ UTR and 8–30 nt of short introns, the same number of triplets were randomly sampled with replacement and used to perform the original MK and the AsymptoticMK tests. The median (points) and the 2.5% and 97.5% quantile (error bars) of *α*_*ori*_ and *α*_*asym*_ in 1,000 replicates were given. The raw data can be found in S1 Data. (G) The *α*_*ori*_ for mutations in uAUGs of Classes I and II (combined), III, and IV in GDL data. Only mutations present in the ISO-1 strain of *D*. *melanogaster* were used in the analysis. The raw data can be found in S11 Table. (H) The *α*_*asym*_ for mutations in uAUGs of Classes I and II (combined), III, and IV in GDL data. AUGs in 8–30 nt of short introns were used as the neutral control. Only mutations present in the ISO-1 strain of *D*. *melanogaster* were used. The error bars indicate 95% CI of *α*_*asym*_. The exact values can be found in S1 Data. The raw data for panels (A-E) can be found in S1 Data. DFE, distribution of fitness effects; DGRP, *Drosophila* Genetic Reference Panel; GDL, Global Diversity Lines; MK test, McDonald-Kreitman test; uAUG, AUG start codon of uORF; uORF, upstream open reading frame; UTR, untranslated region

Genes with different expression levels or different expression breadths show differences in evolutionary patterns [90–93]. Our phyloCSF analysis suggests that the nucleotide substitution patterns in the uORFs that had higher translational breadth are more similar to those in the canonical coding regions (Fig 3C and 3D). Since the uAUG is an essential defining feature of a uORF, here, we further asked whether uORFs with higher translational breadth are evolutionarily more conserved on the uAUGs. The phyloP scores [94], which measure sequence conservation levels based on multiple alignments, were significantly higher for uAUGs compared to their flanking (−3 to +3) triplets, suggesting uAUGs are evolutionarily more conserved. This pattern was consistently observed for the translated uORFs (*P* < 1.3 × 10^−53^ for each class, paired *t* tests; Fig 4A) and for the Class IV uORFs that showed little evidence of translation (*P* < 9.0 × 10^−14^, Fig 4A). Interestingly, for uAUGs and neighboring triplets, the phyloP score decreased with reduced translational breadth among the four classes of uORFs (Fig 4A). Moreover, in each of the 12 samples we surveyed, the uORFs with higher RPF densities tended to have higher phyloP scores (i.e., to be evolutionarily more conserved) at uAUGs (S16 Fig). Altogether, these results suggest that the uORFs with higher translational breadth across samples or highly translated in a sample have experienced stronger selective pressures to be preserved during *Drosophila* evolution.

Frequent gains and losses of uORFs have been observed in human populations, and some of these uORF-altering mutations are deleterious [15, 19, 21]. Here, we asked whether such a pattern exists in *D*. *melanogaster*. In the 84 strains of *D*. *melanogaster* sequenced in the Global Diversity Lines (GDL) project [95], we identified 4,263 and 2,498 SNPs that created or destroyed uAUGs, respectively (*D*. *sechellia* was used as an outgroup to polarize the mutations). Not surprisingly, the mutations that caused polymorphic uORFs associated with ribosomes (Class I, II, and III) had significantly lower derived allele frequency compared to Class IV uORFs (*P* = 0.006, WRST; Fig 4B), suggesting they are under stronger purifying selection. Compared to the mutations in positions 8–30 of short introns (≤65 nt), which evolve neutrally [96–99], both the AUG-creating and AUG-disrupting mutations had significantly lower derived allele frequencies (*P* < 4.4 × 10^−79^ in each comparison, WRST; Fig 4C). Similar results were obtained when we examined data from *Drosophila* Genetic Reference Panel (DGRP) [100, 101] of *D*. *melanogaster* (S17 Fig). In summary, our results suggest the segregating mutations in *D*. *melanogaster* that create new uORFs or destroy the existing ones are overall deleterious.

### Positive selection acts on newly fixed uORFs in *D*. *melanogaster*

Given that uORF-creating mutations are selected against at the population level, one question that remains to be addressed is what shaped the current distribution of uORFs in the genomes. Here, we tested two possible hypotheses about the origin and subsequent evolution of uORFs. The null hypothesis is that many newly emerged uORFs might be neutral or slightly deleterious but become fixed in the populations of *D*. *melanogaster* due to genetic drift. The alternative hypothesis is that although many mutations that create uAUGs are deleterious, the (slightly) beneficial ones would be driven to fixation very rapidly by positive Darwinian selection.

To distinguish between these two hypotheses, we first identified the newly emerged uORFs in the lineage of *D*. *melanogaster* after it diverged from *D*. *sechellia* about 5.4 million years ago [102], with *D*. *yakuba* as the outgroup (Fig 4D). Based on the genome sequence alignments of the 3 *Drosophila* species (indels and repetitive sequences were excluded), we found that 2,198 uAUGs detected in *D*. *melanogaster* are not present in *D*. *sechellia*: 994 (45.2%) of these differences were caused by nucleotide changes that disrupted the uAUGs in *D*. *sechellia*, and 1,204 (54.8%) were caused by the creation of uAUGs in *D*. *melanogaster*. These results suggest (1) that uORFs have undergone frequent gains and losses during evolution and (2) that prevalent new uORFs emerged in the lineage of *D*. *melanogaster* after its divergence from *D*. *sechellia*.

To test whether the newly emerged uORFs in *D*. *melanogaster* bear signatures of positive selection, we conducted a generalized McDonald-Kreitman (MK) test [103, 104] by contrasting the newly fixed uAUGs in the *D*. *melanogaster* lineage and the polymorphic uAUGs in the GDL of *D*. *melanogaster* (Fig 4D). As the neutral controls, we counted the newly fixed and polymorphic AUG triplets in positions 8–30 nt of short introns (≤65 nt) (Materials and methods). Since the possible demographic histories and the (slightly) deleterious mutations in the polymorphic data would cause a bias in estimating *α*, which is the fraction of nucleotide substitutions that are driven to fixation by positive selection [105–107], we estimated *α* with 3 alternative approaches that account for these effects. First, we removed the polymorphic AUG triplets that had low minor allele frequency (MAF < 0.05) and conducted the MK test as previously described [105, 108, 109]. With this original approach, we estimated that at least 7.9% (*α*_*ori*_) of the newly fixed uAUGs in *D*. *melanogaster* lineage were driven by positive Darwinian selection (Fig 4E).

Although we removed the low-frequency polymorphism (MAF < 0.05) in the above MK analysis (the “original” method), the estimation of *α* might still be biased, since some deleterious mutations might segregate at higher frequencies in the populations [110–113]. Thus, we also estimated *α* with the DFE-alpha method (*α*_*dfe*_), which analyzes the unfolded site frequency spectrum (SFS) and infers the distribution of fitness effects (DFE) for deleterious mutations and the prevalence and selective strength for advantageous substitutions [110, 111]. Also, the DFE-alpha method incorporates the demographic change that affects the fixation probability of selected alleles. The third method we used is AsymptoticMK, which first evaluates polymorphism levels for different mutation frequencies separately and then estimates *α* (*α*_*asym*_) by extrapolating a function fitted to the data [112, 114]. Since AsymptoticMK estimates *α*_*asym*_ at different derived allele frequencies, the bias that distorts SFS due to demographic history, background selection, or genetic draft will cancel out. Previous results suggest that both DFE-alpha and AsymptoticMK are more powerful in detecting positive selection than the original MK test [110, 112]. Indeed, we found both *α*_*dfe*_ and *α*_*asym*_ are larger than *α*_*ori*_ in both the DGRP and GDL dataset: 25.2%–53.0% of the newly fixed mutations creating uAUG in the *D*. *melanogaster* lineage were under positive selection (Fig 4E and S18 Fig). These results suggest higher fractions of newly fixed uAUGs are under positive selection after controlling for the effects of slightly deleterious mutations, demographic changes, and epistasis. One caveat in the above analyses is that the *α* values might be biased when pooling loci from different genomic regions that differ in the effective population size [106, 115, 116]. Nevertheless, empirical data analysis suggests summing data across loci in the MK test would not cause severe biases of *α* estimation [110]. Indeed, we still detected strong signals of positive selection in the newly fixed uAUGs when we randomly sampled the uAUGs in the 5′ UTRs and the ATG triplets in positions 8–30 of the short introns (with replacement) and calculated the *α* values (Fig 4F, see Materials and methods for details).

Next, we questioned whether the prevalence and strength of positive selection were different for the newly fixed uAUGs whose uORFs were different in translational breadth or TE. Since our mRNA-Seq and Ribo-Seq experiments were primarily carried out with the ISO-1 strain of *D*. *melanogaster*, which was sequenced to assemble the reference genome of *D*. *melanogaster* [117], in the MK tests, we would only consider the mutations that were present in the ISO-1 strain. Since DFE-alpha relies on the full spectrum of site frequency [110, 111], and the results will be distorted if we only consider the mutations present in the ISO-1 strain, here, we estimated *α* primarily based on the original MK test and AsymptoticMK. With the GDL polymorphism data, for the newly fixed uORFs in Classes I+II (combined), III, and IV, the *α*_*ori*_ analysis suggests that 49.9%, 30.5%, and 20.4% of them, respectively, were under positive selection (Fig 4G, S11 Table). As expected, the AsymptoticMK analysis revealed an even higher *α* value (*α*_*asym*_ = 68.3%) for all the translated uORFs (Classes I+II+III), and *α*_*asym*_ was higher than *α*_*ori*_ for each class of translated uORFs (Fig 4H). Importantly, both the original MK test and AsymptoticMK revealed the strength of positive selection decreased in the order of I+II, III, and IV. It should be noted that here, in both methods, we only considered the mutations present in the ISO-1 strain. To evaluate whether this approach would cause a biased estimation of *α*, we randomly sampled 1,000 genes and performed the MK tests on all the nonsynonymous and synonymous mutations in the populations of *D*. *melanogaster* versus those only present in the ISO-1 strain (the simulations were performed for 1,000 replicates, see Materials and methods). Compared to the MK tests based on the polymorphic data in all the strains, *α*_*ori*_ was overestimated to 1.38 (95% CI 1.21–1.86) and *α*_*asym*_ was overestimated to 1.03 (95% CI 0.91–1.26) folds of the original values when we only used the mutations present in the ISO-1 strain (S19 Fig). Although our analysis might exaggerate *α*_*ori*_, such effects should exist for each class of uORFs and might not distort the relative strength of positive selection on different classes of translated uORFs. The AsymptoticMK analysis, which was only modestly affected when we used only the mutations present in the ISO-1 strain, suggests that *α*_*asym*_ decreased in the order of Class I+II, III, and IV in both the GDL (Fig 4H) and the DGRP (S20 Fig) dataset. Moreover, among the newly fixed uORFs that were expressed in each developmental stage/tissue, those with higher TE_uORF_ in general had a higher *α* value than those with lower TE_uORF_ (S21 Fig). Overall, these results suggest that the newly fixed uORFs that show stronger signals of ribosome occupancy have experienced more substantial positive selection, presumably due to their more important regulatory roles across tissues or developmental stages. Notably, although many sites in the 3′ UTRs of *D*. *melanogaster* are under positive selection [104], we still detected prominent signals of positive selection in the newly fixed uORFs that were translated (*α*_*asym*_ = 0.343 for GDL and *α*_*asym*_ = 0.280 for DGRP data, S22 Fig) when we used the AUG-creating mutations in the 3′ UTRs as putatively neutral controls. Altogether, our results, to our knowledge, demonstrate for the first time that positive Darwinian selection is the driving force for the fixation of uORFs after their origins.

### Translational repression by ribosome-associated uORFs

**Fig 5:**
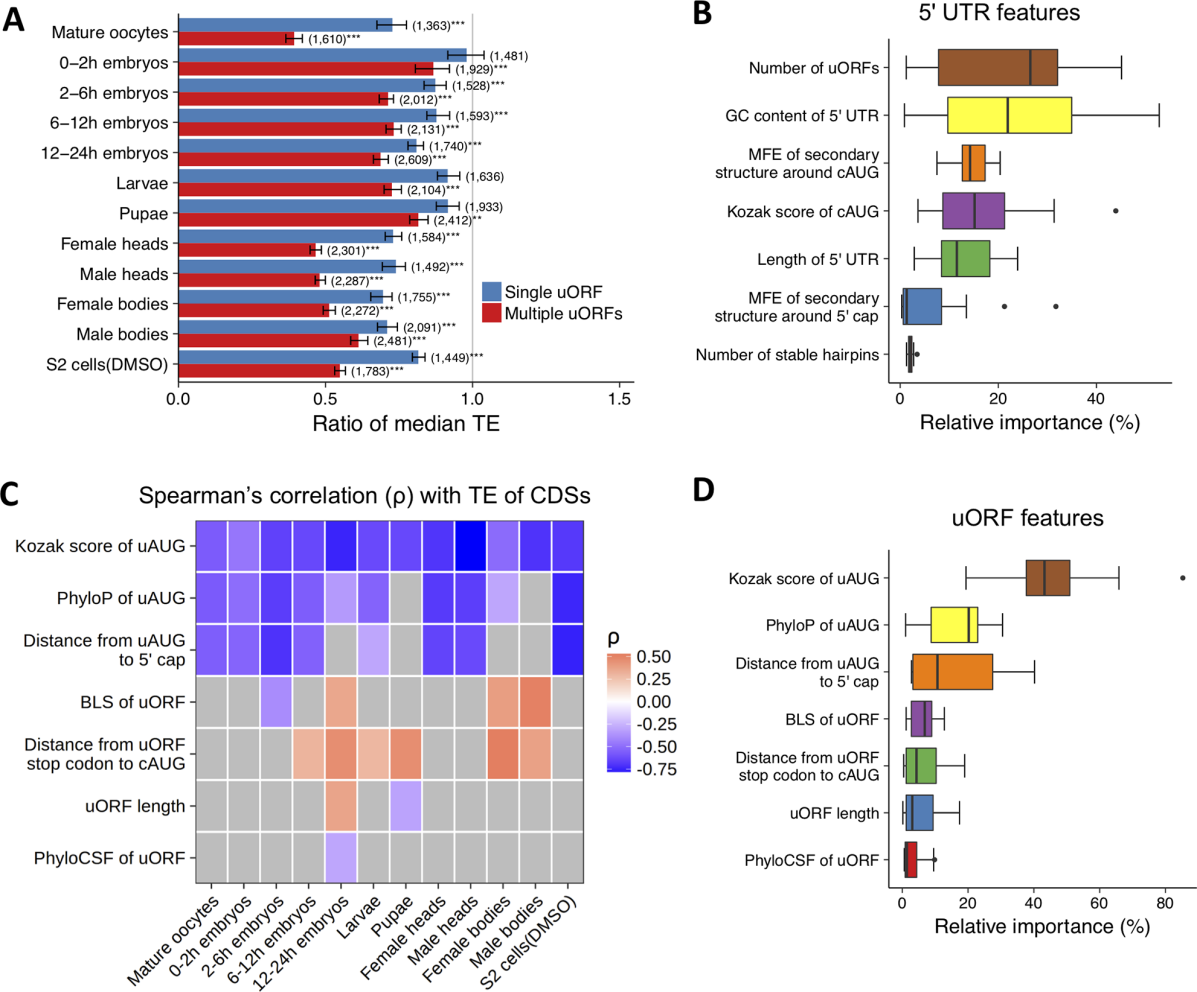
uORFs are prevalent translational repressors during *Drosophila* development. (A) The ratio of median TE for genes with single or multiple ribosome-associated uORFs, relative to the median TE for genes that do not have uORFs in each sample (only genes with mRNA RPKM ≥ 1 were included in analysis). WRSTs were performed to test the differences in each sample (**, *P* < 0.01; ***, *P* < 0.001). The 95% CI were obtained by bootstrapping. The number of genes in each category was given in parenthesis. The raw data can be found in S4 Data. (B) The relative importance (relative proportion of variance explained by each predictor, x-axis) of different features in 5′ UTRs on log_2_(TE) of CDSs. For each feature (y-axis), the 25%, 50%, and 75% quantiles of the relative importance across the 12 samples are manifested in the box plots. Only genes with at least 1 ribosome-associated uORF were included in the analysis. The raw data can be found in S12 Table. (C) Spearman’s correlation between the TE of CDSs and the features of uORFs (y-axis) across samples (x-axis). For each cell in the matrix, the genes were grouped into 50 bins of equal size based on the corresponding feature, and Spearman’s correlations were calculated using median log_2_(TE) and the median value of the feature in each bin. The raw data can be found in S1 Data. (D) The relative importance (x-axis) of various uORF features (y-axis) in the multiple linear regression on log_2_(TE) of CDSs across the 12 samples. The raw data can be found in S13 Table. BLS, branch length score; cAUG, AUG start codon of CDS; CDS, coding DNA sequence; DMSO, dimethyl sulfoxide; MFE, minimum free energy; RPF, ribosome-protected mRNA fragment; RPKM, reads per kilobase of transcript per million mapped reads; TE, translational efficiency; uAUG, AUG start codon of uORF; uORF, upstream open reading frame; UTR, untranslated region; WRST, Wilcoxon rank-sum test.

To detect whether the ribosome-associated uORFs affect the TE of the downstream CDSs, in each sample, we only focused on the major transcript for each gene and examined the relationship between the TE of the downstream CDS (TE_CDS_) and the number of uORFs that were translated in that transcript. As expected [10, 13, 14, 20, 22], we found genes containing ribosome-associated uORFs (TE_uORF_ > 0) have significantly lower TE_CDS_ compared to genes without ribosome-associated uORFs. Roughly speaking, TE_CDS_ was 8.38%–30.4% lower for genes containing 1 single translated uORF and 18.4%–60.7% lower for genes having multiple translated uORFs, except for the sample derived from 0–2 h embryos (Fig 5A; see S23 Fig for results with different cutoffs). Moreover, the number of translated uORFs showed significant negative correlation with TE_CDS_ in all the 12 samples (*rho* ranged from −0.360 to −0.027, *P* < 0.01, S24A Fig; and other TE_uORF_ cutoff yields similar results, S24B and S24C Fig). Our results thus suggest that uORFs inhibit translation of the downstream CDSs, most likely by competing for ribosomes. Notably, the anticorrelation between TE_CDS_ and the number of translated uORFs was weak in the 0–2 h embryos. Ribosome profiling data of 0–2 h embryos generated in other studies [64, 65] show a similar pattern (S25 Fig). Since translation is predominately controlled by poly(A)-tail length in early embryos of *Drosophila* [118], the repressive effects of uORFs on TE_CDS_ in the 0–2 h embryos might be overwhelmed by the activating effects of the poly(A)-tails, which are overall longer for uORF-containing genes (S26 Fig).

Besides uORFs, many *cis*-regulatory elements (CREs) in 5′ UTRs also influence TE_CDS_ [11]. In nearly all the samples, TE_CDS_ was significantly correlated with features in its 5′ UTR (S1 Text), such as the length of the 5′ UTR (negative correlation, S27 Fig), the GC content (negative, S28 Fig), the Kozak context of the cAUG (positive, S29 Fig), the minimum free energy (MFE) of the secondary structure around the cAUG (positive, S30 Fig), the MFE of the secondary structure around the 5′ cap (positive or negative, S31 Fig), and the number of stable hairpin structures in the 5′ UTR (negative, S32 Fig). Nevertheless, our analysis on the relative importance of the aforementioned features (S12 Table; S1 Text) suggests that the number of ribosome-associated uORFs significantly contributes to the reduced TE_CDS_ after controlling other factors (Fig 5B).

Previous studies in yeasts and animals suggest that the repressiveness of a uORF on its downstream CDS is specified by its sequence contexts, including the Kozak score for uAUG, uORF length, distance from uAUG to 5′ cap, and distance from uORF stop codon to cAUG [10, 14, 21, 22]. Our analysis revealed similar patterns in *Drosophila* (Fig 5C and S33–S36 Fig). Moreover, we also found the extent to which TE_CDS_ was repressed was more or less affected by the evolutionary features of uORFs: phyloP for conservation level of uAUGs (S37 Fig), phyloCSF for potentials to encode conserved peptides (S38 Fig), and branch length score (BLS) [119] for uORF sequence conservation levels across 23 *Drosophila* species (S39 Fig). It is possible that these genomic or evolutionary features influence the translational efficacy of uORFs, which further affects their repression efficiency on the translation of downstream CDSs. After multiple regression analysis between TE_CDS_ and these uORF features (S1 Text), we found that optimized Kozak contexts around uAUGs, high conservation level of uAUGs, and long distance between uAUG and 5′ cap are the most important features of uORFs that determine the repressiveness of uORFs on the downstream CDSs (S13 Table, Fig 5D).

In summary, our results suggest that in *Drosophila*, the ribosome-associated uORFs exert widespread regulatory effects in modulating TE of CDSs, and the key features of uORFs that specify their repressiveness might be conserved across *Drosophila*, yeasts, and vertebrates.

### Translational regulation by selective usages of uORFs during *Drosophila* development

**Fig 6:**
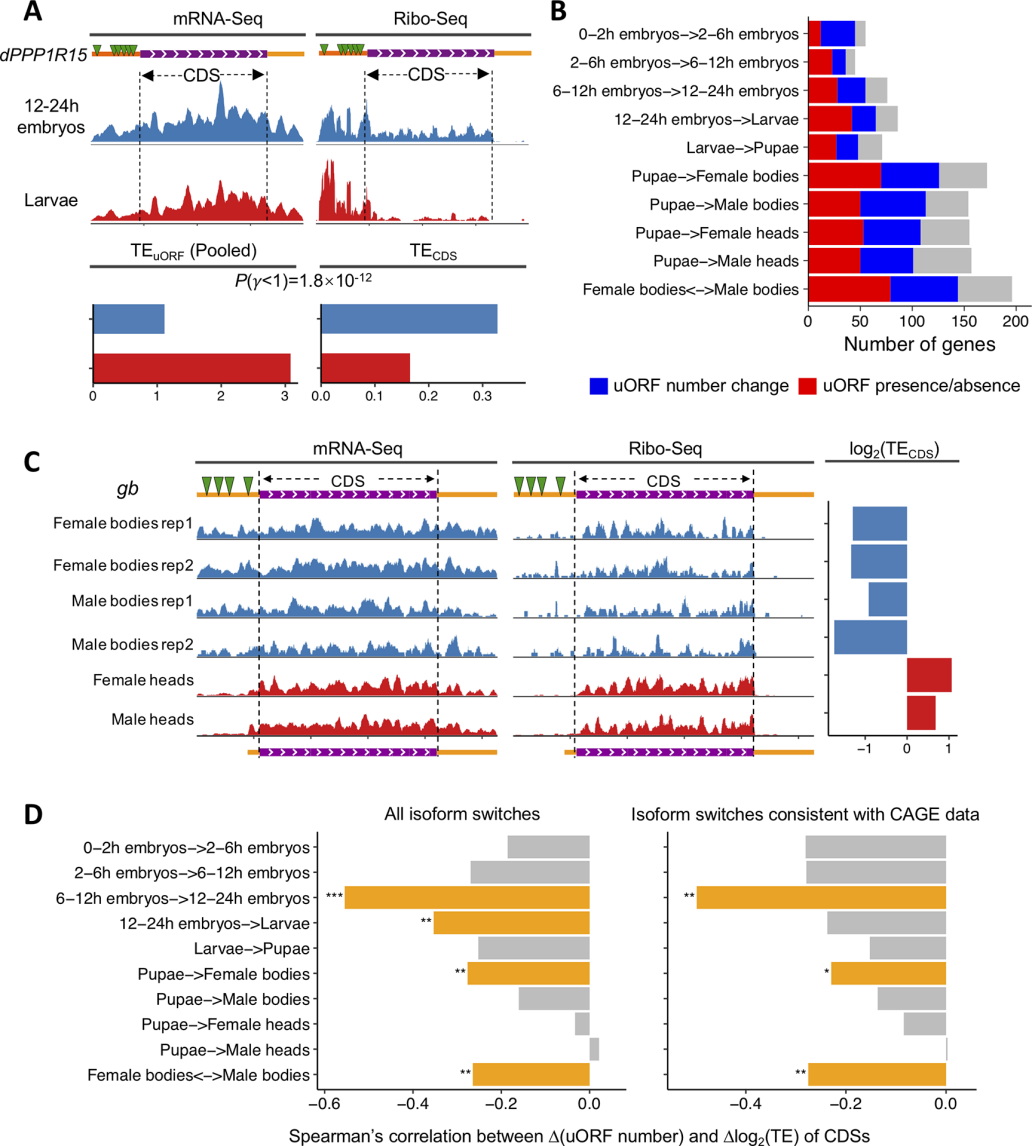
Translational regulation by selective usage of uORFs during *Drosophila* development. (A) The mRNA isoform, the profiles of the mRNA-Seq and Ribo-Seq data, pooled TE_uORF_ and TE_CDS_ of *dPPP1R15* in 12–24 h embryos and third-instar larvae of *D*. *melanogaster*. *dPPP1R15* has only one mRNA isoform, which contains 7 uORFs. The CDS and UTR region in the gene model are in purple and orange, respectively. The CDS region of *dPPP1R15* is also delineated with dashed lines. In larvae, the TE of uORFs (pooled) is remarkably increased, while that of CDS is decreased compared to 12–24 h embryos. Sequencing data are available from SRA under accession SRP067542. (B) The number of genes that switched the major (most abundant) isoforms while maintaining the same CDS between neighboring stages of *Drosophila* development or between different sexes in both our and modENCODE mRNA-Seq data. Only uORFs with mRNA RPKM ≥ 1 were considered. For each gene, the major transcripts should be 2-fold dominant in at least one of the paired samples. Gray indicates that the uORFs are the same despite the switch of major transcripts; blue indicates that the number of uORFs in the major transcripts differ between two stages or between different sexes; and red indicates that the change in major transcripts causes a switch from the presence to the complete absence of a transcript’s uORFs or vice versa. The exact values can be found in S1 Data. (C) The dominant isoforms, the profiles of the mRNA-Seq (left) and Ribo-Seq (middle) data, and log_2_(TE) (right) of *gb* in bodies or heads of *D*. *melanogaster* adults. The long isoform of *gb* (top) that contains four uORFs in its 5′ UTR (green triangles) is predominately expressed in bodies of female and male adults. The short uORF-free isoform is predominately expressed in female and male heads. The CDS and UTR region in the gene model are in purple and orange, respectively. The CDS region of *gb* is also delineated with dashed lines. TE of *gb* is higher in heads, in which the short isoform predominates, compared to that in bodies. The sequencing data r1 and r2 represent 2 biological replicates for both female and male adult bodies. Sequencing data are available from SRA under accession SRP067542. The exact values of log_2_(TE) of *gb* can be found in S1 Data. (D) The Spearman’s *rho* between changes (Δ) in log_2_(TE) of CDSs and the changes (Δ) in uORF numbers. The correlations for all isoform switching events that are supported by mRNA-Seq data of this study and modENCODE are shown in left panel, and those for isoform switching events that are further supported by CAGE data from modENCODE are shown in right panel (*, *P* < 0.05; **, *P* < 0.01; ***, *P* < 0.001). The raw data can be found in S1 Data. CAGE, cap analysis of gene expression; CDS, coding DNA sequence; FDR, false discovery rate; *gb*, *genderblind*; RPKM, reads per kilobase of transcript per million mapped reads; SRA, Sequence Read Archive; TE, translational efficiency; uORF, upstream open reading frame; UTR, untranslated region

Our analyses suggest that many uORFs might vary in TE across samples, even if they are constitutively expressed. Since uORFs impede translation of downstream CDSs by competing for ribosomes, we questioned whether the changes in TE of uORFs would impact TE of the downstream CDSs during *Drosophila* development. To this end, we focused on the genes that have the same dominant isoforms between two neighboring developmental stages as supported by the CAGE and mRNA-Seq data and examined the relationship between changes in TE of well-transcribed uORFs and their downstream CDSs. Notably, the changes in TE_uORF_ were significantly positively correlated with changes in TE_CDS_ in all the pairs of samples we examined (S40 Fig), presumably due to the genewise *trans*-regulatory effects that were exerted on the translation of both the uORFs and their downstream CDSs. Nevertheless, the magnitude of changes in TE_CDS_ was generally less than that in TE_uORF_ if TE_uORF_ is increased and vice versa (S40 Fig), suggesting the magnitude of changes in TE_CDS_ is inversely affected by changes in TE_uORF_ during development. To control for the stochastic sampling effect in this analysis, we first identified uORFs that showed statistically significant changes in TE between the two samples. Briefly, for each uORF that was expressed in both sample 1 and 2, we tested whether log_2_(*β*_*u*_) = log_2_(TE_uORF,2_)–log_2_(TE_uORF,1_) is significantly different from 0 (Materials and methods). We found 9.3%–55.8% of the well-transcribed uORFs we examined showed significant differences in TE (*β*_*u*_ ≠ 1) between neighboring samples (Table 3). To quantitatively examine whether the changes in TE_uORF_ between sample 1 and 2 would inversely impact the magnitude of changes in TE_CDS_ between these two samples, we defined *γ* = (TE_CDS,2_ / TE_CDS,1_) / (TE_uORF,2_ / TE_uORF,1_) and tested whether log_2_(*γ*) is significantly different from 0 (see S41 Fig for the scheme, Materials and methods). Among the well-transcribed uORFs that show *β*_*u*_ ≠ 1 between neighboring samples, 37.4%–79.2% of them had downstream CDSs that showed *γ* ≠ 1 between the matched samples (Table 3). Strikingly, in each pair of samples, uORFs with log_2_(*β*_*u*_) > 0 were usually accompanied with log_2_(*γ*) < 0 and vice versa (*P* < 0.01 in each pair of samples, χ^2^ test). These results further support the notion that the magnitude of changes in TE_CDS_ is inversely affected by changes in TE_uORF_ during development. For example, *dPPP1R15* is a eukaryotic initiation factor 2 alpha (eIF2α) phosphatase that is important for *Drosophila* development [120]. *dPPP1R15* has only one transcript, and the translation of its CDS is regulated by its uORFs [120]. Compared to in 12–24 h embryos, the TE of uORFs is considerably increased, and the TE of the CDS is remarkably reduced in larvae (Fig 6A). Altogether, our results suggest that changes in TE of uORFs might be important to modulate the translation of CDSs during *Drosophila* development.

**Table 3:** Summary of uORFs whose TE changed disproportionally relative to the downstream CDSs between two neighboring developmental stages.

Sample 1	Sample 2	Total uORFs	*β*_*u*_ ≠1	*β*_*u*_ > 1			*β*_*u*_ < 1			*χ*^*2*^ test *P* value
				Total (%*)	γ > 1 (%**)	*γ* < 1 (%**)	Total (%*)	*γ* > 1 (%**)	*γ* < 1 (%**)	
0–2 h embryos	2–6 h embryos	2,679	978 (36.5)	737 (75.4)	0 (0)	331 (44.9)	241 (24.6)	33 (13.7)	2 (0.8)	7.5 × 10^−80^
2–6 h embryos	6–12 h embryos	2,891	1,612 (55.8)	49 (3.0)	0 (0)	20 (40.8)	1,563 (97.0)	1,129 (72.2)	0 (0)	7.4 × 10^−252^
6–12 h embryos	12–24 h embryos	2,669	247 (9.3)	218 (88.3)	0 (0)	97 (44.5)	29 (11.7)	3 (10.3)	0 (0)	1.5 × 10^−23^
12–24 h embryos	Larvae	989	318 (32.2)	300 (94.3)	0 (0)	244 (81.3)	18 (5.7)	8 (44.4)	0 (0)	9.5 × 10^−57^
Larvae	Pupae	828	90 (10.9)	44 (48.9)	0 (0)	10 (22.7)	46 (51.1)	25 (54.3)	0 (0)	3.3 × 10^−09^
Pupae	Female heads	629	217 (34.5)	8 (3.7)	0 (0)	1 (12.5)	209 (96.3)	140 (67.0)	0 (0)	1.6 × 10^−32^
Pupae	Male heads	522	134 (25.7)	10 (7.5)	1 (10.0)	3 (30.0)	124 (92.5)	78 (62.9)	0 (0)	1.0 × 10^−18^

Only uORFs well transcribed and located in 2-fold dominant isoforms that are constitutively expressed and supported by modENCODE CAGE and mRNA-Seq data in both samples are considered in the analysis. *β*_*u*_ = TE_uORF,2_/TE_uORF,1_ is the fold change of TE_uORF_ in sample 2 relative to sample 1. Sample pairs with fewer than 70 uORFs having *β*_*u*_ ≠ 1 were not included. *γ* = (TE_CDS,2_ / TE_CDS,1_) / (TE_uORF,2_ / TE_uORF,1_). Significance was determined at an FDR of 0.05. χ^*2*^ tests were performed to compare the differences in the number of uORFs with *β*_*u*_ > 1 and *γ* < 1or *β*_*u*_ < 1 and *γ* > 1 and the number of uORFs with *β*_*u*_ > 1 and *γ* > 1or *β*_*u*_ < 1 and *γ* < 1.

* Percentage of uORFs with *β*_*u*_ > 1 or *β*_*u*_ < 1 among total uORFs in each pair of samples.

** Percentage of uORFs with *γ* > 1 or *γ* < 1 among all uORFs with *β*_*u*_ > 1 or *β*_*u*_ < 1.

Abbreviations: CAGE, cap analysis of gene expression; CDS, coding DNA sequence; FDR, false discovery rate; TE, translational efficiency; uORF, upstream open reading frame.

Note that in the above analyses, we only focused on the impact of individual uORFs and did not consider the possible interactions between uORFs in the same mRNA. Interestingly, in each sample, the number of expressed uORFs in a gene was negatively correlated with the proportion of uORFs that were translated (TE_uORF_ ≥ 0.1) in that gene. This pattern held for all the dominant transcripts that were constitutively expressed across the samples (*P* < 0.05 in each sample, S42 Fig) or for all the genes expressed in each sample (*P* < 5.6 × 10^−7^ in each sample, S43 Fig). Hence, it is possible that there is competition for ribosome occupancy between different uORFs in a gene, and some uORFs tended to have the stronger tendency of ribosome association. Therefore, we also pooled the mRNA or RPF reads of uORFs in the same mRNAs together and examined the relationship between TE changes in uORFs versus those in CDSs (Materials and methods). We still found the changes in TE_uORF_ inversely affect the changes in TE_CDS_ (S14 Table). Taken together, these results suggest that uORFs can change their TE to inversely modulate the translation of the downstream CDSs during *Drosophila* development.

Next, we focused on the genes that switched their major transcripts between neighboring developmental stages to investigate whether the inclusion or exclusion of uORFs would impact the TE of CDSs (Materials and methods). To increase the accuracy in identifying such genes, we analyzed both our mRNA-Seq and the modENCODE mRNA-Seq data and required the same isoform switching events to be detected in both datasets. We found 36–144 (with a median of 83) genes switched the major transcripts, which caused the numbers of expressed uORFs (mRNA RPKM ≥ 1) to be changed between two samples (Fig 6B, Table 4). These results suggest that uORFs might be selectively transcribed during development to regulate the TE of CDSs. For example, *genderblind* (*gb*) encodes a glial glutamate transporter, and male flies with reduced *gb* show strong homosexual courtship [121]. We found a long isoform of *gb* that contains 4 uORFs predominates in female and male adult bodies, while a short isoform without uORFs, which has higher TE compared to the long isoform, is predominantly expressed in female and male adult heads (Fig 6C). The preferential expression of the short, uORF-free isoform in adult heads might maintain high levels of GB protein in the brain. Stage- or tissue-dependent expression of transcript isoforms with different number of uORFs and consistent CAGE signals was also observed for *dichaete* (S44 Fig)—which is a group B Sox-box transcription factor involved in embryo segmentation and nervous system development [122]—and *glycerol kinase 2* (S45 Fig), which is required for glycerol utilization [123]. To systematically probe the regulatory function of selective transcription of uORFs, we investigated the relationship between changes in TE_CDS_ and the change in the number of expressed uORFs between two samples. Overall, in 9 out of 10 pairs of comparisons, the change in TE_CDS_ was negatively correlated with the change in uORF numbers (*P* < 0.05 in 4 comparisons, left panel of Fig 6D).

**Table 4:** The number of genes that switched the major transcripts between neighboring stages or tissues.

Sample 1	Sample 2	Total genes	Genes having CAGE data		Genes having the same TSS		Genes having different TSS	
			genes	Supported by CAGE (%)	Genes having CAGE data	Supported by CAGE (%)	Genes having CAGE data	Supported by CAGE (%)
0–2 h embryos	2–6 h embryos	45	30	30 (100.0)	1	100.0	29	100.0
2–6 h embryos	6–12 h embryos	36	27	25 (92.6)	4	50.0	23	100.0
6–12 h embryos	12–24 h embryos	55	39	39 (100.0)	2	100.0	37	100.0
12–24 h embryos	Larvae	65	51	50 (98.0)	4	100.0	47	97.9
Larvae	Pupae	48	42	39 (92.9)	8	75.0	34	97.1
Pupae	Female bodies	126	102	99 (97.1)	15	80.0	87	100.0
Pupae	Male bodies	113	94	92 (97.9)	12	100.0	82	97.6
Pupae	Female heads	108	78	77 (98.7)	9	88.9	69	100.0
Pupae	Male heads	101	78	75 (96.2)	16	81.3	62	100.0
Female bodies	Male bodies	144	123	117 (95.1)	10	90.0	113	95.6

Only the genes that had the same isoform switched between the same stages/tissues in both the mRNA-Seq data of this study and the modENCODE project were considered.

Abbreviations: CAGE, cap analysis of gene expression; TSS, transcription start site.

One caveat in this analysis is that the switches of major isoforms were heavily based on the gene models that were annotated in FlyBase. Although the high-throughput mRNA-Seq and CAGE-Seq data have been comprehensively incorporated in the genome annotation of *D*. *melanogaster* [124], we cannot exclude the possibility that some of the isoform switching events we detected were affected by the annotations of gene models. Therefore, we further validated the isoform switching events with the profiles of transcriptional start sites identified by the CAGE-Seq data from the modENCODE project. Overall, 92.6%–100% of the isoform switching events were supported by the CAGE signals when the CAGE-Seq data were available for both samples (Table 4). For example, the CAGE signals well supported the altered expression of uORFs in *gb* (S46 Fig), *dichaete* (S47 Fig), and *glycerol kinase 2* (S48 Fig) across stages. Importantly, with only isoform switches that were consistent with CAGE data in each pair of samples, we still observed negative correlations between changes in TE_CDS_ and the change in the number of expressed uORFs in all of the nine pairs (*P* < 0.05 in 3 comparisons, right panel of Fig 6D). Given these observations, we propose that uORFs might be selectively expressed to regulate the translation of the downstream CDSs.

In summary, our results suggest that uORFs play important roles in shaping the translatomes during *Drosophila* development via selective expression or translation.

## Discussion

**Fig 7:**
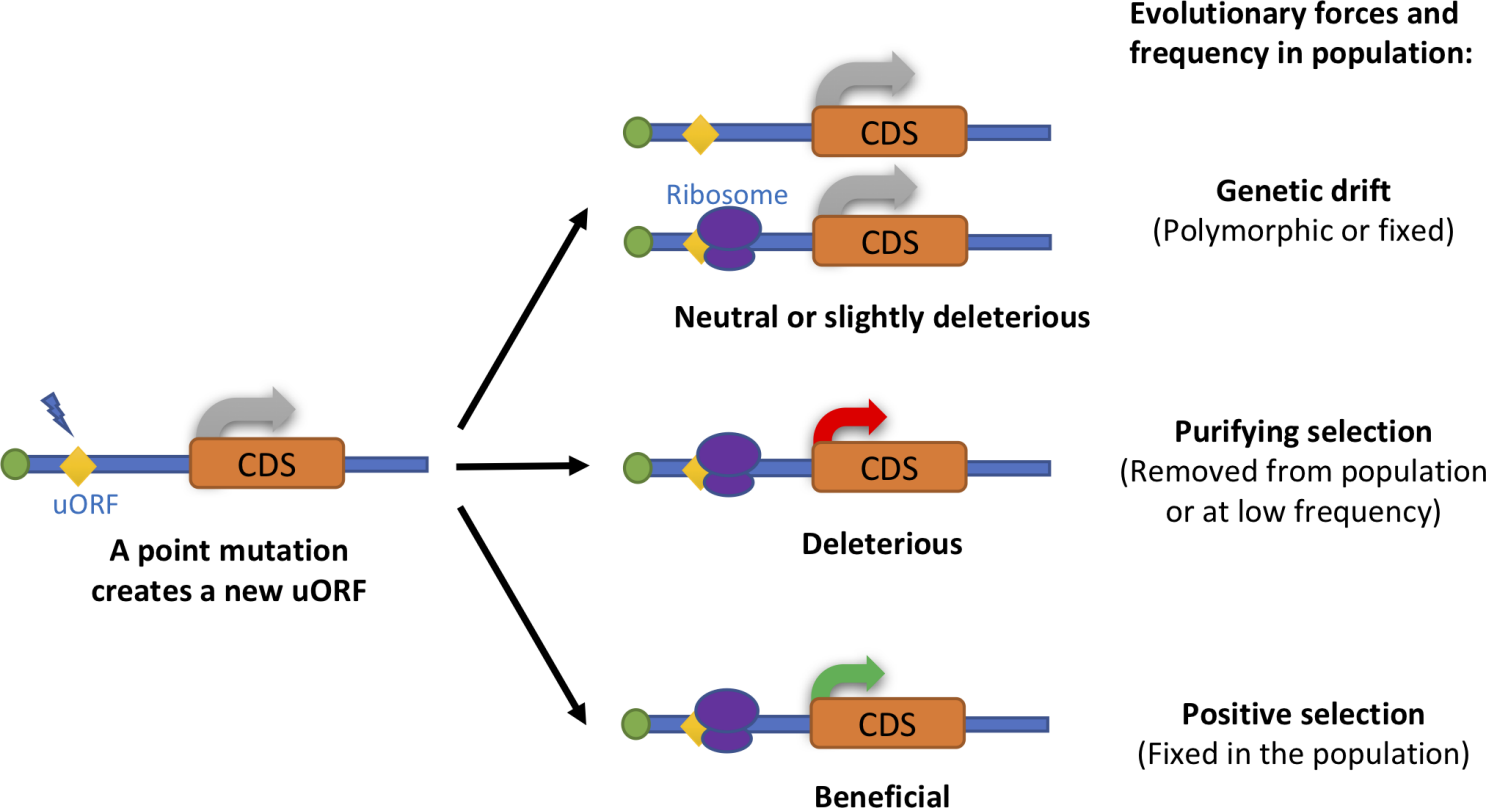
A model of uORF evolution. Mutations frequently generate novel uORFs (uAUGs) in the 5′ UTRs. A newly emerged uORF in the population might be deleterious, neutral, or advantageous. The highly detrimental uORFs are removed by natural selection or persist in the population at low frequencies, whereas the neutral or slightly deleterious ones might randomly drift in the population. The beneficial new uORFs, which often have a higher tendency to be associated with ribosomes, are favored by natural selection and become fixed in the population very rapidly. The newly fixed uORFs, which regulate the translation of their downstream CDSs, are maintained by natural selection during evolution. The fitness effect of uORF-mediated translational repression is represented with arrows: gray, neutral; red, deleterious; green, beneficial. CDS, coding DNA sequence; uAUG, AUG start codon of uORF; uORF, upstream open reading frame; UTR, untranslated region.

In this study, we generated genome-wide maps of ribosome occupancy and TE during the life cycle of *D*. *melanogaster*. Our data allowed us to distinguish the uORFs that show evidence of translation from the putative nonfunctional uORFs. By integrating functional genomic and evolutionary analyses, we for the first time demonstrated that the majority of the newly fixed uORFs in *D*. *melanogaster* were driven by positive Darwinian selection. Herein, we propose a unifying model to describe how natural selection has shaped uORFs during evolution (Fig 7): (1) Frequent nucleotide mutations generate AUG triplets in the 5′ UTRs, giving rise to new uORFs. A newly emerged uORF in the population might be deleterious, neutral, or advantageous. (2) The highly detrimental uORFs are removed by natural selection or persist in the population at low frequencies, whereas the neutral or slightly deleterious ones might randomly drift in the population. (3) The beneficial new uORFs, which often have a higher tendency to be associated with ribosomes, are favored by natural selection and become fixed in the population very rapidly. (4) The newly fixed uORFs, which regulate the translation of their downstream CDSs, are maintained by natural selection and very hard to be lost during evolution. Our model solves the dilemma that (1) uORFs are generally deleterious and selected against, and (2) many uORFs are highly conserved across divergent species.

The newly fixed uORFs with a stronger tendency of ribosome occupancy bear stronger signals of positive selection on the uAUGs. Nevertheless, we also detected signals of positive selection in the uAUGs of the uORFs that did not show compelling evidence of translation (Class IV). In addition, Class IV uORFs also had uAUGs more conserved than flanking triplets and neutral region. It is possible that many of the Class IV uORFs are translated at low levels, but they are beneficial to the hosts. For example, 44.3% (3,842 out of the 8,667) of the Class IV uORFs were associated with at least 1 RPF in our Ribo-Seq dataset, although none of them met the criteria of TE_uORF_ ≥ 0.5. It is also possible that some of the Class IV uORFs are highly translated in other tissues or stages that were not covered in this present study. Moreover, the competition for ribosome association between different uORFs in the same 5′ UTR might cause some uORFs to be weakly translated, although they also contribute to the translational inhibition of the downstream CDSs. Supporting this notion, we found that the proportion of translated uORFs (TE ≥ 0.1) in a gene is negatively correlated with the total number of uORFs in this gene, suggesting that ribosome association at some uORFs will suppress the translation of downstream CDS as well as other uORFs in the same 5′ UTR. Altogether, our results suggest that many newly fixed uAUGs might be favored by natural selection, although the relevant uORFs do not show strong evidence of translation. The large effective population size of *D*. *melanogaster*, which makes natural selection more efficient [125, 126], is crucial for detecting selection signals on the uAUGs. In species with small effective population size, such as humans [125], it might be difficult for natural selection to detect the selective advantages of uORFs. However, human populations experienced frequent gains and losses of uORFs [15, 19, 21], and some uORF-altering mutations cause diseases. Therefore, newly created uORFs in human populations might undergo purifying or positive selection like *D*. *melanogaster*, but the detailed landscape remains to be further determined.

It is known that certain uORFs encode functional peptides [35, 127, 128], and some nascent uORF peptides can even interact with the translating ribosomes to cause ribosome stalling [15, 129]. However, our phyloCSF scores analysis (Fig 3C and 3D) and codon usage bias analysis (S15 Fig) suggest such uORFs are not likely to encode evolutionarily conserved peptides. Our results are consistent with a recent study that detected only about 50 potential coding ORFs in the 5′ UTRs of *Drosophila* [127]. Therefore, we propose that most translational events of uORFs are to compete for ribosomes to impede the translation initiation of the downstream CDSs but not to produce functional peptides.

The systematic characterization of uORFs in this study also allowed us to confirm that genes with ribosome-associated uORFs had reduced TE in *Drosophila*. Moreover, we also found sequence features and conservation patterns of uAUGs are associated with the ability of uORFs to repress translation. Recent studies have revealed that translation of mRNAs is modulated through uORFs in response to stresses [12, 130, 131] or immune induction [132, 133]. Here, we have furthered our understanding of the regulatory roles of uORFs by demonstrating that uORFs could perform regulatory functions in a stage- or tissue-specific manner: (1) changes in TE_uORF_ would inversely influence the TE of the downstream CDSs, even if the dominant transcripts do not change, and (2) inclusion or exclusion of uORFs caused by isoform switching could also modulate the translation of the downstream CDSs. Although the gene expression change caused by uORFs might be weak, the small changes might make a big difference under certain environmental conditions and contribute to phenotypic evolution. Our result is also consistent with recent studies showing how the translation of CDSs is modulated by switching between alternative mRNA isoforms that differ in the content of uORFs through meiotic differentiation in budding yeasts [134–136]. Moreover, many *trans*-acting regulators such as microRNAs [137, 138] and RNA-binding proteins [139] also regulate translation, such as sex lethal (SXL) [140] and density-regulated protein (DENR)–multiple copies in T-cell lymphoma 1 (MCT-1) complex [18]. Recently, the tissue-specific or cell-specific ribosome-profiling technique has been developed [141], which might be helpful to investigate the possible interplays between uORFs and those *trans*-acting regulators in the future.

Taken together, this present study reveals positive Darwinian selection is the major evolutionary force that drives the newly emerged uORFs to fixation. Our functional genomic studies, combined with our evolutionary analyses, shed new light on the molecular mechanisms and functional consequences of uORF-mediated regulation.

## Materials and methods

### Fly materials

The ISO-1 isogenic strain (*y; cn bw sp*) of *D*. *melanogaster*, which was sequenced to assemble the reference genome of *D*. *melanogaster* [117], was used to generate all the libraries in this study. Flies were grown in 12 h light: 12 h dark cycles at 25°C. The 0–2 h, 2–6 h, 6–12 h, and 12–24 h old embryos were collected following a standard protocol at 25°C. Wandering larvae were collected as third-instar larvae. Stage P7–8 pupae were collected approximately 2 d after pupation. The 1–10 d old adult flies were sexed, and the heads and bodies of each sex were separated using brass sieves in liquid nitrogen. The larvae, pupae, and adult heads and bodies were ground into fine powder in liquid nitrogen and then homogenized in the cold room.

### Ribo-Seq and mRNA-Seq

The Ribo-Seq experiments for the 0–2 h, 2–6 h, 6–12 h, and 12–24 h old embryos and for the fine powder of third-instar larvae, pupae, heads, or bodies were performed according to a previous study [64], with some modifications (see S3 Table for key differences). The detailed experimental procedures for Ribo-Seq and for the high-throughput sequencing of mRNAs are fully described in the S1 Text. Two biological replicates of mRNA-Seq and Ribo-Seq (independent sample preparation, library construction, and sequencing under otherwise identical conditions) were prepared for 1–10 d old female bodies and male bodies.

### Harringtonine experiments in S2 cells

*Drosophila* S2 cells were cultured in Schneider's Insect Medium (Sigma-Aldrich) containing 100 U/mL penicillin and 100 μg/ml streptomycin with 10% heat-inactivated fetal bovine serum. The cells were pretreated with 2 μg/ml harringtonine (Sigma-Aldrich, dissolved in DMSO) or DMSO (as control) for 30 min. Then, all cells were treated with 100 μg/ml CHX (cycloheximide, Sigma-Aldrich) for 5 min, washed twice with cold PBS containing 100 μg/ml CHX, and subsequently harvested. The subsequent mRNA-Seq and Ribo-Seq procedures for the S2 cells are presented in the S1 Text.

### Characterizing uORFs with FlyBase and modENCODE data

We identified all the possible ORFs (starting with AUG start codons and ending with UAA/UAG/UGA stop codons) in the mRNA sequences of *D*. *melanogaster* (FlyBase r6.04, http://www.flybase.org) and treated the ORFs with AUG start codons in the 5′ UTRs as uORFs. We did not restrict the length of the uORFs. If a uORF does not overlap with any other uORF on the same transcript, this uORF is classified as a nonoverlapping uORF. If a uORF is in-frame overlapping (i.e., the distance between the two uAUGs is a multiple of 3) or out-of-frame overlapping with at least 1 other uORF or the downstream CDS, this uORF is classified as an overlapping uORF.

We downloaded the mRNA-Seq and matched CAGE data for different developmental stages, tissues, and cells lines of *D*. *melanogaster* that were generated by the modENCODE project [61–63] from Sequence Read Archive (SRA) under accession SRP001602, SRP001065, SRP009459, and SRP000709 (S2 Table). The abundance of annotated transcripts (FlyBase r6.04) in each mRNA-Seq library was determined with kallisto 0.43.1 [74] using default parameters. The NGS reads in both CAGE libraries and mRNA-Seq libraries were mapped to the reference genome of *D*. *melanogaster* using STAR 2.4.2a [142]. We calculated the mRNA-Seq coverage for each nucleotide site as described previously [64] and then calculated the RPKM for a feature (mRNA or uORF) in a sample as $\sum_{k=1}^{L}c_{k}/(L∙N)\times 10^{9}$, where *L* is the length (nucleotides) of that feature, *c*_*k*_ is the mRNA coverage of position *k*, and library size *N* is the total number of mRNA reads uniquely mapped to the transcriptome. For each gene, the RPKM was calculated with the most abundant transcript isoform. The Bam files for alignment of CAGE tags were processed with CAGEr 1.18.1 [143] to identify CAGE tag starting sites (CTSS) in each sample, and CTSSs within 20 bp were merged into a single tag cluster. CAGE tag clusters with fewer than 5 raw reads at dominant CTSS were excluded. The boundary of a tag cluster was calculated as 10% and 90% quantile positions of the distribution of CAGE tags in this cluster. Each tag cluster was assigned to the nearest transcript within 500 bp with bedtools “closest” [144]. To identify uORFs expressed in each sample, we required that (1) the gene containing a uORF should be detected with mRNA-Seq RPKM ≥ 1; (2) the uORF itself should also have RPKM ≥ 1 in the mRNA-Seq data; (3) in case CAGE signals were detected for this gene, the uORF should be located in transcript isoform supported by CAGE tags and at downstream of 3′ boundary of the dominant CAGE tag cluster for this transcript. Under these criteria, we identified 37,619 uORFs that were expressed in at least 1 sample of modENCODE data among all the 41,483 putative uORFs.

To estimate the expected number of uORFs under the assumption of randomness, the 5′ UTR sequences of the longest transcripts of the protein-coding genes were randomly permutated while maintaining the same dinucleotide frequencies with uShuffle [145]. The permutation procedures were repeated for 1,000 replicates. The median, 2.5% and 97.5% quantiles of the numbers of uORFs in the shuffled sequences were tabulated.

### NGS data processing, *H*_*g*_ index, and metagene profile analysis

After removing 3′ adaptors [146] and quality controls, the NGS reads of the mRNA-Seq and Ribo-Seq experiments were mapped to the reference genome of *D*. *melanogaster* (FlyBase, r6.04) using STAR 2.4.2a. In each sample, we assigned a mapped RPF (27–34 nt in length) to its P-site and calculated the RPKM values for a feature (CDS or uORF) with the mRNA or PPF data as previously described [64]. For uORFs that were overlapping with CDSs, only the nonoverlapping regions of the uORFs were used in calculating RPKM. The TE for a feature (CDS or uORF) was calculated as the ratio of RPF RPKM over mRNA RPKM [34, 147]. In each sample, the most abundant transcript in mRNA-Seq for each gene was inferred with kallisto 0.43.1, and only the genes with mRNA RPKM ≥ 1 in the CDS were considered, unless otherwise stated. The tissue specificity index *H*_*g*_ for a CDS or uORF was calculated as previously described [73]. For each mRNA-Seq or Ribo-Seq library, we followed a published procedure [37] to build the metagene profile around start codons by calculating the coverage of a 51-triplet window (including the start codon itself, 10 upstream triplets, and 40 downstream codons) for each cAUG or a 16-triplet window (including the start codon itself, 5 upstream triplets, and 10 downstream triplets) for each uAUG. The detailed analytical procedures are described in the S1 Text.

### Calculation of phyloP, phyloCSF, BLS, and Kozak score

Basewise phyloP scores of *D*. *melanogaster* were downloaded from UCSC genome browser (genome.ucsc.edu) [148], and the phyloP score for each uAUG was extracted with bigWigAverageOverBed [149]. To calculate phyloCSF [88] and BLS [119] of uORFs, the 27-way multiple sequence alignments of *D*. *melanogaster* (dm6) against 26 insect species and the corresponding phylogenetic tree was downloaded from UCSC genome browser [148]. The alignments for uORFs among 23 *Drosophila* species were extracted and stitched together using custom scripts. The PhyloCSF software [88] was used to evaluate each alignment with the parameter “23flies --removeRefGaps --bls --ancComp --aa --files”. For the 13,282 protein-coding genes in *D*. *melanogaster* (the longest transcript isoform was used for each gene), we retrieved the −6 to 1 nucleotides around each cAUG and derived a PPM for Kozak sequence contexts (S10 Table). Then, we calculated the Kozak score for each uAUG or cAUG as a log-odds ratio [150]: Σ_*i*_[log_2_(*P*_*i*,*j*_/0.25)], where *P*_*i*,*j*_ is the probability of observing a nucleotide *j* (A, U, C, and G) at position *i* (−6 to 1) (S10 Table).

### Conservation of AUG triplets in genomic alignments of three *Drosophila* species

To find differences in AUG triplets in the 5′ UTRs and in positions 8–30 nt of short introns (≤65 nt) among *D*. *melanogaster*, *D*. *sechellia*, and *D*. *yakuba*, we extracted and stitched alignments of these regions from the 27-way multiple alignments, using custom scripts, and searched for differences in ATG triplets in regions of interest. The gains and losses of the AUG triplets were inferred using a parsimonious method based on the phylogenetic tree of the three species (Fig 4D).

### Polymorphisms of AUG triplets in *D*. *melanogaster*

We tabulated all the SNPs that cause AUG triplet differences in the genetic variation data from GDL [95] and DGRP2 [101]. We polarized each mutation in *D*. *melanogaster* by comparing the orthologous site in *D*. *sechellia* with LiftOver [151] based on the pairwise alignments of *D*. *melanogaster* and *D*. *sechellia* that were downloaded from the UCSC genome browser.

### MK test on newly emerged *D*. *melanogaster* uAUGs

We investigated each AUG triplet in the reference genome of *D*. *melanogaster* to verify whether it was newly created in the *D*. *melanogaster* linage (fixed or polymorphic in the extant populations of *D*. *melanogaster*). This was done by comparing each AUG triplet with the orthologous sites in *D*. *sechellia* and *D*. *yakuba* (Fig 4D). Next, we masked the AUG triplets that were located in repetitive regions identified by RepeatMasker (http://www.repeatmasker.org) or that overlapped with CDS regions of other transcripts. For the remaining AUG triplets in 5′ UTRs (uORFs) or in positions 8–30 nt of short introns (neutral regions), we examined whether they were polymorphic in the GDL or DGRP2 databases (we required the polymorphic AUG triplets to have MAF of ≥0.05 as previously described [109]) or fixed in the *D*. *melanogaster* lineage. We employed Kimura’s 2-Parameter model [152] to correct for multiple substitutions for the fixed differences. The proportion of newly fixed uAUGs driven by positive selection was calculated as $\alpha_{ori}=1-\frac{D_{SI}∙P_{RI}}{P_{SI}∙D_{RI}}$, where *D* is the fixed difference, and *P* is the polymorphic difference. SI stands for 8–30 nt of short introns (≤65 nt), and RI stands for regions of interest [106].

We also used the AsymptoticMK (https://github.com/MesserLab/asymptoticMK) [114] to estimate *α*_*asym*_ [112]. Briefly, the number of fixed and polymorphic sites was derived as described above. The polymorphic sites in neutral control regions were grouped into bins of equal size based on increasing derived allele frequency, and the same break points were used to divide the polymorphic sites in test region into different bins. Only bins whose derived allele frequencies were within 0.05 and 0.95 were used to estimate *α*_*asym*_ as a function of derived allele frequency. In both the original and AsymptoticMK tests, we not only estimated the *α* values using all the polymorphic data meeting the cutoff criteria but also estimated the *α* values by requiring the mutations to be present in the ISO-1 strain of *D*. *melanogaster*. Both the original and AsymptoticMK tests were also applied to mutations that created uAUGs of Classes I, II, and III (combined) using AUGs in 3′ UTR as neutral controls.

To assess the influence of pooling loci from different genomic regions that differ in the effective population size, we randomly sampled the same number of newly fixed and polymorphic AUG triplets with replacement for newly fixed AUGs or polymorphic AUGs in 5′ UTRs or 8–30 nt of short introns, respectively. Then, we performed the original MK test and the AsymptoticMK analysis. This procedure was repeated for 1,000 replicates, and the median and the 95% CI of *α*_*ori*_ and *α*_*asym*_ were estimated. To evaluate the effect of estimating *α* values by requiring the mutations to be present in the ISO-1 strain, we first followed the procedure described in [114] to estimate the *α* values on the fixed nonsynonymous mutations in 1,000 randomly selected genes of *D*. *melanogaster*, using the synonymous mutations as neutral controls, and then we conducted the same analysis except that we required the mutations to be present in the ISO-1 strain. This procedure was performed for 1,000 replicates to obtain CI.

We estimated *α*_*dfe*_ [110] using the DFE-alpha program (http://www.homepages.ed.ac.uk/pkeightl/dfe_alpha/download-dfe-alpha.html, version 2.15). For the test and neutral regions, the number of triplets that could be mutated into an ATG triplet by a single point mutation was counted. These numbers were further adjusted for multiple hits in the same triplet based on the proportion of ATG-creating mutations that were newly fixed in *D*. *melanogaster* lineage to derive the number of background sites. The polymorphic sites with fewer than 150 alleles in GDL dataset (130 alleles for DGRP dataset) were excluded. For each of the remaining sites, 150 alleles were randomly sampled without replacement to calculate the unfolded SFS in the test and neutral regions, which were then used to estimate DFE with “est_dfe” program in DFE-alpha [110, 153]. A two-epoch model, in which the population size changed from *N1* to *N2 T2* generations ago, was used during estimation. While *N1* was a fixed number, *N2* and *T2* were searched through maximum likelihood estimation. The *α*_*dfe*_ was estimated based on the DFE using the “est_alpha_omega” program in DFE-alpha. The parameters “do_jukes_cantor” and “remove_poly” were set to 0, as the number of fixed sites was already corrected for multiple hits, and polymorphic sites had been removed from fixed sites as described above.

### Statistical modeling of RPF and mRNA read counts with negative binomial distribution

In each sample, we counted the mRNA-Seq reads that were overlapping with a feature (CDS or uORF) and calculated the RPF read count as $\lceil \sum_{k=1}^{L}c_{k}\rceil$, where *L* is the length (nt) of that feature, and *c*_*k*_ is the P-site coverage of RPFs at position *k*. For a sample, we used DESeq2 [66] to determine the size factors of the mRNA-Seq and Ribo-Seq libraries and normalized the mRNA or RPF read counts by dividing the raw counts with the corresponding size factors (S15 Table). The normalized read counts were used throughout the statistical modeling procedures.

We modeled the mRNA or RPF read count *K*_*ij*_ for a feature (CDS or uORF) *i* in a biological replicate *j* (*j =* 1 or 2) with a negative binomial distribution with mean *μ*_*ij*_ and dispersion *φ*_*i*_ as previously described [66–70]. Based on the two biological replicates in female bodies (or male bodies), for the mRNA or RPF data, we first used the “estimateDispersionsGeneEst” function in DESeq2 to estimate the featurewise *φ* values and then used the “estimateDispersionFit” function in DESeq2 to fit *φ* as a function of *μ* (i.e., *φ*(*μ*)) for each type of data (S3A Fig). Here, we only considered the well-transcribed features (RPKM ≥ 1 and normalized counts ≥ 30 in mRNA-Seq) in estimating dispersion. We also analyzed the features (CDSs or uORFs) that were expressed in both female and male bodies and estimated the overall dispersion trend of RPFs (*φ*_*R*_) or mRNA reads (*φ*_*M*_) while taking gender information into consideration (S3A Fig). Note that the dispersion trends are very similar when we considered female bodies and male bodies separately or jointly (S3A Fig).

For other samples that did not have biological replicates, we assumed the mRNA or RPF read count for a feature follows a negative binomial distribution with the same dispersion trend (*φ*_*M*_ or *φ*_*R*_) that was estimated from the biological replicates of female and male bodies.

### Evaluating the statistical differences in TE between uORFs and the downstream CDSs

For a well-transcribed uORF *i* and its downstream CDS *i* in a sample, we denoted the ratio TE_uORF,*i*_/TE_CDS,*i*_ as *β*_*i*_ and tested whether log_2_(*β*_*i*_) = log_2_(TE_uORF,*i*_)–log_2_(TE_CDS,*i*_) is significantly different from 0 in a sample with Wald test. We assumed the log_2_(TE) value of a feature (CDS or uORF) follows a normal distribution, which well approximated the observed distribution of log_2_(TE) obtained with mRNA and RPF counts simulated with negative binomial distributions (S49 Fig). With the biological replicates in female bodies (or male bodies), we contrasted the RPF counts against mRNA-Seq read counts with DESeq2 to estimate the log_2_(TE) and SE of log_2_(TE) values for each feature. Then we fitted the SE values against the normalized mRNA counts and log_2_(TE), using the “gam” function (in the R package “mgcv”) with a log link to obtain a smooth surface (S10 Fig). For other samples that did not have biological replicates, we derived the SE of log_2_(TE) for a feature (CDS or uORF) by subjecting the observed mRNA counts and log_2_(TE) to the fitted surface obtained based on the biological replicates of female and male bodies. Once the SE values of the uORF *i* and the CDS *i* were estimated, the SE of log_2_(*β*_*i*_) can be derived as

$${SE}_{{log}_{2}(\beta_{i})}=\sqrt{{SE}_{{{log}_{2}(TE}_{uORF,i})}^{2}+{SE}_{{{log}_{2}(TE}_{CDS,i})}^{2}}.$$

As the Wald statistic $\frac{{log}_{2}(\beta_{i})}{{SE}_{{log}_{2}(\beta_{i})}}$ follows a standard normal distribution under the null hypothesis that log_2_(*β*_*i*_) = 0, we calculated the *P* value with $2∙(1-\Phi \left(|\frac{{log}_{2}(\beta_{i})}{{SE}_{{log}_{2}(\beta_{i})}}|\right))$. Note that occasionally the TE values we estimated based on the normalized counts of RPFs and mRNA reads are slightly different from those calculated using the RPKM method as previously described [34, 64, 71]. Throughout this study, we only considered the difference that showed the same trend in both methods when we compared the TE values of two features or compared the TE values of a feature in different samples.

### Estimating the probability that a well-transcribed uORF with 0 RPF reads is translated

For a uORF *i* that is expressed with *K*_*im*_ normalized mRNA reads but not covered by any RPF in a sample *m*, we calculated *P*_*m*_(*R*_*0*_), the probability that this uORF is translated. Under the null hypothesis *H*_*0*_(*c*), we assumed the expected TE of this uORF (*x*) is the same as that of the downstream CDS (TE_CDS,*i*_, see S13 Fig). We first estimated the prior distribution of *x* and *K*_*im*_. By assuming the log_2_(TE_CDS,*i*_) follows a normal distribution, we can obtain the prior distribution of *x* under *H*_*0*_(*c*) as

$$f(x)=f_{Norm}({log}_{2}\left(x\right);{log}_{2}({TE}_{CDS,i}),2{SE}_{{log}_{2}({TE}_{CDS,i})}^{2})=\frac{1}{2\sqrt{\pi }{SE}_{{log}_{2}({TE}_{CDS,i})}}e^{-\frac{{\left(log2(x)-{log}_{2}({TE}_{CDS,i})\right)}^{2}}{4{SE}_{{log}_{2}({TE}_{CDS,i})}^{2}}}$$

where ${SE}_{{{log}_{2}(TE}_{CDS,i})}$ is the *SE* of log_2_(TE_CDS,*i*_) and estimated as described above. *K*_*im*_ follows a negative binomial distribution with the formula

$$f_{NB}(K_{im};\mu_{im},\varphi_{M}(\mu_{im}))=\frac{\Gamma (K_{im}+{\varphi_{M}(\mu_{im})}^{-1})}{\Gamma (K_{im}+1)\Gamma ({\varphi_{M}(\mu_{im})}^{-1})}{\left(\frac{1}{1+\varphi_{M}(\mu_{im})\mu_{im}}\right)}^{{\varphi_{M}(\mu_{im})}^{-1}}{\left(\frac{\varphi_{M}(\mu_{im})\mu_{im}}{1+\varphi_{M}(\mu_{im})\mu_{im}}\right)}^{K_{im}},$$

where *φ*_*M*_ is the dispersion trend of mRNA read counts estimated above. By modeling the RPF count of the uORF using a negative binominal distribution with mean *xK*_*im*_ at given *x* and *K*_*im*_, we can derive the posterior probability of observing 0 RPF reads as

$$P_{m}(R_{0})=\int f(x)∙\sum_{K_{im}\geq 0}f_{NB}(K_{im};\mu_{im},\varphi_{M}(\mu_{im}))f_{NB}\left(0;xK_{im},\varphi_{R}(xK_{im}\right))dx,$$

where *φ*_*R*_ is the dispersion trend of RPF counts estimated above. Using the similar approach, we also estimated *P*_*m*_(*R*_*0*_) under two other null hypotheses: (1) *H*_*0*_(*u*): *x* is the average TE (*u)* of the uORF in at least 2 other samples in which the uORF is well expressed (≥30 normalized mRNA reads and ≥3 normalized RPF reads); and (2) *H*_*0*_(*0*.*1*): *x* has a fixed value of 0.1.

### Evaluating the statistical significance that the changes in TE_uORF_ inversely affect the magnitude of changes in TE_CDS_ between two samples

For a uORF and its downstream CDS in an mRNA that dominates in both sample 1 and 2, we denoted *β*_*u*_ = TE_uORF,2_/TE_uORF,1_ and examined whether log_2_(*β*_*u*_) is significantly different from 0 using the Wald test as above described (S10 Fig). Then, we defined γ = (TE_CDS,2_ / TE_CDS,1_) / (TE_uORF,2_ / TE_uORF,1_) and tested whether log_2_(γ) is significantly different from 0 (S41 Fig). We modeled the log_2_(TE_uORF,1_), log_2_(TE_CDS,1_), log_2_(TE_uORF,2_), and log_2_(TE_CDS,2_) with normal distributions and estimated ${SE}_{{{log}_{2}(TE}_{uORF,1})},\;{SE}_{{{log}_{2}(TE}_{CDS,1})},\;{SE}_{{{log}_{2}(TE}_{uORF,2})}$, and ${SE}_{{{log}_{2}(TE}_{CDS,2})}$ with the biological replicates from female and male bodies. Thus, log_2_(γ) also follows a normal distribution with

$${SE}_{{log}_{2}(\gamma )}=\sqrt{{SE}_{{{log}_{2}(TE}_{uORF,1})}^{2}+{SE}_{{{log}_{2}(TE}_{CDS,1})}^{2}+{SE}_{{{log}_{2}(TE}_{uORF,2})}^{2}+{SE}_{{{log}_{2}(TE}_{CDS,2})}^{2}}.$$

Therefore, we calculated the *P* value under the null hypothesis log_2_(γ) = 0 with $2∙(1-\Phi \left(|\frac{{log}_{2}(\gamma )}{{SE}_{{log}_{2}(\gamma )}}|\right))$. For each sample, the same analysis was also performed after pooling mRNA or RPF reads of all the uORFs in the same dominant isoform.

### Reference accessions

For the mature oocytes and activated eggs of *D*. *melanogaster*, the raw sequencing data were downloaded from Gene Expression Omnibus (GEO) with accession number GSE52799 [65]. The Ribo-Seq of S2 cells at different ion concentrations and mRNA-Seq and Ribo-Seq of 0–2 h fly embryos were downloaded from GEO with GSE49197 [64].

### Data accession

All deep-sequencing data generated in this study were deposited in the Sequence Read Archive (SRA) under accession number SRP067542. The numeric values underlying the main figures and supplementary figures can be found in S1–S8 Data.

## Supporting information

S1 Text Supplementary methods. (PDF)

S1 Table List of the 37,619 uORFs supported by modENCODE mRNA-Seq and CAGE data. CAGE, cap analysis of gene expression; uORF, upstream open reading frame.
(XLSX)

S2 Table The numbers of genes and uORFs expressed in 34 modENCODE samples that have both CAGE-Seq and mRNA-Seq data available. CAGE, cap analysis of gene expression; uORF, upstream open reading frame.
(DOCX)

S3 Table Summary of treatment, inhibitors, and ribonuclease used in mRNA-Seq and Ribo-Seq library constructions. (DOCX)

S4 Table Mapping statistics of mRNA-Seq and Ribo-Seq libraries. (DOCX)

S5 Table Gene ontology analysis of genes without or with ribosome-associated uORFs (TE_uORF_ ≥ 0.5). TE, translational efficiency; uORF, upstream open reading frame.
(DOCX)

S6 Table The numbers and proportions of expressed uORFs that are overlapping with other uORFs or downstream CDSs. CDS, coding DNA sequence; uORF, upstream open reading frame
(DOCX)

S7 Table The proportions of nonoverlapping and overlapping uORFs that are translated (TE_uORF_ ≥ 0.5). TE, translational efficiency; uORF, upstream open reading frame.
(DOCX)

S8 Table The major transcripts in the mRNA-Seq data generated in this study and the cross-validation by the modENCODE mRNA-Seq data. (DOCX)

S9 Table Summary of uORFs that are well transcribed but not translated in at least 1 sample. uORF, upstream open reading frame.
(DOCX)

S10 Table Position probability matrix for Kozak sequence context around the cAUGs in *D*. *melanogaster*. cAUG, AUG start codon of coding DNA sequence.
(DOCX)

S11 Table The number of mutations creating newly fixed uAUGs (K80 adjusted) or polymorphic uAUGs (MAF ≥ 0.05) and *α*_*ori*_ for uORFs of different classes. MAF, minor allele frequency; uAUG, AUG start codon of uORF; uORF, upstream open reading frame.
(DOCX)

S12 Table Summary of multiple linear regression of log_2_(TE_CDS_) against features of 5′ UTRs and relative importance of these features. CDS, coding DNA sequence; TE, translational efficiency; UTR, untranslated region.
(DOCX)

S13 Table Summary of multiple linear regression of log_2_(TE_CDS_) against features of uORFs in each sample. CDS, coding DNA sequence; TE, translational efficiency; uORF, upstream open reading frame.
(DOCX)

S14 Table Summary of genes whose TE_uORF_ (pooled) changed disproportionally relative to the downstream CDSs between two neighboring developmental stages. CDS, coding DNA sequence; TE, translational efficiency; uORF, upstream open reading frame.
(DOCX)

S15 Table The size factor for mRNA-Seq or Ribo-Seq library and raw reads required to reach a normalized mRNA read count of 30 in each sample. (DOCX)

S16 Table Oligos used in this study. (DOCX)

S1 Fig Profiles of polysomes (undigested) and monosomes (digested with MNase) of fly samples. au, arbitrary unit; MNase, micrococcal nuclease.
(PDF)

S2 Fig The correlations between two biological replicates of 1–10 d old female bodies and male bodies in the mRNA-Seq and Ribo-Seq experiments. (A) High correlations in RPKM of CDSs between the two biological replicates. (B) Correlations in RPKM of uORFs between the two biological replicates. (C) Correlations in RPKM of 5′ regions of CDSs that begin at the start codons and end at the same lengths of uORFs in the 5′ UTRs. The raw data for panels (A-C) can be found in S5 Data. CDS, coding DNA sequence; RPKM, reads per kilobase of transcript per million mapped reads; uORF, upstream open reading frame; UTR, untranslated region.
(PDF)

S3 Fig The estimated dispersion of NGS read counts for CDSs and uORFs in the biological replicates of female and male bodies. (A) Maximum likelihood estimation of dispersions of mRNA or RPF read counts for CDSs (red) and uORFs (black) in biological replicates of female bodies, male bodies, or combined data. Dispersions of mRNA read counts were estimated for features (CDSs and uORFs) with mRNA RPKM ≥ 1. Dispersions of RPF counts were only estimated for well-transcribed features. (RPKM ≥ 1 and normalized reads ≥ 30 in mRNA-Seq). The blue lines are fit of dispersions against average read counts of biological replicates and reflect the dispersion-mean dependency. (B) The distribution of dispersions of NGS read counts for uORFs and CDSs as shown in (A). The raw data for panels (A and B) can be found in S6 Data. CDS, coding DNA sequence; NGS, next-generation sequencing; RPF, ribosome-protected mRNA fragment; RPKM, reads per kilobase of transcript per million mapped reads; uORF, upstream open reading frame; UTR, untranslated region.
(PDF)

S4 Fig The relative positions of the 5′ ends of RPFs in the three frames of codons in all Ribo-Seq libraries generated in this study and previous studies. The enzymes used in digestion were presented under the bars. The 3 nt periodicity of the 5′ RPF reads mapped along CDSs is readily manifested in the RNase I experiment [65] but compromised in the MNase experiments by Dunn and colleagues [64] and Ribo-Seq data generated in this study. The raw data can be found in S1 Data. CDS, coding DNA sequence; MNase, micrococcal nuclease; RPF, ribosome-protected mRNA fragment.
(PDF)

S5 Fig Site coverage for the 5′ end positions of uniquely mapped RPF reads around the cAUGs in each Ribo-Seq library. The raw data can be found in S1 Data. cAUG, AUG start codon of coding DNA sequence; RPF, ribosome-protected mRNA fragment.
(PDF)

S6 Fig The normalized coverage around the cAUGs and the uAUGs in each sample. The blue line and red line represent mRNA-Seq and Ribo-Seq of each sample, respectively. cAUG, AUG start codon of coding DNA sequence; uAUG, start codon of upstream open reading frame.
(PDF)

S7 Fig The distribution of log_2_(TE) for a feature (CDS or uORF) in each library. Only a feature that has mRNA RPKM ≥ 1 was considered. The left panel is the log_2_(TE) for all the features (CDSs and uORFs combined), and the log_2_(TE) for the uORFs and CDSs in a sample was drawn separately in the right panel. CDS, coding DNA sequence; RPKM, reads per kilobase of transcript per million mapped reads; TE, translational efficiency; uORF, upstream open reading frame.
(PDF)

S8 Fig The distribution of log_2_(TE) (left panel) and the proportion with TE ≥ 0.1 (middle panel) or 0.5 (right panel) for CDSs and uORFs in CEGs or NCEGs, respectively. CEGs: genes that are expressed with mRNA-Seq RPKM ≥ 1 in all 12 samples. NCEGs: genes that are not constitutively expressed in all the samples but expressed with mRNA-Seq RPKM ≥ 1 in at least 1 of the 12 samples. The number of expressed genes or uORFs are displayed beside each box in the left panel. The raw data can be found in S7 Data. CDS, coding DNA sequence; CEG, constitutively expressed gene; NCEG, nonconstitutively expressed gene; RPKM, reads per kilobase of transcript per million mapped reads; TE, translational efficiency; uORF, upstream open reading frame.
(PDF)

S9 Fig **The GO enrichment analysis of genes without ribosome-associated uORFs (A) or genes with ribosome-associated uORFs (B).** The raw data can be found in S5 Table. GO, gene ontology; uORF, upstream open reading frame.
(PDF)

S10 Fig The scheme of evaluating the statistical differences in TE between uORFs and the downstream CDSs. For a uORF *i* and its downstream CDS *i* in a sample, we assumed log_2_(TE_uORF,*i*_) and log_2_(TE_CDS,*i*_) follow normal distributions. For female bodies and male bodies, we estimated the SE values of log_2_(TE) of a feature (CDS or uORF) by contrasting the RPF counts against the mRNA counts of biological replicates with DESeq2 [66]. Then, we fitted a smooth surface of SE of log_2_(TE) against the mRNA counts and log_2_(TE) of features. For a sample without biological replicates, the SE value of log_2_(TE) for each feature is inferred from the smooth surface obtained with biological replicates of female and male bodies. Then, we denoted TE_uORF,*i*_/TE_CDS,*i*_ as *β*_*i*_ and estimated the SE value of log_2_(*β*_*i*_). The *P* value under the null hypothesis log_2_(*β*_*i*_) = 0 was obtained with a two-tailed Wald test. The raw data can be found in S7 Data. CDS, coding DNA sequence; RFP, ribosome-protected mRNA fragment; SE, standard error; TE, translational efficiency; uORF, upstream open reading frame.
(PDF)

S11 Fig The relationship between TE of uORFs and the downstream CDSs for 9,162 uORFs that are constitutively expressed in all 12 samples. Each row represents a uORF, and each column represents a sample. uORFs that are well transcribed (RPKM ≥ 1 and normalized reads ≥ 30 in mRNA-Seq) and have *β* > 1 or *β* < 1 in a sample at the FDR of 0.05 are shown in red and blue, respectively. uORFs that are well-transcribed but not translated in a sample under *H*_*0*_(*c*) at the FDR of 0.05 are shown in black. Well-transcribed uORFs that have no significant differences in TE compared to the downstream CDSs in a sample are shown in gray. The remaining uORFs that are not well transcribed are shown in white. The raw data can be found in S7 Data. CDS, coding DNA sequence; FDR, false discovery rate; RPKM, reads per kilobase of transcript per million mapped reads; TE, translational efficiency; uORF, upstream open reading frame.
(PDF)

S12 Fig **Differences in *H*_*g*_ between uORFs and the corresponding 5′ parts of CDSs in mRNA-Seq (left) and Ribo-Seq (right) data, respectively.** For each uORF of *n* codons in length, the RPKM for the 5′ part of the downstream CDS was calculated for a region of *n* codons beginning from the downstream cAUG in both mRNA-Seq and Ribo-Seq data. *H*_*g*_ was calculated based on the RPKM for the uORFs or the 5′ parts of CDSs in the mRNA-Seq and Ribo-Seq data, respectively. Note in both mRNA-Seq and Ribo-Seq that the uORFs have significantly lower *H*_*g*_ values compared to the 5′ parts of CDSs. The raw data can be found in S1 Data. The raw data can be found in S7 Data. CDS, coding DNA sequence; RPKM, reads per kilobase of transcript per million mapped reads; uORF, upstream open reading frame.
(PDF)

S13 Fig The scheme of estimating the *P* value that a well-transcribed uORF with 0 RPF reads is not translated. For a uORF *i* that is expressed with *K*_*im*_ normalized mRNA reads but not covered by any RPF in a sample *m*, we assumed the expected TE of this uORF (*x*) is the same as that of the downstream CDS (TE_CDS,*i*_) under the null hypothesis *H*_*0*_(*c*). Then, we estimated the prior distribution of *x* with the smooth surface of SE values of log_2_(TE) against the mRNA counts and log_2_(TE) of well-transcribed features in biological replicates of female and male bodies. The prior distribution of *K*_*im*_ was also estimated with the dispersion trend of mRNA counts in biological replicates of female and male bodies. By modeling the RPF count of the uORF using a negative binominal distribution with mean *xK*_*im*_ at given *x* and *K*_*im*_, we can derive the posterior probability of observing 0 RPF reads *P*_*m*_(*R*_*0*_) under null hypothesis *H*_*0*_(*c*). CDS, coding DNA sequence; RPF, ribosome-protected mRNA fragment; SE, standard error; TE, translational efficiency; uORF, upstream open reading frame.
(PDF)

S14 Fig uORFs with higher Kozak score and a shorter distance from uAUG to cAUG have higher TE. (A) Positive correlations between Kozak score of uAUG (x-axis) and log_2_(TE) (y-axis) of ribosome-associated uORFs in each of the 12 samples. The ribosome-associated uORFs were ranked with increasing Kozak score and were divided into 200 bins of equal size. Median Kozak score and median log_2_(TE) in each bin were displayed in the plot and used to calculate Spearman’s correlation. In each sample, only uORFs in genes with mRNA RPKM ≥ 1 and TE ≥ 0.5 were used in the analysis. (B) Negative correlations between the distance from uAUG to cAUG (x-axis) and log_2_(TE) (y-axis) of ribosome-associated uORFs in each of the 12 samples. The ribosome-associated uORFs were ranked with increasing distance from uAUG to cAUG and were divided into 200 bins of equal size. Median distance from uAUG to cAUG (log10 transformed) and median log_2_(TE) in each bin were displayed in the plot and used to calculate Spearman’s correlation. In each sample, only uORFs in genes with mRNA RPKM ≥ 1 and TE ≥ 0.5 were used in the analysis. The raw data for panels (A and B) can be found in S2 Data. cAUG, AUG start codon of coding DNA sequence; RPKM, reads per kilobase of transcript per million mapped reads; TE, translational efficiency; uAUG, AUG start codon of upstream open reading frame; uORF, upstream open reading frame.
(PDF)

S15 Fig The RSCU of CDSs, 5′ UTRs, and uORFs of *D*. *melanogaster*. For uORFs and CDSs, both the start and stop codons are excluded. For uORFs, the regions overlapping with CDSs are also excluded. For each 5′ UTR, first reading frame of entire 5′ UTR is used to calculate triplet frequencies (stop codons were excluded). RSCU calculation was based on [89]. The raw data can be found in S1 Data. CDS, coding DNA sequence; RSCU, relative synonymous codon usage; uORF, upstream open reading frame; UTR, untranslated region.
(PDF)

S16 Fig The distribution of phyloP scores for uAUGs of uORFs that have different RPF densities (uORFs were divided into three categories based on increasing RPF RPKM: <1, 1–20, and >20) in each sample. The raw data can be found in S8 Data. RPF, ribosome-protected mRNA fragment; RPKM, reads per kilobase of transcript per million mapped reads; uAUG, AUG start codon of uORF; uORF, upstream open reading frame.
(PDF)

S17 Fig Evolutionary analysis of uAUGs with DGRP dataset. (A) The derived allele frequency of uAUGs (from Classes I to IV) that are polymorphic in *D*. *melanogaster* (***, *P* < 0.001). The raw data can be found in S1 Data. (B) Frequencies of the derived mutations that cause the gain or loss of uORFs in the 5′ UTR, the remaining derived mutations in the 5′ UTR, and the derived mutations in positions 8–30 nt of short introns in *D*. *melanogaster* (***, *P* < 0.001). The raw data can be found in S3 Data. DGRP, *Drosophila* Genetic Reference Panel; uAUG, AUG start codon of uORF; uORF, upstream open reading frame.
(PDF)

S18 Fig **The estimation of *α***_***asym***_ **for newly fixed mutations in uAUGs by AsymptoticMK for the GDL (left) and DGRP (right) data.** AUGs in 8–30 nt of short introns were used as the neutral control, and all mutations in the populations were used. The *α*_*ori*_ (the dashed line) was estimated with polymorphic sites whose derived allele frequencies were within 0.05–0.95 (delineated by the blue lines). To estimate *α*_*asym*_, the *α* values were calculated with polymorphic sites of different derived allele frequencies (*x*). An exponential function was fitted to the *α* values (red line). Gray bars denote the 95% confidence of *α*_*asym*_. The input for AsymptoticMK can be found in S1 Data. DGRP, *Drosophila* Genetic Reference Panel; GDL, Global Diversity Lines; uAUG, start codon of upstream open reading frame.
(PDF)

S19 Fig The MK test for the newly fixed nonsynonymous mutations in CDSs of randomly selected 1,000 genes using all mutations in the populations or only those present in the ISO-1 strain of *D*. *melanogaster*. DGRP data were used in analysis, and both the original method and AsymptoticMK tests were performed. The simulations were repeated for 1,000 replicates. (A) The distribution of *α*_*ori*_ (left panel) estimated with all mutations in the populations (red) or only those present in ISO-1 strain (blue) and their ratios (the later versus the former, right panel). The median and the 2.5% and 97.5% quantiles of the ratios were shown and indicated with dashed lines. (B) Same as A but showing the results for *α*_*asym*_. The raw data can be found in S1 Data. CDS, coding DNA sequence; DGRP, *Drosophila* Genetic Reference Panel; MK test, McDonald-Kreitman test.
(PDF)

S20 Fig The *α*_*asym*_ for mutations in uAUGs of Classes I and II (combined), III, and IV in DGRP data. AUGs in 8–30 nt of short introns were used as the neutral control. Only mutations present in the ISO-1 strain of *D*. *melanogaster* were used. The error bars indicate 95% confidence intervals of *α*_*asym*_. The exact values can be found in S1 Data. DGRP, *Drosophila* Genetic Reference panel; uAUG, start codon of upstream open reading frame.
(PDF)

S21 Fig The proportion of the newly fixed mutations in uAUGs in *D*. *melanogaster* that are under positive selection (*α*_*ori*_) for uORFs that have higher (red) and lower (blue) TE_uORF_ in each sample. The newly fixed and the polymorphic uORFs that are expressed in a sample (mRNA RPKM ≥ 1) were combined and equally split into two groups based on TE_uORF_. Paired *t* tests were performed to compare differences in *α*_*ori*_ between the higher versus lower TE groups across samples. The exact values can be found in S1 Data. RPKM, reads per kilobase of transcript per million mapped reads; TE, translational efficiency; uAUG, AUG start codon of uORF; uORF, upstream open reading frame.
(PDF)

S22 Fig The *α*_*asym*_ for mutations in uAUGs of Classes I, II, and III (combined) with AUGs in 3′ UTR as the neutral control for the GDL (blue) and DGRP (orange) data. Only mutations present in the ISO-1 strain of *D*. *melanogaster* were used. The error bars indicate 95% confidence intervals of *α*_*asym*_. The exact values can be found in S1 Data. DGRP, *Drosophila* Genetic Reference Panel; GDL, Global Diversity Lines; uAUG, start codon of upstream open reading frame; UTR, untranslated region.
(PDF)

S23 Fig The ratio of median TE for genes with single or multiple ribosome-associated uORFs relative to the median TE for genes without ribosome-associated uORFs in each sample (only genes with mRNA RPKM ≥ 1 were included in analysis). Wilcoxon rank-sum tests were performed to test the differences in each sample (*, *P* < 0.05; **, *P* < 0.01; ***, *P* < 0.001). Different cutoffs were used to define ribosome-associated uORFs as displayed above each plot. The raw data can be found in S4 Data. RPKM, reads per kilobase of transcript per million mapped reads; TE, translational efficiency; uORF, upstream open reading frame.
(PDF)

S24 Fig The correlation between the number of ribosome-associated uORFs and log_2_(TE) of main CDSs in each sample. Different cutoffs were employed to define ribosome-associated uORFs: (A) mRNA RPKM ≥ 1, TE > 0; (B) mRNA RPKM ≥ 1, TE ≥ 0.1; and (C) mRNA RPKM ≥ 1, TE ≥ 0.5. The raw data can be found in S4 Data. CDS, coding DNA sequence; RPKM, reads per kilobase of transcript per million mapped reads; TE, translational efficiency; uORF, upstream open reading frame.
(PDF)

S25 Fig The correlations between the number of ribosome-associated uORFs and log_2_(TE) of main CDSs in 0–2 h embryo data of this study and two previous studies. Different cutoffs were employed to define ribosome-associated uORFs: (A) mRNA RPKM ≥ 1, TE > 0; (B) mRNA RPKM ≥ 1, TE ≥ 0.1; (C) mRNA RPKM ≥ 1, TE ≥ 0.5. The “activated egg” is 0–2 h embryos studied by Kronja and colleagues [65]. The raw data can be found in S1 Data. CDS, coding DNA sequence; RPKM, reads per kilobase of transcript per million mapped reads; TE, translational efficiency; uORF, upstream open reading frame.
(PDF)

S26 Fig **The distribution of poly(A)-tail lengths of expressed genes (mRNA RPKM ≥ 1) without ribosome-associated uORFs (left) or with ribosome-associated uORFs (right) in 0–1 h embryos of *D*. *melanogaster*.** Differences in poly(A)-tail lengths were compared with *t* test. Data of poly(A)-tail lengths were from a previous study [118]. The raw data can be found in S1 Data. CDS, coding DNA sequence; RPKM, reads per kilobase of transcript per million mapped reads; TE, translational efficiency; uORF, upstream open reading frame.
(PDF)

S27 Fig Negative correlations between the lengths of 5′ UTRs and log_2_(TE) of main CDSs (in each sample, only genes with mRNA RPKM ≥ 1 were included in the analysis). Genes were grouped into 200 bins of equal size based on increasing length of 5′ UTRs. Median log_10_(5′ UTR length) and log_2_(TE) in each bin were displayed in the plots. A pseudo-count of 1 was added to 5′ UTR length before logarithmic transformation. The raw data can be found in S4 Data. CDS, coding DNA sequence; RPKM, reads per kilobase of transcript per million mapped reads; TE, translational efficiency; UTR, untranslated region.
(PDF)

S28 Fig The Spearman’s *rho* between the GC content of 5′ UTRs and log_2_(TE) of main CDSs (in each sample, only genes with mRNA RPKM ≥ 1 were included in the analysis). Genes were grouped into 200 bins of equal size based on increasing GC content of 5′ UTRs. The median GC content of 5′ UTR and log_2_(TE) in each bin were displayed in the plots. The raw data can be found in S4 Data. CDS, coding DNA sequence; RPKM, reads per kilobase of transcript per million mapped reads; TE, translational efficiency; UTR, untranslated region.
(PDF)

S29 Fig Positive correlations between Kozak scores of cAUGs and log_2_(TE) of main CDSs (in each sample, only genes with mRNA RPKM ≥ 1 were included in the analysis). Genes were grouped into 200 bins of equal size based on increasing Kozak score. Median Kozak score and log_2_(TE) in each bin were displayed in the plots. The raw data can be found in S4 Data. CDS, coding DNA sequence; RPKM, reads per kilobase of transcript per million mapped reads; TE, translational efficiency; UTR, untranslated region.
(PDF)

S30 Fig Positive correlations between MFE of secondary structure around cAUGs (the last 42 nt of 5′ UTRs) and log_2_(TE) of main CDSs (in each sample, only genes with mRNA RPKM ≥ 1 were included in the analysis). Genes were grouped into 200 bins of equal size based on increasing MFE. Median MFE and log_2_(TE) in each bin were displayed in the plots. The raw data can be found in S4 Data. cAUG, AUG start codon of CDS; CDS, coding DNA sequence; MFE, minimum free energy; RPKM, reads per kilobase of transcript per million mapped reads; TE, translational efficiency; UTR, untranslated region.
(PDF)

S31 Fig The relationship between MFE of secondary structure around 5′ cap (the first 42 nt of 5′ UTRs) and log_2_(TE) of main CDSs (in each sample, only genes with mRNA RPKM ≥ 1 were included in the analysis). Genes were grouped into 200 bins of equal size based on MFE. Median MFE and log_2_(TE) in each bin were displayed in the plots. The raw data can be found in S4 Data. CDS, coding DNA sequence; MFE, minimum free energy; RPKM, reads per kilobase of transcript per million mapped reads; TE, translational efficiency; UTR, untranslated region.
(PDF)

S32 Fig The differences in log_2_(TE) of main CDSs between genes with or without local stable hairpin structures (in each sample, only genes with mRNA RPKM ≥ 1 were included in the analysis). The raw data can be found in S4 Data. CDS, coding DNA sequence; RPKM, reads per kilobase of transcript per million mapped reads; TE, translational efficiency.
(PDF)

S33 Fig Negative correlations between Kozak score of uAUGs and log_2_(TE) of main CDSs for all expressed genes (mRNA RPKM ≥ 1) that contain a single ribosome-associated uORF. Genes were grouped into 50 bins of equal size based on increasing Kozak scores of uAUGs. Median Kozak score and log_2_(TE) in each bin were displayed in the plots. The raw data can be found in S1 Data. CDS, coding DNA sequence; RPKM, reads per kilobase of transcript per million mapped reads; TE, translational efficiency; uAUG, start codon of upstream open reading frame.
(PDF)

S34 Fig The relationship between uORF length and log_2_(TE) of main CDSs for all expressed genes (mRNA RPKM ≥ 1) that contain a single ribosome-associated uORF. Genes were grouped into 50 bins of equal size based on increasing uORF lengths. Median log_10_(uORF length) and log_2_(TE) in each bin were displayed in the plots. The raw data can be found in S1 Data. CDS, coding DNA sequence; RPKM, reads per kilobase of transcript per million mapped reads; TE, translational efficiency; uORF, upstream open reading frame.
(PDF)

S35 Fig The relationship between the distance from uAUG to 5′ cap (nt) and log_2_(TE) of main CDSs for the expressed genes (mRNA RPKM ≥ 1) that contain a single ribosome-associated uORF. Genes were grouped into 50 bins based on distance from uAUG to 5′ cap. Median log_10_(distance from uAUG to 5′ cap) and log_2_(TE) in each bin were displayed in the plots. The raw data can be found in S1 Data. CDS, coding DNA sequence; RPKM, reads per kilobase of transcript per million mapped reads; TE, translational efficiency; uAUG, start codon of upstream open reading frame.
(PDF)

S36 Fig The relationship between the distance from uORF stop codon to cAUG (nt) and log_2_(TE) of main CDSs for the expressed genes (mRNA RPKM ≥ 1) that contain a single ribosome-associated uORF. Genes were grouped into 50 bins based on distances from uORF stop codon to cAUG. Median distance from uORF stop codon to cAUG and log_2_(TE) in each bin were displayed in the plots. The raw data can be found in S1 Data. cAUG, AUG start codon of CDS; CDS, coding DNA sequence; RPKM, reads per kilobase of transcript per million mapped reads; TE, translational efficiency; uORF, upstream open reading frame.
(PDF)

S37 Fig Negative correlations between phyloP scores of uAUGs and log_2_(TE) of main CDSs for the expressed genes (mRNA RPKM ≥ 1) that contain a single ribosome-associated uORF. Genes were grouped into 50 bins based on increasing phyloP scores. Median phyloP score and log_2_(TE) in each bin were displayed in the plots. The raw data can be found in S1 Data. CDS, coding DNA sequence; RPKM, reads per kilobase of transcript per million mapped reads; TE, translational efficiency; uAUG, AUG start codon of uORF; uORF, upstream open reading frame.
(PDF)

S38 Fig Negative correlations between phyloCSF of uORFs and log_2_(TE) of main CDSs for all expressed genes (mRNA RPKM ≥ 1) that contain a single ribosome-associated uORF. Genes were grouped into 50 bins based on increasing phyloCSF. Median phyloCSF and log_2_(TE) in each bin were displayed in the plots. The raw data can be found in S1 Data. The raw data can be found in S1 Data. CDS, coding DNA sequence; RPKM, reads per kilobase of transcript per million mapped reads; TE, translational efficiency; uORF, upstream open reading frame.
(PDF)

S39 Fig The relationship between BLS of uORFs and log_2_(TE) of main CDSs for the expressed genes (mRNA RPKM ≥ 1) that contain a single ribosome-associated uORF. Genes were grouped into 50 bins based on increasing BLSs. Median uORF BLS and log_2_(TE) in each bin were displayed in the plots. The raw data can be found in S1 Data. BLS, branch length score; CDS, coding DNA sequence; RPKM, reads per kilobase of transcript per million mapped reads; TE, translational efficiency; uORF, upstream open reading frame.
(PDF)

S40 Fig The relationship between changes in TE_uORF_ and changes in TE_CDS_ for well-transcribed uORFs in genes that have the same dominant isoforms as supported by modENCODE CAGE and mRNA-Seq data in two neighboring samples. For each pair of samples, the Pearson’s correlation coefficient (*r*) and associated *P* value and the total number of well-transcribed uORFs (*n*) were shown. The blue dashed line is the linear fit of log_2_(TE_CDS,2_/TE_CDS,1_) against log_2_(TE_uORF,2_/TE_uORF,1_). The red dashed line denotes where log_2_(TE_CDS,2_/TE_CDS,1_) = log_2_(TE_uORF,2_/TE_uORF,1_). The raw data can be found in S1 Data. CAGE, cap analysis of gene expression; CDS, coding DNA sequence; TE, translational efficiency; uORF, upstream open reading frame.
(PDF)

S41 Fig The scheme for evaluating the statistical significance that the changes in TE_uORF_ inversely affect the magnitude of changes in TE_CDS_ between two samples. For a uORF and its downstream CDS in an mRNA that dominates in both sample 1 and 2, we defined *γ* = (TE_CDS,2_/TE_CDS,1_)/ (TE_uORF,2_/TE_uORF,1_) and tested whether log_2_(*γ*) is significantly different from 0. To estimate the SE of log_2_(*γ*), we modeled the log_2_(TE) of the uORF and the CDS in sample 1 or 2 with normal distributions. For female bodies and male bodies, we estimated the SE values of log_2_(TE) of a feature (CDS or uORF) by contrasting the RPF counts against the mRNA counts of biological replicates with DESeq2 [66]. For the remaining samples, the SE values were determined with the smooth surface of SE values of log_2_(TE) against the mRNA counts and log_2_(TE) of well-transcribed features in biological replicates of female and male bodies. The *P* value under the null hypothesis log_2_(*γ*) = 0 was obtained with a two-tailed Wald test. CDS, coding DNA sequence; RPF, ribosome-protected mRNA fragment; SE, standard error; TE, translational efficiency; uORF, upstream open reading frame.
(PDF)

S42 Fig The proportion of translated uORFs (TE_uORF_ ≥ 0.1) in genes with the different number of uORFs (mRNA RPKM ≥ 1) in each sample for 2-fold dominant transcripts that were constitutively expressed across all the 12 samples. Genes with at least 3 uORFs are grouped together for visualization. Spearman’s correlations between the number of uORFs in a gene and the proportion of translated uORFs are shown below the sample names. RPKM, reads per kilobase of transcript per million mapped reads; TE, translational efficiency; uORF, upstream open reading frame.
(PDF)

S43 Fig The proportion of translated uORFs (TE_uORF_ ≥ 0.1) in genes with the different number of uORFs (mRNA RPKM ≥ 1) for all uORF-containing genes in each sample. Genes with at least 3 uORFs are grouped together for visualization. Spearman’s correlations between the number of uORFs in a gene and the proportion of translated uORFs are shown below the sample names. RPKM, reads per kilobase of transcript per million mapped reads; TE, translational efficiency; uORF, upstream open reading frame.
(PDF)

S44 Fig **The dominant isoforms, the profiles of the mRNA-Seq (left) and Ribo-Seq (middle) data, and log**_**2**_**(TE) (right) of *D*. *melanogaster dichaete* in different developmental stages or tissues.** The CDS and UTR region in the gene model are in purple and orange, respectively. The CDS region of *dichaete* is also delineated with dashed lines. The short uORF-free isoform of *dichaete* (top) is predominately expressed in all samples except bodies of male adults, while a long isoform with many uORFs (dark green) is predominately expressed in bodies of male adults. Accordingly, log_2_(TE) of *dichaete* is much lower in bodies of male adults. The sequencing data are available from SRA under accession SRP067542 and rep1 and rep2 represent 2 biological replicates. CDS, coding DNA sequence; SRA, Sequence Read Archive; TE, translational efficiency; uORF, upstream open reading frame; UTR, untranslated region.
(PDF)

S45 Fig **The dominant isoforms, the profiles of the mRNA-Seq (left) and Ribo-Seq (middle) data, and log**_**2**_**(TE) (right) of *glycerol kinase 2* in different embryonic stages of *D*. *melanogaster*.** The CDS and UTR region in the gene model are in purple and orange, respectively. The CDS region of *glycerol kinase 2* is also delineated with dashed lines. In the fly embryo, an isoform containing 4 uORFs is predominately expressed during 0–12 h, while another isoform without uORFs predominates during 12–24 h, which might be related to the increased translation of *glycerol kinase 2* at this stage. The sequencing data are available from SRA under accession SRP067542. CDS, coding DNA sequence; TE, translational efficiency; uORF, upstream open reading frame; UTR, untranslated region.
(PDF)

S46 Fig The distribution of 5′ ends of CAGE tags around the TSSs of *genderblind* in different samples. For each sample, the corresponding CAGE samples of modENCODE were pooled together. The coverage at each site was calculated as the number of 5′ ends of CAGE tags at this site. The TSSs annotated by FlyBase and uAUGs were displayed with green arrows and red arrows, respectively. The CDS region was displayed in purple. CAGE, cap analysis of gene expression; CDS, coding DNA sequence; TSS, transcription start site; uAUG, start codon of upstream open reading frame.
(PDF)

S47 Fig The distribution of 5′ ends of CAGE tags around the TSSs of *dichaete* in different samples. For each sample, the corresponding CAGE samples of modENCODE were pooled together. The coverage at each site was calculated as the number of 5′ ends of CAGE tags at this site. The TSSs annotated by FlyBase and uAUGs were displayed with green arrows and red arrows, respectively. The CDS region was displayed in purple. CAGE, cap analysis of gene expression; CDS, coding DNA sequence; TSS, transcription start site; uAUG, start codon of upstream open reading frame.
(PDF)

S48 Fig The distribution of 5′ ends of CAGE tags around the TSS of *glycerol kinase 2* in different samples. For each sample, the corresponding CAGE samples of modENCODE were pooled together. The coverage at each site was calculated as the number of 5′ ends of CAGE tags at this site. The TSSs annotated by FlyBase and uAUGs were displayed with green arrows and red arrows, respectively. CAGE, cap analysis of gene expression; CDS, coding DNA sequence; TSS, transcription start site; uAUG, start codon of upstream open reading frame.
(PDF)

S49 Fig The distributions of log_2_(TE) for a feature with mRNA and RPF counts simulated under negative binomial distributions using different parameters. Under given expected mRNA count *μ*_*M*_ and RPF count *μ*_*R*_, the corresponding dispersion parameters were estimated with the overall dispersion trend of mRNA or RPF counts obtained with biological replicates of female and male bodies. Then, 10,000 mRNA counts and RPF counts were simulated with these parameters, and log_2_(TE) values were calculated with simulated counts. The observed distribution of log_2_(TE) was denoted with black line. The normal distribution fitted with maximum likelihood method was shown in red. RPF, ribosome-protected mRNA fragment; TE, translational efficiency.
(PDF)

S1 Data Numeric values underlying main figures and supplementary figures. (XLSX)

S2 Data Raw data used to generate Fig 3D and S14 Fig. (ZIP)

S3 Data Raw data used to generate Fig 4C and S17B Fig. (XLSX)

S4 Data Raw data used to generate Fig 5A, S23 Fig, S24 Fig and S27–S32 Fig. (XLSX)

S5 Data Raw data used to generate S2 Fig. (ZIP)

S6 Data Raw data used to generate S3 Fig. (ZIP)

S7 Data Raw data used to generate S8 Fig. (ZIP)

S8 Data Raw data used to generate S16 Fig. (ZIP)

## Abbreviations

BLS: branch length score
CAGE: cap analysis of gene expression
cAUG: AUG start codon of coding DNA sequence
CDKN2A: cyclin-dependent kinase inhibitor 2A
CDS: coding DNA sequence
CEG: constitutively expressed gene
CHX: cycloheximide
CRE: *cis*-regulatory element
CTSS: CAGE tag starting site
DENR: density-regulated protein
DFE: distribution of fitness effects
DGRP: *Drosophila* Genetic Reference Panel
DMSO: dimethyl sulfoxide
eIF2α: eukaryotic initiation factor 2 alpha
FDR: false discovery rate
gb: *genderblind*
GDL: Global Diversity Lines
GEO: Gene Expression Omnibus
MAF: minor allele frequency
MCT-1: multiple copies in T-cell lymphoma 1
MFE: minimum free energy
MK test: McDonald-Kreitman test
MNase: micrococcal nuclease
NCEG: nonconstitutively expressed gene
PIC: 43S preinitiation complex
PPM: position probability matrix
RPF: ribosome-protected mRNA fragment
RPKM: reads per kilobase of transcript per million mapped reads
RSCU: relative synonymous codon usage
SE: standard error
SFS: site frequency spectrum
SRA: Sequence Read Archive
SXL: sex lethal
TE: translational efficiency
uAUG: AUG start codon of upstream open reading frame
uORF: upstream open reading frame
UTR: untranslated region
WRST: Wilcoxon rank-sum test
