# Supplementary material for: Genome-wide maps of ribosomal occupancy provide insights into adaptive evolution and regulatory roles of uORFs during Drosophila development
Source: PLoS Biol. 2018 Jul 20;16(7):e2003903. doi: 10.1371/journal.pbio.2003903 (PMC6070289; doi:10.1371/journal.pbio.2003903)
Supplement: S1 Text — (PDF) [file pbio.2003903.s001.pdf]

**S1 Text. Supplementary Methods**

**High-throughput sequencing of mRNAs**

Poly(A)+ mRNA libraries for 0–2 h, 2–6 h, 6–12 h and 12–24 h old embryos, third-instar larvae, pupae, female heads, male heads, female bodies and male bodies were constructed using a procedure described previously [1]. Briefly, total RNA was extracted using the TRIzol reagent (Life Technologies, Inc.) and chloroform (Bejing Chemical Works) following the manufacturer's instructions. For each library, 10~20 μg poly(A)+ RNAs were selected on oligo-dT25 DynaBeads (Thermo Fisher) and fragmented at 70°C for 15min with RNA Fragmentation Reagents (Ambion). The fragmented RNAs within 40~80 nt were 3'-dephosphorylated with T4 Polynucleotide Kinase (NEB), ligated with 3'-adaptor and purified on a 15% TBE-Urea gel. The 3'-ligated RNAs were subsequently 5'-phosphorylated with T4 Polynucleotide Kinase and ATP (NEB), ligated with 5'-adaptor, and reverse transcribed into cDNAs with SuperScript™ III Reverse Transcriptase (Thermo Fisher) (see S16 Table for sequence of reverse transcriptional primer). The cDNA libraries of mRNAs were amplified through 14 PCR cycles (see S16 Table for sequences of PCR primers), and the products within the correct size ranges were collected in 20% TBE gels for quality tests (Fragment Analyzer, Agilent Technologies) and sequencing (Illumina HiSeq-2500 sequencer; run type: single-end; read length: 50 nt).

**Ribo-Seq of fly samples**

The Ribo-Seq experiments were conducted following a previous study [2] with modifications (see S3 Table for key differences). Briefly, the 0–2 h, 2–6 h, 6–12 h and 12–24 h old embryos, and the fine powder of third-instar larvae, pupae, heads, or bodies were homogenized in a dounce homogenizer with the lysis buffer [50 mM Tris pH 7.5, 150 mM NaCl, 5 mM $MgCl_2$, 1% Triton X-100, 2 mM DTT, 20 U/ml SuperaseIn (Ambion), 0.5 tablet of proteinase inhibitor (Roche), 100 μg/ml emetine (Sigma Aldrich), and 50 μM GMP-PNP (Sigma Aldrich)] at 4°C. Lysates were clarified by spinning at 4°C, 20,000×g for 8 min, and the supernatants were transferred to new 1.5 ml tubes. Then, 2 μl of each lysate was diluted 1:100 in DEPC-treated water, and OD260 of these dilutions were determined using NanoDrop (Thermo Fisher). For each sample, prepared lysates with ~400 μg total RNA were diluted 2:1 in digestion buffer (50 mM Tris pH 7.5, 15 mM $CaCl_2$, 5 mM $MgCl_2$, 1% Triton X-100, 2 mM DTT, 20 U/ml SuperaseIn, 0.5 tablet of proteinase inhibitor, 100 μg/ml emetine) and treated with micrococcal nuclease (NEB, 3 U/μg RNA in each sample) for 40 min at 25°C. Reactions were quenched by adding 0.1M EGTA to a final concentration of 6.25 mM and placed on ice. 10~45% sucrose gradients were prepared in the buffer (250 mM NaCl, 50 mM Tris pH 7.5, 15 mM $MgCl_2$, 0.5 mM DTT, 12 U/ml RNaseOUT, 0.5 tablet of protease inhibitor and 20 μg/ml emetine) using a Gradient Master (Biocomp Instruments) in ULTRA-CLEAR Thinwall Tubes (Beckman Coulter). Up to 300 μl of samples was applied to

the top of each gradient. After ultracentrifugation by a Hitachi P40ST rotor at 35,000 × rpm for 3 h at 4°C, the monosome fractions were collected, flash-frozen in liquid nitrogen, and stored at -80°C until further use. The polysome profiles of undigested lysates were footprinted as above.

SuperaseIn was applied throughout the protocol. To extract the ribosome protected fragments, monosome fractions were digested with 200 μg/ml Proteinase K (Roche) and 1% SDS (Sigma Aldrich) at 42°C for 30 min and RNAs were subsequently extracted once using acid phenol and once using phenol/chloroform. The 25~35-nt RNA fragment band was isolated from 15% TBE-Urea gels. To remove the rRNA-derived fragments, the RNAs were treated with Ribo-Zero Gold rRNA Removal Kit (Illumina). The remaining RNAs were sequentially subject to 3'-dephosphorylated, 3'-ligation, 5'-phosphorylation, 5'-ligation, and reverse-transcription. The cDNAs were treated with synthesized biotinylated oligos (S16 Table) on MyOne Streptavidin C1 DynaBeads (Invitrogen) for further depletion of the rRNA-derived fragments. The purified cDNAs were subjected to the same procedures described above for library generation.

Two biological replicates of mRNA-Seq and Ribo-Seq (independent sample preparation, library construction and sequencing under otherwise same conditions) were prepared for 1–10-day-old female bodies and male bodies.

**Ribo-Seq and harringtonine experiments of S2 cells**

*Drosophila* S2 cells were cultured in Schneider's Insect Medium (Sigma-Aldrich) containing 100 U/ml penicillin and 100 μg/ml streptomycin with 10% heat-inactivated fetal bovine serum. The cells were pre-treated with 2 μg/ml harringtonine (Sigma-Aldrich, dissolved in DMSO) or DMSO (as control) for 30 min. Then all cells were treated with 100 μg/ml CHX (Sigma-Aldrich) for 5 min, washed twice with cold PBS containing 100μg/ml CHX, and subsequently harvested. About 20% of the DMSO-treated cells were subjected to total RNA extraction and mRNA-Seq experiments as described above. And then the S2 cells were lysed in buffer with 50 mM Tris pH 7.5, 150 mM NaCl, 5 mM $MgCl_2$, 1% Triton X-100, 2 mM DTT, 20 U/ml SuperaseIn, 0.5 tablet of Protease inhibitor, 100μg/ml CHX. The resulting lysates were clarified by spinning 10 min at 1,300 × g at 4°C, and the supernatants were transferred to new tubes and placed on ice. After that, 2 μl of each lysate was diluted 1:100 in DEPC-treated water, and OD260 of these dilutions were determined using NanoDrop. The prepared lysates with ~400 μg total RNA were diluted 2:1 in a digestion buffer (50 mM Tris pH 7.5, 15 mM $CaCl_2$, 5 mM $MgCl_2$, 1% Triton X-100, 2 mM DTT, 20 U/ml SuperaseIn, 0.5 tablet of Protease inhibitor and 100μg/ml emetine) to bring the final concentration of NaCl to 100 mM and $CaCl_2$ to 5 mM, then treated with micrococcal nuclease (3 U/μg RNA) for 40 min at 25°C and were subjected to the same procedures described above.

**Deep-sequencing NGS data processing**

The 3' adaptors were trimmed using cutadapt 1.9 [3], and the NGS reads that were mapped to the genomes of yeast (S288C_R64.2.1), Wolbachia (GenBank accession NC_002987.6), or the sequences of tRNAs, rRNAs, snRNAs or snoRNAs of *D. melanogaster* (FlyBase r6.04) using bowtie 1.1.1 [4] with default parameters. The mapped reads on these genomes/sequences were further removed in the downstream analysis.

The poly(A)+ RNA-Seq reads and Ribo-Seq reads after filtering were mapped to the reference genome of *D. melanogaster* (FlyBase, r6.04) using STAR [5]. Reads mapped to multiple locations in genome were excluded for both mRNA-Seq and Ribo-Seq libraries. For Ribo-Seq libraries, only the reads with mapped length between 27 and 34 nt were used in further analysis. In each sample, we assigned a mapped RPF (27-34 nt in length) to its P-site, and calculated the RPKM values for a feature (CDS or uORF) with the mRNA or PPF data as previously described [2]. For uORFs that were overlapping with CDSs, only the non-overlapping regions of uORFs were used in calculating RPKM. The translational efficiency (TE) for a CDS or uORF was calculated as the ratio of RPF RPKM over mRNA RPKM [2, 6, 7]. For female bodies or male bodies, RPKM values from the two biological replicates were averaged before calculating TE. In each sample, the most abundant transcript in mRNA-Seq for each gene was inferred with kallisto [8].

**Gene ontology analysis**

Gene ontology analysis of genes without ribosome-associated uORFs or genes with ribosome-associated uORFs were performed with topGO [9]. All the 13,372 protein coding genes with annotated 5' UTRs were used as background gene list. When running topGO, the default algorithm "weight01" and test statistic "fisher" were used so that the resultant *P* values were calculated conditioned on other terms and not affected by multiple testing [9].

**Meta-gene profile analysis**

For each mRNA-Seq or Ribo-Seq library, profiles of reads across the most abundant isoforms of translated genes (mRNA-Seq RPKM > 10 and TE ≥ 0.1) were constructed by summing up the length-weighted coverage of reads at each nucleotide position along the transcripts. The coverage of a codon or triplet in a transcript was calculated as the total coverage of its three nucleotide positions. To build the meta-gene profile around start codons, we calculated the coverage of a 51-triplet window (including the start codon itself, 10 upstream triplets and 40 downstream codons) for each cAUG or a 16-triplet window (including the start codon itself, 5 upstream triplets and 10 downstream triplets) for each uAUG. For both CDS and uORFs in a transcript, the coverage at each triplet or codon was normalized by dividing the median coverage of all CDS codons in this transcript. The transcripts whose median coverage of CDS codons was zero were excluded in the analysis.

**Multiple regression analysis**

In order to assess the contribution of different features in 5' UTRs to TE of CDSs, we

performed multiple linear regression analysis with 'lm' function in R (https://www.r-project.org). The features include 5' UTR length, GC-content, Kozak score of cAUG, MFE of secondary structure near cAUG (last 42 nt of 5' UTR), MFE of secondary structure near 5' cap (first 42 nt of 5' UTR), numbers of stable hairpin structures in 5' UTR, and numbers of uORFs in the 5' UTR. MFE around start codon and 5' cap was calculated with RNAfold from Vienna RNA package [10]. In order to find local stable hairpin in 5' UTR, each 5' UTR sequence was scanned with a window size of 40 nt and a step size of 10 nt. For each window, RNAfold was employed with the parameter "-g" to search for G-quadruplexes while simultaneously calculating MFE. A local stable hairpin structure was defined as a window with at least one hairpin structure and MFE < -20 kcal/mol. AIC (Akaike Information Content) of the linear regression fit was extracted with function "extractAIC" and variable importance was calculated with "calc.relimp" from R Software package "relaimpo" [11] using "lmg" method ($r^2$ contribution averaged over orderings of predictors).

The relative contribution by the features of uORFs on CDS TEs were examined with similar approaches.

**Tissue specificity index $H_g$**

Tissue specificity index $H_g$ is the entropy of a gene's expression distribution [12]. Given the expression levels of a CDS or uORF in $n$ samples ($w_1$, $w_2$, …, $w_n$), the relative expression level of this CDS or uORF in sample $k$ would be $p_k = w_k / \sum_{i=1}^{n} w_i$. Then the tissue specificity for this CDS or uORF was calculated as $H_g = \sum_{k=1}^{n} -p_k \cdot \log_2(p_k)$.

## Supplementary References


1. Wang Y, Luo J, Zhang H, Lu J. microRNAs in the Same Clusters Evolve to Coordinately Regulate Functionally Related Genes. Molecular Biology and Evolution. 2016;33(9):2232-47.
2. Dunn JG, Foo CK, Belletier NG, Gavis ER, Weissman JS. Ribosome profiling reveals pervasive and regulated stop codon readthrough in Drosophila melanogaster. eLife. 2013;2:e01179.
3. Martin M. Cutadapt removes adapter sequences from high-throughput sequencing reads. EMBnetjournal. 2011;17(1):10-2.
4. Langmead B, Trapnell C, Pop M, Salzberg SL. Ultrafast and memory-efficient alignment of short DNA sequences to the human genome. Genome biology. 2009;10(3):R25.
5. Dobin A, Davis CA, Schlesinger F, Drenkow J, Zaleski C, Jha S, et al. STAR: ultrafast universal RNA-seq aligner. Bioinformatics. 2013;29(1):15-21.
6. Ingolia NT, Ghaemmaghami S, Newman JR, Weissman JS. Genome-wide analysis in vivo of translation with nucleotide resolution using ribosome profiling. Science. 2009;324(5924):218-23.

7. Tuller T, Carmi A, Vestsigian K, Navon S, Dorfan Y, Zaborske J, et al. An evolutionarily conserved mechanism for controlling the efficiency of protein translation. Cell. 2010;141(2):344-54.
8. Bray NL, Pimentel H, Melsted P, Pachter L. Near-optimal probabilistic RNA-seq quantification. Nature biotechnology. 2016;34(5):525-7. Epub 2016/04/05.
9. Alexa A, Rahnenfuhrer J. topGO: enrichment analysis for gene ontology. R package. 2016.
10. Lorenz R, Bernhart SH, Honer Zu Siederdissen C, Tafer H, Flamm C, Stadler PF, et al. ViennaRNA Package 2.0. Algorithms for molecular biology : AMB. 2011;6:26. Epub 2011/11/26.
11. Groemping U. Relative Importance for Linear Regression in R: The Package relaimpo. 2006. 2006;17(1):27. Epub 2006-09-01.
12. Schug J, Schuller W-P, Kappen C, Salbaum JM, Bucan M, Stoeckert CJ. Promoter features related to tissue specificity as measured by Shannon entropy. Genome Biology. 2005;6(4):R33.
