# Supplementary material for: Genome-wide maps of ribosomal occupancy provide insights into adaptive evolution and regulatory roles of uORFs during Drosophila development
Source: PLoS Biol. 2018 Jul 20;16(7):e2003903. doi: 10.1371/journal.pbio.2003903 (PMC6070289; doi:10.1371/journal.pbio.2003903)
Supplement: S2 Table — CAGE, cap analysis of gene expression; uORF, upstream open reading frame. (DOCX) [file pbio.2003903.s003.docx]

**S2 Table. The numbers of genes and uORFs expressed in 34 modENCODE samples that have both CAGE-Seq and mRNA-Seq data available.**

| Sample name | Accessions of CAGE data | Accession of mRNA-Seq data | Expressed genes | Expressed uORFs |
| --- | --- | --- | --- | --- |
| Kc167 cells | SRR488273, SRR488275, SRR488274, SRR488276 | SRR015084, SRR015086, SRR015088, SRR015090, SRR015092 | 7,819 | 16,033 |
| ML-DmBG3-c2 cells | SRR488307 | SRR015094, SRR015096, SRR015098, SRR015100, SRR015102 | 7,467 | 14,628 |
| S2-DRSC cells | SRR488286, SRR488287 | SRR015104, SRR015106, SRR015108, SRR015110, SRR015112 | 7,631 | 15,399 |
| Embryo 00-02hr | SRR488290, SRR488297 | SRR023199, SRR023659, SRR023663, SRR023671, SRR023747, SRR023755, SRR024013, SRR027110, SRR035417, SRR035418 | 7,261 | 13,479 |
| Embryo 02-04hr | SRR488291, SRR488298 | SRR023502, SRR023660, SRR023705, SRR023722, SRR023745, SRR027112 | 7,793 | 15,897 |
| Embryo 04-06hr | SRR488292 | SRR023539, SRR023669, SRR023696, SRR023746, SRR023836, SRR024014, SRR035220, SRR035405, SRR035406 | 7,989 | 17,172 |
| Embryo 06-08hr | SRR488300 | SRR023504, SRR023654, SRR023668, SRR023688, SRR023691, SRR023732, SRR024009, SRR024217, SRR027113, SRR035407, SRR035408 | 8,266 | 18,082 |
| Embryo 08-10hr | SRR488301 | SRR023549, SRR023596, SRR023603, SRR023657, SRR023701, SRR023749, SRR023750, SRR023754, SRR023759, SRR024219, SRR035409 | 8,658 | 19,072 |
| Embryo 12-14hr | SRR488302 | SRR023602, SRR023643, SRR023703, SRR023724, SRR023832, SRR024012, SRR035221, SRR035398, SRR035399 | 9,268 | 22,082 |
| Embryo 16-18hr | SRR488303 | SRR023600, SRR023707, SRR023715, SRR023720, SRR023751, SRR023826, SRR035402 | 9,866 | 24,006 |
| Embryo 20-22hr | SRR488304 | SRR023506, SRR023665, SRR023684, SRR023718, SRR023728, SRR027109 | 9,917 | 23,070 |
| Larva L1 | SRR488305 | SRR023597, SRR023646, SRR023661, SRR023666, SRR023706, SRR023835, SRR035410 | 9,857 | 21,966 |
| Larva L2 | SRR488293 | SRR023542, SRR023670, SRR023719, SRR023761, SRR023824, SRR024016, SRR035223, SRR035411, SRR035412 | 9,879 | 22,386 |
| Larva L3, 12hr old | SRR488306 | SRR023507, SRR023649, SRR023677, SRR023731, SRR023760, SRR027111 | 9,680 | 19,563 |
| Larva L3, central nervous system | SRR488299, SRR488321 | SRR384913, SRR384951 | 8,846 | 17,276 |
| Larva L3, puff stage 3–6 | SRR488294 | SRR023505, SRR023676, SRR023683, SRR023690, SRR023692, SRR023742, SRR027108 | 11,227 | 22,787 |
| Larva L3, wandering stage carcass | SRR488324 | SRR384949, SRR384950 | 9,236 | 16,429 |
| Larva L3, wandering stage digestive system | SRR488326 | SRR384912, SRR384948 | 8,724 | 11,940 |
| Larva L3, wandering stage imaginal discs | SRR488281, SRR488310, SRR488311, SRR488312, SRR488313 | SRR384911, SRR384945 | 10,943 | 19,041 |
| Pupae, 12hr after white-prepupae | SRR488295 | SRR023544, SRR023639, SRR023647, SRR023689, SRR023716, SRR023833, SRR035414 | 11,569 | 26,205 |
| Pupae, 2day after white-prepupae | SRR488296 | SRR023667, SRR023721, SRR023743, SRR023785, SRR023829, SRR026431 | 11,724 | 27,260 |
| 2day after white-prepupae, central nervous system | SRR488322 | SRR384955, SRR384956 | 9,025 | 19,991 |
| 2day after white-prepupae, fat body | SRR488327 | SRR384915, SRR384957 | 8,547 | 5,336 |
| Mated female, 1 day after eclosion, heads | SRR488271, SRR488277 | SRR384918, SRR384958 | 8,752 | 15,944 |
| Virgin female, 4 days after eclosion, ovaries | SRR488283 | SRR384926, SRR384927 | 7,527 | 13,926 |
| Mated female, 4 days eclosion, ovaries | SRR488282 | SRR384928, SRR384962 | 7,448 | 12,829 |
| Mated female, 20 days after eclosion, heads | SRR488279 | SRR384937, SRR384938 | 8,820 | 16,368 |
| Mated male, 1 day after eclosion, heads | SRR488272, SRR488278 | SRR384919, SRR384959 | 9,099 | 18,356 |
| Mated male, 4 days after eclosion, accessory glands | SRR488323 | SRR384907, SRR384931 | 10,399 | 14,078 |
| Mated male, 4days after eclosion, testes | SRR488284, SRR488285, SRR488308, SRR488309 | SRR384929, SRR384930 | 10,410 | 12,572 |
| Mated male, 20 days after eclosion, heads | SRR488280 | SRR384905, SRR384939 | 9,395 | 16,732 |
| Mixed adults, 4 days after eclosion, carcass | SRR488325 | SRR384908, SRR384934 | 9,511 | 15,400 |
| Mixed adults, 4 days after eclosion, digestive system | SRR488288 | SRR384932 | 8,993 | 11,782 |
| Mixed adults, 20 days after eclosion, digestive system | SRR488289 | SRR384940, SRR384941 | 8,873 | 13,035 |

We only counted genes with mRNA RPKM ≥ 1 in each sample. For an expressed uORF, we required both its host gene and the uORF itself to have mRNA RPKM ≥ 1. In case the CAGE-Seq data was available for a transcript, we also required the uORFs in that transcript to be downstream the 3' boundary of the major CAGE tag cluster for that transcript.
