# Supplementary material for: Genome-wide maps of ribosomal occupancy provide insights into adaptive evolution and regulatory roles of uORFs during Drosophila development
Source: PLoS Biol. 2018 Jul 20;16(7):e2003903. doi: 10.1371/journal.pbio.2003903 (PMC6070289; doi:10.1371/journal.pbio.2003903)
Supplement: S3 Table — (DOCX) [file pbio.2003903.s004.docx]

**S3 Table. Summary of treatment, inhibitors and ribonuclease used in mRNA-Seq and Ribo-Seq library constructions.**

| Sample | Library type | Pre-treatment | Inhibitor used in lysate preparation | Inhibitor and ribonuclease used in digestion | Library construction |
| --- | --- | --- | --- | --- | --- |
| S2 cells | mRNA-Seq | DMSO, 30min | - | - | no circularization |
| S2 cells | Ribo-Seq | DMSO, 30min | cycloheximide | emetine + MNase | no circularization |
| S2 cells | Ribo-Seq | harringtonine, 30min | cycloheximide | emetine + MNase | no circularization |
| 0-2h embryos | mRNA-Seq | none | - | - | no circularization |
| 2-6h embryos | mRNA-Seq | none | - | - | no circularization |
| 6-12h embryos | mRNA-Seq | none | - | - | no circularization |
| 12-24h embryos | mRNA-Seq | none | - | - | no circularization |
| Third-instar larvae | mRNA-Seq | none | - | - | no circularization |
| P7-8 pupae | mRNA-Seq | none | - | - | no circularization |
| Female adult heads | mRNA-Seq | none | - | - | no circularization |
| Male adult heads | mRNA-Seq | none | - | - | no circularization |
| Female adult bodies rep 1 | mRNA-Seq | none | - | - | no circularization |
| Female adult bodies rep 2 | mRNA-Seq | none | - | - | no circularization |
| Male adult bodies rep 1 | mRNA-Seq | none | - | - | no circularization |
| Male adult bodies rep 2 | mRNA-Seq | none | - | - | no circularization |
| 0-2h embryos | Ribo-Seq | none | emetine + GMP-PNP | emetine + MNase | no circularization |
| 2-6h embryos | Ribo-Seq | none | emetine + GMP-PNP | emetine + MNase | no circularization |
| 6-12h embryos | Ribo-Seq | none | emetine + GMP-PNP | emetine + MNase | no circularization |
| 12-24h embryos | Ribo-Seq | none | emetine + GMP-PNP | emetine + MNase | no circularization |
| Third-instar larvae | Ribo-Seq | none | emetine + GMP-PNP | emetine + MNase | no circularization |
| P7-8 pupae | Ribo-Seq | none | emetine + GMP-PNP | emetine + MNase | no circularization |
| Female adult heads | Ribo-Seq | none | emetine + GMP-PNP | emetine + MNase | no circularization |
| Male adult heads | Ribo-Seq | none | emetine + GMP-PNP | emetine + MNase | no circularization |
| Female adult bodies rep 1 | Ribo-Seq | none | emetine + GMP-PNP | emetine + MNase | no circularization |
| Female adult bodies rep 2 | Ribo-Seq | none | emetine + GMP-PNP | emetine + MNase | no circularization |
| Male adult bodies rep 1 | Ribo-Seq | none | emetine + GMP-PNP | emetine + MNase | no circularization |
| Male adult bodies rep 2 | Ribo-Seq | none | emetine + GMP-PNP | emetine + MNase | no circularization |
