# Supplementary material for: Genome-wide maps of ribosomal occupancy provide insights into adaptive evolution and regulatory roles of uORFs during Drosophila development
Source: PLoS Biol. 2018 Jul 20;16(7):e2003903. doi: 10.1371/journal.pbio.2003903 (PMC6070289; doi:10.1371/journal.pbio.2003903)
Supplement: S4 Table — (DOCX) [file pbio.2003903.s005.docx]

**S4 Table. Mapping statistics of mRNA-Seq and Ribo-Seq libraries.**

| Sample | Library type | Pre-  treatment | Total reads  (M) | After quality control (M)* | Unique mapping rate (%) | Multi mapping rate (%) | Library size  (M) | Mean coverage  (×) |
| --- | --- | --- | --- | --- | --- | --- | --- | --- |
| S2 cells | mRNA-Seq | DMSO,  30min | 30.8 | 21.7 | 90.5 | 2.3 | 19.2 | 9.0 |
| S2 cells | Ribo-Seq | DMSO,  30min | 33.4 | 14.3 | 65.2 | 6.6 | 7.9 | 2.5 |
| S2 cells | Ribo-Seq | harringtonine, 30min | 52.6 | 24.2 | 75.1 | 9.4 | 14.9 | 4.7 |
| 0-2h embryos | mRNA-Seq | none | 62.8 | 52.7 | 93.1 | 2.6 | 49.0 | 22.9 |
| 2-6h embryos | mRNA-Seq | none | 60.3 | 52.1 | 93.3 | 2.8 | 48.3 | 22.5 |
| 6-12h embryos | mRNA-Seq | none | 54.9 | 45.1 | 92.3 | 2.7 | 41.5 | 19.3 |
| 12-24h embryos | mRNA-Seq | none | 62.3 | 50.6 | 91.9 | 4.0 | 46.4 | 21.6 |
| Third-instar larvae | mRNA-Seq | none | 59.9 | 45.5 | 88.5 | 5.4 | 40.1 | 18.7 |
| P7-8 pupae | mRNA-Seq | none | 54.7 | 41.3 | 92.4 | 3.8 | 37.9 | 17.7 |
| Female adult heads | mRNA-Seq | none | 21.9 | 16.9 | 92.9 | 2.8 | 15.6 | 7.3 |
| Male adult heads | mRNA-Seq | none | 16.8 | 14.3 | 90.4 | 3.4 | 12.9 | 6.0 |
| Female adult bodies rep 1 | mRNA-Seq | none | 16.9 | 11.2 | 88.6 | 4.9 | 9.9 | 4.6 |
| Female adult bodies rep 2 | mRNA-Seq | none | 25.1 | 19.4 | 88.6 | 5.0 | 17.1 | 8.0 |
| Male adult bodies  rep 1 | mRNA-Seq | none | 20.0 | 16.0 | 91.5 | 5.8 | 14.6 | 6.8 |
| Male adult bodies  rep 2 | mRNA-Seq | none | 11.3 | 7.7 | 89.6 | 6.1 | 6.8 | 3.2 |
| 0-2h embryos | Ribo-Seq | none | 65.6 | 52.0 | 90.3 | 3.1 | 41.4 | 13.0 |
| 2-6h embryos | Ribo-Seq | none | 64.3 | 41.3 | 69.5 | 8.3 | 23.2 | 7.3 |
| 6-12h embryos | Ribo-Seq | none | 55.5 | 46.8 | 86.0 | 3.1 | 36.4 | 11.4 |
| 12-24h embryos | Ribo-Seq | none | 66.6 | 47.5 | 88.9 | 5.2 | 38.5 | 12.1 |
| Third-instar larvae | Ribo-Seq | none | 40.1 | 22.2 | 58.6 | 5.5 | 12.1 | 3.8 |
| P7-8 pupae | Ribo-Seq | none | 43.0 | 20.5 | 80.5 | 3.8 | 11.8 | 3.7 |
| Female adult heads | Ribo-Seq | none | 64.6 | 40.0 | 94.2 | 2.5 | 34.0 | 10.7 |
| Male adult heads | Ribo-Seq | none | 58.9 | 23.9 | 93.2 | 2.8 | 19.3 | 6.1 |
| Female adult bodies rep 1 | Ribo-Seq | none | 44.7 | 20.0 | 83.8 | 5.0 | 14.9 | 4.7 |
| Female adult bodies rep 2 | Ribo-Seq | none | 43.0 | 19.0 | 70.1 | 9.8 | 11.3 | 3.5 |
| Male adult bodies  rep 1 | Ribo-Seq | none | 45.6 | 19.1 | 79.1 | 7.4 | 13.1 | 4.1 |
| Male adult bodies  rep 2 | Ribo-Seq | none | 40.2 | 28.7 | 81.1 | 6.4 | 21.6 | 6.8 |

Library size for each library was calculated as total number of reads uniquely mapped to transcriptome. Mean coverage for each library was calculated as (library size × mean read length)/[total length of transcriptome (overlapping regions were merged strand specifically)]. Mean read length of 46 nt for mRNA-Seq libraries and 31 nt for Ribo-Seq libraries were used.

* number of remaining reads after removing reads mapped to rRNA, misc RNA (snoRNA, snRNA, rRNA, tRNA), yeast genome, Wolbachia genome.
