# Supplementary material for: Genome-wide maps of ribosomal occupancy provide insights into adaptive evolution and regulatory roles of uORFs during Drosophila development
Source: PLoS Biol. 2018 Jul 20;16(7):e2003903. doi: 10.1371/journal.pbio.2003903 (PMC6070289; doi:10.1371/journal.pbio.2003903)
Supplement: S5 Table — TE, translational efficiency; uORF, upstream open reading frame. (DOCX) [file pbio.2003903.s006.docx]

**S5 Table. Gene ontology analysis of genes without or with ribosome associated-uORFs (TE_uORF_ ≥ 0.5).**

| Ontology | GO ID | Term | Annotated genes | *P* value | |
| --- | --- | --- | --- | --- | --- |
| **Enriched terms in genes without ribosome-associated uORFs** | | | | |  |
| MF | GO:0008010 | Structural constituent of chitin-based larval cuticle | 105 | 1.0×10^-16^ | |
| MF | GO:0004252 | Serine-type endopeptidase activity | 211 | 6.7×10^-9^ | |
| MF | GO:0009055 | Electron carrier activity | 140 | 4.8×10^-8^ | |
| MF | GO:0003735 | Structural constituent of ribosome | 172 | 5.0×10^-6^ | |
| MF | GO:0005549 | Odorant binding | 64 | 1.2×10^-5^ | |
| MF | GO:0005506 | Iron ion binding | 148 | 1.6×10^-5^ | |
| MF | GO:0016705 | Oxidoreductase activity | 163 | 5.0×10^-5^ | |
| MF | GO:0004364 | Glutathione transferase activity | 39 | 3.3×10^-4^ | |
| MF | GO:0003899 | DNA-directed 5'-3' RNA polymerase activity | 29 | 0.002 | |
| MF | GO:0030234 | Enzyme regulator activity | 296 | 0.008 | |
| BP | GO:0006333 | Chromatin assembly or disassembly | 154 | 4.3×10^-16^ | |
| BP | GO:0032504 | Multicellular organism reproduction | 1,155 | 1.5×10^-13^ | |
| BP | GO:0040003 | Chitin-based cuticle development | 178 | 5.8×10^-13^ | |
| BP | GO:0006508 | Proteolysis | 732 | 2.6×10^-10^ | |
| BP | GO:0055114 | Oxidation-reduction process | 541 | 8.6×10^-9^ | |
| BP | GO:0006412 | Translation | 389 | 9.2×10^-5^ | |
| BP | GO:0006749 | Glutathione metabolic process | 46 | 5.5×10^-4^ | |
| BP | GO:0019236 | Response to pheromone | 21 | 0.001 | |
| BP | GO:0042254 | Ribosome biogenesis | 78 | 0.002 | |
| BP | GO:0045859 | Regulation of protein kinase activity | 76 | 0.003 | |
| **Enriched terms in genes with ribosome-associated uORFs** | | | | |  |
| MF | GO:0003700 | Transcription factor activity | 420 | 1.1×10^-15^ | |
| MF | GO:0005524 | ATP binding | 608 | 2.6×10^-14^ | |
| MF | GO:0004674 | Protein serine/threonine kinase activity | 184 | 1.7×10^-13^ | |
| MF | GO:0046872 | Metal ion binding | 1,337 | 1.9×10^-12^ | |
| MF | GO:0005096 | GTPase activator activity | 78 | 1.9×10^-11^ | |
| MF | GO:0004888 | Transmembrane signaling receptor activity | 258 | 3.8×10^-10^ | |
| MF | GO:0042803 | Protein homodimerization activity | 118 | 3.6×10^-8^ | |
| MF | GO:0003779 | Actin binding | 121 | 1.6×10^-7^ | |
| MF | GO:0005089 | Rho guanyl-nucleotide exchange factor activity | 28 | 6.0×10^-7^ | |
| MF | GO:0008092 | Cytoskeletal protein binding | 286 | 1.8×10^-5^ | |
| BP | GO:0007476 | Imaginal disc-derived wing morphogenesis | 350 | 9.3×10^-18^ | |
| BP | GO:0007411 | Axon guidance | 253 | 6.3×10^-16^ | |
| BP | GO:0007015 | Actin filament organization | 144 | 3.2×10^-10^ | |
| BP | GO:0006355 | Regulation of transcription, DNA-templated | 878 | 4.0×10^-10^ | |
| BP | GO:0055085 | Transmembrane transport | 442 | 5.8×10^-10^ | |
| BP | GO:0006468 | Protein phosphorylation | 394 | 9.4×10^-10^ | |
| BP | GO:0007616 | Long-term memory | 65 | 6.8×10^-9^ | |
| BP | GO:0043087 | Regulation of GTPase activity | 81 | 8.2×10^-9^ | |
| BP | GO:0008406 | Gonad development | 56 | 8.8×10^-9^ | |
| BP | GO:0007186 | G-protein coupled receptor signaling pathway | 207 | 2.0×10^-7^ | |

MF: molecular function; BP: biological process.
