# Supplementary material for: Genome-wide maps of ribosomal occupancy provide insights into adaptive evolution and regulatory roles of uORFs during Drosophila development
Source: PLoS Biol. 2018 Jul 20;16(7):e2003903. doi: 10.1371/journal.pbio.2003903 (PMC6070289; doi:10.1371/journal.pbio.2003903)
Supplement: S6 Table — CDS, coding DNA sequence; uORF, upstream open reading frame (DOCX) [file pbio.2003903.s007.docx]

**S6 Table. The numbers and proportions of expressed uORFs that are overlapping with other uORFs or CDSs.**

| Sample | Total number of expressed uORFs | Number of uORFs overlapping with other uORFs | Number of uORFs overlapping with CDSs | Number of uORFs overlapping with other uORFs or CDSs (%) |
| --- | --- | --- | --- | --- |
| Mature oocytes | 14,505 | 8,416 | 1,606 | 9,120 (62.9) |
| 0-2h embryos | 17,603 | 10,334 | 1,843 | 11,117 (63.2) |
| 2-6h embryos | 18,730 | 11,172 | 1,916 | 11,978 (64.0) |
| 6-12h embryos | 21,601 | 12,874 | 2,130 | 13,774 (63.8) |
| 12-24h embryos | 25,924 | 15,710 | 2,508 | 16,765 (64.7) |
| Larvae | 23,184 | 13,959 | 2,430 | 14,987 (64.6) |
| Pupae | 26,899 | 16,386 | 2,852 | 17,589 (65.4) |
| Female heads | 24,058 | 14,582 | 2,339 | 15,559 (64.7) |
| Male heads | 23,916 | 14,371 | 2,292 | 15,327 (64.1) |
| Female bodies | 22,455 | 13,422 | 2,286 | 14,398 (64.1) |
| Male bodies | 25,112 | 15,288 | 2,752 | 16,448 (65.5) |
| S2 cells(DMSO) | 17,112 | 10,164 | 1,798 | 10,914 (63.8) |
