# Supplementary material for: Genome-wide maps of ribosomal occupancy provide insights into adaptive evolution and regulatory roles of uORFs during Drosophila development
Source: PLoS Biol. 2018 Jul 20;16(7):e2003903. doi: 10.1371/journal.pbio.2003903 (PMC6070289; doi:10.1371/journal.pbio.2003903)
Supplement: S7 Table — TE, translational efficiency; uORF, upstream open reading frame. (DOCX) [file pbio.2003903.s008.docx]

**S7 Table. The proportions of non-overlapping and overlapping uORFs that are translated (TE_uORF_ ≥ 0.5).**

| Sample | Total Number of expressed uORFs | Non-overlapping uORFs | | Overlapping uORFs | | Longest uORFs of overlapping uORFs | |
| --- | --- | --- | --- | --- | --- | --- | --- |
|  |  | Total | Percentage of translated uORFs | Total | Percentage of translated uORFs | Total | Percentage of translated uORFs |
| Mature oocytes | 14,505 | 5,385 | 75.1% | 9,120 | 64.6% | 3,499 | 72.0% |
| 0-2h embryos | 17,603 | 6,486 | 54.7% | 11,117 | 58.3% | 4,177 | 54.7% |
| 2-6h embryos | 18,730 | 6,752 | 75.2% | 11,978 | 75.5% | 4,433 | 77.8% |
| 6-12h embryos | 21,601 | 7,827 | 49.2% | 13,774 | 57.2% | 5,073 | 52.1% |
| 12-24h embryos | 25,924 | 9,159 | 65.2% | 16,765 | 66.0% | 6,121 | 64.7% |
| Larvae | 23,184 | 8,197 | 66.1% | 14,987 | 59.9% | 5,444 | 65.1% |
| Pupae | 26,899 | 9,310 | 69.6% | 17,589 | 62.2% | 6,324 | 67.6% |
| Female heads | 24,058 | 8,499 | 52.9% | 15,559 | 55.5% | 5,718 | 52.5% |
| Male heads | 23,916 | 8,589 | 56.3% | 15,327 | 58.7% | 5,646 | 58.0% |
| Female bodies | 22,455 | 8,057 | 64.9% | 14,398 | 59.9% | 5,328 | 61.1% |
| Male bodies | 25,112 | 8,664 | 65.5% | 16,448 | 57.2% | 5,943 | 59.4% |
| S2 cells(DMSO) | 17,112 | 6,198 | 74.7% | 10,914 | 69.5% | 4,045 | 75.0% |
