# Supplementary material for: Genome-wide maps of ribosomal occupancy provide insights into adaptive evolution and regulatory roles of uORFs during Drosophila development
Source: PLoS Biol. 2018 Jul 20;16(7):e2003903. doi: 10.1371/journal.pbio.2003903 (PMC6070289; doi:10.1371/journal.pbio.2003903)
Supplement: S8 Table — (DOCX) [file pbio.2003903.s009.docx]

**S8 Table. The major transcripts in the mRNA-Seq data generated in this study and the cross-validation by the modENCODE mRNA-Seq data.**

| Sample | Number of expressed genes | Genes with two-fold dominant isoforms | Two-fold dominant isoforms that are also major isoforms in modENCODE data | Proportion |
| --- | --- | --- | --- | --- |
| Mature oocytes | 6,737 | 5,581 | - | - |
| 0-2h embryos | 7,897 | 6,307 | 5,739 | 91.0% |
| 2-6h embryos | 8,427 | 6,633 | 6,088 | 91.8% |
| 6-12h embryos | 8,994 | 6,949 | 6,361 | 91.5% |
| 12-24h embryos | 10,200 | 7,891 | 7,212 | 91.4% |
| Larvae | 11,089 | 8,801 | 7,885 | 89.6% |
| Pupae | 11,703 | 9,324 | 8,537 | 91.6% |
| Female heads | 9,542 | 7,351 | 6,369 | 86.6% |
| Male heads | 9,277 | 7,104 | 6,075 | 85.5% |
| Female bodies | 9,513 | 7,563 | 6,604 | 87.3% |
| Male bodies | 11,707 | 9,183 | 8,499 | 92.6% |
| S2 cells(DMSO) | 7,080 | 5,639 | 5,217 | 92.5% |

Major isoform: the most abundant transcript isoform for a gene. Two-fold dominant isoform: a major isoform that is the only isoform for a gene or expressed a least twice as much as the second most abundant isoform for a gene.
