# Supplementary material for: Genome-wide maps of ribosomal occupancy provide insights into adaptive evolution and regulatory roles of uORFs during Drosophila development
Source: PLoS Biol. 2018 Jul 20;16(7):e2003903. doi: 10.1371/journal.pbio.2003903 (PMC6070289; doi:10.1371/journal.pbio.2003903)
Supplement: S9 Table — uORF, upstream open reading frame. (DOCX) [file pbio.2003903.s010.docx]

**S9 Table. Summary of uORFs that are well transcribed but not translated in at least one sample.**

| Class | uORFs well transcribed in at least one sample | uORFs well transcribed with zero RPFs in at least one sample (%) | *P_m_*(*R_0_*) Fisher’s method | | | *P_m_*(*R_0_*) Pooling method | | |
| --- | --- | --- | --- | --- | --- | --- | --- | --- |
|  |  |  | *H_0_*(*c*)  (%*) | *H_0_*(*u*)  (%*) | *H_0_*(*0.1*)  (%*) | *H_0_*(*c*)  (%*) | *H_0_*(*u*)  (%*) | *H_0_*(*0.1*)  (%*) |
| I | 3,922 | 12 (0.3) | 7 (58.3) | 7 (58.3) | 0 (0) | 7 (58.3) | 7 (58.3) | 0 (0) |
| II | 8,401 | 286 (3.4) | 151 (52.8) | 85 (29.7) | 0 (0) | 153 (53.5) | 96 (33.6) | 3 (1.0) |
| III | 5,506 | 860 (15.6) | 503 (58.5) | 166 (19.3) | 7 (0.8) | 519 (60.3) | 193 (22.4) | 61 (7.1) |
| IV | 2,489 | 919 (36.9) | 491 (53.4) | 69 (7.5) | 16 (1.7) | 511 (55.6) | 84 (9.1) | 99 (10.8) |
| Total | 20,318 | 2,077 (10.2) | 1,152 (55.5) | 327 (15.7) | 23 (1.1) | 1,190 (57.3) | 380 (18.3) | 163 (7.8) |

*P_m_*(*R_0_*) is the probability of observing zero RPFs at a uORF under different null hypotheses: *H_0_*(*c*), that the uORF has the same TE as the downstream CDS; *H_0_*(*u*), that TE_uORF_ is equal to the average TE (*u*) of this uORF in least two other samples where it has ≥ 30 normalized mRNA reads and ≥ 3 normalized RPF reads; *H_0_*(*0.1*), that the uORF has a minimum TE of 0.1. For a uORF with zero RPFs but well transcribed (RPKM ≥ 1 and normalized read count ≥ 30 in mRNA-Seq) in multiple samples, *P_m_*(*R_0_*) values were aggregated with Fisher’s method or calculated after pooling the mRNA or RPF reads across those samples.

For each class, percentage of uORFs with zero RPFs among all uORFs well transcribed in at least one sample was shown in parenthesis.

* percentage of well translated uORFs that are truly not translated under different null hypothesis is calculated relative to all uORFs well expressed with zero RPFs in at least one sample in this class.

The number of significant uORFs were determined at an FDR of 0.05.
