# Supplementary material for: Genome-wide maps of ribosomal occupancy provide insights into adaptive evolution and regulatory roles of uORFs during Drosophila development
Source: PLoS Biol. 2018 Jul 20;16(7):e2003903. doi: 10.1371/journal.pbio.2003903 (PMC6070289; doi:10.1371/journal.pbio.2003903)
Supplement: S10 Table — cAUG, AUG start codon of coding DNA sequence. (DOCX) [file pbio.2003903.s011.docx]

**S10 Table. Position probability matrix for Kozak sequence context around the start codons of CDSs (cAUGs) in *D. melanogaster*.**

| Nucleotide | -6 | -5 | -4 | -3 | -2 | -1 | 3 |
| --- | --- | --- | --- | --- | --- | --- | --- |
| A | 0.320 | 0.259 | 0.306 | 0.642 | 0.488 | 0.431 | 0.266 |
| C | 0.190 | 0.263 | 0.396 | 0.060 | 0.257 | 0.265 | 0.172 |
| G | 0.246 | 0.209 | 0.148 | 0.247 | 0.118 | 0.192 | 0.335 |
| T | 0.243 | 0.268 | 0.150 | 0.051 | 0.137 | 0.112 | 0.227 |

The matrix is derived by counting the occurrence of A, C, G, T at positions from -6 to -1 and +3 relative to the first base of start codons of all protein coding genes with 5' UTR length ≥ 6 nt in *D. melanogaster* (for each gene the longest transcript isoform was used).
