# Supplementary material for: Genome-wide maps of ribosomal occupancy provide insights into adaptive evolution and regulatory roles of uORFs during Drosophila development
Source: PLoS Biol. 2018 Jul 20;16(7):e2003903. doi: 10.1371/journal.pbio.2003903 (PMC6070289; doi:10.1371/journal.pbio.2003903)
Supplement: S11 Table — MAF, minor allele frequency; uAUG, AUG start codon of uORF; uORF, upstream open reading frame. (DOCX) [file pbio.2003903.s012.docx]

**S11 Table. The number of mutations creating newly fixed uAUGs (K80 adjusted) or polymorphic uAUGs (minor allele frequency ≥ 0.05) and *α_ori_* for uORFs of different classes.**

| Class | Fixed | Polymorphic | Ratio | *α_ori_* |
| --- | --- | --- | --- | --- |
| I+II | 511 | 125 | 4.09 | 0.499 |
| III | 462 | 157 | 2.94 | 0.305 |
| IV | 344 | 130 | 2.57 | 0.204 |

For polymorphic AUGs, only mutations present in the ISO-1 strain of *D. melanogaster* were considered. The ratio of fixed to polymorphic AUG-creating mutations in 8-30 nt of short introns is 577/282 = 2.05.
