# Supplementary material for: Genome-wide maps of ribosomal occupancy provide insights into adaptive evolution and regulatory roles of uORFs during Drosophila development
Source: PLoS Biol. 2018 Jul 20;16(7):e2003903. doi: 10.1371/journal.pbio.2003903 (PMC6070289; doi:10.1371/journal.pbio.2003903)
Supplement: S12 Table — CDS, coding DNA sequence; TE, translational efficiency; UTR, untranslated region. (DOCX) [file pbio.2003903.s013.docx]

**S12 Table. Summary of multiple linear regression of log_2_(TE) of CDSs against features of 5' UTRs and the relative importance of these features.**

| Sample | Number of genes | *r^2^* | *P* value | Relative importance in multiple linear regression | | | | | | |
| --- | --- | --- | --- | --- | --- | --- | --- | --- | --- | --- |
|  |  |  |  | 5' UTR  length | GC  content | Kozak score of cAUG | MFE of secondary structure around cAUG | MFE of secondary structure around 5' cap | Stable  hairpins | uORF  number |
| Mature oocytes | 6,261 | 0.127 | 9.8×10^-179^ | 0.176 | 0.218 | 0.097 | 0.143 | 0.006 | 0.021 | 0.339 |
| 0-2h embryos | 7,346 | 0.073 | 5.5×10^-116^ | 0.122 | 0.528 | 0.058 | 0.163 | 0.067 | 0.021 | 0.041 |
| 2-6h embryos | 7,744 | 0.137 | 1.2×10^-242^ | 0.144 | 0.525 | 0.049 | 0.135 | 0.035 | 0.023 | 0.088 |
| 6-12h embryos | 8,217 | 0.104 | 1.4×10^-189^ | 0.092 | 0.346 | 0.111 | 0.173 | 0.011 | 0.035 | 0.233 |
| 12-24h embryos | 9,049 | 0.087 | 7.0×10^-173^ | 0.109 | 0.088 | 0.187 | 0.186 | 0.004 | 0.027 | 0.399 |
| Larvae | 9,542 | 0.037 | 5.8×10^-74^ | 0.033 | 0.100 | 0.440 | 0.142 | 0.213 | 0.025 | 0.048 |
| Pupae | 10,281 | 0.058 | 7.4×10^-128^ | 0.029 | 0.234 | 0.314 | 0.075 | 0.317 | 0.019 | 0.012 |
| Female heads | 8,547 | 0.172 | < 10^-307^ | 0.204 | 0.220 | 0.130 | 0.125 | 0.013 | 0.020 | 0.287 |
| Male heads | 8,373 | 0.128 | 7.4×10^-243^ | 0.239 | 0.123 | 0.173 | 0.127 | 0.006 | 0.016 | 0.315 |
| Female bodies | 8,419 | 0.118 | 1.6×10^-224^ | 0.104 | 0.044 | 0.180 | 0.204 | 0.003 | 0.013 | 0.451 |
| Male bodies | 10,160 | 0.074 | 9.5×10^-165^ | 0.062 | 0.009 | 0.291 | 0.174 | 0.135 | 0.017 | 0.312 |
| S2 cells (DMSO) | 6,599 | 0.230 | < ×10^-307^ | 0.234 | 0.361 | 0.036 | 0.095 | 0.013 | 0.017 | 0.243 |

Only expressed genes (mRNA RPKM ≥ 1) with annotated 5' UTR and ribosome-associated uORFs (uORF mRNA RPKM ≥ 1 and TE ≥ 0.5) in each sample were used in analysis.
