# Supplementary material for: Genome-wide maps of ribosomal occupancy provide insights into adaptive evolution and regulatory roles of uORFs during Drosophila development
Source: PLoS Biol. 2018 Jul 20;16(7):e2003903. doi: 10.1371/journal.pbio.2003903 (PMC6070289; doi:10.1371/journal.pbio.2003903)
Supplement: S13 Table — CDS, coding DNA sequence; TE, translational efficiency; uORF, upstream open reading frame. (DOCX) [file pbio.2003903.s014.docx]

**S13 Table. Summary of multiple linear regression of log_2_(TE) of CDSs against features of uORFs in each sample.**

| Sample | Number of genes | *r*^2^ | *P* value | Relative importance in multiple linear regression | | | | | | |
| --- | --- | --- | --- | --- | --- | --- | --- | --- | --- | --- |
|  |  |  |  | Kozak score of uAUG | Distance from uAUG to 5' cap | PhyloP of uAUG | PhyloCSF of uORF | Distance from uAUG to cAUG | BLS of uORF | uORF length |
| Mature oocytes | 1,243 | 0.066 | 2.2×10^-15^ | 0.436 | 0.030 | 0.263 | 0.095 | 0.025 | 0.119 | 0.032 |
| 0-2h embryos | 1,354 | 0.047 | 1.8×10^-11^ | 0.329 | 0.322 | 0.214 | 0.009 | 0.059 | 0.029 | 0.037 |
| 2-6h embryos | 1,405 | 0.100 | 1.8×10^-28^ | 0.194 | 0.307 | 0.305 | 0.007 | 0.036 | 0.128 | 0.025 |
| 6-12h embryos | 1,474 | 0.049 | 2.5×10^-13^ | 0.581 | 0.129 | 0.202 | 0.047 | 0.013 | 0.023 | 0.006 |
| 12-24h embryos | 1,605 | 0.062 | 4.2×10^-19^ | 0.428 | 0.028 | 0.095 | 0.015 | 0.177 | 0.085 | 0.172 |
| Larvae | 1,506 | 0.032 | 2.5×10^-8^ | 0.853 | 0.028 | 0.095 | 0.007 | 0.004 | 0.012 | 0.002 |
| Pupae | 1,767 | 0.038 | 2.7×10^-12^ | 0.478 | 0.033 | 0.010 | 0.098 | 0.190 | 0.060 | 0.131 |
| Female heads | 1,471 | 0.090 | 8.7×10^-27^ | 0.395 | 0.219 | 0.280 | 0.013 | 0.008 | 0.076 | 0.009 |
| Male heads | 1,384 | 0.089 | 1.4×10^-24^ | 0.486 | 0.264 | 0.202 | 0.012 | 0.006 | 0.017 | 0.013 |
| Female bodies | 1,593 | 0.060 | 3.3×10^-18^ | 0.393 | 0.085 | 0.067 | 0.032 | 0.147 | 0.102 | 0.174 |
| Male bodies | 1,896 | 0.049 | 1.5×10^-17^ | 0.659 | 0.040 | 0.012 | 0.043 | 0.088 | 0.077 | 0.081 |
| S2 cells(DMSO) | 1,316 | 0.125 | 2.7×10^-34^ | 0.249 | 0.402 | 0.218 | 0.006 | 0.051 | 0.045 | 0.029 |

Only expressed genes (mRNA RPKM ≥ 1) with single ribosome-associated uORFs (uORF mRNA RPKM ≥ 1 and TE ≥ 0.5) in each sample were used in analysis.
