# Supplementary material for: Genome-wide maps of ribosomal occupancy provide insights into adaptive evolution and regulatory roles of uORFs during Drosophila development
Source: PLoS Biol. 2018 Jul 20;16(7):e2003903. doi: 10.1371/journal.pbio.2003903 (PMC6070289; doi:10.1371/journal.pbio.2003903)
Supplement: S14 Table — CDS, coding DNA sequence; TE, translational efficiency; uORF, upstream open reading frame. (DOCX) [file pbio.2003903.s015.docx]

**S14 Table. Summary of genes whose TE_uORF_ (pooled) changed disproportionally relative to the downstream CDSs between two neighboring developmental stages.**

| Sample 1 | Sample 2 | Total  genes | *β_u_* ≠1  (%) | *β_u_* > 1 | | | *β_u_* < 1 | | | *χ^2^* test  *P* value |
| --- | --- | --- | --- | --- | --- | --- | --- | --- | --- | --- |
|  |  |  |  | Total  (%*) | *γ* > 1  (%**) | *γ* < 1  (%**) | Total  (%*) | *γ* > 1  (%**) | *γ* < 1  (%**) |  |
| 0-2h embryos | 2-6h embryos | 1,460 | 646  (44.2) | 531  (82.2) | 1  (0.2) | 275  (51.8) | 115  (17.8) | 7  (6.1) | 13  (11.3) | 1.0×10^-54^ |
| 2-6h embryos | 6-12h embryos | 1,420 | 997  (70.2) | 17  (1.7) | 2  (11.8) | 4  (23.5) | 980  (98.3) | 812  (82.9) | 0  (0) | 3.6×10^-178^ |
| 6-12h embryos | 12-24h embryos | 1,458 | 306  (21.0) | 282  (92.2) | 1  (0.4) | 153  (54.3) | 24  (7.8) | 4  (16.7) | 1  (4.2) | 1.0×10^-34^ |
| 12-24h embryos | Larvae | 909 | 415  (45.7) | 396  (95.4) | 0  (0) | 340  (85.9) | 19  (4.6) | 7  (36.8) | 0  (0) | 1.9×10^-77^ |
| Larvae | Pupae | 774 | 74  (10.8) | 47  (63.5) | 0  (0) | 1  (2.1) | 27  (36.5) | 7  (25.9) | 0  (0) | 4.7×10^-3^ |
| Pupae | Female heads | 668 | 381  (57.0) | 5  (1.3) | 0  (0) | 1  (20.0) | 376  (98.7) | 239  (63.6) | 0  (0) | 3.9×10^-54^ |
| Pupae | Male heads | 597 | 259  (43.4) | 15  (5.8) | 1  (6.7) | 2  (13.3) | 244  (95.2) | 122  (50.0) | 0  (0) | 3.8×10^-28^ |

For each gene with the same two-fold dominant isoform as supported by modENCODE CAGE and mRNA-Seq data in both samples, we pooled the mRNA or RPF reads of uORFs in each sample. Only genes with pooled uORF RPKM ≥ 1 and pooled uORF reads ≥ 30 in mRNA-Seq were considered in the analysis.

*β_u_* = TE_uORF,2_/TE_uORF,1_ is the fold change of pooled TE_uORF_ in sample 2 relative to sample 1. Sample pairs with less than 70 genes that have *β_u_* ≠ 1 were not considered.

*γ* = (TE_CDS,2_/TE_CDS,1_)/ (TE_uORF,2_/TE_uORF,1_).

* Percentage of uORFs with *β_u_* > 1 or *β_u_* < 1 among all total uORFs in each pair of samples.

** Percentage of uORFs with *γ* > 1 or *γ* < 1 among all uORFs with *β_u_* > 1 or *β_u_* < 1.

Significance was determined at an FDR of 0.05.

*χ^2^* tests were performed to compare the differences in the number of genes with *β_u_* > 1 and *γ* < 1or *β_u_* < 1 and *γ* > 1 and the number of uORFs with *β_u_* > 1 and *γ* > 1or *β_u_* < 1 and *γ* < 1.
