# Supplementary material for: Genome-wide maps of ribosomal occupancy provide insights into adaptive evolution and regulatory roles of uORFs during Drosophila development
Source: PLoS Biol. 2018 Jul 20;16(7):e2003903. doi: 10.1371/journal.pbio.2003903 (PMC6070289; doi:10.1371/journal.pbio.2003903)
Supplement: S15 Table — (DOCX) [file pbio.2003903.s016.docx]

**S15 Table. The size factor for mRNA-Seq or Ribo-Seq library and raw reads required to reach a normalized mRNA read count of 30 in each sample.**

| Sample | mRNA-Seq  size factor | Raw mRNA reads equal to 30 normalized reads | Ribo-Seq  size factor |
| --- | --- | --- | --- |
| Mature oocytes | 2.23 | 67 | 0.45 |
| 0-2h embryos | 1.20 | 36 | 0.83 |
| 2-6h embryos | 1.36 | 41 | 0.74 |
| 6-12h embryos | 0.98 | 30 | 1.02 |
| 12-24h embryos | 1.02 | 31 | 0.98 |
| Larvae | 1.84 | 56 | 0.54 |
| Pupae | 1.78 | 54 | 0.56 |
| Female heads | 0.64 | 20 | 1.55 |
| Male heads | 0.78 | 24 | 1.29 |
| Female bodies rep 1 | 0.79 | 24 | 1.26 |
| Female bodies rep 2 | 1.19 | 36 | 0.84 |
| Male bodies rep 1 | 1.14 | 35 | 0.88 |
| Male bodies rep 2 | 0.63 | 19 | 1.60 |
| S2 cells (DMSO) | 1.44 | 44 | 0.70 |
