# Supplementary material for: Genome-wide maps of ribosomal occupancy provide insights into adaptive evolution and regulatory roles of uORFs during Drosophila development
Source: PLoS Biol. 2018 Jul 20;16(7):e2003903. doi: 10.1371/journal.pbio.2003903 (PMC6070289; doi:10.1371/journal.pbio.2003903)
Supplement: S16 Table — (DOCX) [file pbio.2003903.s017.docx]

**S16 Table. Oligos used in this study.**

| Oligo | Purpose | Sequence |
| --- | --- | --- |
| RA3 | 3' adaptor for RNA fragments | /5phos/tggaattctcgggtgccaagg/3ddc |
| RA5 | 5' adaptor for RNA fragments | guucagaguucuacaguccgacgauc |
| RTP | Primer for reverse transcription | gccttggcacccgagaattcca |
| oJGD132 | Subtractive hybridization of rRNA-derived fragments for Ribo-Seq library construction | /5biosg/cattgtaatctattagcatataccaaattt |
| oJGD133 | Subtractive hybridization of rRNA-derived fragments for Ribo-Seq library construction | /5biosg/tgataaagtgctgatagatttatatgatta |
| oJGD134 | Subtractive hybridization of rRNA-derived fragments for Ribo-Seq library construction | /5biosg/gctaattaacacaatcccg/ideoxyi//ideoxyi/gcgttctat |
| oJGD135 | Subtractive hybridization of rRNA-derived fragments for Ribo-Seq library construction | /5biosg/acgacaatggatgtgatgccaatgtaattt |
| oJGD136 | Subtractive hybridization of rRNA-derived fragments for Ribo-Seq library construction | /5biosg/ggttgaactctagataacatgcagatcgta |
| oJGD161 | Subtractive hybridization of rRNA-derived fragments for Ribo-Seq library construction | /5biosg/tttgatgcaagcttcttgatcaaagtatcacgagt |
| oJGD162 | Subtractive hybridization of rRNA-derived fragments for Ribo-Seq library construction | /5biosg/tgcattgtatggcttctaaaccatttaaagtttat |
| oJGD163 | Subtractive hybridization of rRNA-derived fragments for Ribo-Seq library construction | /5biosg/cttggactacatatggttgagggttg |
| oJGD164 | Subtractive hybridization of rRNA-derived fragments for Ribo-Seq library construction | /5biosg/ttggactacatatggttgaggg |
| L1 | Subtractive hybridization of rRNA-derived fragments for Ribo-Seq library construction | /5biosg/attaggctaaaaccaagcgatcgcaagatcg |
| L2 | Subtractive hybridization of rRNA-derived fragments for Ribo-Seq library construction | /5biosg/acaacctcaactcatatgggactaccccctga |
| L3 | Subtractive hybridization of rRNA-derived fragments for Ribo-Seq library construction | /5biosg/acgatgccagctagcaattgggtgtagc |
| L4 | Subtractive hybridization of rRNA-derived fragments for Ribo-Seq library construction | /5biosg/ttgccaaagatgttttcattaatcaagaacga |
| L5 | Subtractive hybridization of rRNA-derived fragments for Ribo-Seq library construction | /5biosg/ataactgtggtaattctagagctaatacat |
| L6 | Subtractive hybridization of rRNA-derived fragments for Ribo-Seq library construction | /5biosg/aactctaagcggtggatcactcggctcatggg |
| L7 | Subtractive hybridization of rRNA-derived fragments for Ribo-Seq library construction | /5biosg/aggtcgtatccgtgctggactgcaatgataa |
| L8 | Subtractive hybridization of rRNA-derived fragments for Ribo-Seq library construction | /5biosg/cattgtgatggccctagcgggtgttgacacaa |
| L9 | Subtractive hybridization of rRNA-derived fragments for Ribo-Seq library construction | /5biosg/tgtgcttttattaggctaaaaccaagcg |
| Dro-01 | Subtractive hybridization of rRNA-derived fragments for Ribo-Seq library construction | /5biosg/atgtatgtaagcgtattaccggtgg |
| Dro-02 | Subtractive hybridization of rRNA-derived fragments for Ribo-Seq library construction | /5biosg/gcttggactacatatggttgagggttgta |
| Dro-03 | Subtractive hybridization of rRNA-derived fragments for Ribo-Seq library construction | /5biosg/atgtaagtggagccgtacctgttggtttgtccc |
| Dro-04 | Subtractive hybridization of rRNA-derived fragments for Ribo-Seq library construction | /5biosg/agttacgtagccaattgtggaactttcttgct |
| RP1 | Amplification of libraries before sequencing. Pair with any of RPI1-16 | aatgatacggcgaccaccgagatctacacgttcagagttctacagtccga |
| RPI1 | Amplification of libraries before sequencing. Contains barcode CGTGAT | caagcagaagacggcatacgagatcgtgatgtgactggagttccttggcacccgagaattcca |
| RPI2 | Amplification of libraries before sequencing. Contains barcode ACATCG | caagcagaagacggcatacgagatacatcggtgactggagttccttggcacccgagaattcca |
| RPI3 | Amplification of libraries before sequencing. Contains barcode GCCTAA | caagcagaagacggcatacgagatgcctaagtgactggagttccttggcacccgagaattcca |
| RPI4 | Amplification of libraries before sequencing. Contains barcode TGGTCA | caagcagaagacggcatacgagattggtcagtgactggagttccttggcacccgagaattcca |
| RPI5 | Amplification of libraries before sequencing. Contains barcode CACTGT | caagcagaagacggcatacgagatcactgtgtgactggagttccttggcacccgagaattcca |
| RPI6 | Amplification of libraries before sequencing. Contains barcode ATTGGC | caagcagaagacggcatacgagatattggcgtgactggagttccttggcacccgagaattcca |
| RPI7 | Amplification of libraries before sequencing. Contains barcode GATCTG | caagcagaagacggcatacgagatgatctggtgactggagttccttggcacccgagaattcca |
| RPI8 | Amplification of libraries before sequencing. Contains barcode TCAAGT | caagcagaagacggcatacgagattcaagtgtgactggagttccttggcacccgagaattcca |
| RPI9 | Amplification of libraries before sequencing. Contains barcode CTGATC | caagcagaagacggcatacgagatctgatcgtgactggagttccttggcacccgagaattcca |
| RPI10 | Amplification of libraries before sequencing. Contains barcode AAGCTA | caagcagaagacggcatacgagataagctagtgactggagttccttggcacccgagaattcca |
| RPI11 | Amplification of libraries before sequencing. Contains barcode GTAGCC | caagcagaagacggcatacgagatgtagccgtgactggagttccttggcacccgagaattcca |
| RPI12 | Amplification of libraries before sequencing. Contains barcode TACAAG | caagcagaagacggcatacgagattacaaggtgactggagttccttggcacccgagaattcca |
| RPI13 | Amplification of libraries before sequencing. Contains barcode TTGACT | caagcagaagacggcatacgagatttgactgtgactggagttccttggcacccgagaattcca |
| RPI14 | Amplification of libraries before sequencing. Contains barcode GGAACT | caagcagaagacggcatacgagatggaactgtgactggagttccttggcacccgagaattcca |
| RPI15 | Amplification of libraries before sequencing. Contains barcode TGACAT | caagcagaagacggcatacgagattgacatgtgactggagttccttggcacccgagaattcca |
| RPI16 | Amplification of libraries before sequencing. Contains barcode GGACGG | caagcagaagacggcatacgagatggacgggtgactggagttccttggcacccgagaattcca |

The following codes denotes different types of modifications on base: /5phos/, 5' phospohate; /3ddc, 3' dideoxycytidine; /ideoxyi/, internal inosine residue; /5biosg/, 5' biotin.

Oligo oJGD132-136 and oJGD161-164 were based on a previous study [1].

**References:**

1. Dunn JG, Foo CK, Belletier NG, Gavis ER, Weissman JS. Ribosome profiling reveals pervasive and regulated stop codon readthrough in Drosophila melanogaster. eLife. 2013;2:e01179.
