# Supplementary figures and images for: Genome-wide maps of ribosomal occupancy provide insights into adaptive evolution and regulatory roles of uORFs during Drosophila development
Source: PLoS Biol. 2018 Jul 20;16(7):e2003903. doi: 10.1371/journal.pbio.2003903 (PMC6070289; doi:10.1371/journal.pbio.2003903)

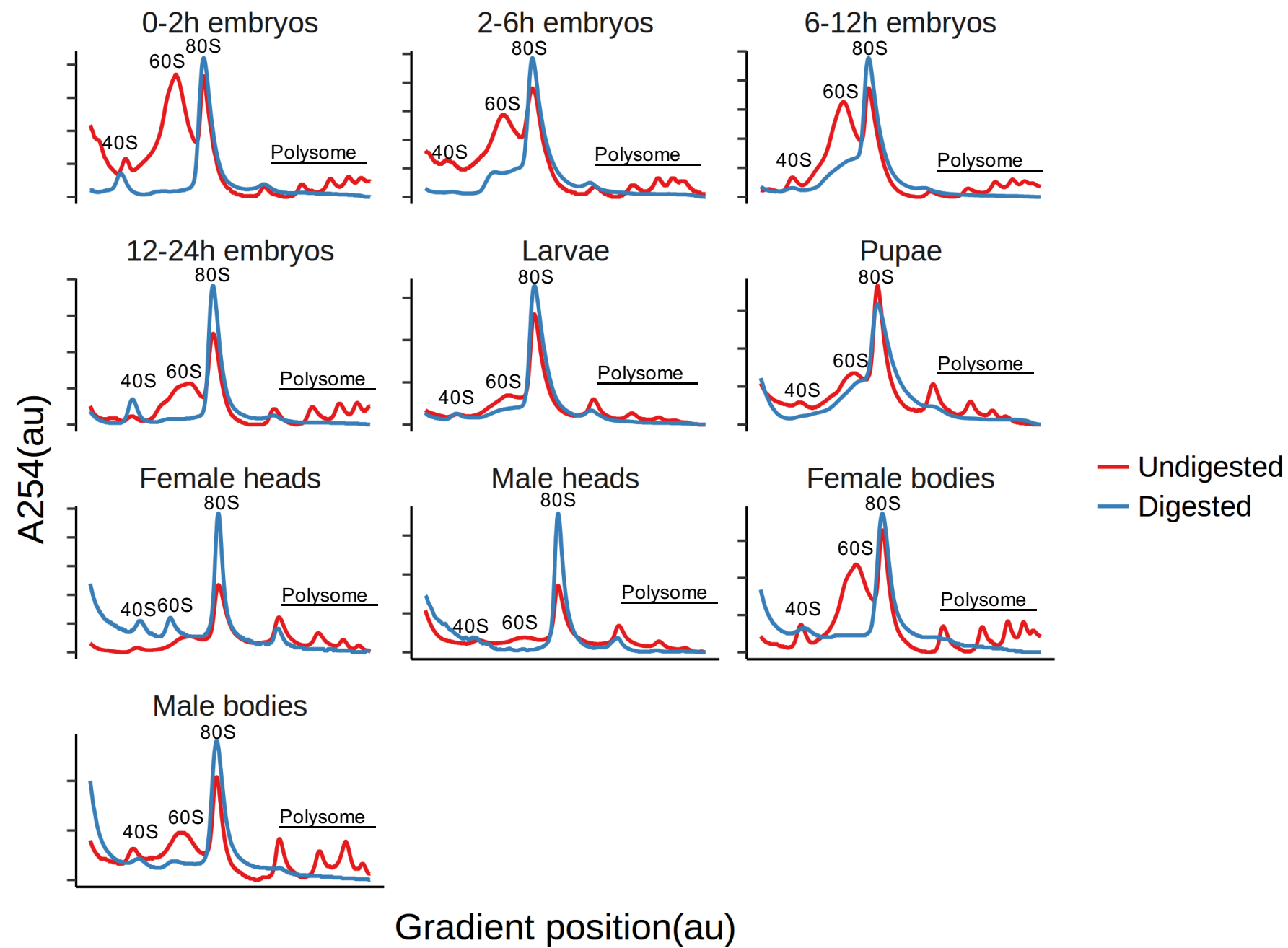

Supplement: S1 Fig — au, arbitrary unit; MNase, micrococcal nuclease. (PDF) [file pbio.2003903.s018.pdf]

A

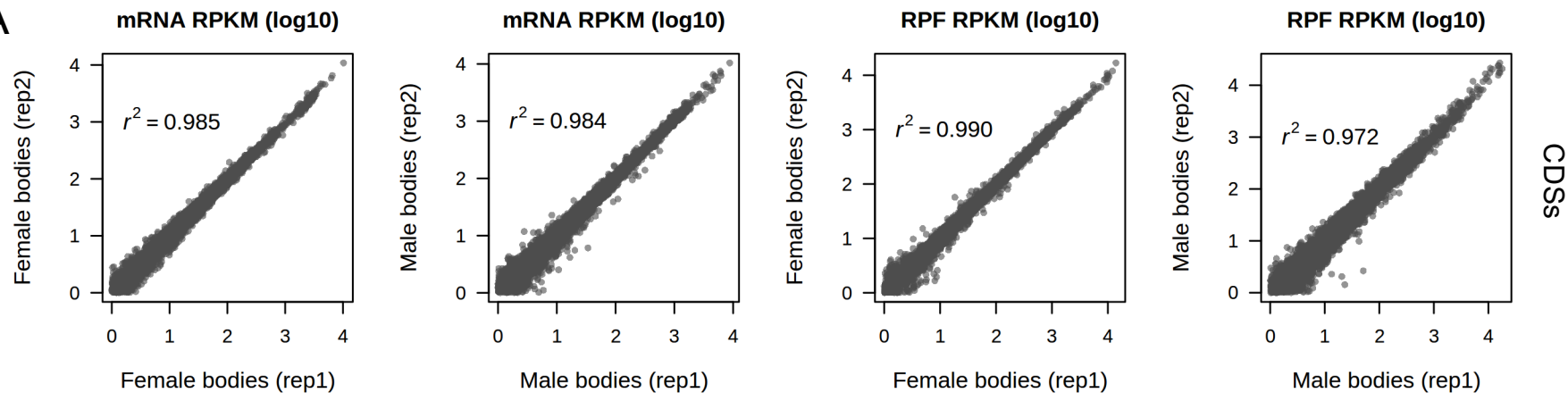

B

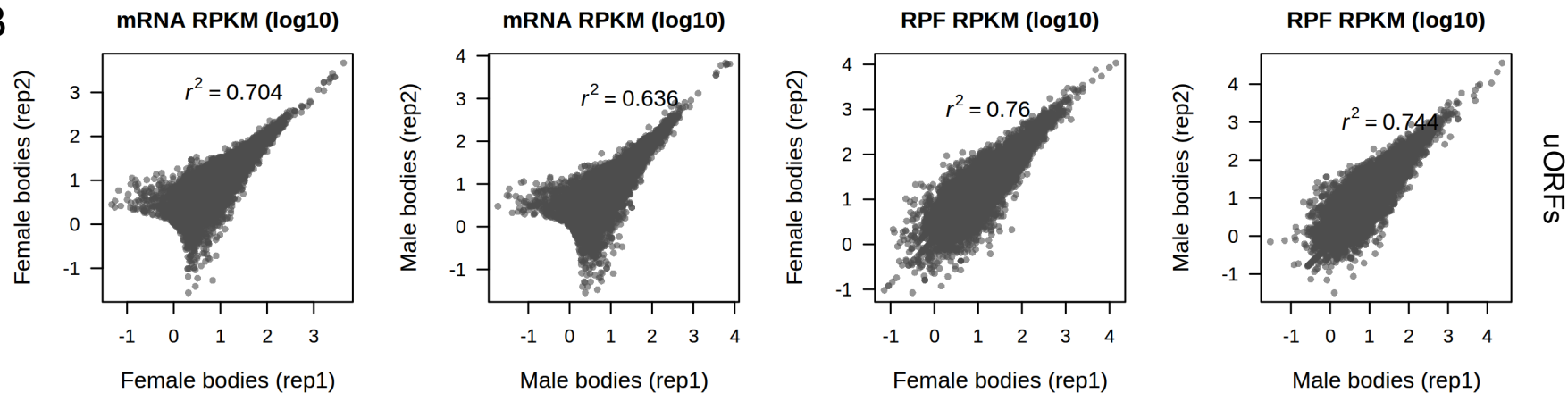

C

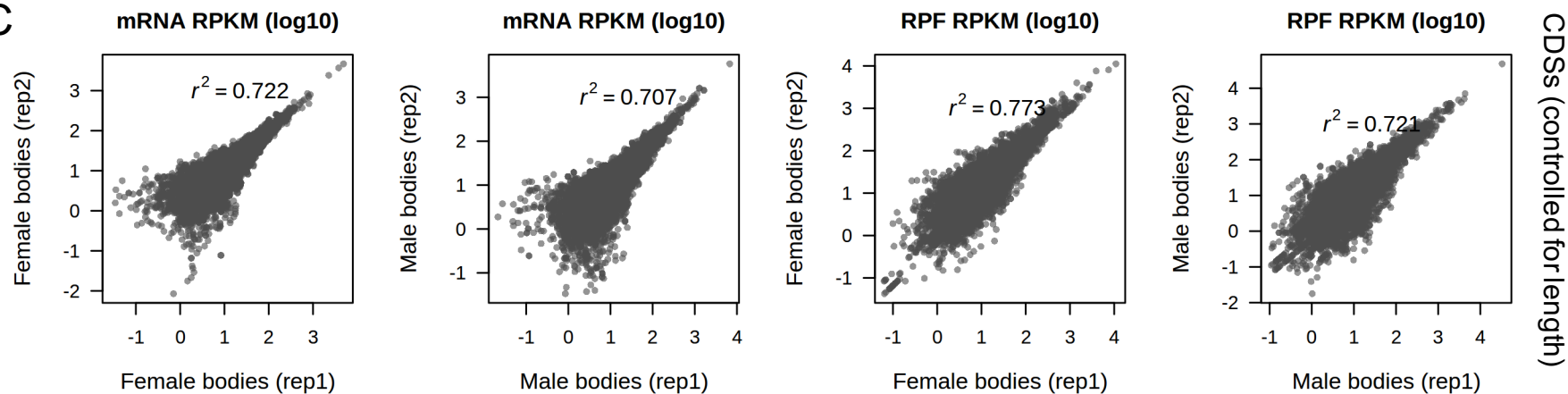

Supplement: S2 Fig — (A) High correlations in RPKM of CDSs between the two biological replicates. (B) Correlations in RPKM of uORFs between the two biological replicates. (C) Correlations in RPKM of 5′ regions of CDSs that begin at the start codons and end at the same lengths of uORFs in the 5′ UTRs. The raw data for panels (A-C) can be found in S5 Data. CDS, coding DNA sequence; RPKM, reads per kilobase of transcript per million mapped reads; uORF, upstream open reading frame; UTR, untranslated region. (PDF) [file pbio.2003903.s019.pdf]

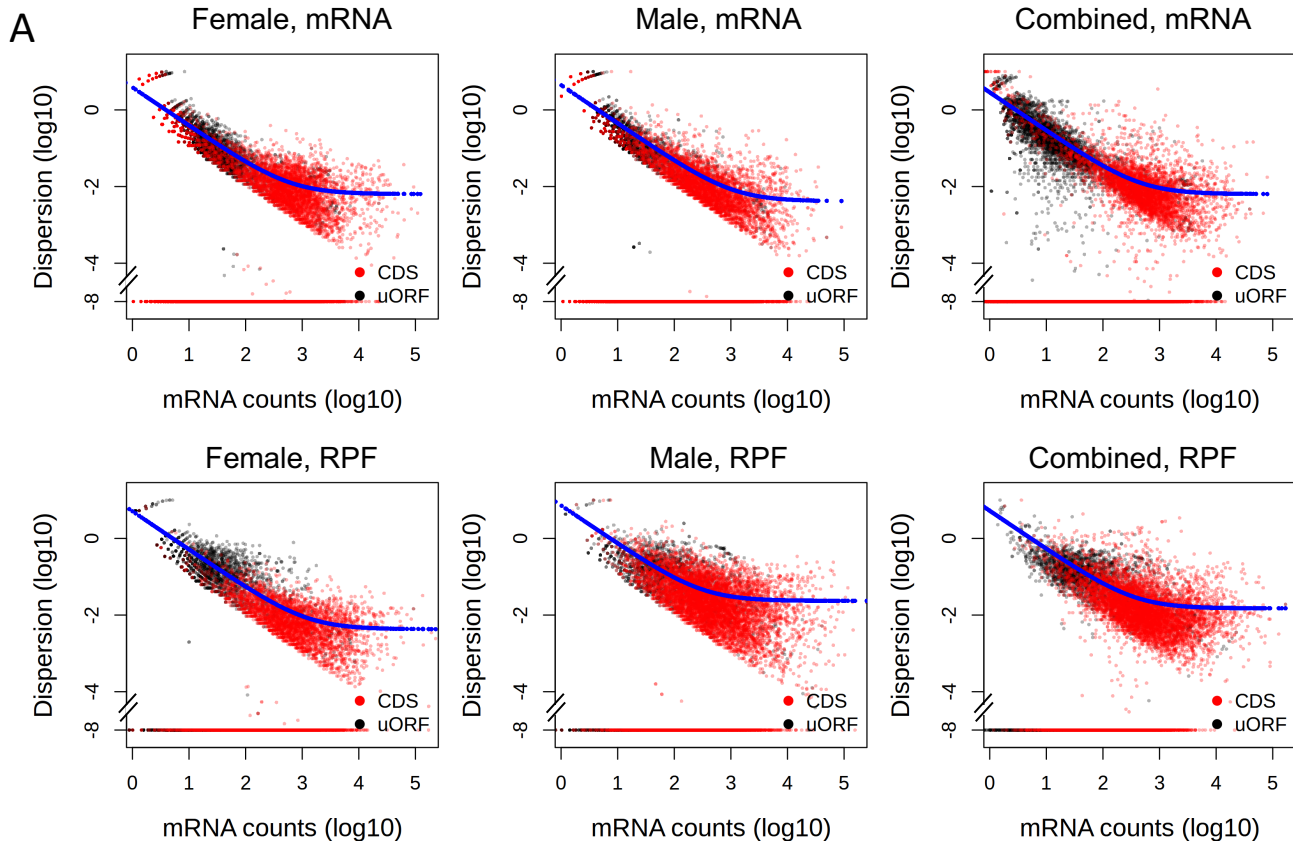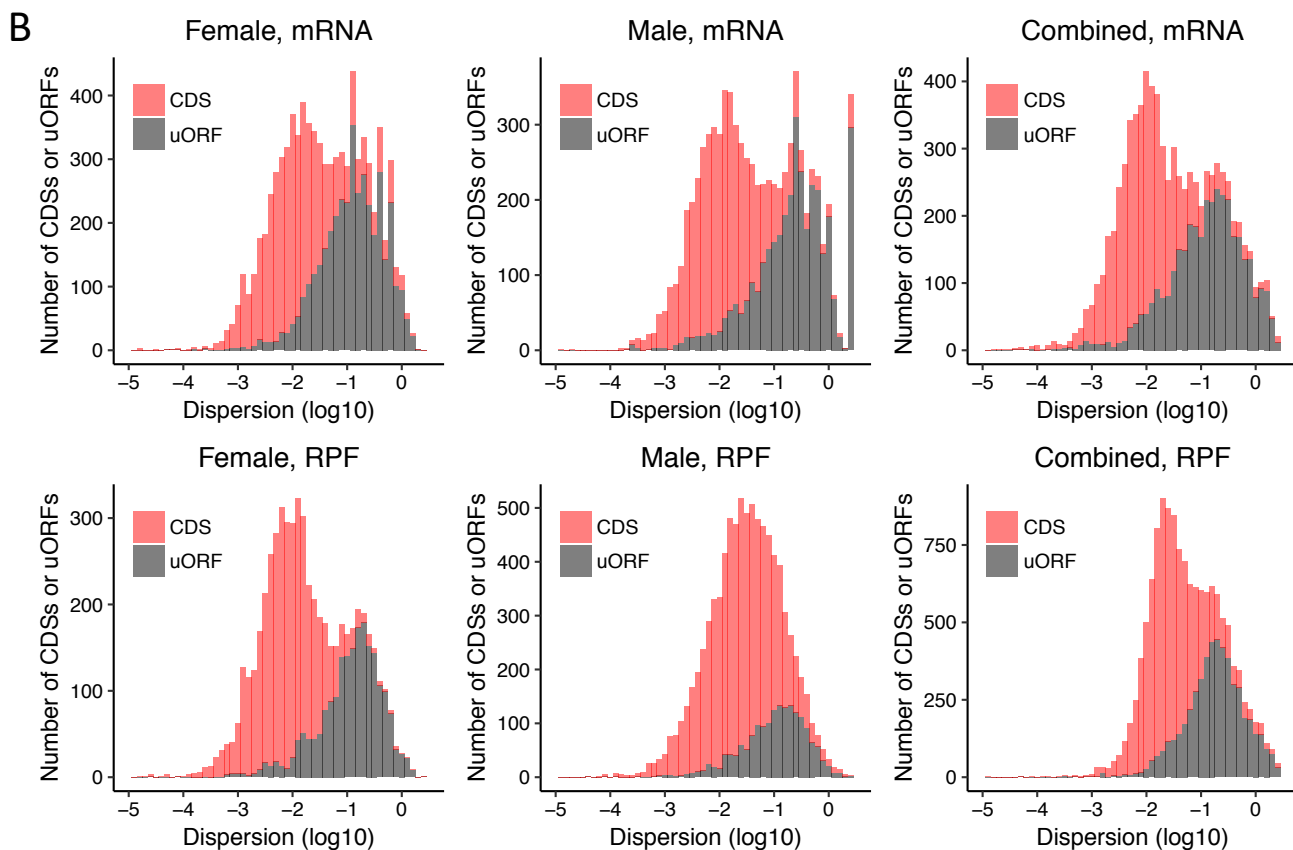

Supplement: S3 Fig — (A) Maximum likelihood estimation of dispersions of mRNA or RPF read counts for CDSs (red) and uORFs (black) in biological replicates of female bodies, male bodies, or combined data. Dispersions of mRNA read counts were estimated for features (CDSs and uORFs) with mRNA RPKM ≥ 1. Dispersions of RPF counts were only estimated for well-transcribed features. (RPKM ≥ 1 and normalized reads ≥ 30 in mRNA-Seq). The blue lines are fit of dispersions against average read counts of biological replicates and reflect the dispersion-mean dependency. (B) The distribution of dispersions of NGS read counts for uORFs and CDSs as shown in (A). The raw data for panels (A and B) can be found in S6 Data. CDS, coding DNA sequence; NGS, next-generation sequencing; RPF, ribosome-protected mRNA fragment; RPKM, reads per kilobase of transcript per million mapped reads; uORF, upstream open reading frame; UTR, untranslated region. (PDF) [file pbio.2003903.s020.pdf]

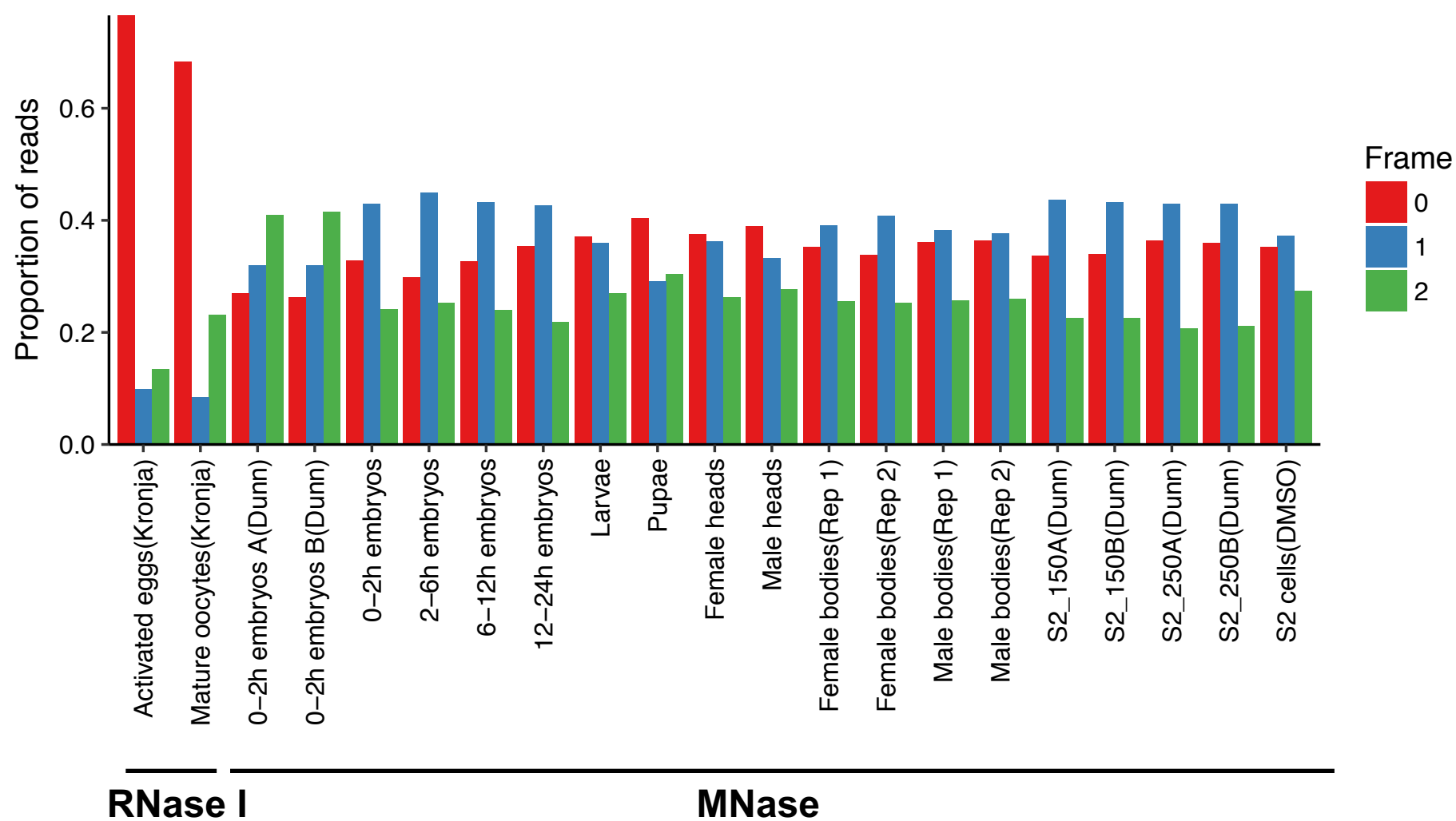

Supplement: S4 Fig — The enzymes used in digestion were presented under the bars. The 3 nt periodicity of the 5′ RPF reads mapped along CDSs is readily manifested in the RNase I experiment [65] but compromised in the MNase experiments by Dunn and colleagues [64] and Ribo-Seq data generated in this study. The raw data can be found in S1 Data. CDS, coding DNA sequence; MNase, micrococcal nuclease; RPF, ribosome-protected mRNA fragment. (PDF) [file pbio.2003903.s021.pdf]

Site coverage (number of reads)

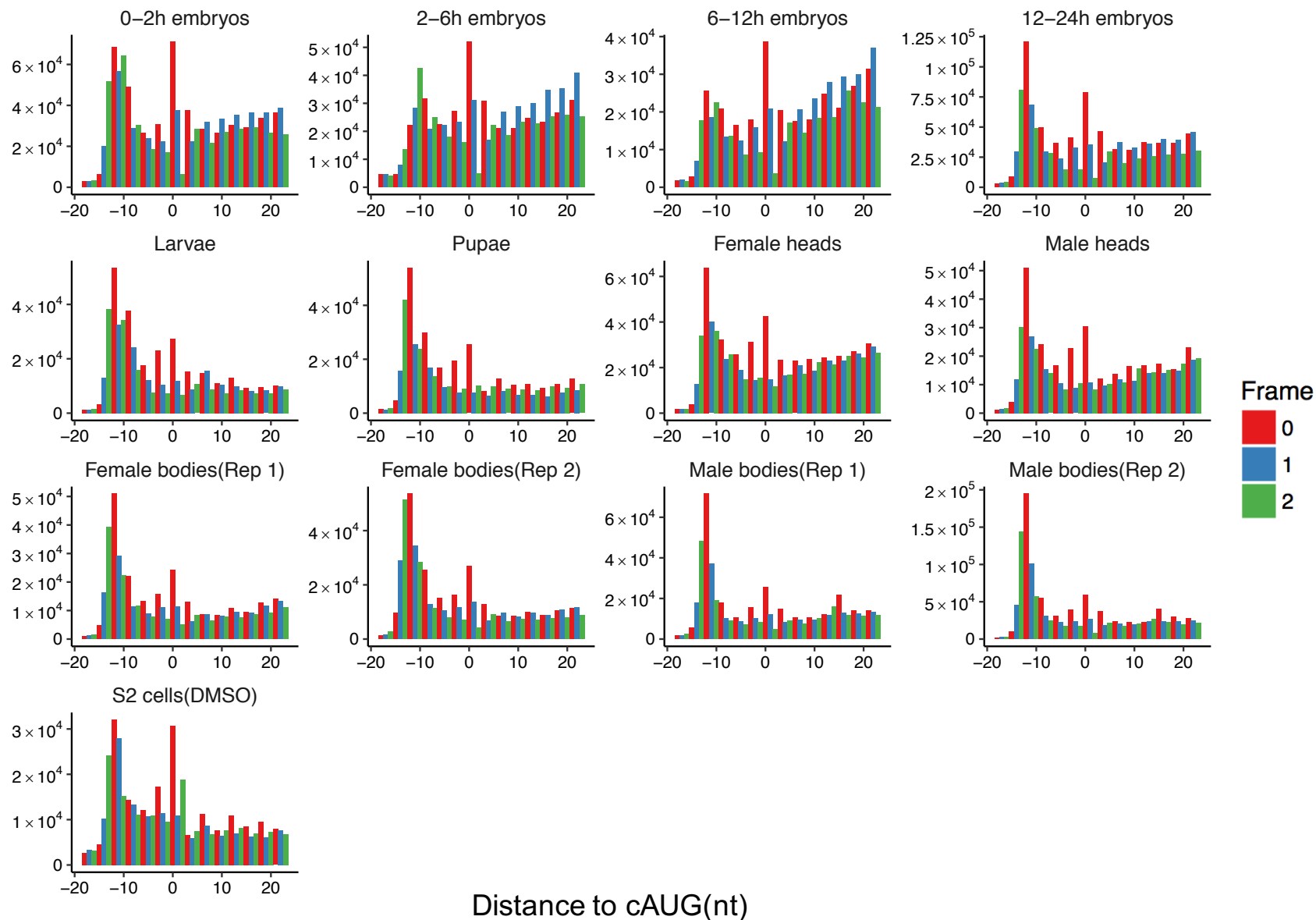

Supplement: S5 Fig — The raw data can be found in S1 Data. cAUG, AUG start codon of coding DNA sequence; RPF, ribosome-protected mRNA fragment. (PDF) [file pbio.2003903.s022.pdf]

Normalized coverage

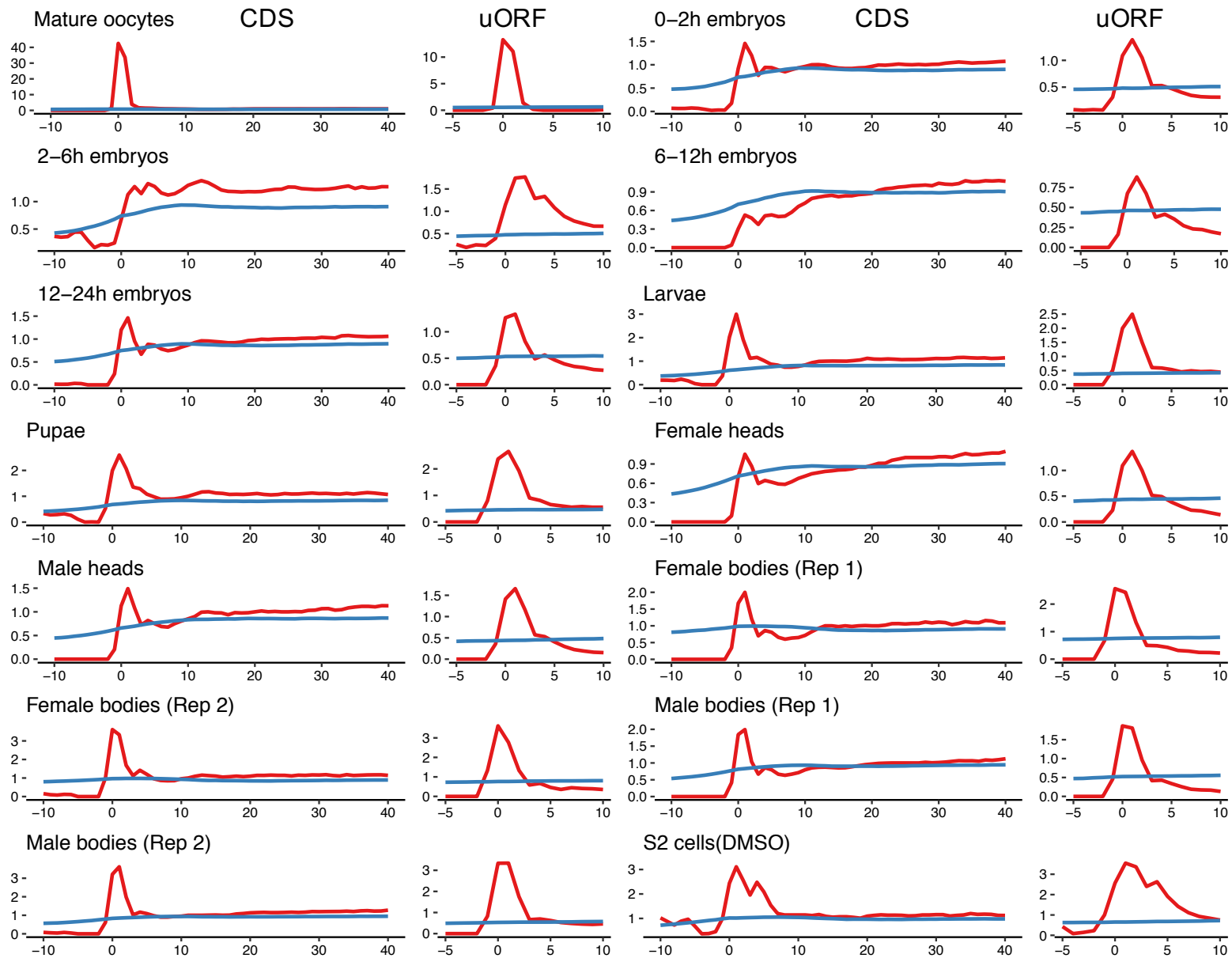

Distance to AUG (triplets) — Ribo-Seq — mRNA-Seq

Supplement: S6 Fig — The blue line and red line represent mRNA-Seq and Ribo-Seq of each sample, respectively. cAUG, AUG start codon of coding DNA sequence; uAUG, start codon of upstream open reading frame. (PDF) [file pbio.2003903.s023.pdf]

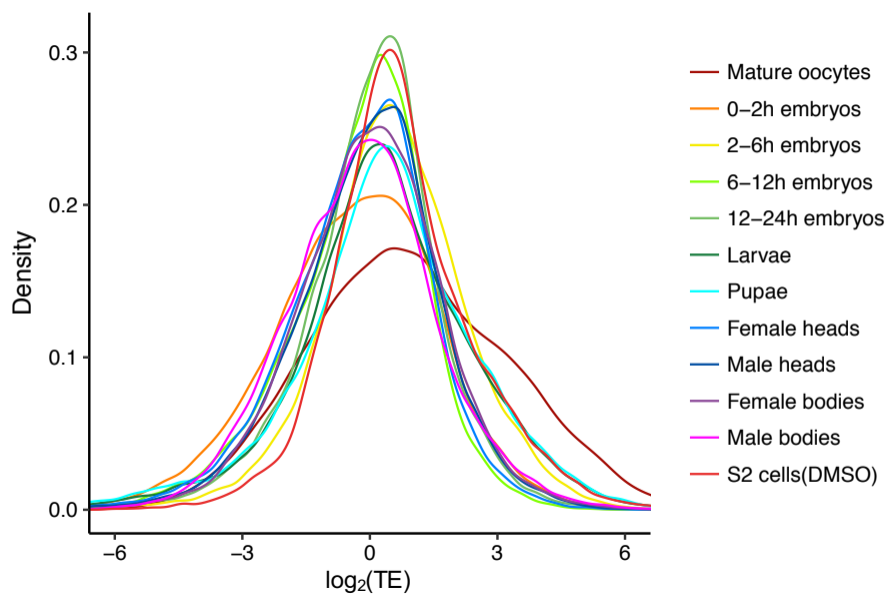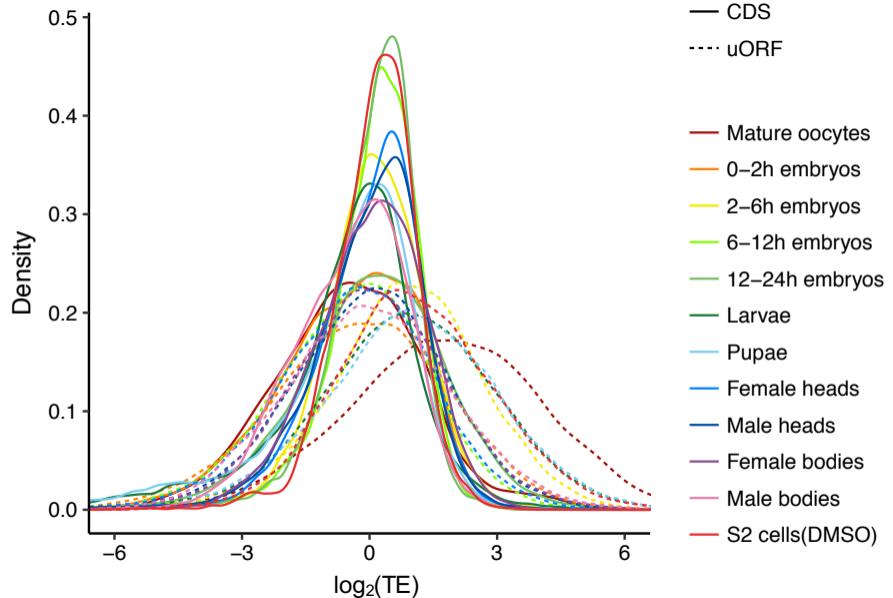

Supplement: S7 Fig — Only a feature that has mRNA RPKM ≥ 1 was considered. The left panel is the log2(TE) for all the features (CDSs and uORFs combined), and the log2(TE) for the uORFs and CDSs in a sample was drawn separately in the right panel. CDS, coding DNA sequence; RPKM, reads per kilobase of transcript per million mapped reads; TE, translational efficiency; uORF, upstream open reading frame. (PDF) [file pbio.2003903.s024.pdf]

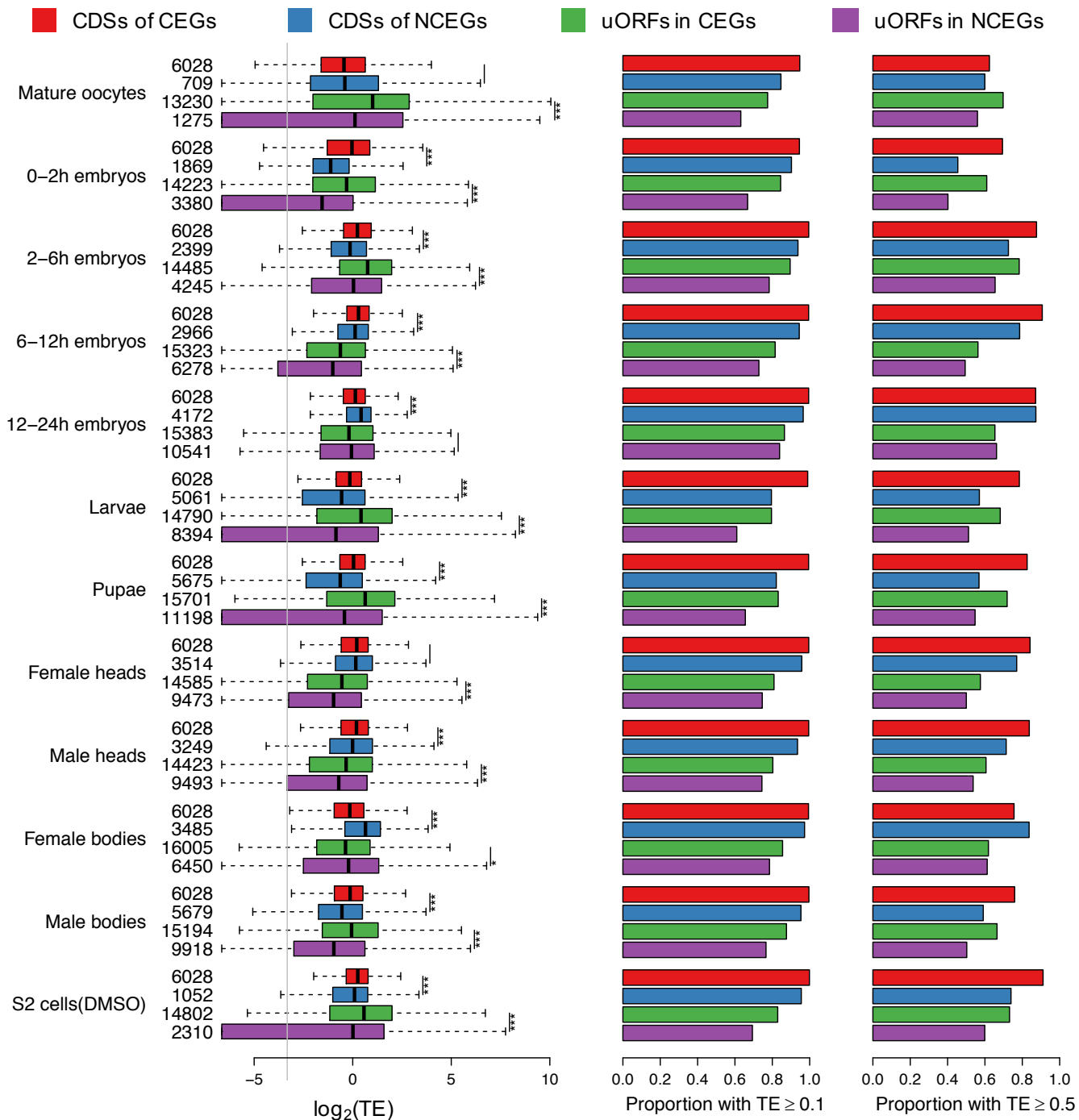

Supplement: S8 Fig — CEGs: genes that are expressed with mRNA-Seq RPKM ≥ 1 in all 12 samples. NCEGs: genes that are not constitutively expressed in all the samples but expressed with mRNA-Seq RPKM ≥ 1 in at least 1 of the 12 samples. The number of expressed genes or uORFs are displayed beside each box in the left panel. The raw data can be found in S7 Data. CDS, coding DNA sequence; CEG, constitutively expressed gene; NCEG, nonconstitutively expressed gene; RPKM, reads per kilobase of transcript per million mapped reads; TE, translational efficiency; uORF, upstream open reading frame. (PDF) [file pbio.2003903.s025.pdf]

**A** Genes without ribosome-associated uORFs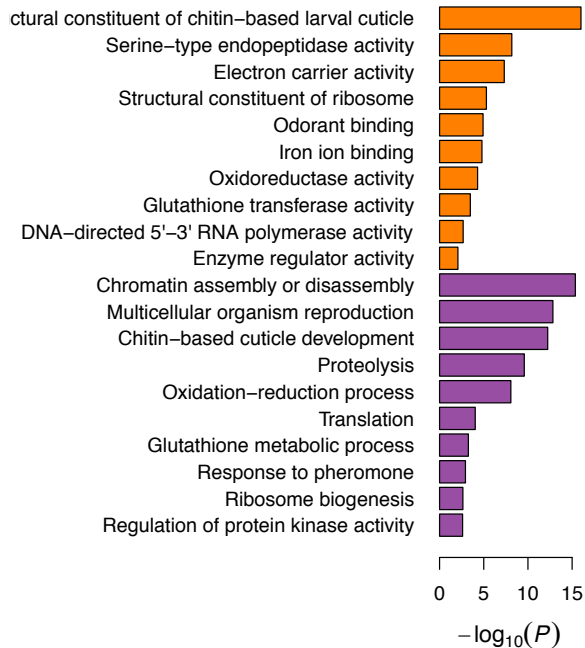**B** Genes with ribosome-associated uORFs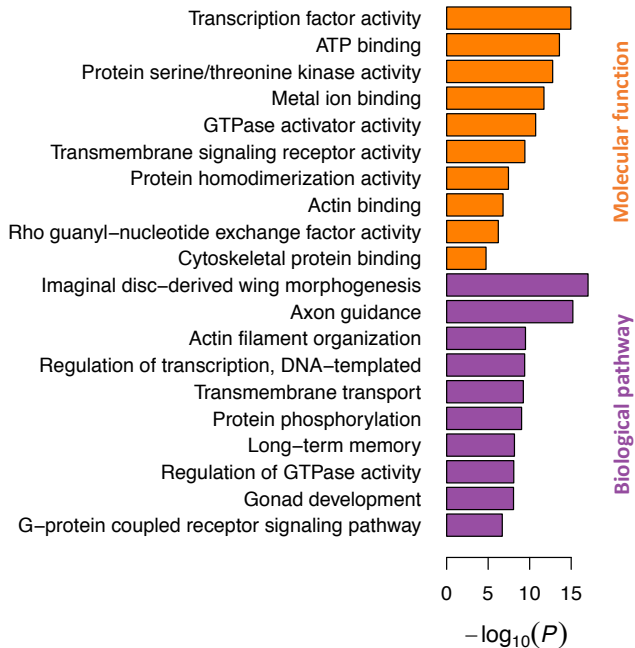

Supplement: S9 Fig — The GO enrichment analysis of genes without ribosome-associated uORFs (A) or genes with ribosome-associated uORFs (B). The raw data can be found in S5 Table. GO, gene ontology; uORF, upstream open reading frame. (PDF) [file pbio.2003903.s026.pdf]

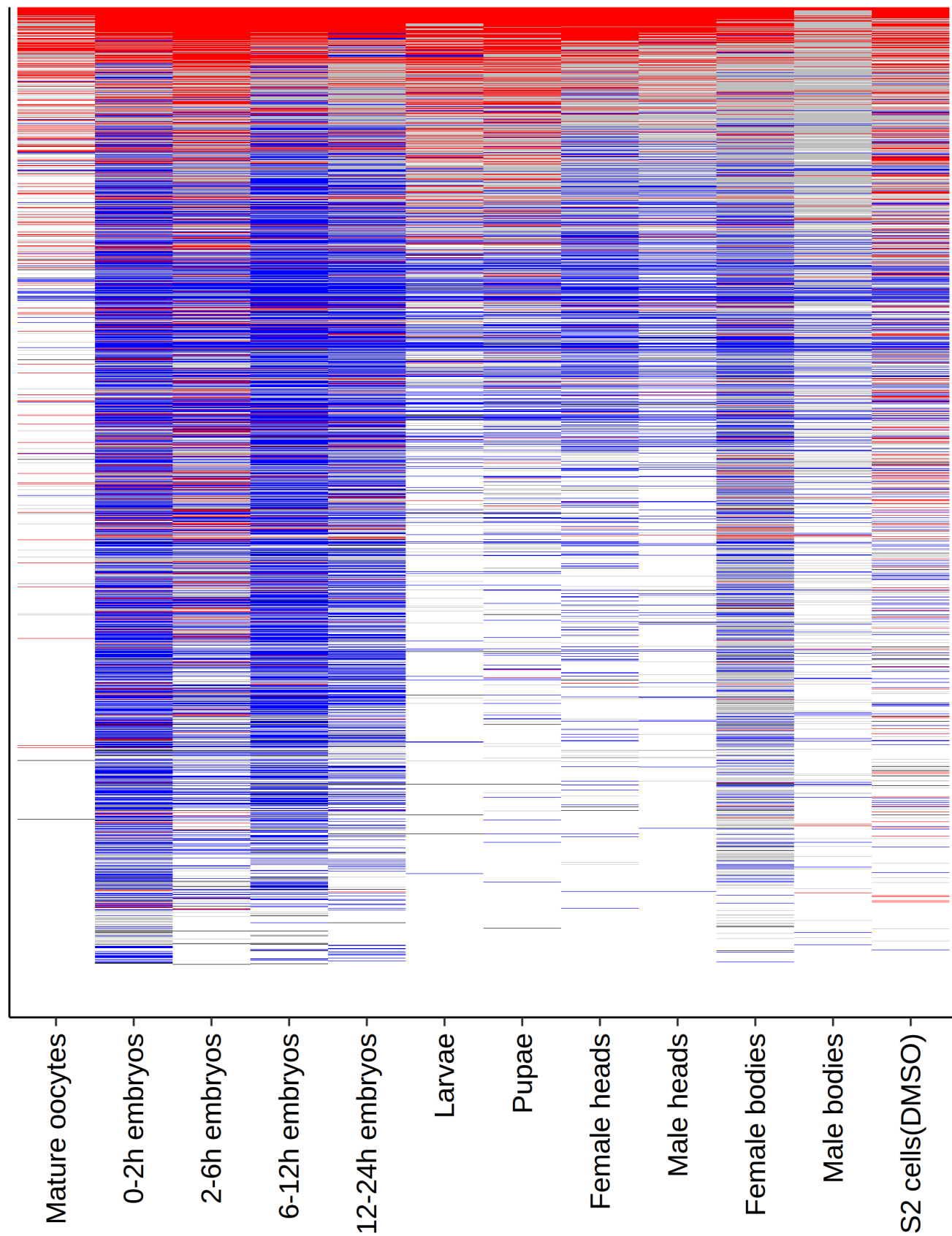

### Categories

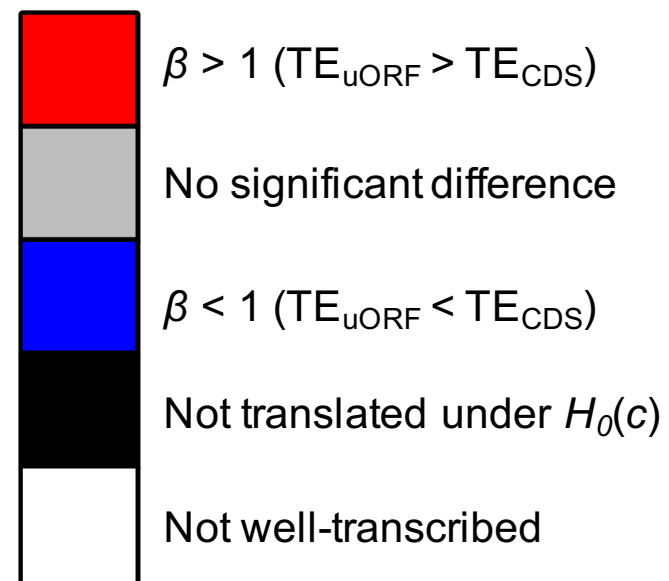

Supplement: S11 Fig — Each row represents a uORF, and each column represents a sample. uORFs that are well transcribed (RPKM ≥ 1 and normalized reads ≥ 30 in mRNA-Seq) and have β > 1 or β < 1 in a sample at the FDR of 0.05 are shown in red and blue, respectively. uORFs that are well-transcribed but not translated in a sample under H0(c) at the FDR of 0.05 are shown in black. Well-transcribed uORFs that have no significant differences in TE compared to the downstream CDSs in a sample are shown in gray. The remaining uORFs that are not well transcribed are shown in white. The raw data can be found in S7 Data. CDS, coding DNA sequence; FDR, false discovery rate; RPKM, reads per kilobase of transcript per million mapped reads; TE, translational efficiency; uORF, upstream open reading frame. (PDF) [file pbio.2003903.s028.pdf]

mRNA

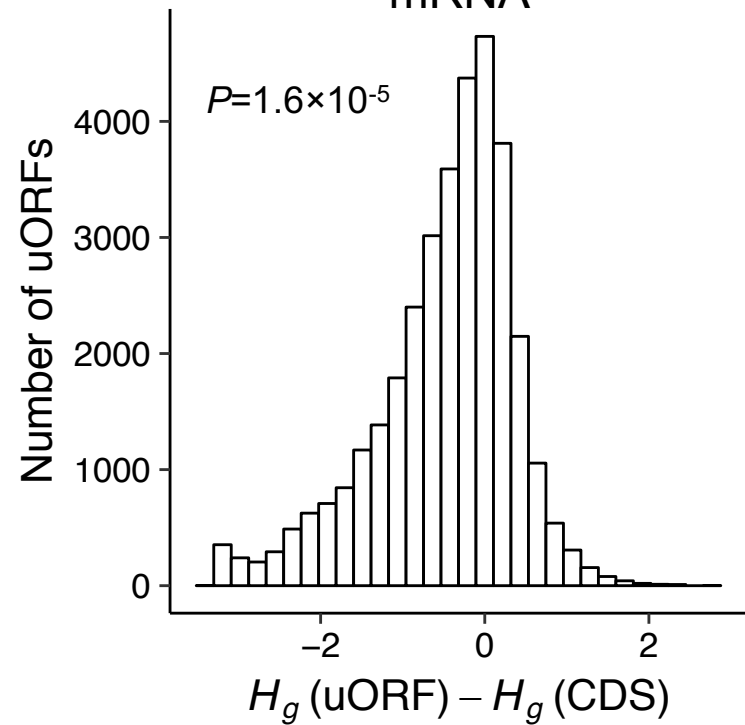

RPF

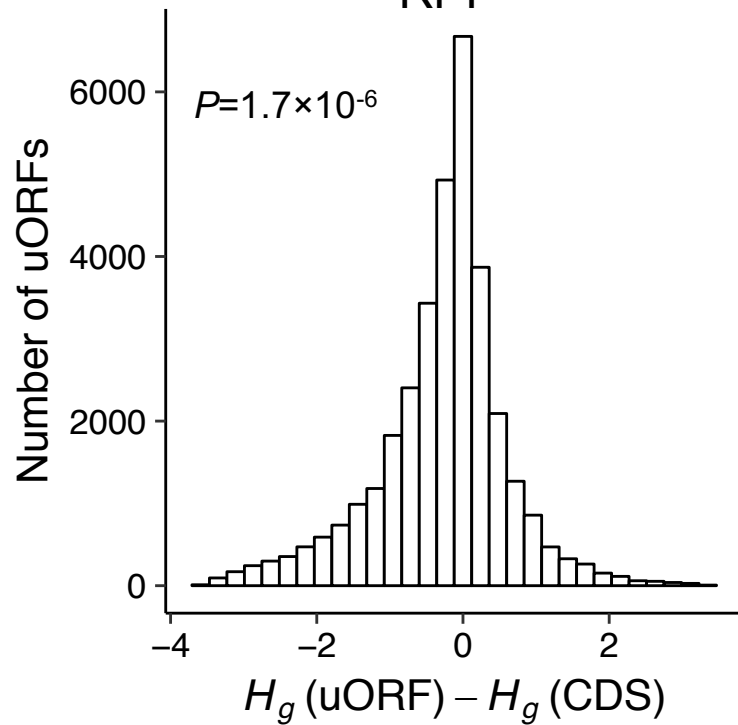

Supplement: S12 Fig — Differences in Hg between uORFs and the corresponding 5′ parts of CDSs in mRNA-Seq (left) and Ribo-Seq (right) data, respectively. For each uORF of n codons in length, the RPKM for the 5′ part of the downstream CDS was calculated for a region of n codons beginning from the downstream cAUG in both mRNA-Seq and Ribo-Seq data. Hg was calculated based on the RPKM for the uORFs or the 5′ parts of CDSs in the mRNA-Seq and Ribo-Seq data, respectively. Note in both mRNA-Seq and Ribo-Seq that the uORFs have significantly lower Hg values compared to the 5′ parts of CDSs. The raw data can be found in S1 Data. The raw data can be found in S7 Data. CDS, coding DNA sequence; RPKM, reads per kilobase of transcript per million mapped reads; uORF, upstream open reading frame. (PDF) [file pbio.2003903.s029.pdf]

**A**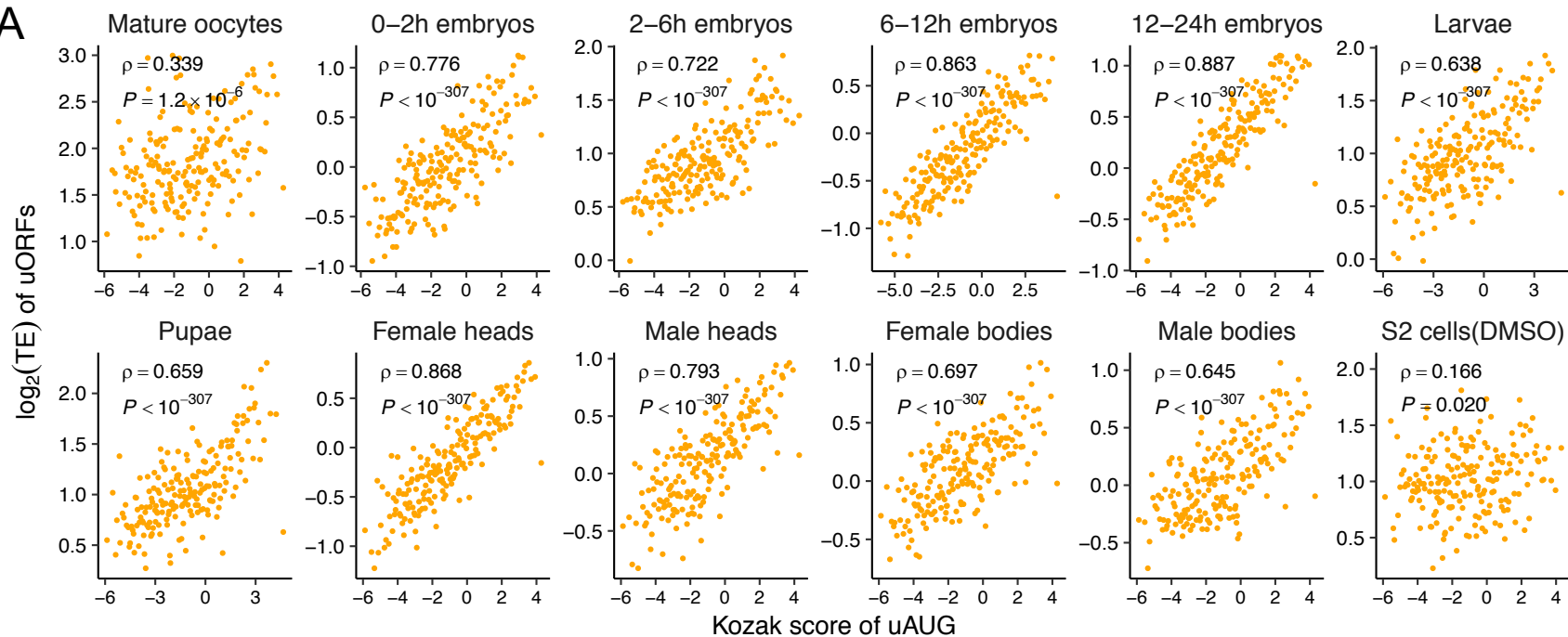**B**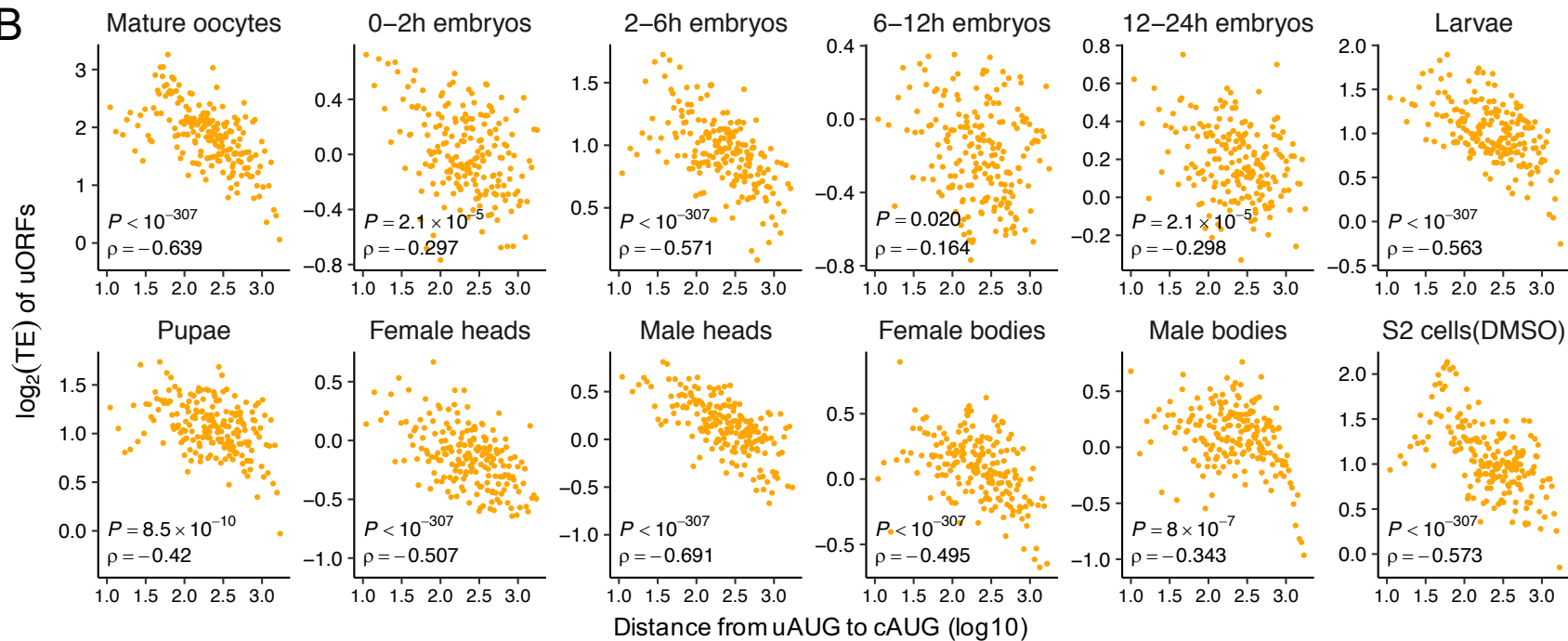

Supplement: S14 Fig — (A) Positive correlations between Kozak score of uAUG (x-axis) and log2(TE) (y-axis) of ribosome-associated uORFs in each of the 12 samples. The ribosome-associated uORFs were ranked with increasing Kozak score and were divided into 200 bins of equal size. Median Kozak score and median log2(TE) in each bin were displayed in the plot and used to calculate Spearman’s correlation. In each sample, only uORFs in genes with mRNA RPKM ≥ 1 and TE ≥ 0.5 were used in the analysis. (B) Negative correlations between the distance from uAUG to cAUG (x-axis) and log2(TE) (y-axis) of ribosome-associated uORFs in each of the 12 samples. The ribosome-associated uORFs were ranked with increasing distance from uAUG to cAUG and were divided into 200 bins of equal size. Median distance from uAUG to cAUG (log10 transformed) and median log2(TE) in each bin were displayed in the plot and used to calculate Spearman’s correlation. In each sample, only uORFs in genes with mRNA RPKM ≥ 1 and TE ≥ 0.5 were used in the analysis. The raw data for panels (A and B) can be found in S2 Data. cAUG, AUG start codon of coding DNA sequence; RPKM, reads per kilobase of transcript per million mapped reads; TE, translational efficiency; uAUG, AUG start codon of upstream open reading frame; uORF, upstream open reading frame. (PDF) [file pbio.2003903.s031.pdf]

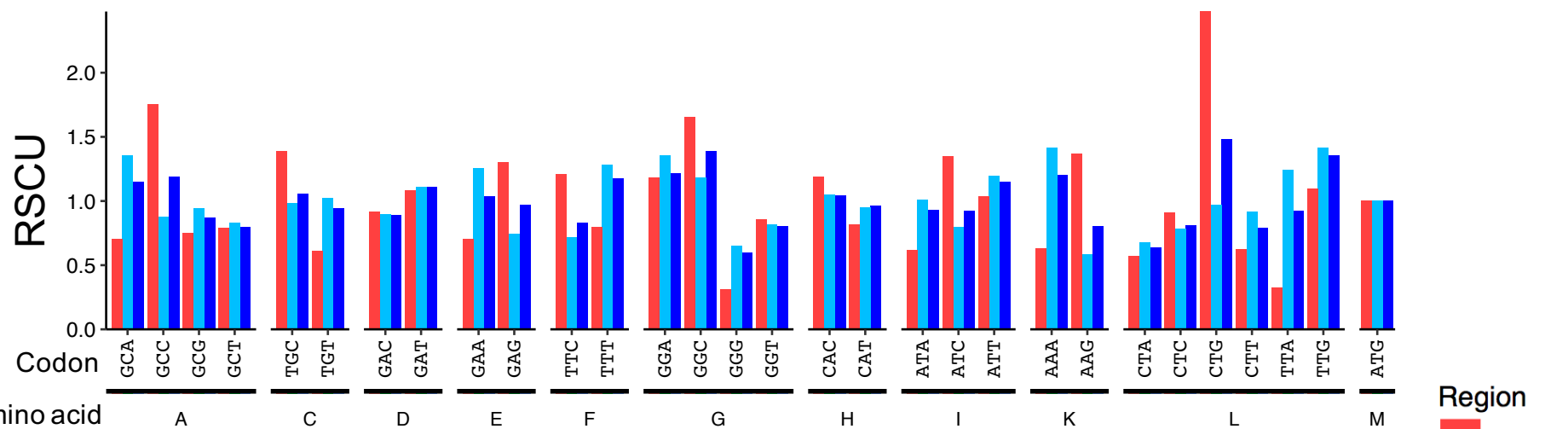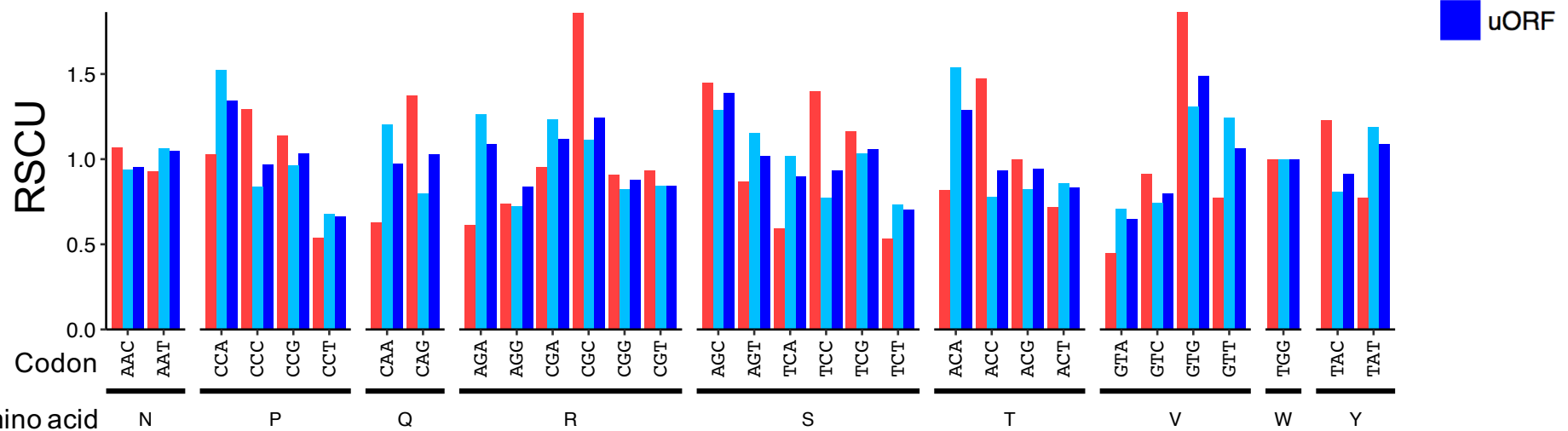

Supplement: S15 Fig — For uORFs and CDSs, both the start and stop codons are excluded. For uORFs, the regions overlapping with CDSs are also excluded. For each 5′ UTR, first reading frame of entire 5′ UTR is used to calculate triplet frequencies (stop codons were excluded). RSCU calculation was based on [89]. The raw data can be found in S1 Data. CDS, coding DNA sequence; RSCU, relative synonymous codon usage; uORF, upstream open reading frame; UTR, untranslated region. (PDF) [file pbio.2003903.s032.pdf]

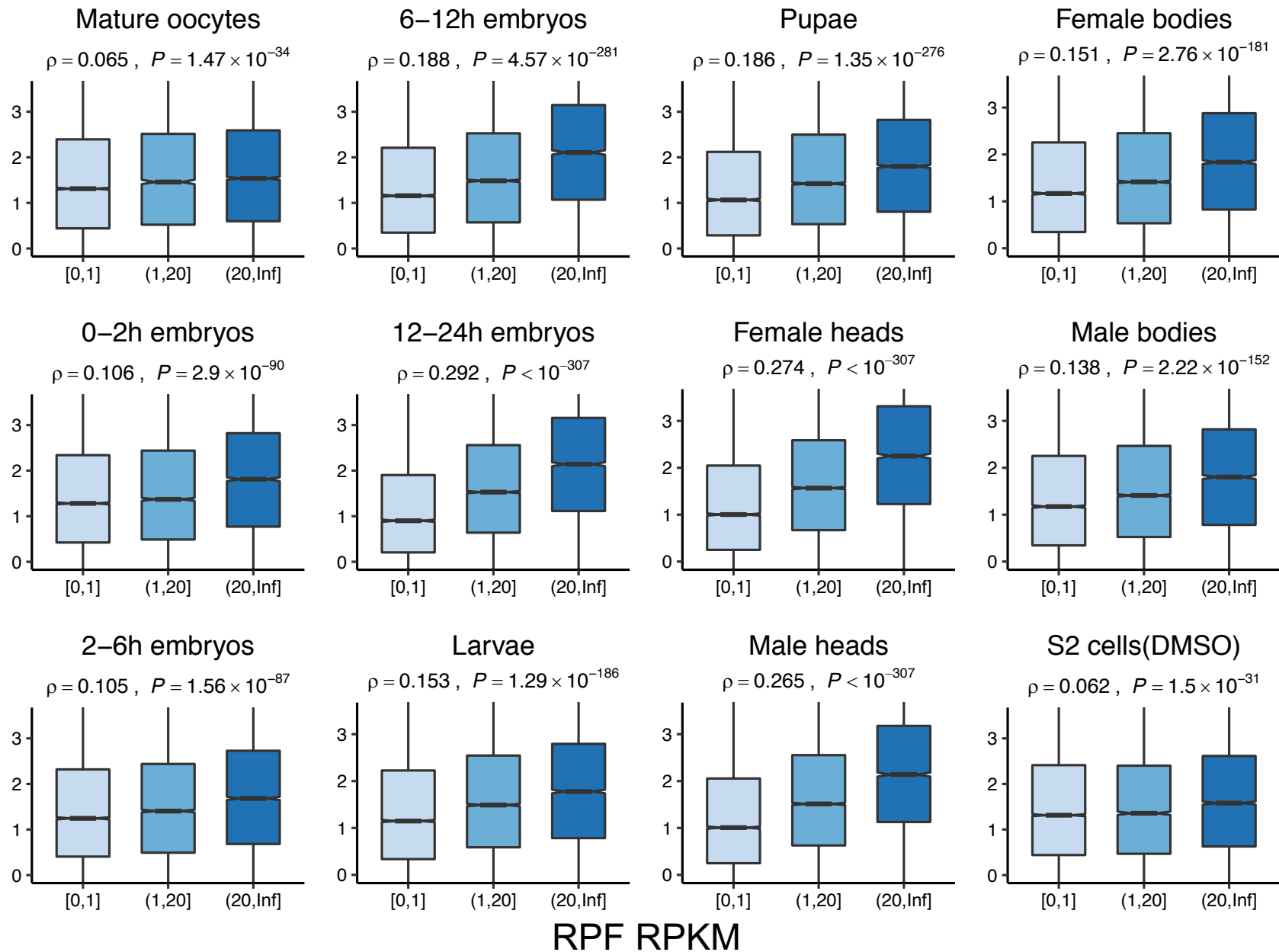

Supplement: S16 Fig — The raw data can be found in S8 Data. RPF, ribosome-protected mRNA fragment; RPKM, reads per kilobase of transcript per million mapped reads; uAUG, AUG start codon of uORF; uORF, upstream open reading frame. (PDF) [file pbio.2003903.s033.pdf]

**A**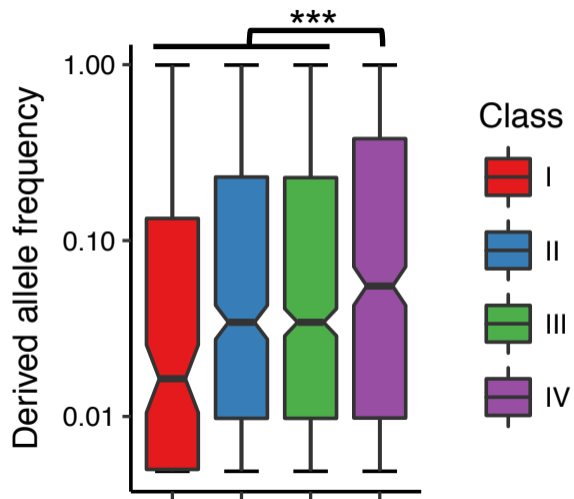**B**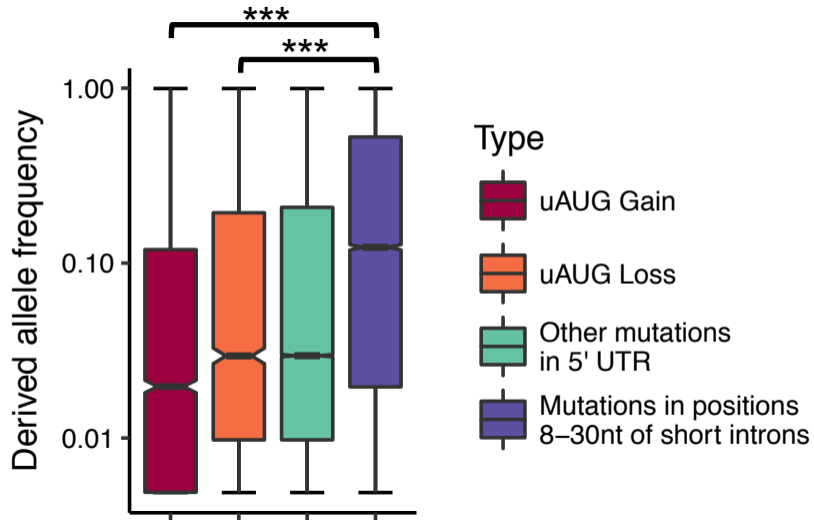

Supplement: S17 Fig — (A) The derived allele frequency of uAUGs (from Classes I to IV) that are polymorphic in D. melanogaster (***, P < 0.001). The raw data can be found in S1 Data. (B) Frequencies of the derived mutations that cause the gain or loss of uORFs in the 5′ UTR, the remaining derived mutations in the 5′ UTR, and the derived mutations in positions 8–30 nt of short introns in D. melanogaster (***, P < 0.001). The raw data can be found in S3 Data. DGRP, Drosophila Genetic Reference Panel; uAUG, AUG start codon of uORF; uORF, upstream open reading frame. (PDF) [file pbio.2003903.s034.pdf]

GDL

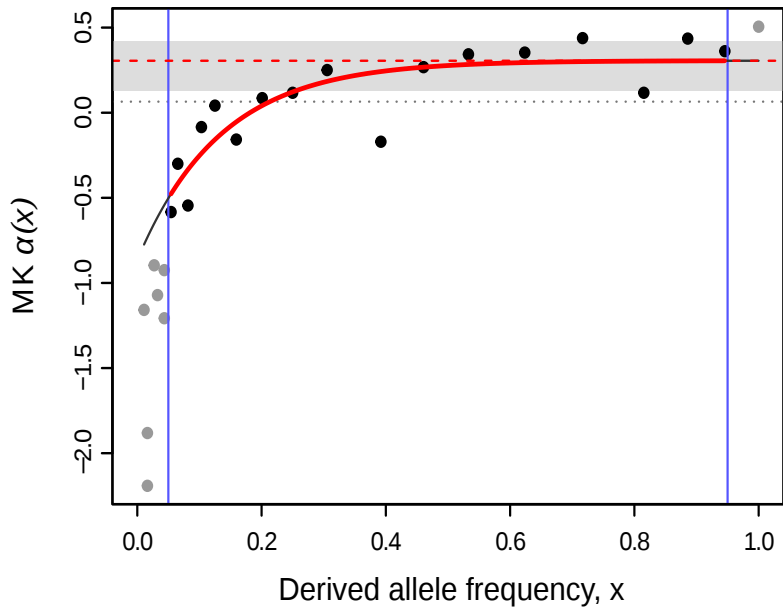

DGRP

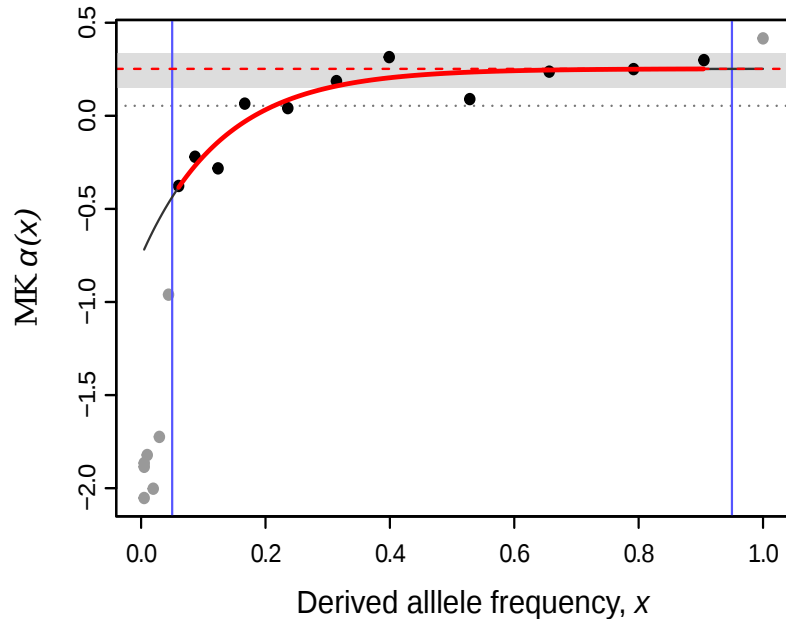

Supplement: S18 Fig — The estimation of αasym for newly fixed mutations in uAUGs by AsymptoticMK for the GDL (left) and DGRP (right) data. AUGs in 8–30 nt of short introns were used as the neutral control, and all mutations in the populations were used. The αori (the dashed line) was estimated with polymorphic sites whose derived allele frequencies were within 0.05–0.95 (delineated by the blue lines). To estimate αasym, the α values were calculated with polymorphic sites of different derived allele frequencies (x). An exponential function was fitted to the α values (red line). Gray bars denote the 95% confidence of αasym. The input for AsymptoticMK can be found in S1 Data. DGRP, Drosophila Genetic Reference Panel; GDL, Global Diversity Lines; uAUG, start codon of upstream open reading frame. (PDF) [file pbio.2003903.s035.pdf]

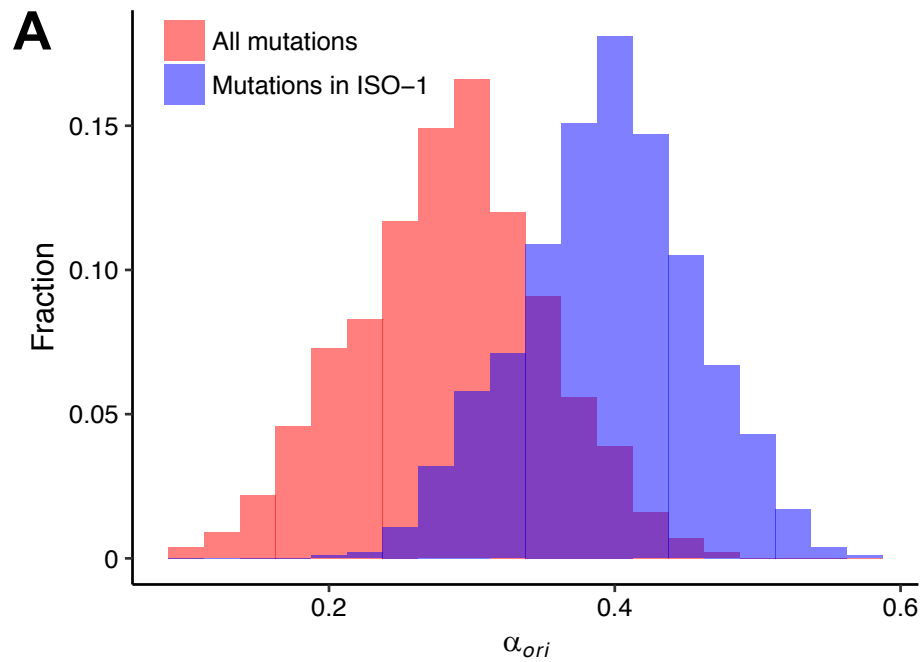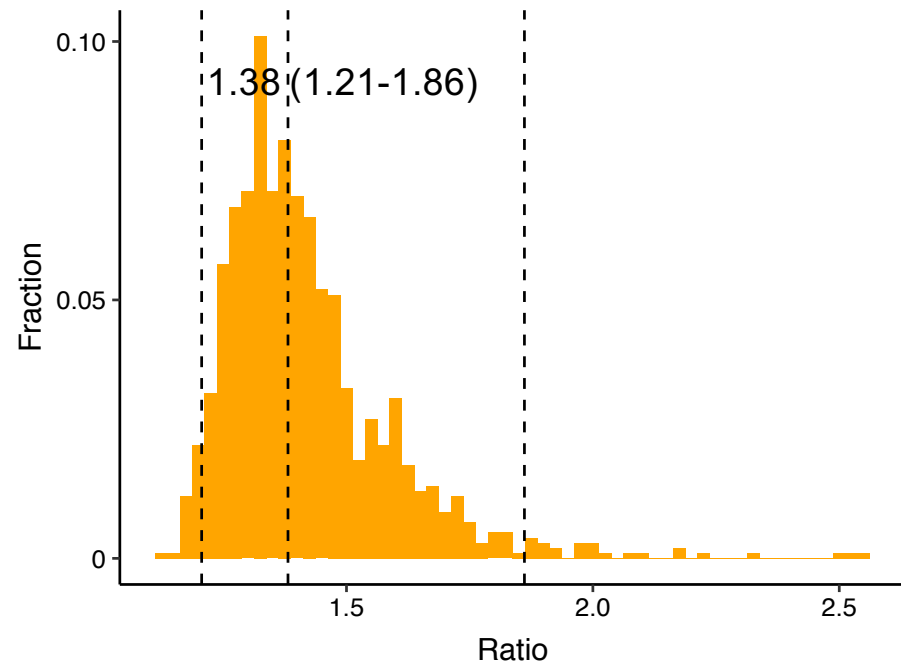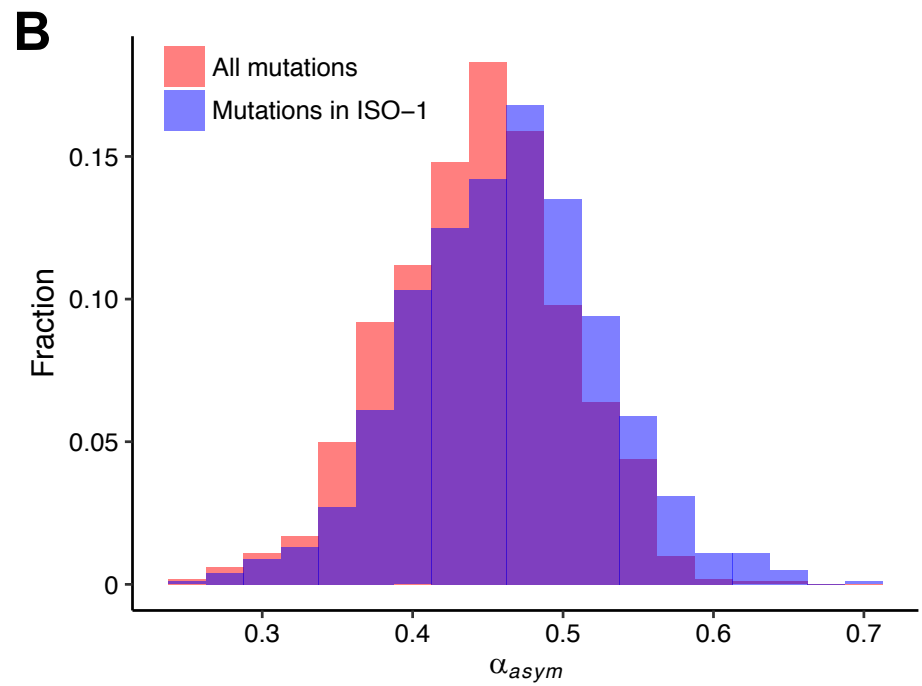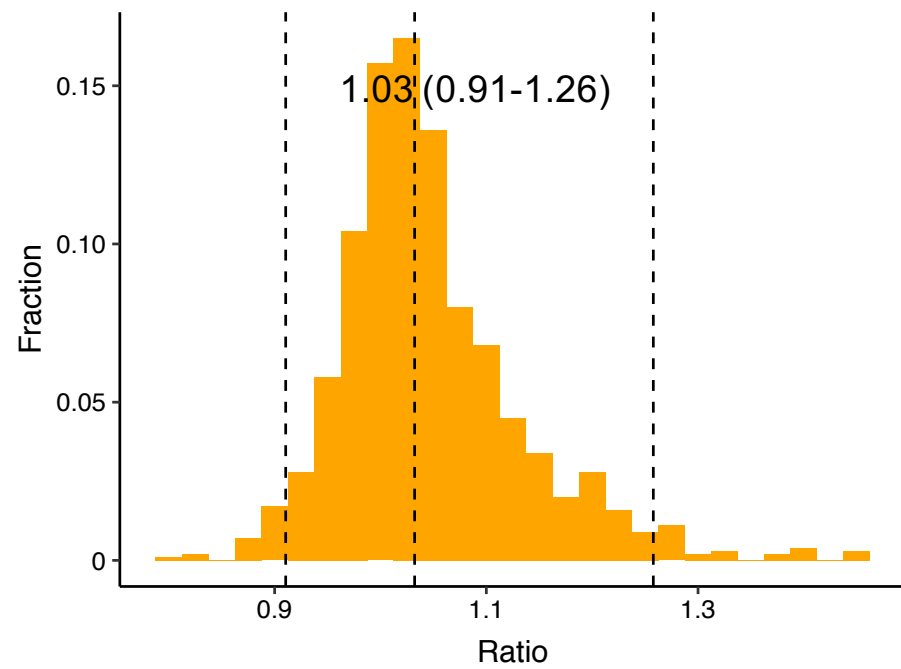

Supplement: S19 Fig — DGRP data were used in analysis, and both the original method and AsymptoticMK tests were performed. The simulations were repeated for 1,000 replicates. (A) The distribution of αori (left panel) estimated with all mutations in the populations (red) or only those present in ISO-1 strain (blue) and their ratios (the later versus the former, right panel). The median and the 2.5% and 97.5% quantiles of the ratios were shown and indicated with dashed lines. (B) Same as A but showing the results for αasym. The raw data can be found in S1 Data. CDS, coding DNA sequence; DGRP, Drosophila Genetic Reference Panel; MK test, McDonald-Kreitman test. (PDF) [file pbio.2003903.s036.pdf]

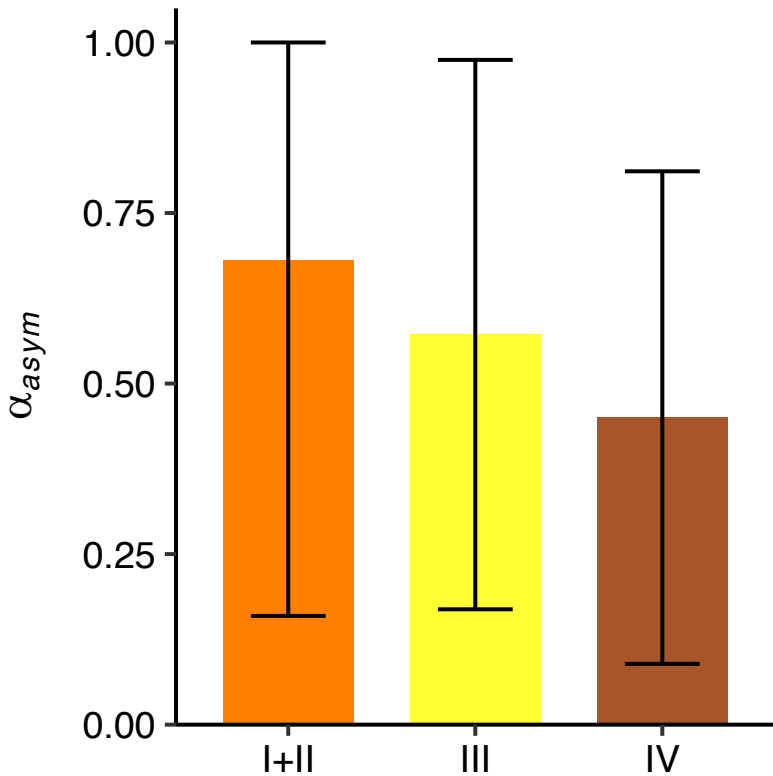

Supplement: S20 Fig — AUGs in 8–30 nt of short introns were used as the neutral control. Only mutations present in the ISO-1 strain of D. melanogaster were used. The error bars indicate 95% confidence intervals of αasym. The exact values can be found in S1 Data. DGRP, Drosophila Genetic Reference panel; uAUG, start codon of upstream open reading frame. (PDF) [file pbio.2003903.s037.pdf]

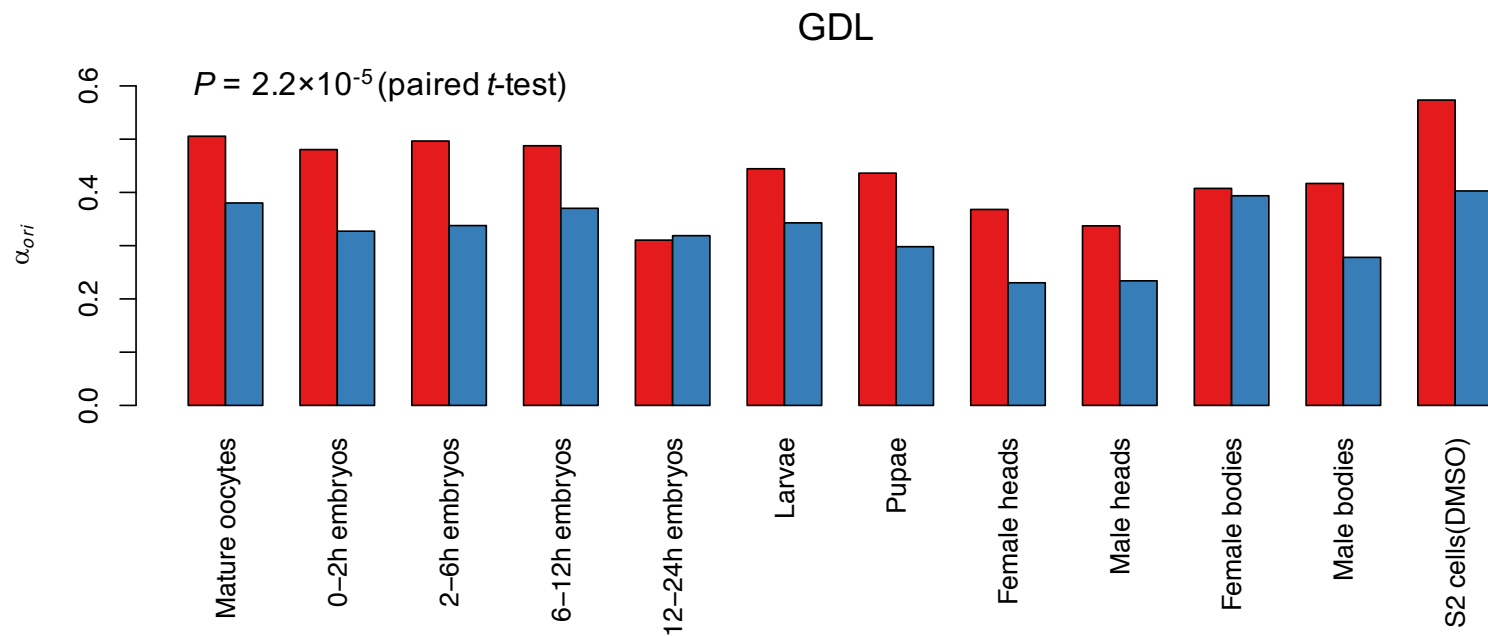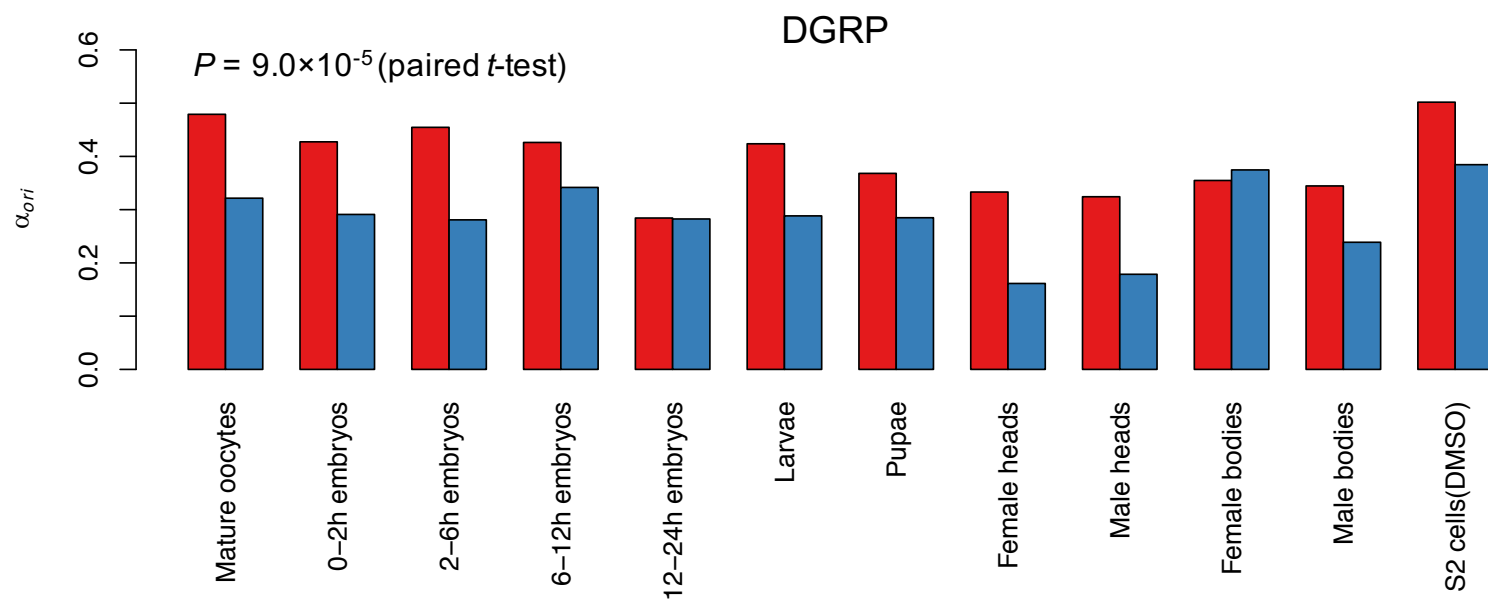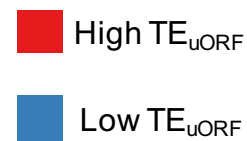

Supplement: S21 Fig — The newly fixed and the polymorphic uORFs that are expressed in a sample (mRNA RPKM ≥ 1) were combined and equally split into two groups based on TEuORF. Paired t tests were performed to compare differences in αori between the higher versus lower TE groups across samples. The exact values can be found in S1 Data. RPKM, reads per kilobase of transcript per million mapped reads; TE, translational efficiency; uAUG, AUG start codon of uORF; uORF, upstream open reading frame. (PDF) [file pbio.2003903.s038.pdf]

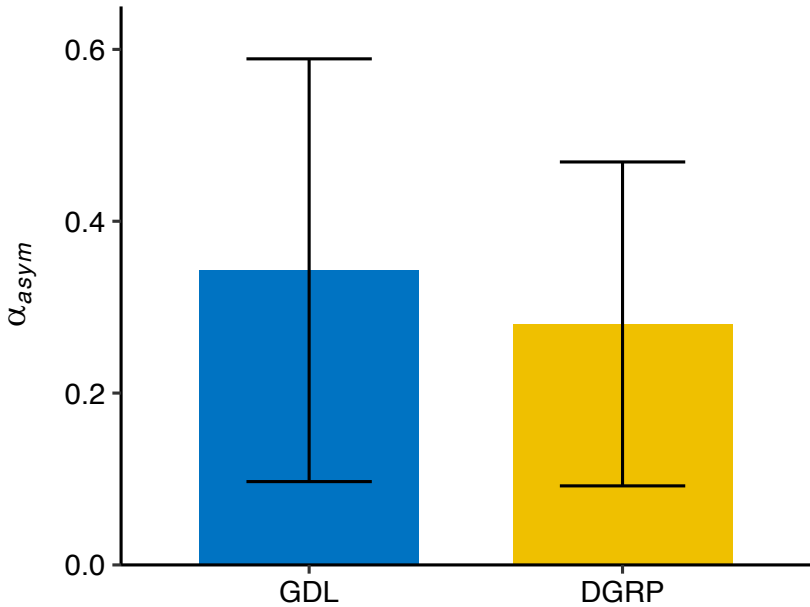

Supplement: S22 Fig — Only mutations present in the ISO-1 strain of D. melanogaster were used. The error bars indicate 95% confidence intervals of αasym. The exact values can be found in S1 Data. DGRP, Drosophila Genetic Reference Panel; GDL, Global Diversity Lines; uAUG, start codon of upstream open reading frame; UTR, untranslated region. (PDF) [file pbio.2003903.s039.pdf]

mRNA RPKM  $\geq 1$  and TE<sub>uORF</sub>  $\geq 0.1$ 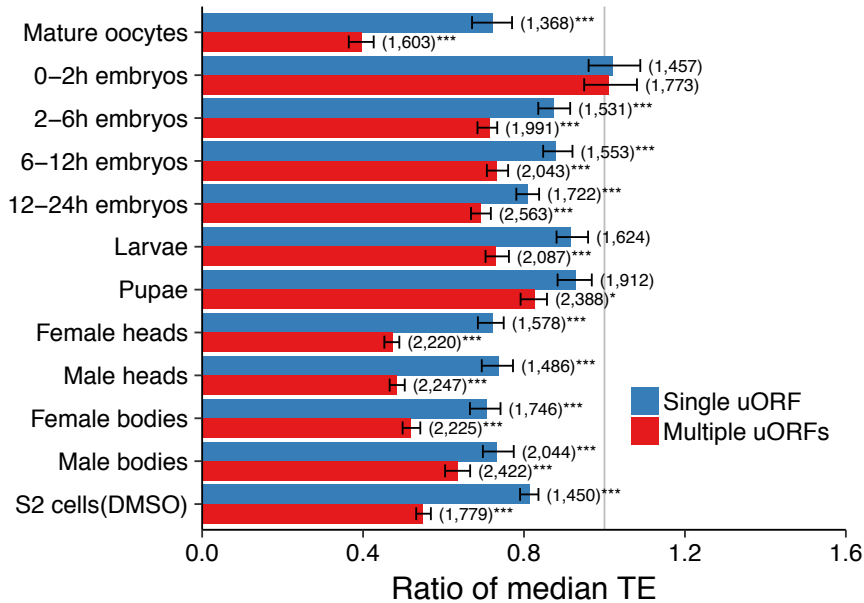mRNA RPKM  $\geq 1$  and TE<sub>uORF</sub>  $\geq 0.5$ 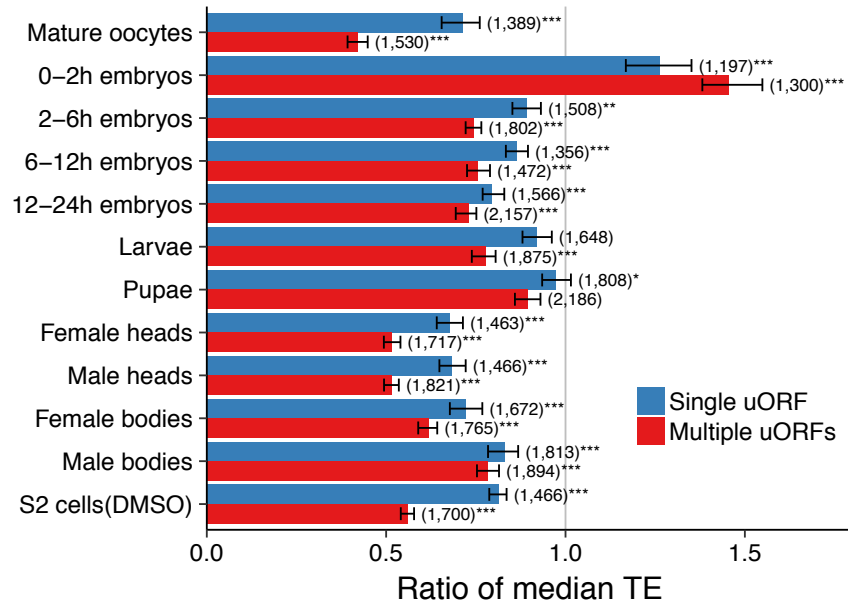

Supplement: S23 Fig — Wilcoxon rank-sum tests were performed to test the differences in each sample (*, P < 0.05; **, P < 0.01; ***, P < 0.001). Different cutoffs were used to define ribosome-associated uORFs as displayed above each plot. The raw data can be found in S4 Data. RPKM, reads per kilobase of transcript per million mapped reads; TE, translational efficiency; uORF, upstream open reading frame. (PDF) [file pbio.2003903.s040.pdf]

**A**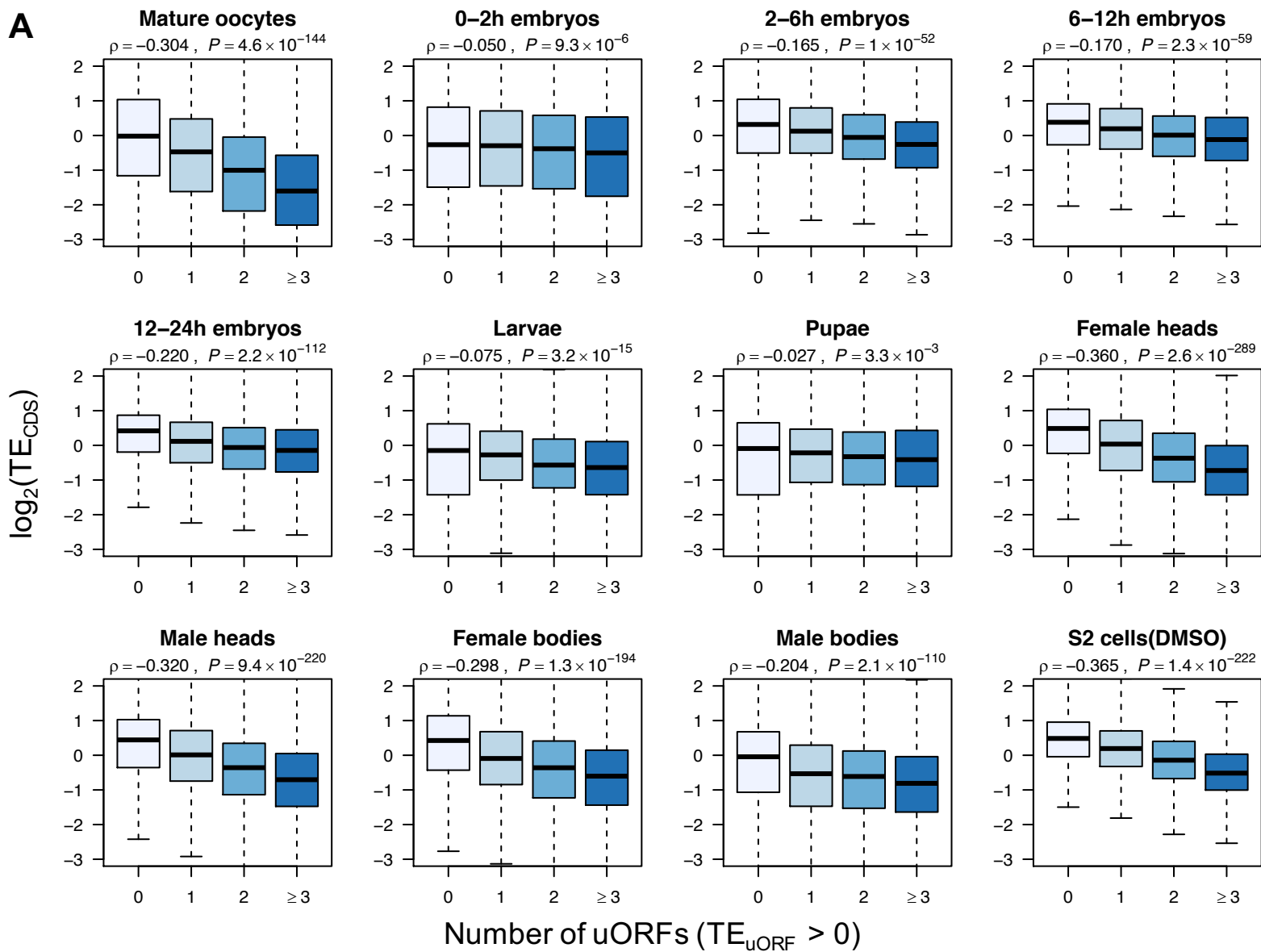

**B**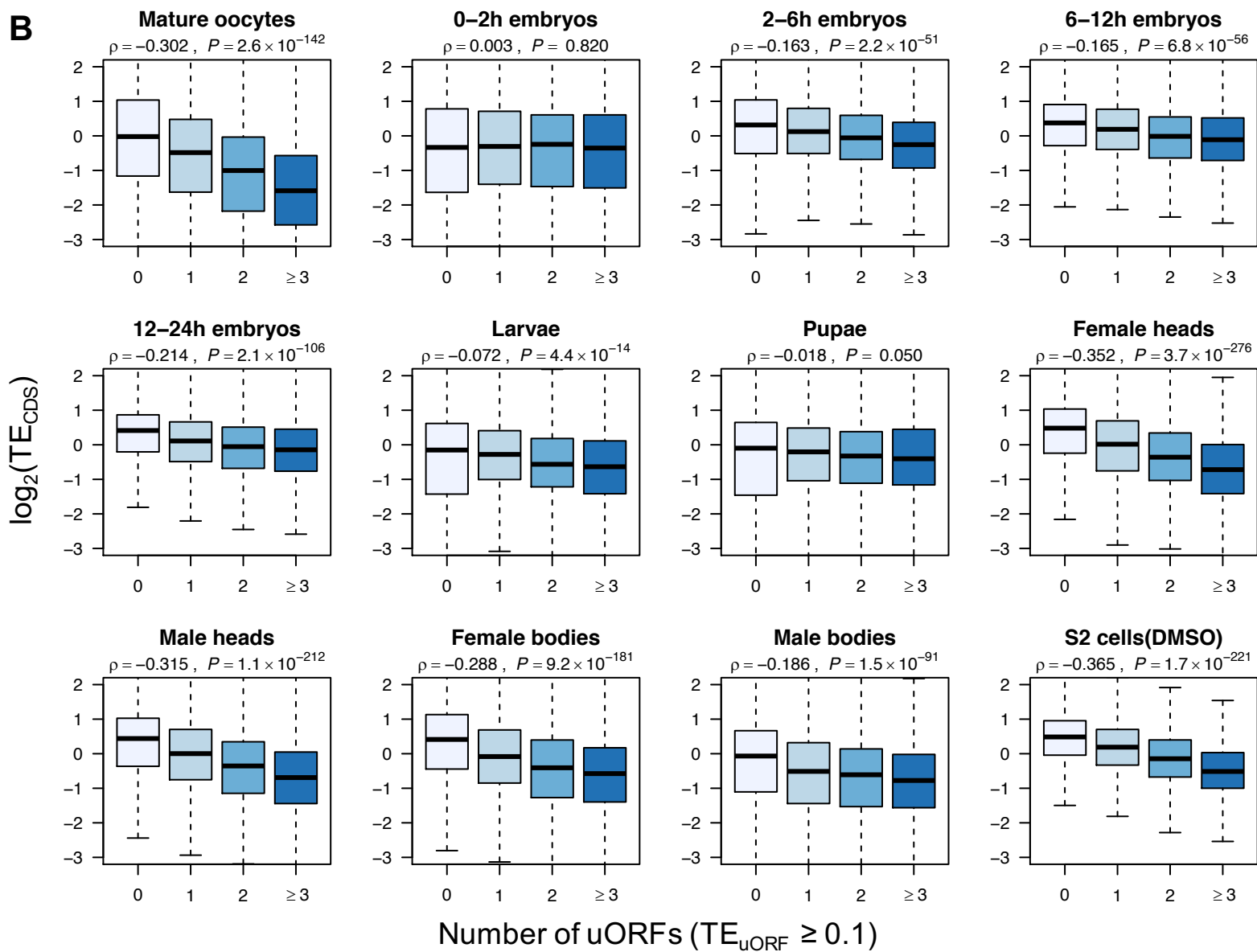

**C**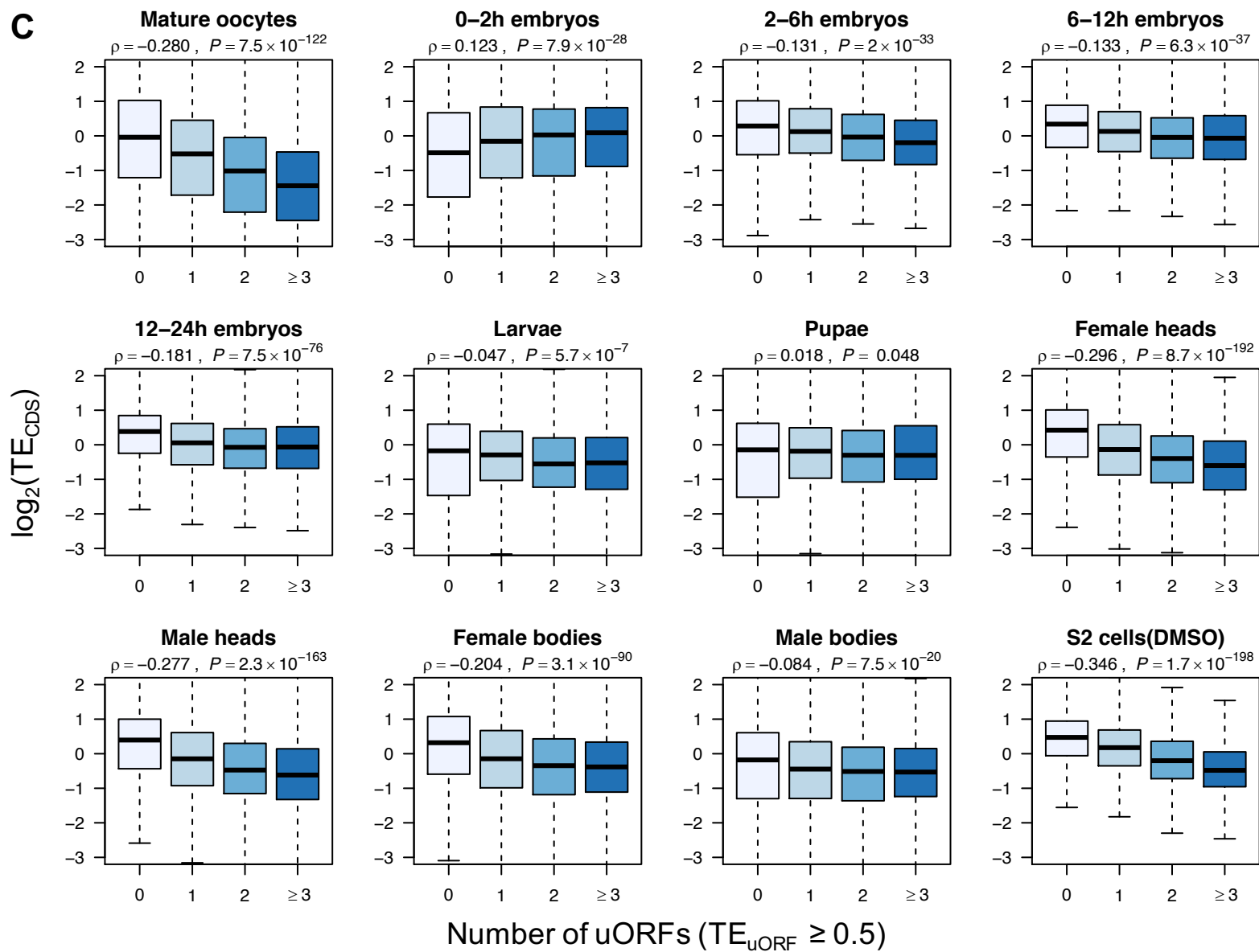

Supplement: S24 Fig — Different cutoffs were employed to define ribosome-associated uORFs: (A) mRNA RPKM ≥ 1, TE > 0; (B) mRNA RPKM ≥ 1, TE ≥ 0.1; and (C) mRNA RPKM ≥ 1, TE ≥ 0.5. The raw data can be found in S4 Data. CDS, coding DNA sequence; RPKM, reads per kilobase of transcript per million mapped reads; TE, translational efficiency; uORF, upstream open reading frame. (PDF) [file pbio.2003903.s041.pdf]

A

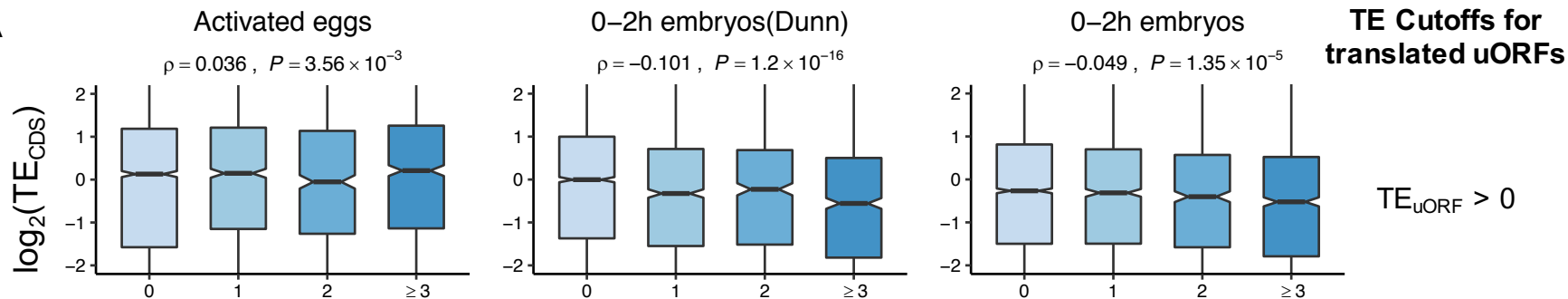

B

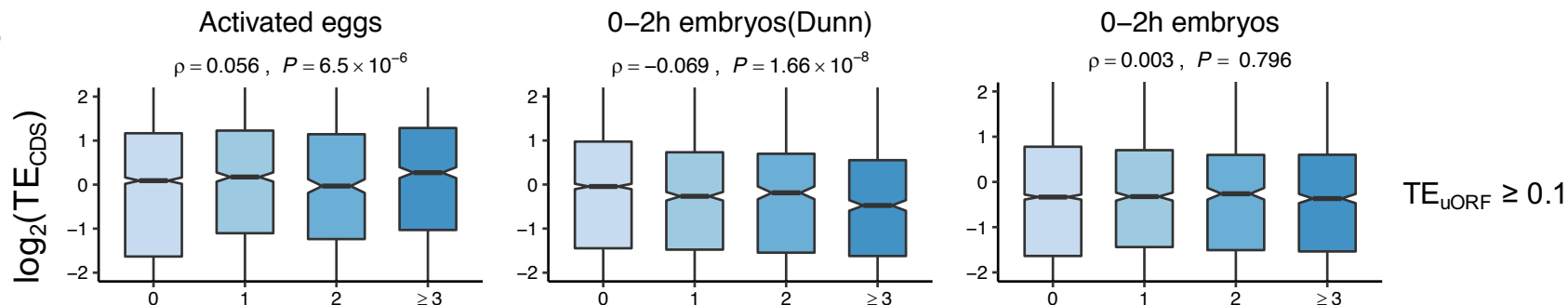

C

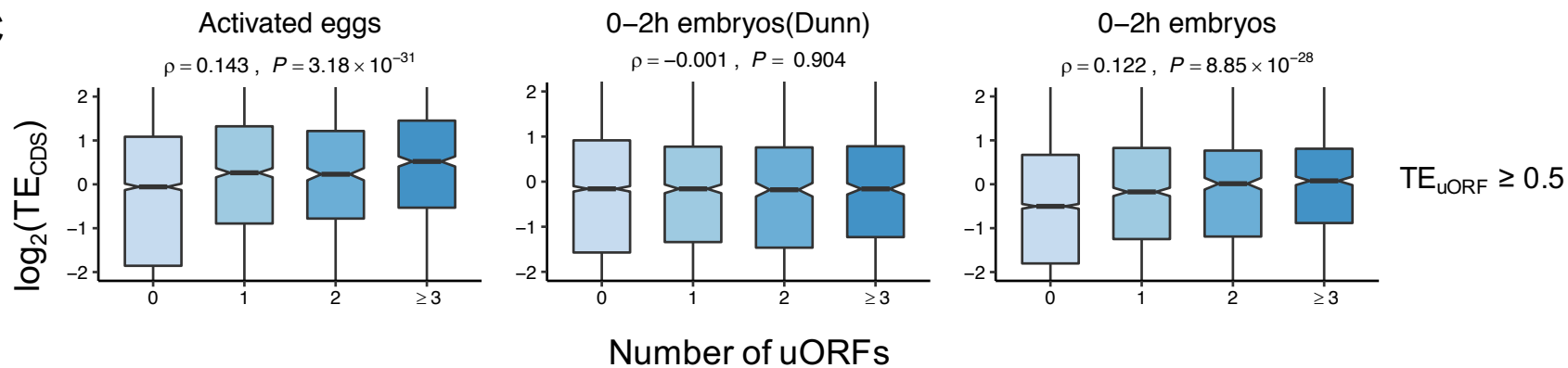

Supplement: S25 Fig — Different cutoffs were employed to define ribosome-associated uORFs: (A) mRNA RPKM ≥ 1, TE > 0; (B) mRNA RPKM ≥ 1, TE ≥ 0.1; (C) mRNA RPKM ≥ 1, TE ≥ 0.5. The “activated egg” is 0–2 h embryos studied by Kronja and colleagues [65]. The raw data can be found in S1 Data. CDS, coding DNA sequence; RPKM, reads per kilobase of transcript per million mapped reads; TE, translational efficiency; uORF, upstream open reading frame. (PDF) [file pbio.2003903.s042.pdf]

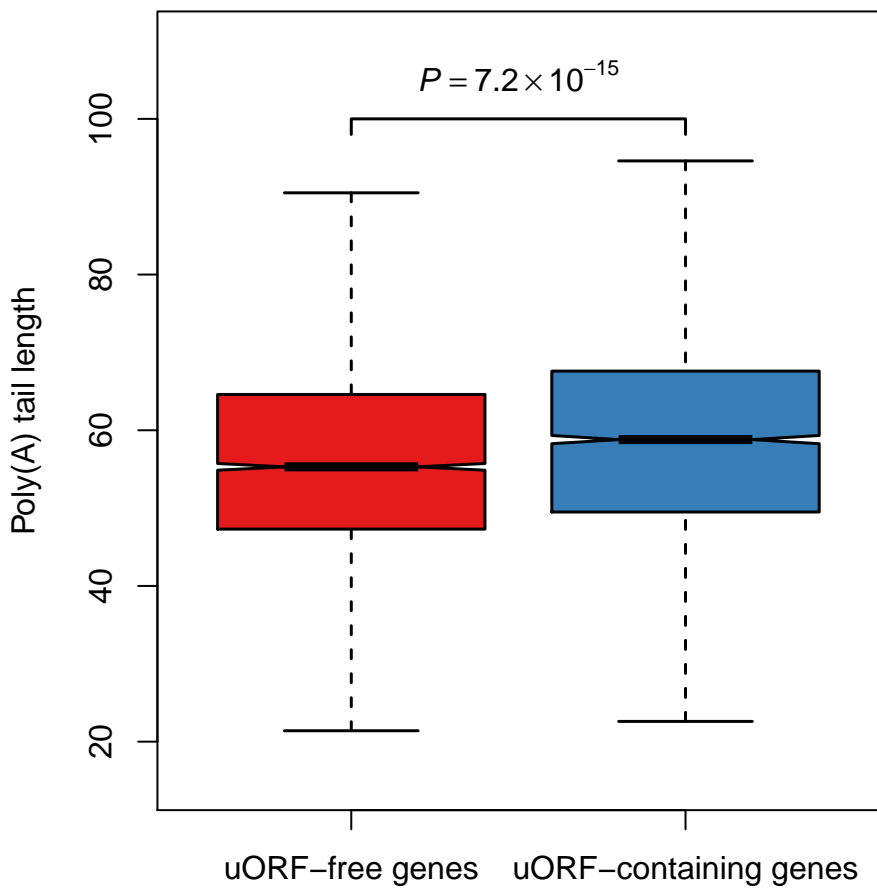

Supplement: S26 Fig — The distribution of poly(A)-tail lengths of expressed genes (mRNA RPKM ≥ 1) without ribosome-associated uORFs (left) or with ribosome-associated uORFs (right) in 0–1 h embryos of D. melanogaster. Differences in poly(A)-tail lengths were compared with t test. Data of poly(A)-tail lengths were from a previous study [118]. The raw data can be found in S1 Data. CDS, coding DNA sequence; RPKM, reads per kilobase of transcript per million mapped reads; TE, translational efficiency; uORF, upstream open reading frame. (PDF) [file pbio.2003903.s043.pdf]

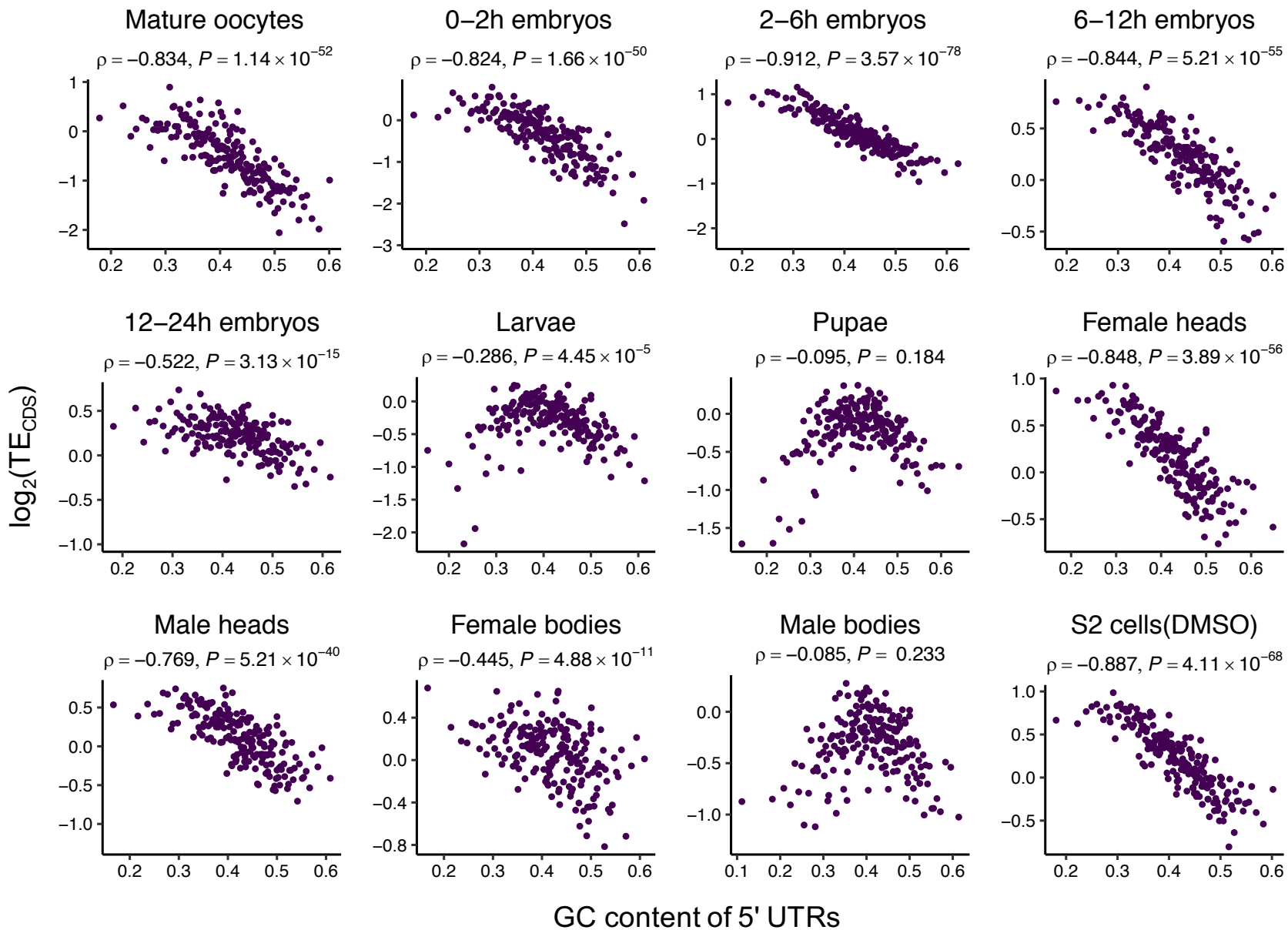

Supplement: S28 Fig — Genes were grouped into 200 bins of equal size based on increasing GC content of 5′ UTRs. The median GC content of 5′ UTR and log2(TE) in each bin were displayed in the plots. The raw data can be found in S4 Data. CDS, coding DNA sequence; RPKM, reads per kilobase of transcript per million mapped reads; TE, translational efficiency; UTR, untranslated region. (PDF) [file pbio.2003903.s045.pdf]

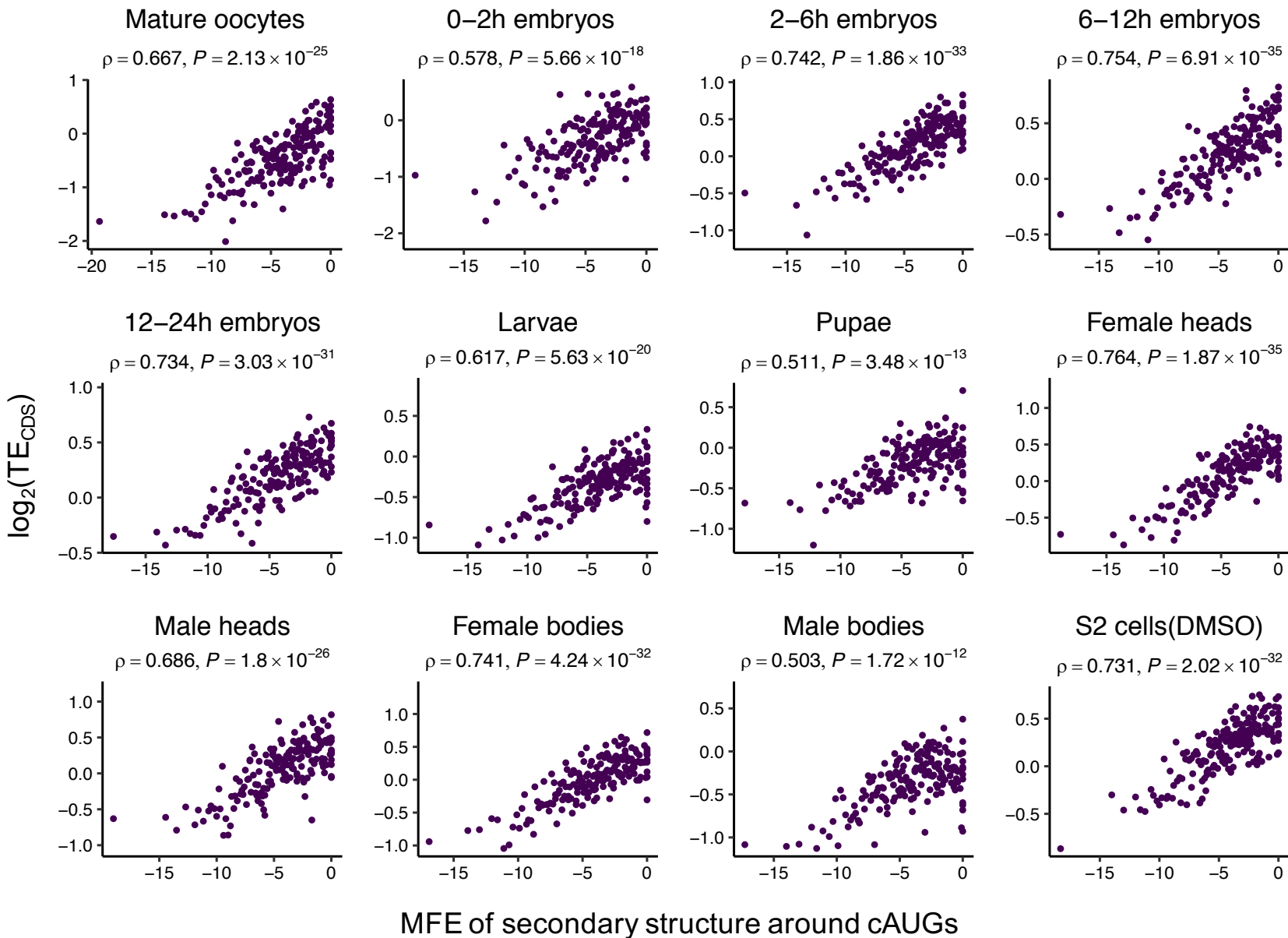

Supplement: S30 Fig — Genes were grouped into 200 bins of equal size based on increasing MFE. Median MFE and log2(TE) in each bin were displayed in the plots. The raw data can be found in S4 Data. cAUG, AUG start codon of CDS; CDS, coding DNA sequence; MFE, minimum free energy; RPKM, reads per kilobase of transcript per million mapped reads; TE, translational efficiency; UTR, untranslated region. (PDF) [file pbio.2003903.s047.pdf]

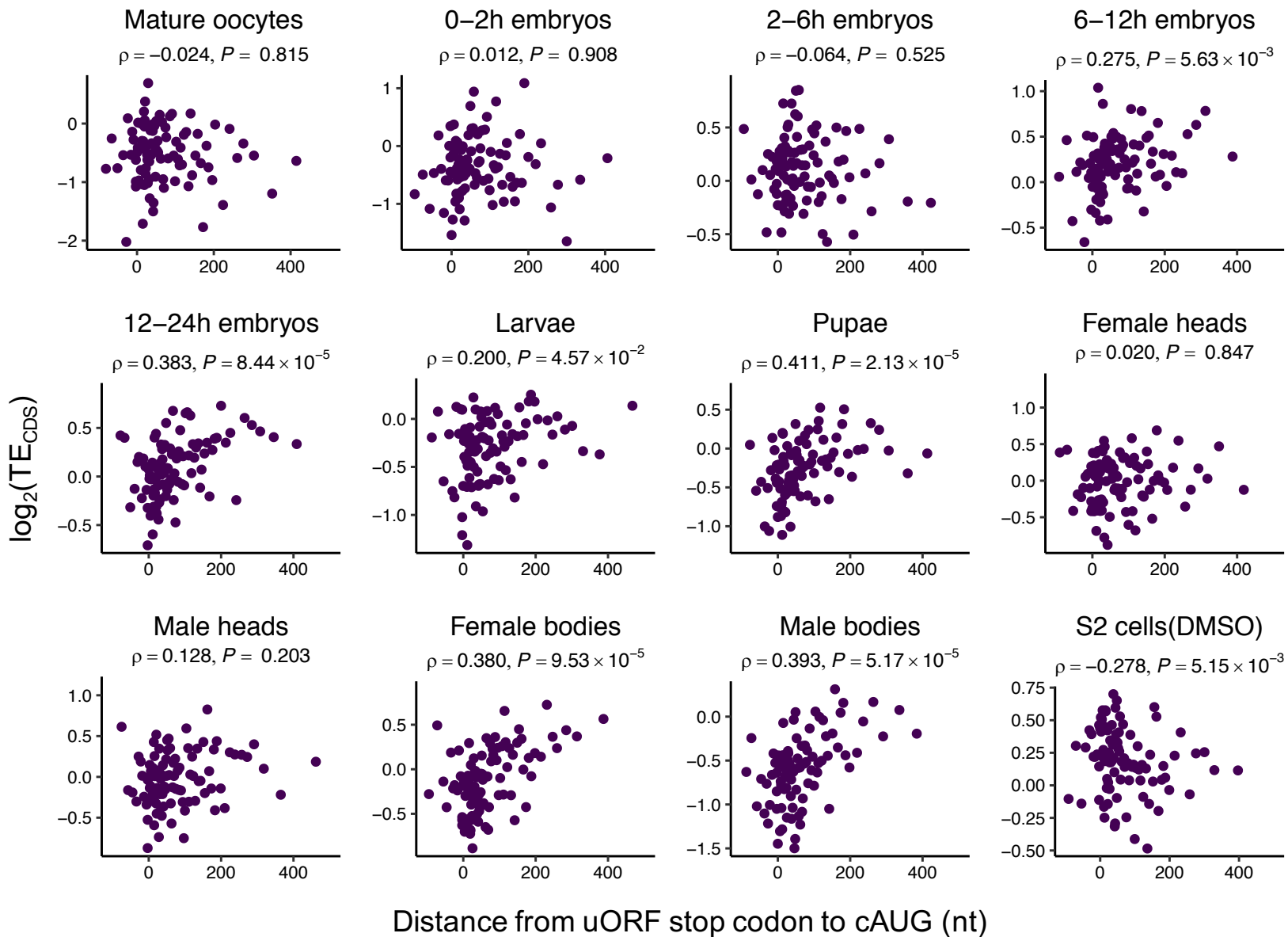

Supplement: S36 Fig — Genes were grouped into 50 bins based on distances from uORF stop codon to cAUG. Median distance from uORF stop codon to cAUG and log2(TE) in each bin were displayed in the plots. The raw data can be found in S1 Data. cAUG, AUG start codon of CDS; CDS, coding DNA sequence; RPKM, reads per kilobase of transcript per million mapped reads; TE, translational efficiency; uORF, upstream open reading frame. (PDF) [file pbio.2003903.s053.pdf]

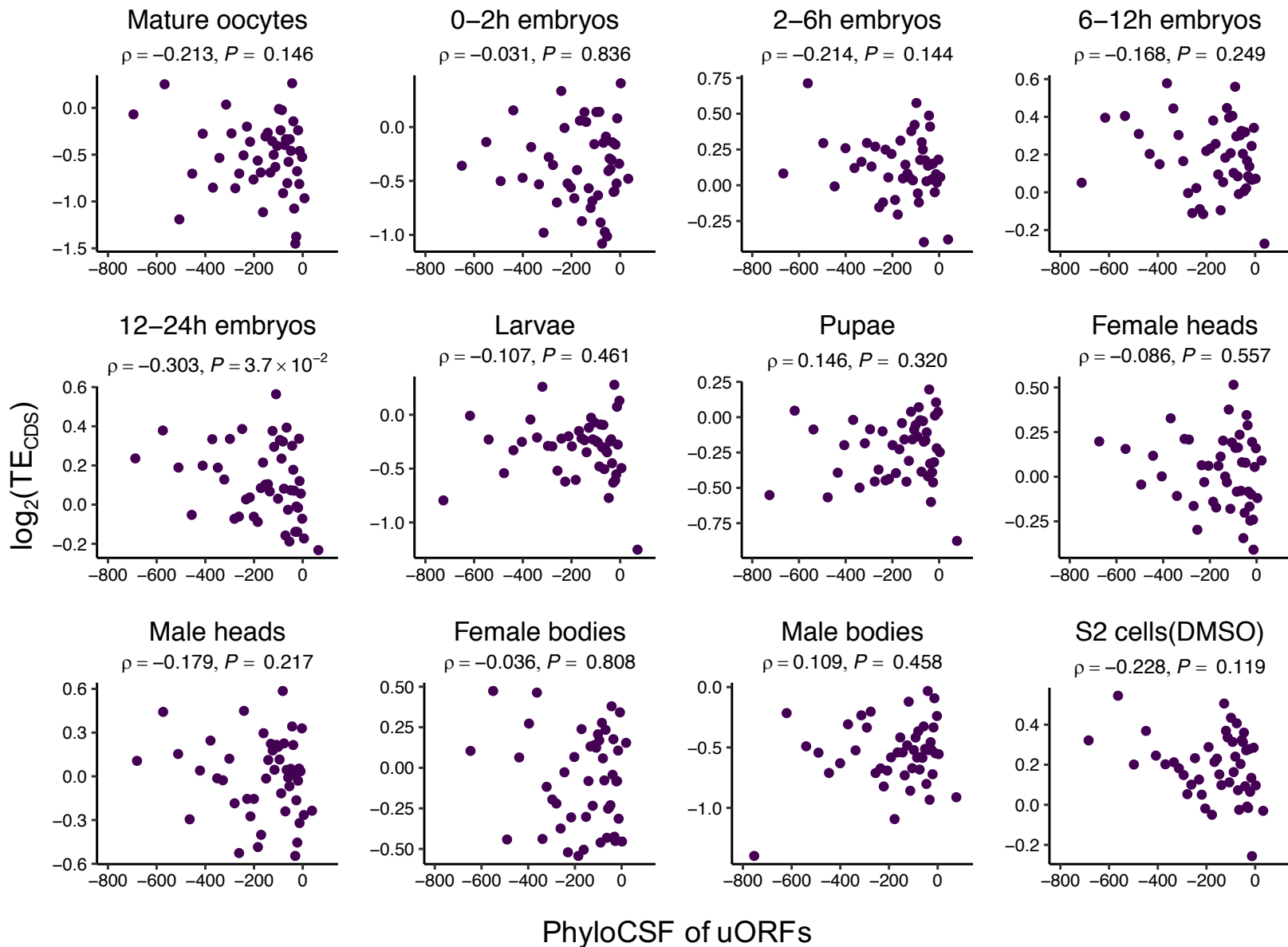

Supplement: S38 Fig — Genes were grouped into 50 bins based on increasing phyloCSF. Median phyloCSF and log2(TE) in each bin were displayed in the plots. The raw data can be found in S1 Data. The raw data can be found in S1 Data. CDS, coding DNA sequence; RPKM, reads per kilobase of transcript per million mapped reads; TE, translational efficiency; uORF, upstream open reading frame. (PDF) [file pbio.2003903.s055.pdf]

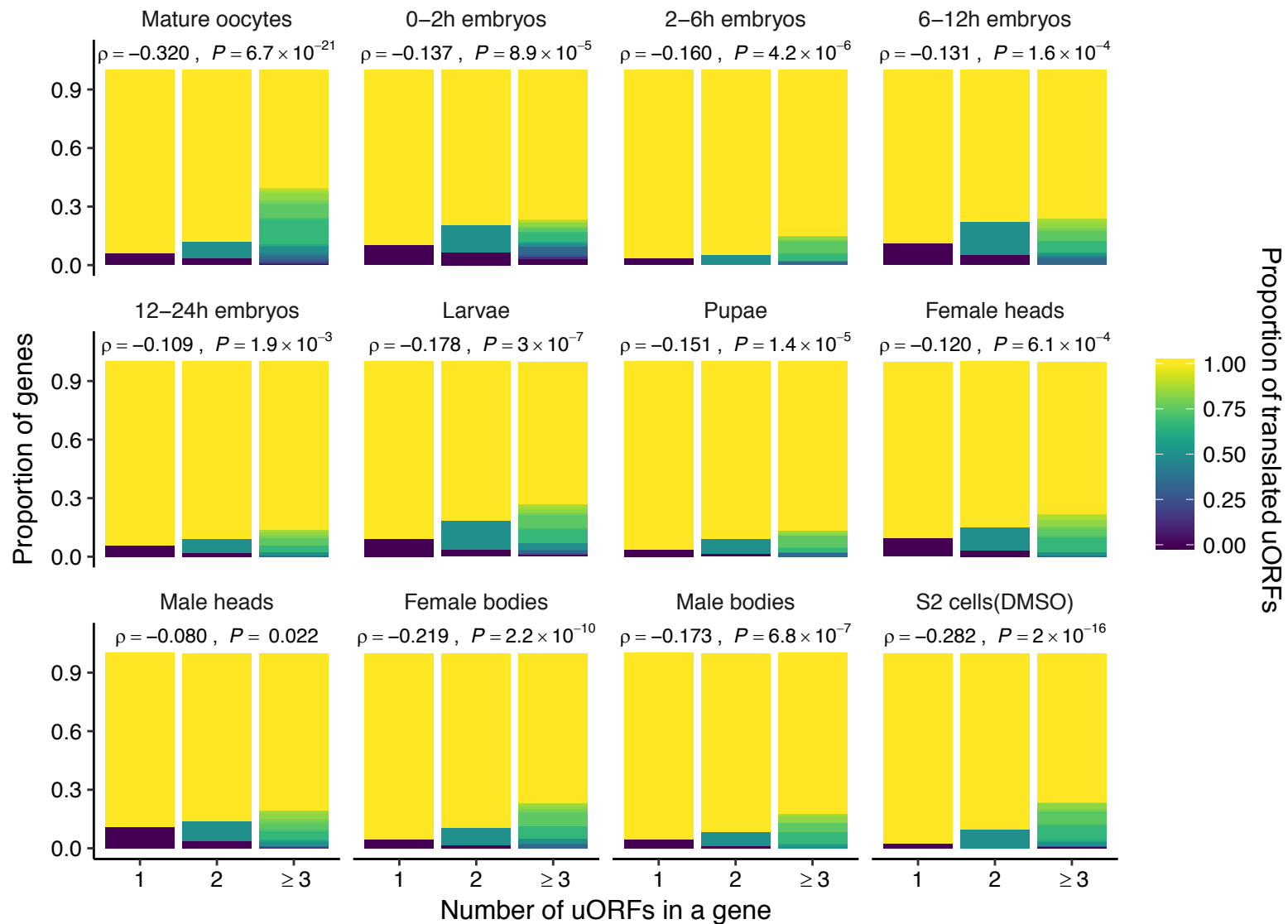

Supplement: S42 Fig — Genes with at least 3 uORFs are grouped together for visualization. Spearman’s correlations between the number of uORFs in a gene and the proportion of translated uORFs are shown below the sample names. RPKM, reads per kilobase of transcript per million mapped reads; TE, translational efficiency; uORF, upstream open reading frame. (PDF) [file pbio.2003903.s059.pdf]

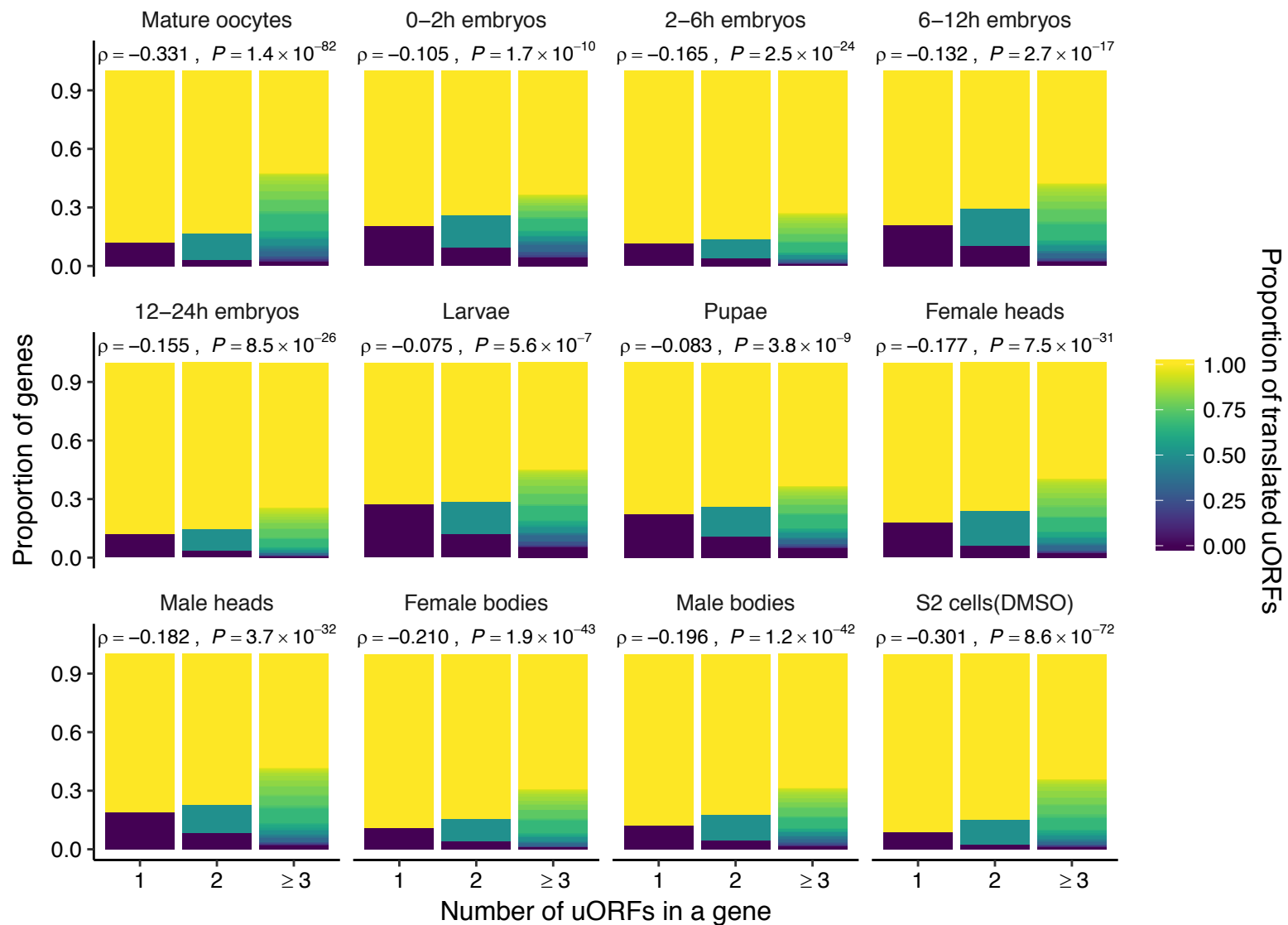

Supplement: S43 Fig — Genes with at least 3 uORFs are grouped together for visualization. Spearman’s correlations between the number of uORFs in a gene and the proportion of translated uORFs are shown below the sample names. RPKM, reads per kilobase of transcript per million mapped reads; TE, translational efficiency; uORF, upstream open reading frame. (PDF) [file pbio.2003903.s060.pdf]

*dichaete*

mRNA-Seq

Ribo-Seq

 $\log_2(\text{TE}_{\text{CDS}})$ 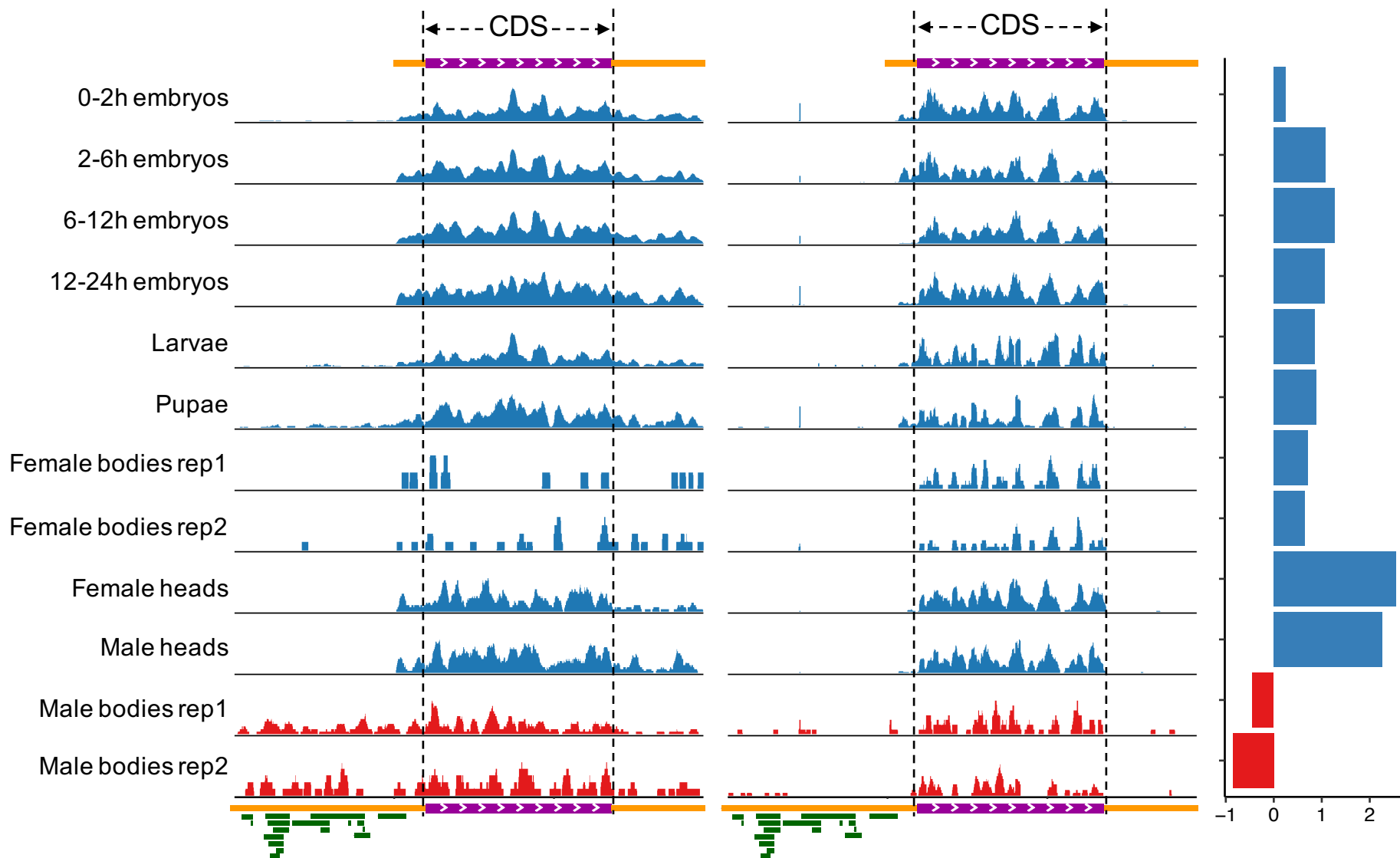

Supplement: S44 Fig — The dominant isoforms, the profiles of the mRNA-Seq (left) and Ribo-Seq (middle) data, and log2(TE) (right) of D. melanogaster dichaete in different developmental stages or tissues. The CDS and UTR region in the gene model are in purple and orange, respectively. The CDS region of dichaete is also delineated with dashed lines. The short uORF-free isoform of dichaete (top) is predominately expressed in all samples except bodies of male adults, while a long isoform with many uORFs (dark green) is predominately expressed in bodies of male adults. Accordingly, log2(TE) of dichaete is much lower in bodies of male adults. The sequencing data are available from SRA under accession SRP067542 and rep1 and rep2 represent 2 biological replicates. CDS, coding DNA sequence; SRA, Sequence Read Archive; TE, translational efficiency; uORF, upstream open reading frame; UTR, untranslated region. (PDF) [file pbio.2003903.s061.pdf]

*glycerol kinase 2*

mRNA-Seq

Ribo-Seq

$\log_2(\text{TE}_{\text{CDS}})$

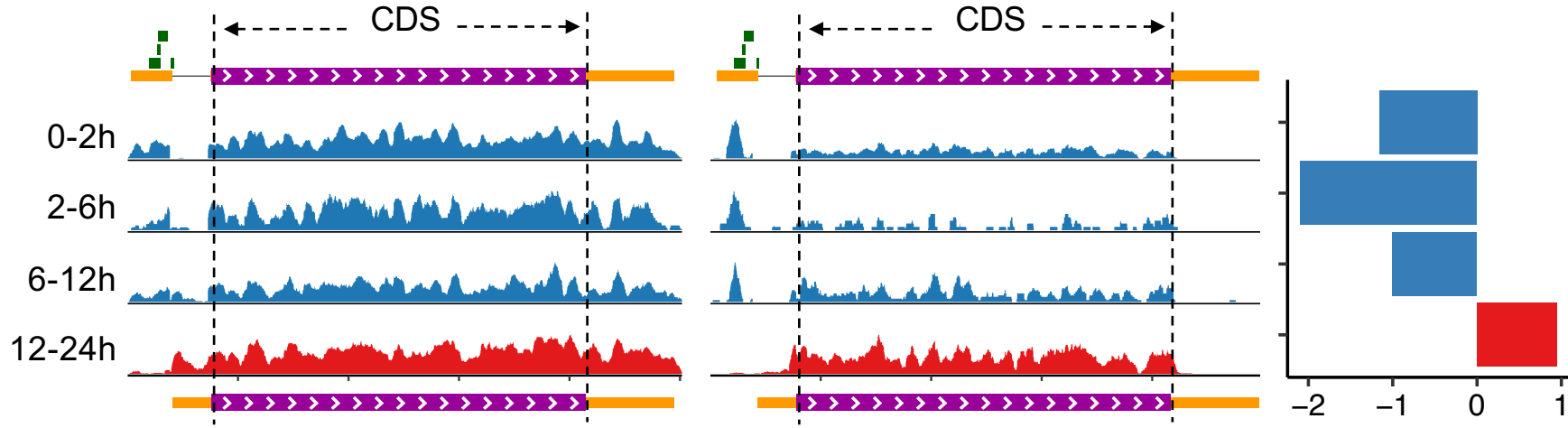

Supplement: S45 Fig — The dominant isoforms, the profiles of the mRNA-Seq (left) and Ribo-Seq (middle) data, and log2(TE) (right) of glycerol kinase 2 in different embryonic stages of D. melanogaster. The CDS and UTR region in the gene model are in purple and orange, respectively. The CDS region of glycerol kinase 2 is also delineated with dashed lines. In the fly embryo, an isoform containing 4 uORFs is predominately expressed during 0–12 h, while another isoform without uORFs predominates during 12–24 h, which might be related to the increased translation of glycerol kinase 2 at this stage. The sequencing data are available from SRA under accession SRP067542. CDS, coding DNA sequence; TE, translational efficiency; uORF, upstream open reading frame; UTR, untranslated region. (PDF) [file pbio.2003903.s062.pdf]

Coverage

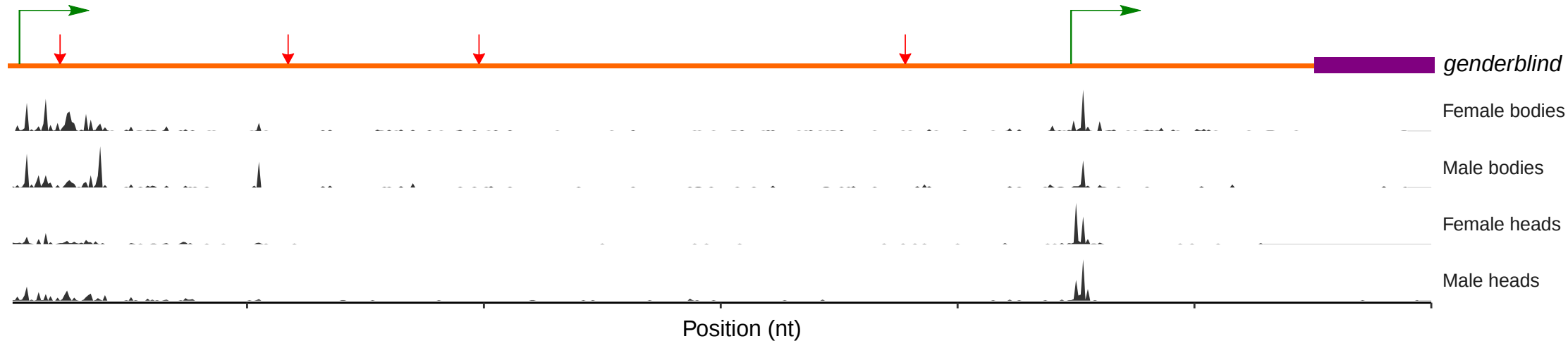

Supplement: S46 Fig — For each sample, the corresponding CAGE samples of modENCODE were pooled together. The coverage at each site was calculated as the number of 5′ ends of CAGE tags at this site. The TSSs annotated by FlyBase and uAUGs were displayed with green arrows and red arrows, respectively. The CDS region was displayed in purple. CAGE, cap analysis of gene expression; CDS, coding DNA sequence; TSS, transcription start site; uAUG, start codon of upstream open reading frame. (PDF) [file pbio.2003903.s063.pdf]

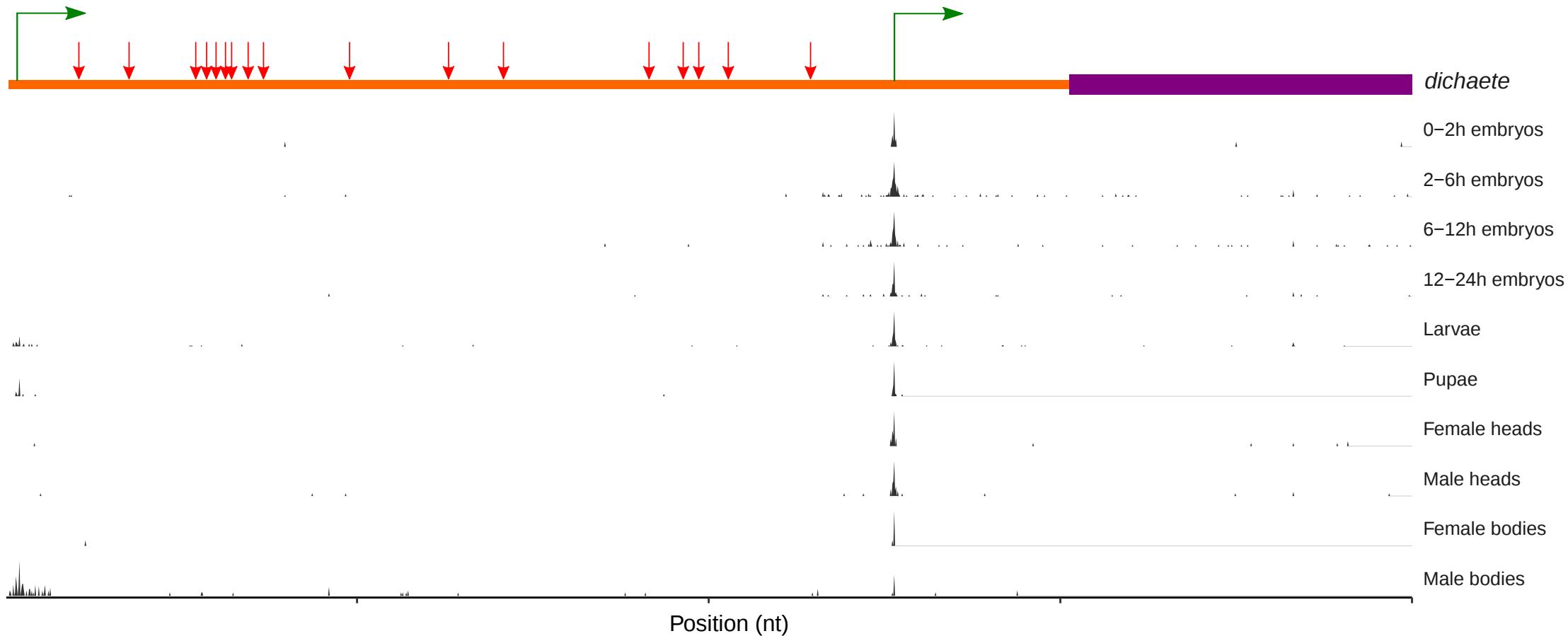

Supplement: S47 Fig — For each sample, the corresponding CAGE samples of modENCODE were pooled together. The coverage at each site was calculated as the number of 5′ ends of CAGE tags at this site. The TSSs annotated by FlyBase and uAUGs were displayed with green arrows and red arrows, respectively. The CDS region was displayed in purple. CAGE, cap analysis of gene expression; CDS, coding DNA sequence; TSS, transcription start site; uAUG, start codon of upstream open reading frame. (PDF) [file pbio.2003903.s064.pdf]

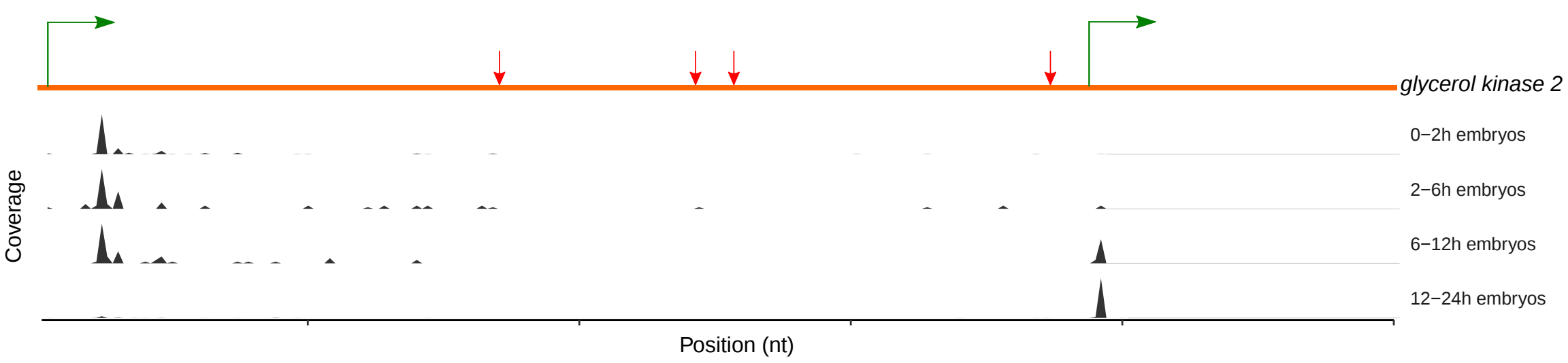

Supplement: S48 Fig — For each sample, the corresponding CAGE samples of modENCODE were pooled together. The coverage at each site was calculated as the number of 5′ ends of CAGE tags at this site. The TSSs annotated by FlyBase and uAUGs were displayed with green arrows and red arrows, respectively. CAGE, cap analysis of gene expression; CDS, coding DNA sequence; TSS, transcription start site; uAUG, start codon of upstream open reading frame. (PDF) [file pbio.2003903.s065.pdf]

Density

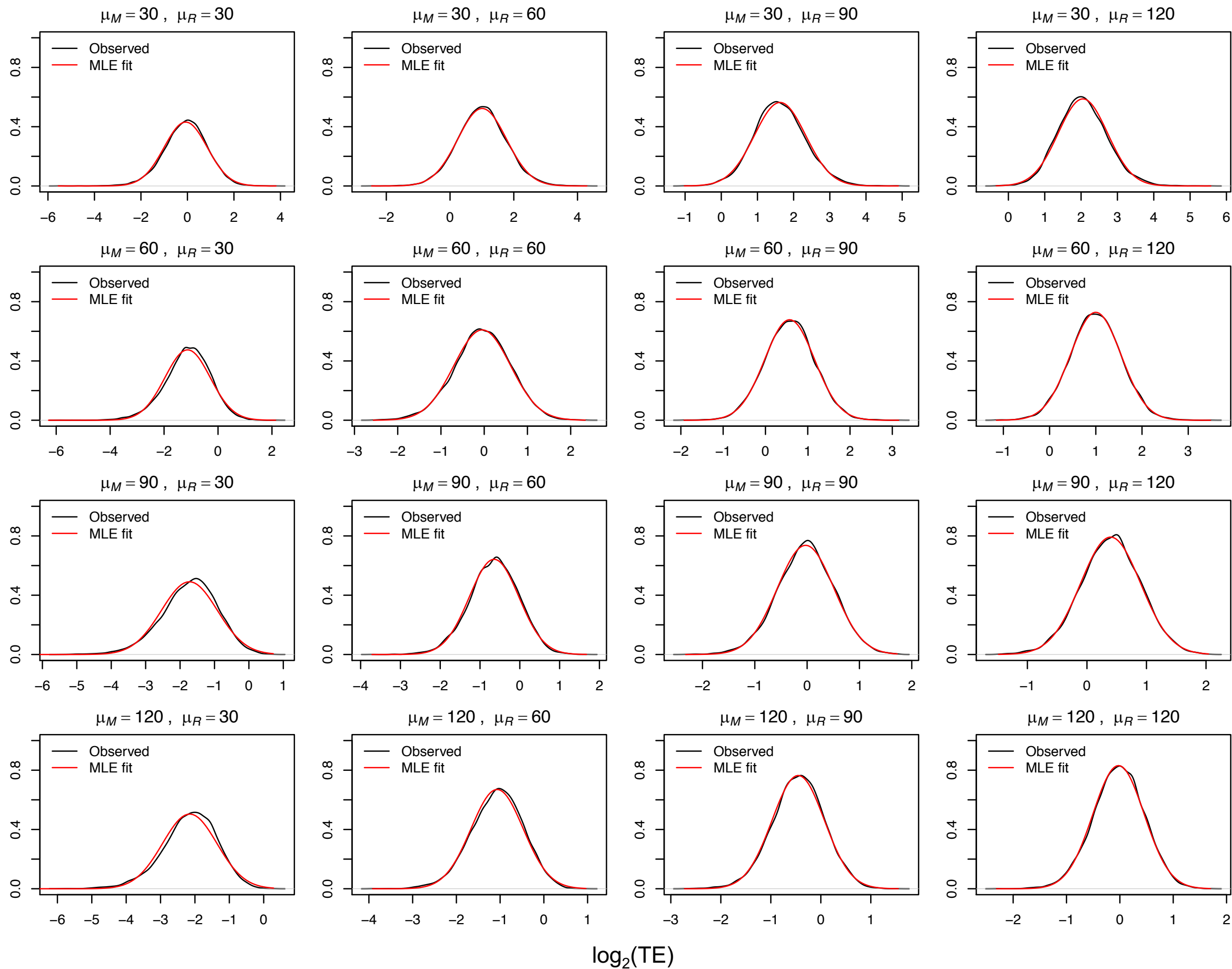

Supplement: S49 Fig — Under given expected mRNA count μM and RPF count μR, the corresponding dispersion parameters were estimated with the overall dispersion trend of mRNA or RPF counts obtained with biological replicates of female and male bodies. Then, 10,000 mRNA counts and RPF counts were simulated with these parameters, and log2(TE) values were calculated with simulated counts. The observed distribution of log2(TE) was denoted with black line. The normal distribution fitted with maximum likelihood method was shown in red. RPF, ribosome-protected mRNA fragment; TE, translational efficiency. (PDF) [file pbio.2003903.s066.pdf]
