# Supplementary material for: Genome-wide maps of ribosomal occupancy provide insights into adaptive evolution and regulatory roles of uORFs during Drosophila development
Source: PLoS Biol. 2018 Jul 20;16(7):e2003903. doi: 10.1371/journal.pbio.2003903 (PMC6070289; doi:10.1371/journal.pbio.2003903)
Supplement: S10 Fig — For a uORF i and its downstream CDS i in a sample, we assumed log2(TEuORF,i) and log2(TECDS,i) follow normal distributions. For female bodies and male bodies, we estimated the SE values of log2(TE) of a feature (CDS or uORF) by contrasting the RPF counts against the mRNA counts of biological replicates with DESeq2 [66]. Then, we fitted a smooth surface of SE of log2(TE) against the mRNA counts and log2(TE) of features. For a sample without biological replicates, the SE value of log2(TE) for each feature is inferred from the smooth surface obtained with biological replicates of female and male bodies. Then, we denoted TEuORF,i/TECDS,i as βi and estimated the SE value of log2(βi). The P value under the null hypothesis log2(βi) = 0 was obtained with a two-tailed Wald test. The raw data can be found in S7 Data. CDS, coding DNA sequence; RFP, ribosome-protected mRNA fragment; SE, standard error; TE, translational efficiency; uORF, upstream open reading frame. (PDF) [file pbio.2003903.s027.pdf]

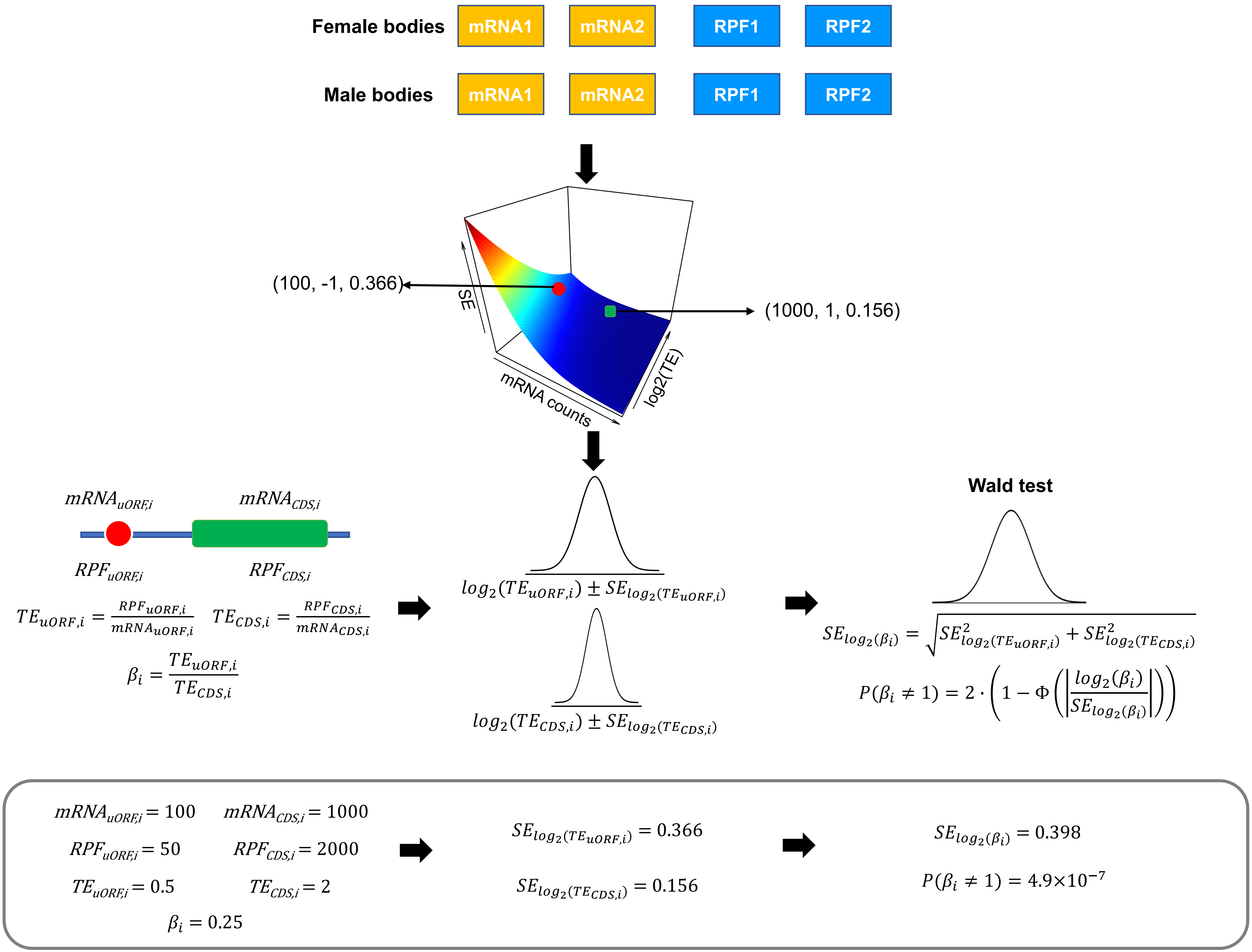
Female bodies
mRNA1
mRNA2
RPF1
RPF2
Male bodies
mRNA1
mRNA2
RPF1
RPF2
(100, -1, 0.366)
(1000, 1, 0.156)
SE
mRNA counts
log2(TE)
$mRNA_{uORF,i}$
$mRNA_{CDS,i}$
$RPF_{uORF,i}$
$RPF_{CDS,i}$
$TE_{uORF,i} = \frac{RPF_{uORF,i}}{mRNA_{uORF,i}}$
$TE_{CDS,i} = \frac{RPF_{CDS,i}}{mRNA_{CDS,i}}$
$\beta_i = \frac{TE_{uORF,i}}{TE_{CDS,i}}$
$log_2(TE_{uORF,i}) \pm SE_{log_2(TE_{uORF,i})}$
$log_2(TE_{CDS,i}) \pm SE_{log_2(TE_{CDS,i})}$
Wald test
$SE_{log_2(\beta_i)} = \sqrt{SE^2_{log_2(TE_{uORF,i})} + SE^2_{log_2(TE_{CDS,i})}}$
$P(\beta_i \neq 1) = 2 \cdot \left(1 - \Phi\left(\left|\frac{log_2(\beta_i)}{SE_{log_2(\beta_i)}}\right|\right)\right)$
$mRNA_{uORF,i} = 100$
$mRNA_{CDS,i} = 1000$
$RPF_{uORF,i} = 50$
$RPF_{CDS,i} = 2000$
$TE_{uORF,i} = 0.5$
$TE_{CDS,i} = 2$
$\beta_i = 0.25$
$SE_{log_2(TE_{uORF,i})} = 0.366$
$SE_{log_2(TE_{CDS,i})} = 0.156$
$SE_{log_2(\beta_i)} = 0.398$
$P(\beta_i \neq 1) = 4.9\times10^{-7}$
