# Supplementary material for: Genome-wide maps of ribosomal occupancy provide insights into adaptive evolution and regulatory roles of uORFs during Drosophila development
Source: PLoS Biol. 2018 Jul 20;16(7):e2003903. doi: 10.1371/journal.pbio.2003903 (PMC6070289; doi:10.1371/journal.pbio.2003903)
Supplement: S13 Fig — For a uORF i that is expressed with Kim normalized mRNA reads but not covered by any RPF in a sample m, we assumed the expected TE of this uORF (x) is the same as that of the downstream CDS (TECDS,i) under the null hypothesis H0(c). Then, we estimated the prior distribution of x with the smooth surface of SE values of log2(TE) against the mRNA counts and log2(TE) of well-transcribed features in biological replicates of female and male bodies. The prior distribution of Kim was also estimated with the dispersion trend of mRNA counts in biological replicates of female and male bodies. By modeling the RPF count of the uORF using a negative binominal distribution with mean xKim at given x and Kim, we can derive the posterior probability of observing 0 RPF reads Pm(R0) under null hypothesis H0(c). CDS, coding DNA sequence; RPF, ribosome-protected mRNA fragment; SE, standard error; TE, translational efficiency; uORF, upstream open reading frame. (PDF) [file pbio.2003903.s030.pdf]

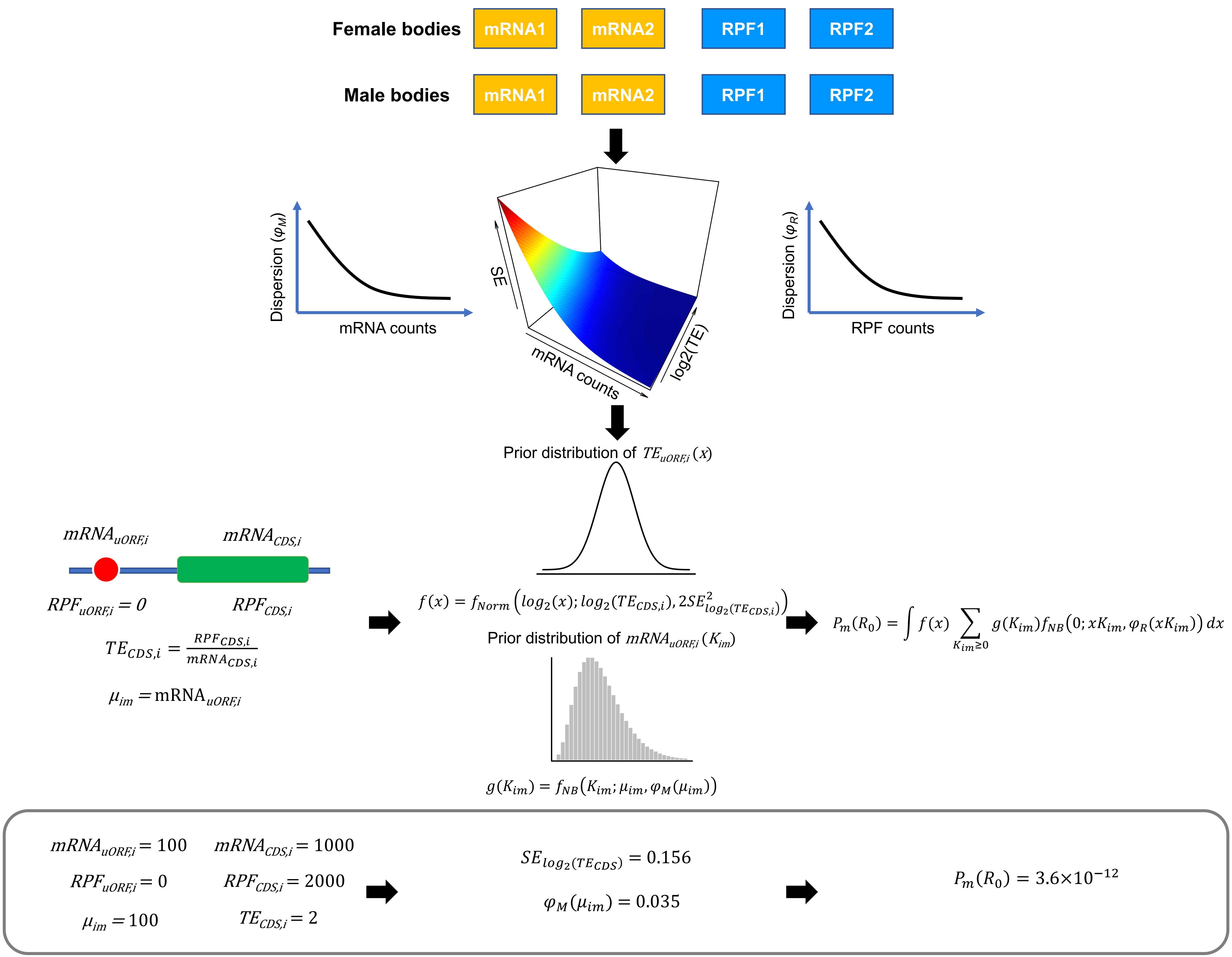

Female bodies
mRNA1
mRNA2
RPF1
RPF2
Male bodies
mRNA1
mRNA2
RPF1
RPF2
Dispersion ($\varphi_M$)
mRNA counts
SE
mRNA counts
log2(TE)
Dispersion ($\varphi_R$)
RPF counts
Prior distribution of $TE_{uORF,i}\,(x)$
$mRNA_{uORF,i}$
$mRNA_{CDS,i}$
$RPF_{uORF,i} = 0$
$RPF_{CDS,i}$
$TE_{CDS,i} = \frac{RPF_{CDS,i}}{mRNA_{CDS,i}}$
$\mu_{im} = \mathrm{mRNA}_{uORF,i}$
$f(x) = f_{Norm}\left(log_2(x); log_2(TE_{CDS,i}), 2SE^2_{log_2(TE_{CDS,i})}\right)$
Prior distribution of $mRNA_{uORF,i}\,(K_{im})$
$g(K_{im}) = f_{NB}\left(K_{im}; \mu_{im}, \varphi_M(\mu_{im})\right)$
$P_m(R_0) = \int f(x) \sum_{K_{im} \geq 0} g(K_{im}) f_{NB}\left(0; xK_{im}, \varphi_R(xK_{im})\right) dx$
$mRNA_{uORF,i} = 100$
$mRNA_{CDS,i} = 1000$
$RPF_{uORF,i} = 0$
$RPF_{CDS,i} = 2000$
$\mu_{im} = 100$
$TE_{CDS,i} = 2$
$SE_{log_2(TE_{CDS})} = 0.156$
$\varphi_M(\mu_{im}) = 0.035$
$P_m(R_0) = 3.6\times10^{-12}$
