# Supplementary material for: Genome-wide maps of ribosomal occupancy provide insights into adaptive evolution and regulatory roles of uORFs during Drosophila development
Source: PLoS Biol. 2018 Jul 20;16(7):e2003903. doi: 10.1371/journal.pbio.2003903 (PMC6070289; doi:10.1371/journal.pbio.2003903)
Supplement: S27 Fig — Genes were grouped into 200 bins of equal size based on increasing length of 5′ UTRs. Median log10(5′ UTR length) and log2(TE) in each bin were displayed in the plots. A pseudo-count of 1 was added to 5′ UTR length before logarithmic transformation. The raw data can be found in S4 Data. CDS, coding DNA sequence; RPKM, reads per kilobase of transcript per million mapped reads; TE, translational efficiency; UTR, untranslated region. (PDF) [file pbio.2003903.s044.pdf]

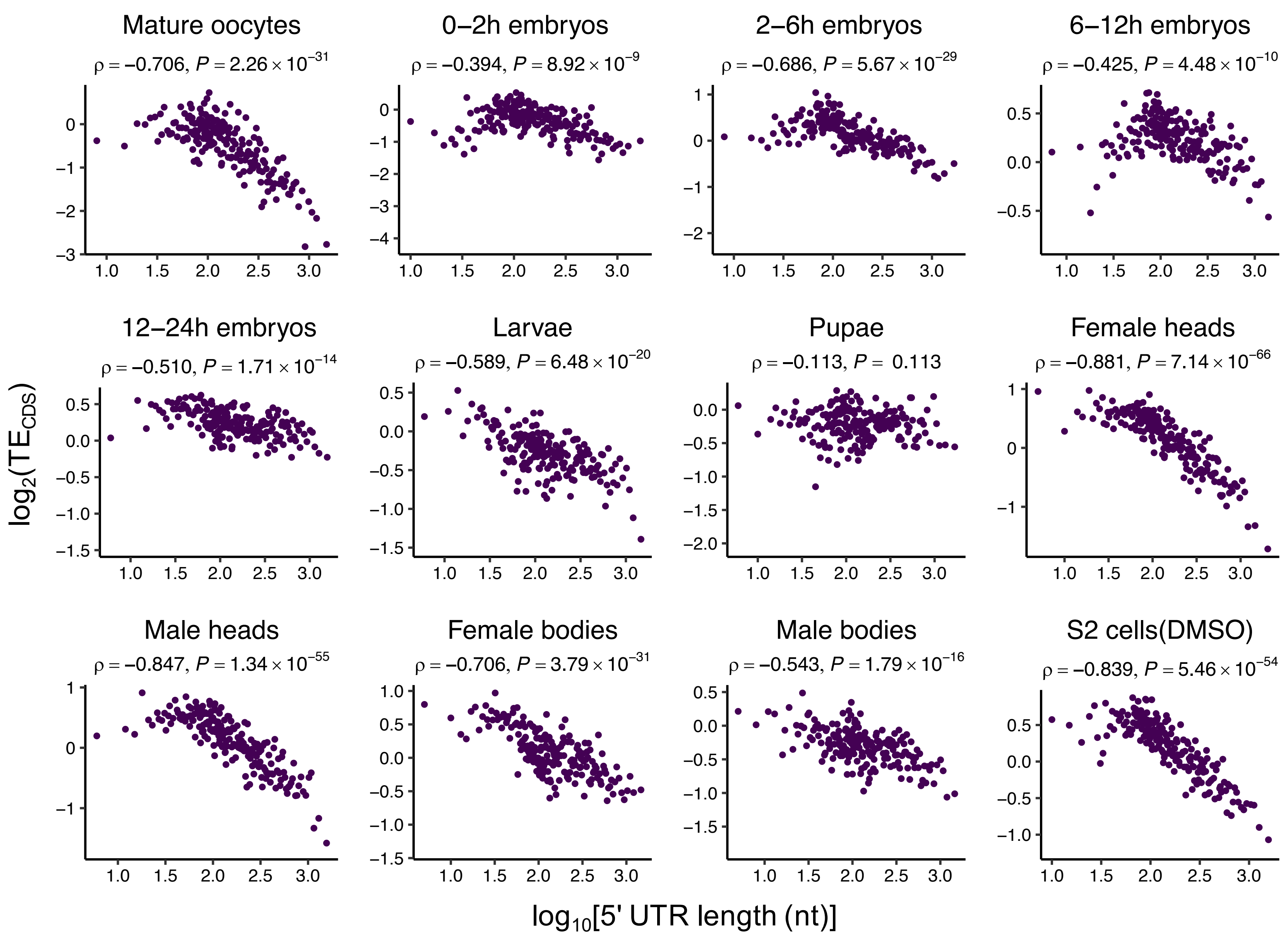
Mature oocytes
ρ = −0.706, P = 2.26 × 10^−31
0–2h embryos
ρ = −0.394, P = 8.92 × 10^−9
2–6h embryos
ρ = −0.686, P = 5.67 × 10^−29
6–12h embryos
ρ = −0.425, P = 4.48 × 10^−10
12–24h embryos
ρ = −0.510, P = 1.71 × 10^−14
Larvae
ρ = −0.589, P = 6.48 × 10^−20
Pupae
ρ = −0.113, P = 0.113
Female heads
ρ = −0.881, P = 7.14 × 10^−66
Male heads
ρ = −0.847, P = 1.34 × 10^−55
Female bodies
ρ = −0.706, P = 3.79 × 10^−31
Male bodies
ρ = −0.543, P = 1.79 × 10^−16
S2 cells(DMSO)
ρ = −0.839, P = 5.46 × 10^−54
log2(TECDS)
log10[5' UTR length (nt)]
