# Supplementary material for: Genome-wide maps of ribosomal occupancy provide insights into adaptive evolution and regulatory roles of uORFs during Drosophila development
Source: PLoS Biol. 2018 Jul 20;16(7):e2003903. doi: 10.1371/journal.pbio.2003903 (PMC6070289; doi:10.1371/journal.pbio.2003903)
Supplement: S29 Fig — Genes were grouped into 200 bins of equal size based on increasing Kozak score. Median Kozak score and log2(TE) in each bin were displayed in the plots. The raw data can be found in S4 Data. CDS, coding DNA sequence; RPKM, reads per kilobase of transcript per million mapped reads; TE, translational efficiency; UTR, untranslated region. (PDF) [file pbio.2003903.s046.pdf]

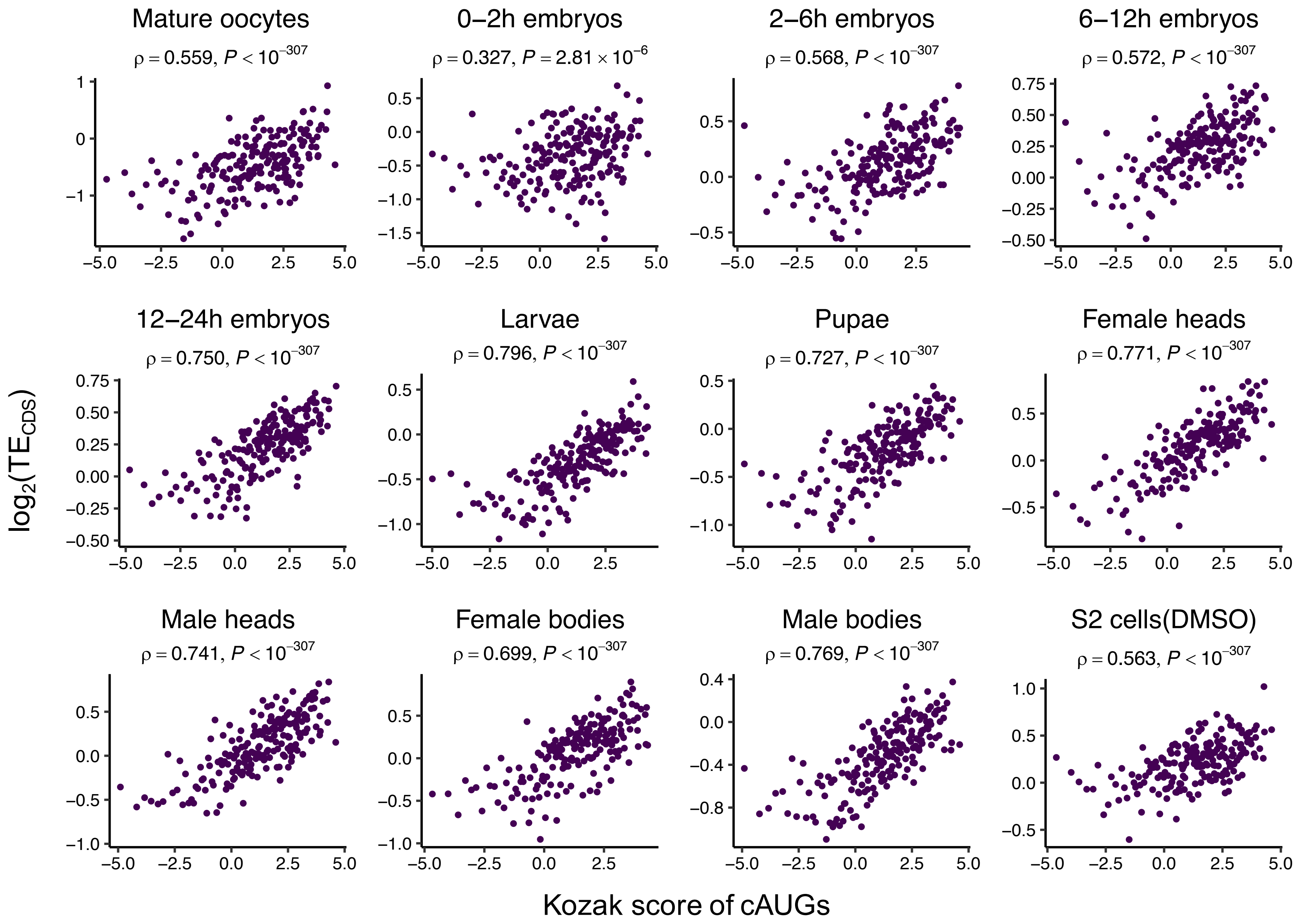

Mature oocytes
ρ = 0.559, P < 10^−307
0–2h embryos
ρ = 0.327, P = 2.81 × 10^−6
2–6h embryos
ρ = 0.568, P < 10^−307
6–12h embryos
ρ = 0.572, P < 10^−307
12–24h embryos
ρ = 0.750, P < 10^−307
Larvae
ρ = 0.796, P < 10^−307
Pupae
ρ = 0.727, P < 10^−307
Female heads
ρ = 0.771, P < 10^−307
Male heads
ρ = 0.741, P < 10^−307
Female bodies
ρ = 0.699, P < 10^−307
Male bodies
ρ = 0.769, P < 10^−307
S2 cells(DMSO)
ρ = 0.563, P < 10^−307
log2(TECDS)
Kozak score of cAUGs
