# Supplementary material for: Genome-wide maps of ribosomal occupancy provide insights into adaptive evolution and regulatory roles of uORFs during Drosophila development
Source: PLoS Biol. 2018 Jul 20;16(7):e2003903. doi: 10.1371/journal.pbio.2003903 (PMC6070289; doi:10.1371/journal.pbio.2003903)
Supplement: S31 Fig — Genes were grouped into 200 bins of equal size based on MFE. Median MFE and log2(TE) in each bin were displayed in the plots. The raw data can be found in S4 Data. CDS, coding DNA sequence; MFE, minimum free energy; RPKM, reads per kilobase of transcript per million mapped reads; TE, translational efficiency; UTR, untranslated region. (PDF) [file pbio.2003903.s048.pdf]

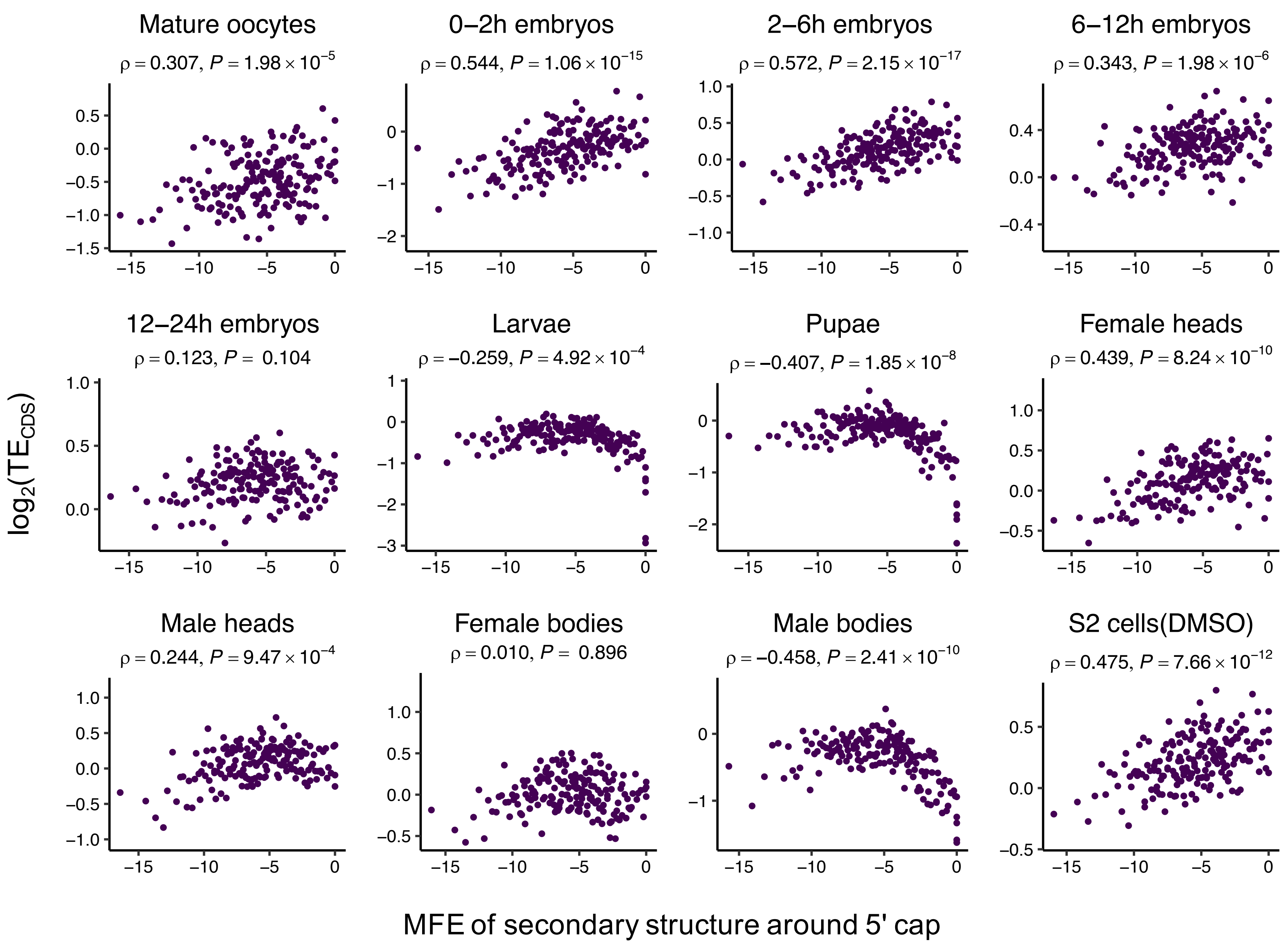

Mature oocytes
ρ = 0.307, P = 1.98 × 10^−5
0–2h embryos
ρ = 0.544, P = 1.06 × 10^−15
2–6h embryos
ρ = 0.572, P = 2.15 × 10^−17
6–12h embryos
ρ = 0.343, P = 1.98 × 10^−6
12–24h embryos
ρ = 0.123, P = 0.104
Larvae
ρ = −0.259, P = 4.92 × 10^−4
Pupae
ρ = −0.407, P = 1.85 × 10^−8
Female heads
ρ = 0.439, P = 8.24 × 10^−10
Male heads
ρ = 0.244, P = 9.47 × 10^−4
Female bodies
ρ = 0.010, P = 0.896
Male bodies
ρ = −0.458, P = 2.41 × 10^−10
S2 cells(DMSO)
ρ = 0.475, P = 7.66 × 10^−12
log2(TECDS)
MFE of secondary structure around 5' cap
