# Supplementary material for: Genome-wide maps of ribosomal occupancy provide insights into adaptive evolution and regulatory roles of uORFs during Drosophila development
Source: PLoS Biol. 2018 Jul 20;16(7):e2003903. doi: 10.1371/journal.pbio.2003903 (PMC6070289; doi:10.1371/journal.pbio.2003903)
Supplement: S32 Fig — The raw data can be found in S4 Data. CDS, coding DNA sequence; RPKM, reads per kilobase of transcript per million mapped reads; TE, translational efficiency. (PDF) [file pbio.2003903.s049.pdf]

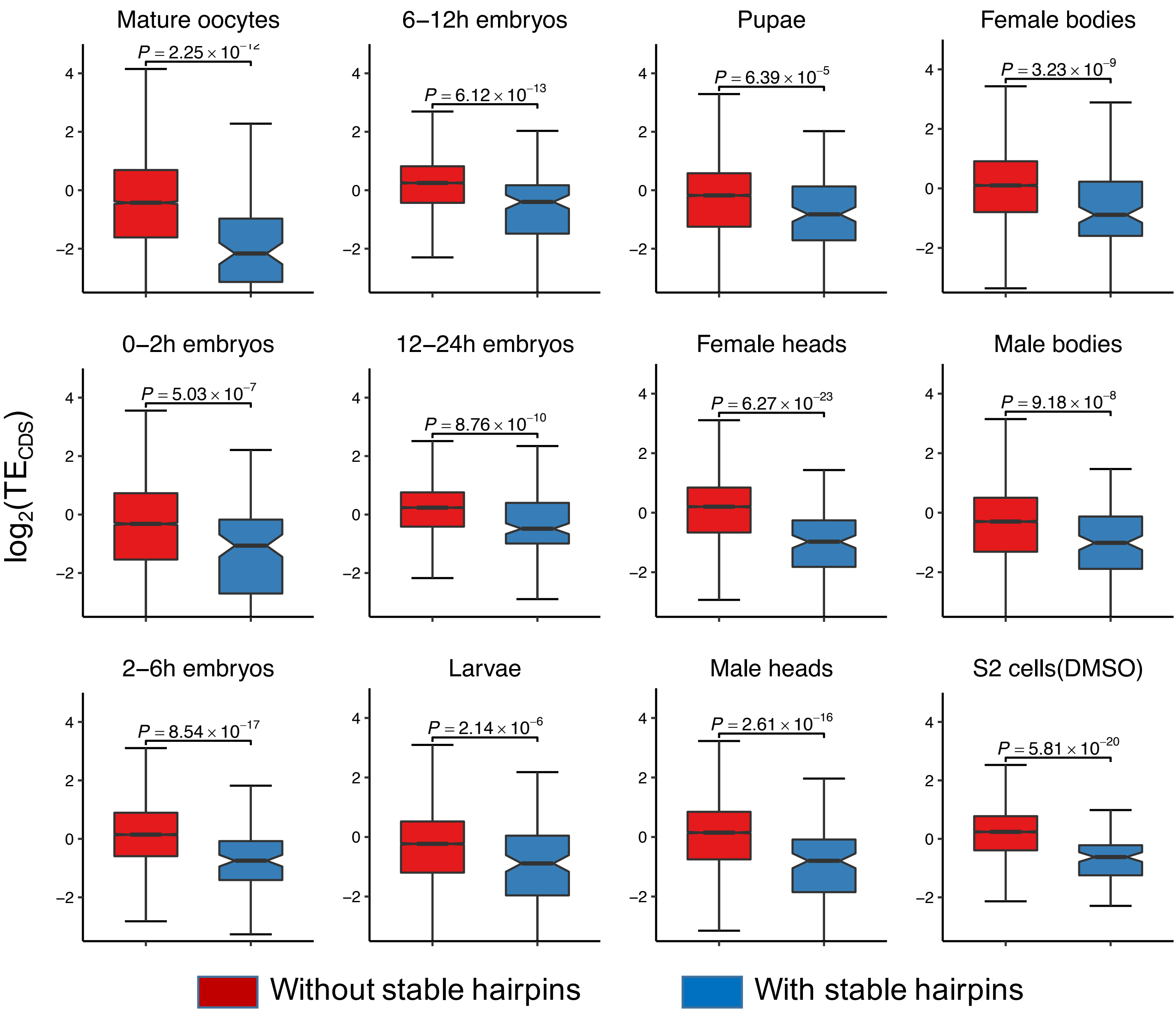

Mature oocytes
P = 2.25 × 10^−12
6–12h embryos
P = 6.12 × 10^−13
Pupae
P = 6.39 × 10^−5
Female bodies
P = 3.23 × 10^−9
0–2h embryos
P = 5.03 × 10^−7
12–24h embryos
P = 8.76 × 10^−10
Female heads
P = 6.27 × 10^−23
Male bodies
P = 9.18 × 10^−8
2–6h embryos
P = 8.54 × 10^−17
Larvae
P = 2.14 × 10^−6
Male heads
P = 2.61 × 10^−16
S2 cells(DMSO)
P = 5.81 × 10^−20
log2(TECDS)
4
2
0
−2
Without stable hairpins
With stable hairpins
