# Supplementary material for: Genome-wide maps of ribosomal occupancy provide insights into adaptive evolution and regulatory roles of uORFs during Drosophila development
Source: PLoS Biol. 2018 Jul 20;16(7):e2003903. doi: 10.1371/journal.pbio.2003903 (PMC6070289; doi:10.1371/journal.pbio.2003903)
Supplement: S33 Fig — Genes were grouped into 50 bins of equal size based on increasing Kozak scores of uAUGs. Median Kozak score and log2(TE) in each bin were displayed in the plots. The raw data can be found in S1 Data. CDS, coding DNA sequence; RPKM, reads per kilobase of transcript per million mapped reads; TE, translational efficiency; uAUG, start codon of upstream open reading frame. (PDF) [file pbio.2003903.s050.pdf]

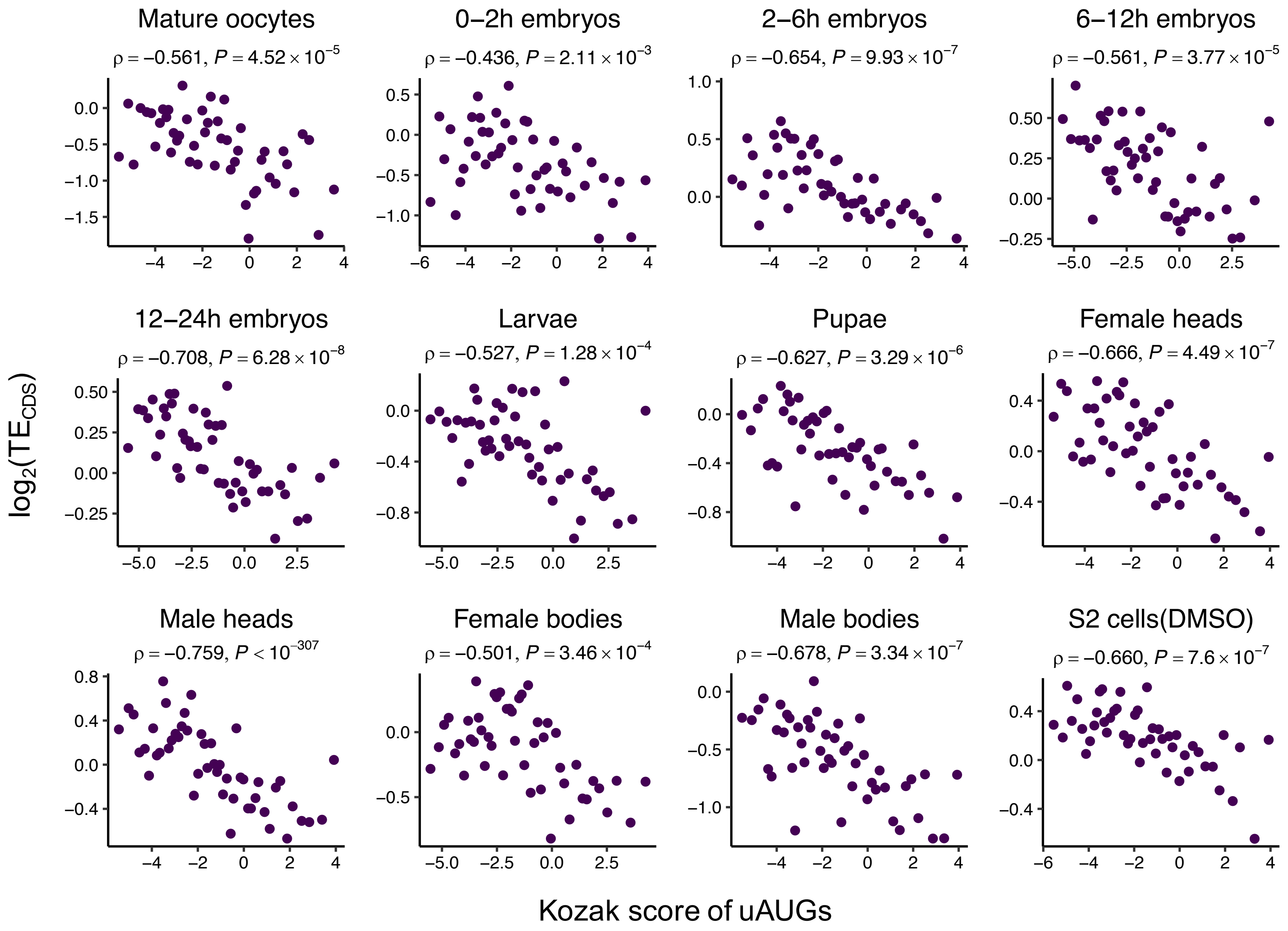

Mature oocytes
ρ = −0.561, P = 4.52 × 10−5
0–2h embryos
ρ = −0.436, P = 2.11 × 10−3
2–6h embryos
ρ = −0.654, P = 9.93 × 10−7
6–12h embryos
ρ = −0.561, P = 3.77 × 10−5
12–24h embryos
ρ = −0.708, P = 6.28 × 10−8
Larvae
ρ = −0.527, P = 1.28 × 10−4
Pupae
ρ = −0.627, P = 3.29 × 10−6
Female heads
ρ = −0.666, P = 4.49 × 10−7
Male heads
ρ = −0.759, P < 10−307
Female bodies
ρ = −0.501, P = 3.46 × 10−4
Male bodies
ρ = −0.678, P = 3.34 × 10−7
S2 cells(DMSO)
ρ = −0.660, P = 7.6 × 10−7
log2(TECDS)
Kozak score of uAUGs
