# Supplementary material for: Genome-wide maps of ribosomal occupancy provide insights into adaptive evolution and regulatory roles of uORFs during Drosophila development
Source: PLoS Biol. 2018 Jul 20;16(7):e2003903. doi: 10.1371/journal.pbio.2003903 (PMC6070289; doi:10.1371/journal.pbio.2003903)
Supplement: S34 Fig — Genes were grouped into 50 bins of equal size based on increasing uORF lengths. Median log10(uORF length) and log2(TE) in each bin were displayed in the plots. The raw data can be found in S1 Data. CDS, coding DNA sequence; RPKM, reads per kilobase of transcript per million mapped reads; TE, translational efficiency; uORF, upstream open reading frame. (PDF) [file pbio.2003903.s051.pdf]

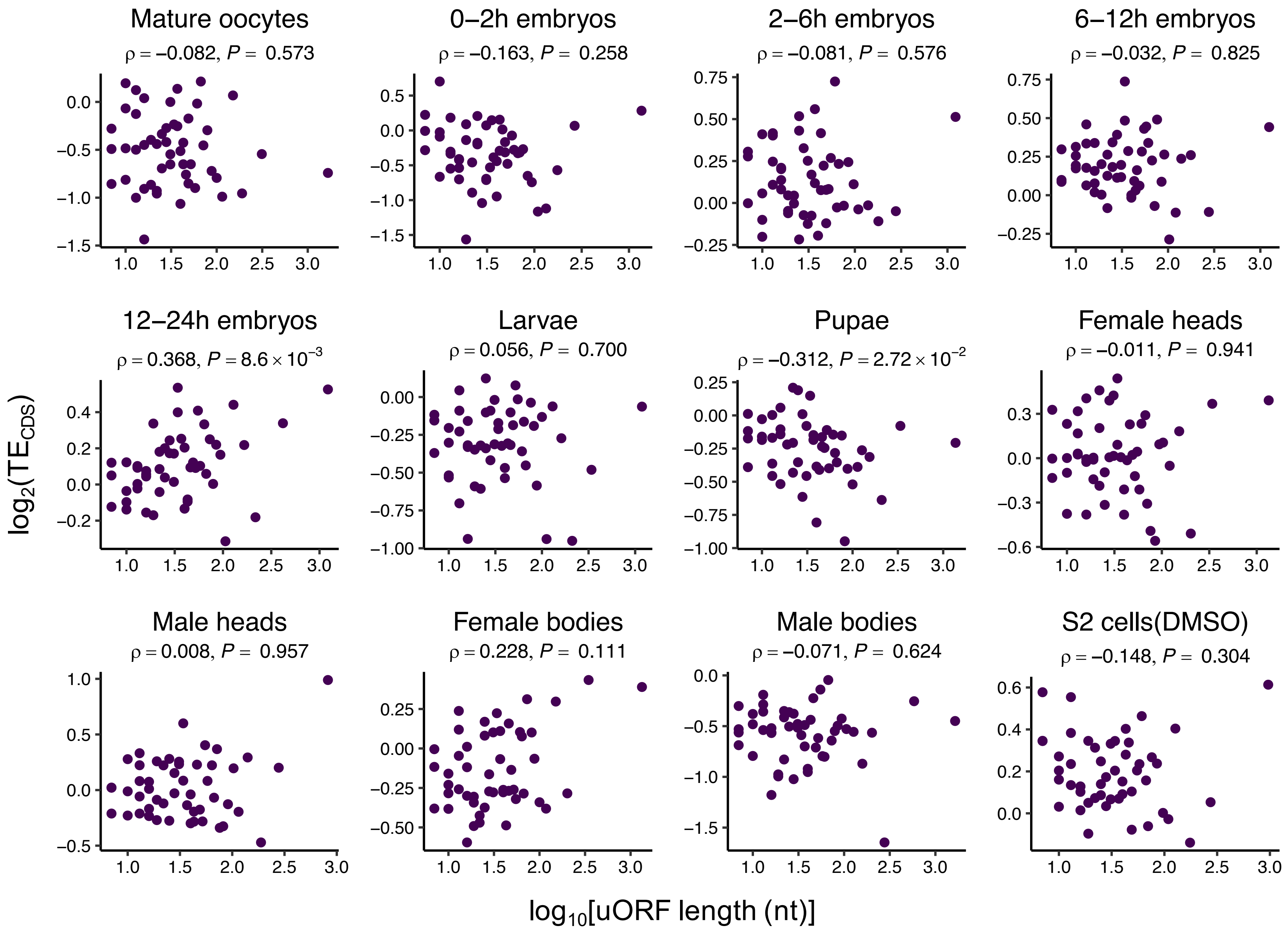

Mature oocytes
ρ = −0.082, P = 0.573
0–2h embryos
ρ = −0.163, P = 0.258
2–6h embryos
ρ = −0.081, P = 0.576
6–12h embryos
ρ = −0.032, P = 0.825
12–24h embryos
ρ = 0.368, P = 8.6 × 10^−3
Larvae
ρ = 0.056, P = 0.700
Pupae
ρ = −0.312, P = 2.72 × 10^−2
Female heads
ρ = −0.011, P = 0.941
Male heads
ρ = 0.008, P = 0.957
Female bodies
ρ = 0.228, P = 0.111
Male bodies
ρ = −0.071, P = 0.624
S2 cells(DMSO)
ρ = −0.148, P = 0.304
log2(TECDS)
log10[uORF length (nt)]
