# Supplementary material for: Genome-wide maps of ribosomal occupancy provide insights into adaptive evolution and regulatory roles of uORFs during Drosophila development
Source: PLoS Biol. 2018 Jul 20;16(7):e2003903. doi: 10.1371/journal.pbio.2003903 (PMC6070289; doi:10.1371/journal.pbio.2003903)
Supplement: S35 Fig — Genes were grouped into 50 bins based on distance from uAUG to 5′ cap. Median log10(distance from uAUG to 5′ cap) and log2(TE) in each bin were displayed in the plots. The raw data can be found in S1 Data. CDS, coding DNA sequence; RPKM, reads per kilobase of transcript per million mapped reads; TE, translational efficiency; uAUG, start codon of upstream open reading frame. (PDF) [file pbio.2003903.s052.pdf]

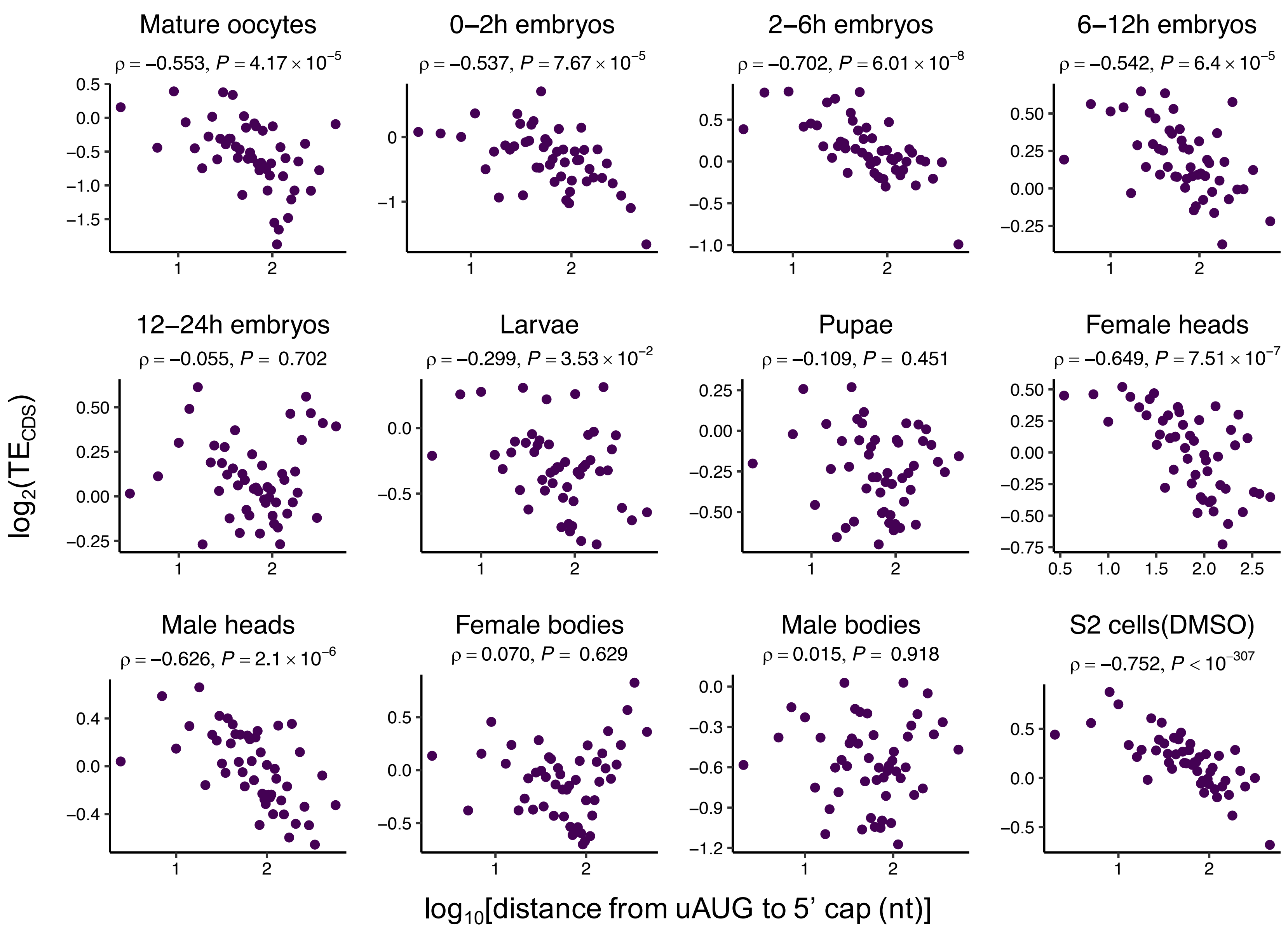
Mature oocytes
ρ = −0.553, P = 4.17 × 10^−5
0–2h embryos
ρ = −0.537, P = 7.67 × 10^−5
2–6h embryos
ρ = −0.702, P = 6.01 × 10^−8
6–12h embryos
ρ = −0.542, P = 6.4 × 10^−5
12–24h embryos
ρ = −0.055, P = 0.702
Larvae
ρ = −0.299, P = 3.53 × 10^−2
Pupae
ρ = −0.109, P = 0.451
Female heads
ρ = −0.649, P = 7.51 × 10^−7
Male heads
ρ = −0.626, P = 2.1 × 10^−6
Female bodies
ρ = 0.070, P = 0.629
Male bodies
ρ = 0.015, P = 0.918
S2 cells(DMSO)
ρ = −0.752, P < 10^−307
log2(TE_CDS)
log10[distance from uAUG to 5' cap (nt)]
