# Supplementary material for: Genome-wide maps of ribosomal occupancy provide insights into adaptive evolution and regulatory roles of uORFs during Drosophila development
Source: PLoS Biol. 2018 Jul 20;16(7):e2003903. doi: 10.1371/journal.pbio.2003903 (PMC6070289; doi:10.1371/journal.pbio.2003903)
Supplement: S37 Fig — Genes were grouped into 50 bins based on increasing phyloP scores. Median phyloP score and log2(TE) in each bin were displayed in the plots. The raw data can be found in S1 Data. CDS, coding DNA sequence; RPKM, reads per kilobase of transcript per million mapped reads; TE, translational efficiency; uAUG, AUG start codon of uORF; uORF, upstream open reading frame. (PDF) [file pbio.2003903.s054.pdf]

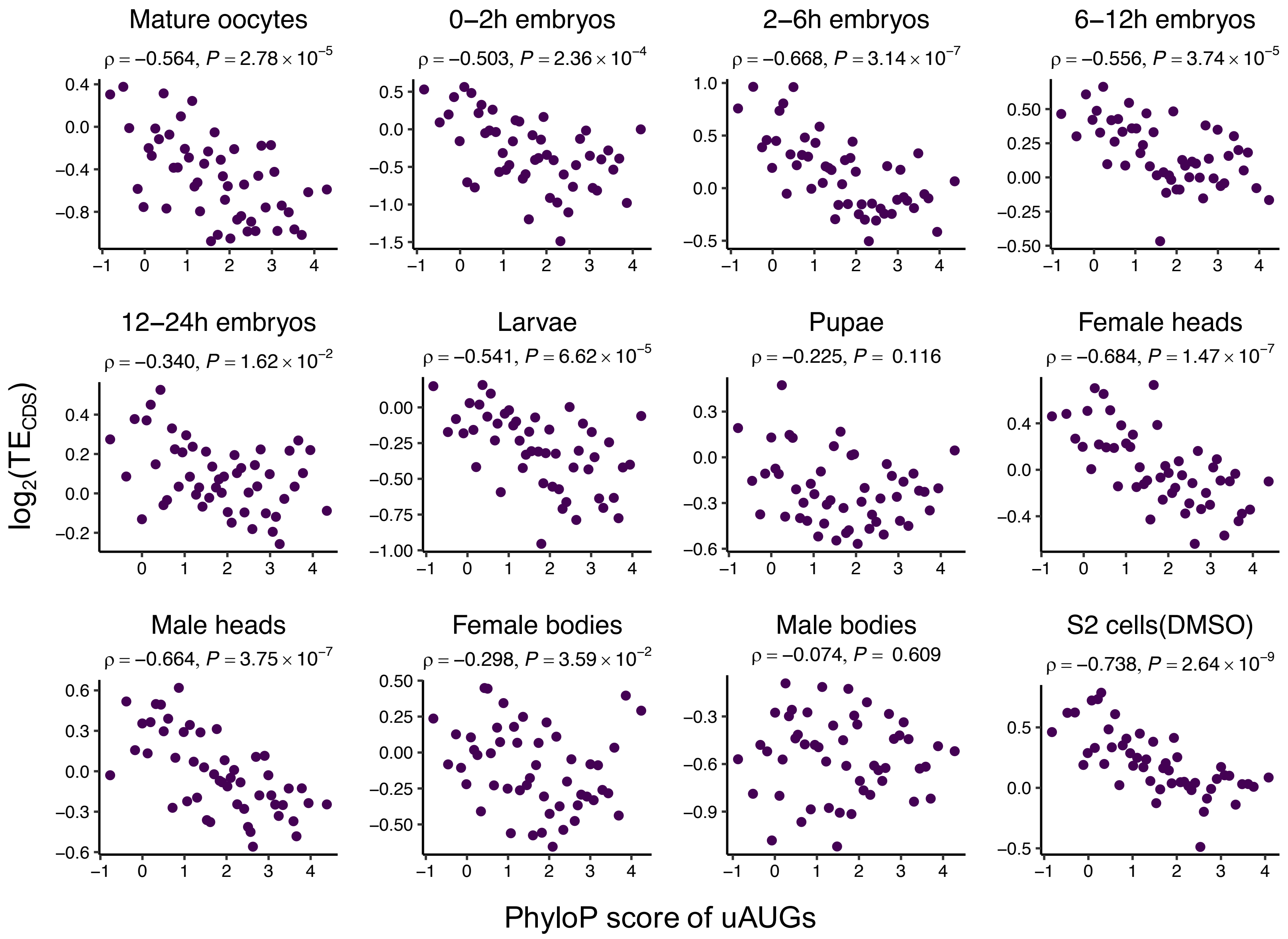

Mature oocytes
ρ = −0.564, P = 2.78 × 10^−5
0–2h embryos
ρ = −0.503, P = 2.36 × 10^−4
2–6h embryos
ρ = −0.668, P = 3.14 × 10^−7
6–12h embryos
ρ = −0.556, P = 3.74 × 10^−5
12–24h embryos
ρ = −0.340, P = 1.62 × 10^−2
Larvae
ρ = −0.541, P = 6.62 × 10^−5
Pupae
ρ = −0.225, P = 0.116
Female heads
ρ = −0.684, P = 1.47 × 10^−7
Male heads
ρ = −0.664, P = 3.75 × 10^−7
Female bodies
ρ = −0.298, P = 3.59 × 10^−2
Male bodies
ρ = −0.074, P = 0.609
S2 cells(DMSO)
ρ = −0.738, P = 2.64 × 10^−9
log2(TECDS)
PhyloP score of uAUGs
