# Supplementary material for: Genome-wide maps of ribosomal occupancy provide insights into adaptive evolution and regulatory roles of uORFs during Drosophila development
Source: PLoS Biol. 2018 Jul 20;16(7):e2003903. doi: 10.1371/journal.pbio.2003903 (PMC6070289; doi:10.1371/journal.pbio.2003903)
Supplement: S39 Fig — Genes were grouped into 50 bins based on increasing BLSs. Median uORF BLS and log2(TE) in each bin were displayed in the plots. The raw data can be found in S1 Data. BLS, branch length score; CDS, coding DNA sequence; RPKM, reads per kilobase of transcript per million mapped reads; TE, translational efficiency; uORF, upstream open reading frame. (PDF) [file pbio.2003903.s056.pdf]

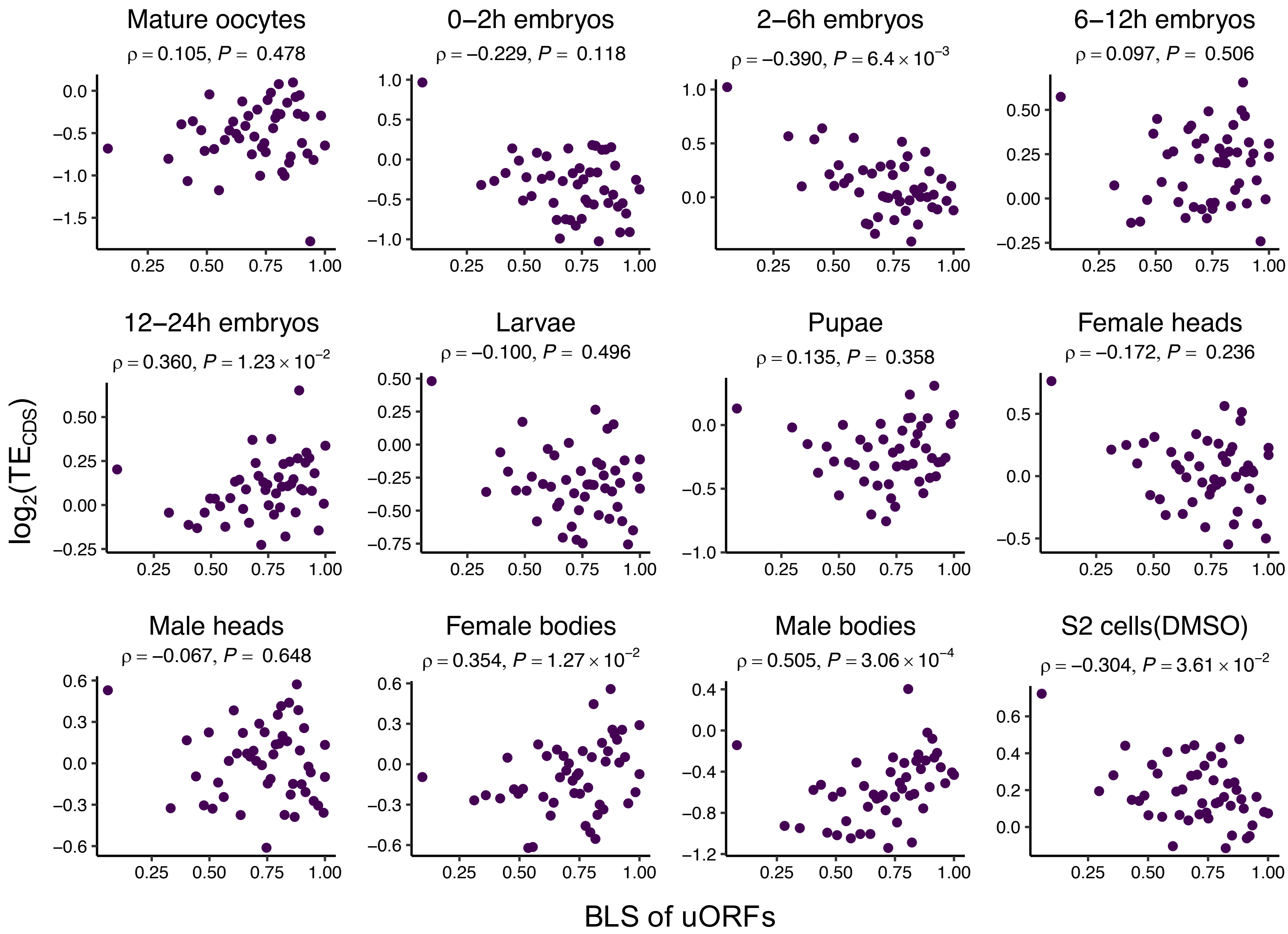

Mature oocytes
ρ = 0.105, P = 0.478
0–2h embryos
ρ = −0.229, P = 0.118
2–6h embryos
ρ = −0.390, P = 6.4 × 10^−3
6–12h embryos
ρ = 0.097, P = 0.506
12–24h embryos
ρ = 0.360, P = 1.23 × 10^−2
Larvae
ρ = −0.100, P = 0.496
Pupae
ρ = 0.135, P = 0.358
Female heads
ρ = −0.172, P = 0.236
Male heads
ρ = −0.067, P = 0.648
Female bodies
ρ = 0.354, P = 1.27 × 10^−2
Male bodies
ρ = 0.505, P = 3.06 × 10^−4
S2 cells(DMSO)
ρ = −0.304, P = 3.61 × 10^−2
log2(TECDS)
BLS of uORFs
