# Supplementary material for: Genome-wide maps of ribosomal occupancy provide insights into adaptive evolution and regulatory roles of uORFs during Drosophila development
Source: PLoS Biol. 2018 Jul 20;16(7):e2003903. doi: 10.1371/journal.pbio.2003903 (PMC6070289; doi:10.1371/journal.pbio.2003903)
Supplement: S40 Fig — For each pair of samples, the Pearson’s correlation coefficient (r) and associated P value and the total number of well-transcribed uORFs (n) were shown. The blue dashed line is the linear fit of log2(TECDS,2/TECDS,1) against log2(TEuORF,2/TEuORF,1). The red dashed line denotes where log2(TECDS,2/TECDS,1) = log2(TEuORF,2/TEuORF,1). The raw data can be found in S1 Data. CAGE, cap analysis of gene expression; CDS, coding DNA sequence; TE, translational efficiency; uORF, upstream open reading frame. (PDF) [file pbio.2003903.s057.pdf]

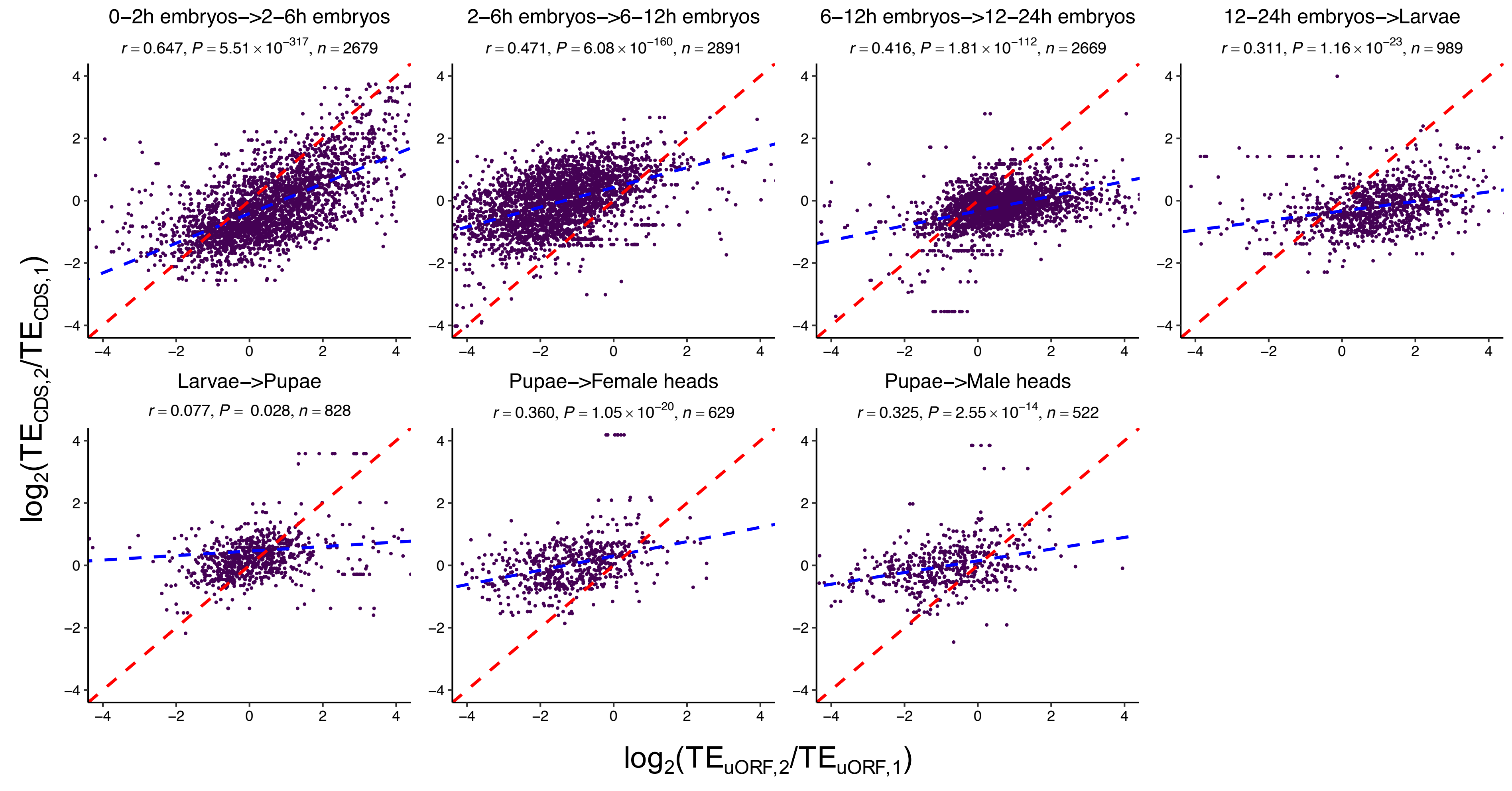
0–2h embryos–>2–6h embryos
r = 0.647, P = 5.51 × 10^−317, n = 2679
2–6h embryos–>6–12h embryos
r = 0.471, P = 6.08 × 10^−160, n = 2891
6–12h embryos–>12–24h embryos
r = 0.416, P = 1.81 × 10^−112, n = 2669
12–24h embryos–>Larvae
r = 0.311, P = 1.16 × 10^−23, n = 989
Larvae–>Pupae
r = 0.077, P = 0.028, n = 828
Pupae–>Female heads
r = 0.360, P = 1.05 × 10^−20, n = 629
Pupae–>Male heads
r = 0.325, P = 2.55 × 10^−14, n = 522
log2(TE_CDS,2/TE_CDS,1)
log2(TE_uORF,2/TE_uORF,1)
