# Supplementary material for: Genome-wide maps of ribosomal occupancy provide insights into adaptive evolution and regulatory roles of uORFs during Drosophila development
Source: PLoS Biol. 2018 Jul 20;16(7):e2003903. doi: 10.1371/journal.pbio.2003903 (PMC6070289; doi:10.1371/journal.pbio.2003903)
Supplement: S41 Fig — For a uORF and its downstream CDS in an mRNA that dominates in both sample 1 and 2, we defined γ = (TECDS,2/TECDS,1)/ (TEuORF,2/TEuORF,1) and tested whether log2(γ) is significantly different from 0. To estimate the SE of log2(γ), we modeled the log2(TE) of the uORF and the CDS in sample 1 or 2 with normal distributions. For female bodies and male bodies, we estimated the SE values of log2(TE) of a feature (CDS or uORF) by contrasting the RPF counts against the mRNA counts of biological replicates with DESeq2 [66]. For the remaining samples, the SE values were determined with the smooth surface of SE values of log2(TE) against the mRNA counts and log2(TE) of well-transcribed features in biological replicates of female and male bodies. The P value under the null hypothesis log2(γ) = 0 was obtained with a two-tailed Wald test. CDS, coding DNA sequence; RPF, ribosome-protected mRNA fragment; SE, standard error; TE, translational efficiency; uORF, upstream open reading frame. (PDF) [file pbio.2003903.s058.pdf]

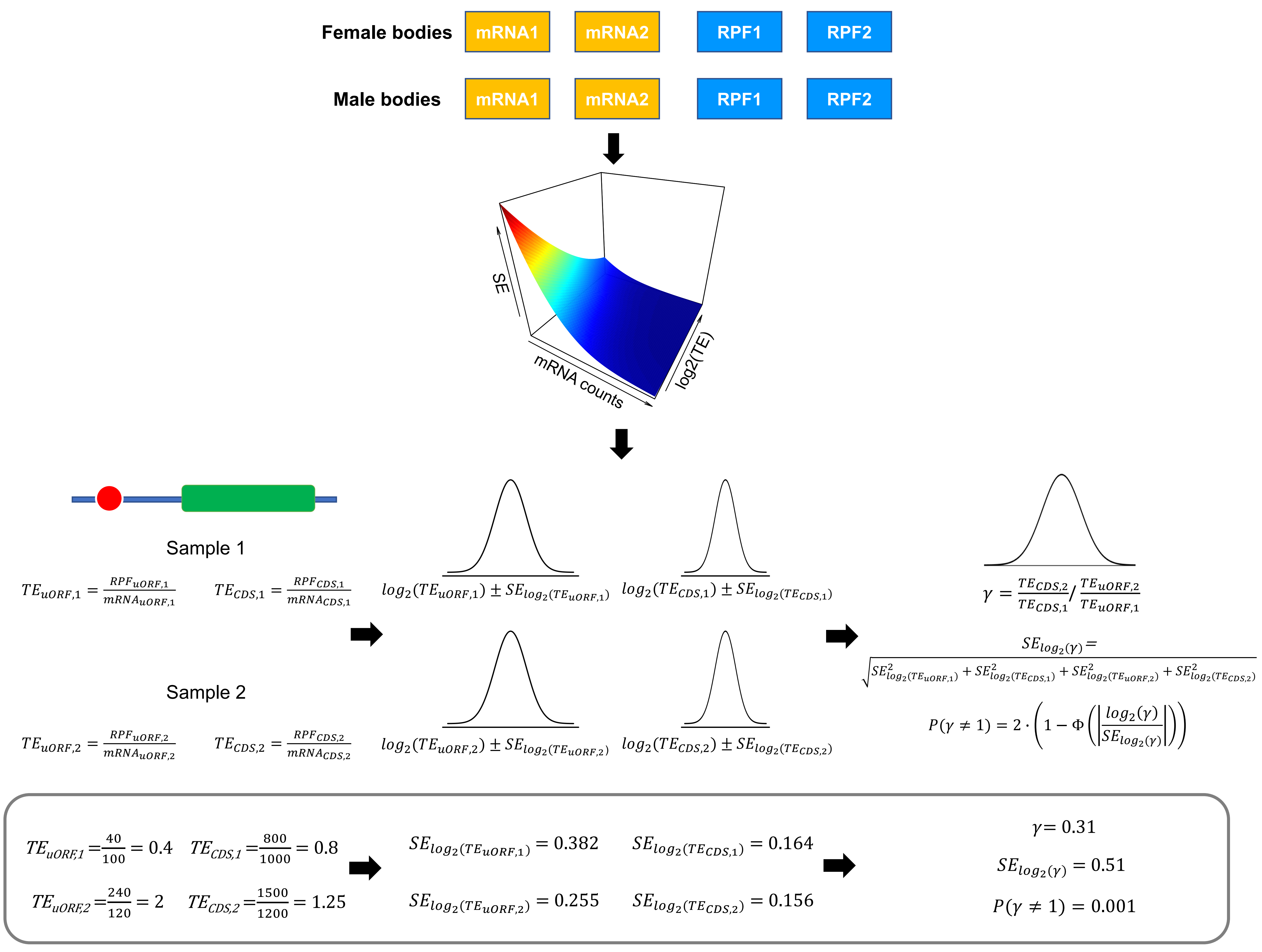
Female bodies
mRNA1
mRNA2
RPF1
RPF2
Male bodies
mRNA1
mRNA2
RPF1
RPF2
SE
mRNA counts
log2(TE)
Sample 1
$TE_{uORF,1} = \frac{RPF_{uORF,1}}{mRNA_{uORF,1}}$
$TE_{CDS,1} = \frac{RPF_{CDS,1}}{mRNA_{CDS,1}}$
Sample 2
$TE_{uORF,2} = \frac{RPF_{uORF,2}}{mRNA_{uORF,2}}$
$TE_{CDS,2} = \frac{RPF_{CDS,2}}{mRNA_{CDS,2}}$
$log_2(TE_{uORF,1}) \pm SE_{log_2(TE_{uORF,1})}$
$log_2(TE_{CDS,1}) \pm SE_{log_2(TE_{CDS,1})}$
$log_2(TE_{uORF,2}) \pm SE_{log_2(TE_{uORF,2})}$
$log_2(TE_{CDS,2}) \pm SE_{log_2(TE_{CDS,2})}$
$\gamma = \frac{TE_{CDS,2}}{TE_{CDS,1}} / \frac{TE_{uORF,2}}{TE_{uORF,1}}$
$SE_{log_2(\gamma)} = \sqrt{SE^2_{log_2(TE_{uORF,1})} + SE^2_{log_2(TE_{CDS,1})} + SE^2_{log_2(TE_{uORF,2})} + SE^2_{log_2(TE_{CDS,2})}}$
$P(\gamma \neq 1) = 2 \cdot \left(1 - \Phi\left(\left|\frac{log_2(\gamma)}{SE_{log_2(\gamma)}}\right|\right)\right)$
$TE_{uORF,1} = \frac{40}{100} = 0.4$
$TE_{CDS,1} = \frac{800}{1000} = 0.8$
$TE_{uORF,2} = \frac{240}{120} = 2$
$TE_{CDS,2} = \frac{1500}{1200} = 1.25$
$SE_{log_2(TE_{uORF,1})} = 0.382$
$SE_{log_2(TE_{CDS,1})} = 0.164$
$SE_{log_2(TE_{uORF,2})} = 0.255$
$SE_{log_2(TE_{CDS,2})} = 0.156$
$\gamma = 0.31$
$SE_{log_2(\gamma)} = 0.51$
$P(\gamma \neq 1) = 0.001$
